# Azasulfur(iv) derivatives of sulfite and sulfinate esters by formal S–S bond insertion of dichloramines†

**DOI:** 10.1039/d4sc00500g

**Published:** 2024-03-05

**Authors:** Peng Wu, Joachim Demaerel, Benjamin J. Statham, Carsten Bolm

**Affiliations:** a Institute of Organic Chemistry RWTH Aachen University Landoltweg 1a 52074 Aachen Germany Carsten.Bolm@oc.rwth-aachen.de; b Dept. of Chemistry, KU Leuven, Sustainable Chemistry for Metals and Molecules Celestijnenlaan 200F Box 2404 3001 Leuven Belgium

## Abstract

Azasulfur(vi) compounds such as sulfoximines and sulfonimidamides are attractive due to the unique properties of the S=N bond. While the synthesis of these carbon-attached sulfonimidoyl derivatives is well-established, the situation is different for their heteroatom-bound counterparts. In this work, we propose azasulfur(iv) esters as platform chemicals that can be derivatized to obtain all types of S^VI^=N functional groups, among these are the poorly accessible, all-heteroatom imidosulfate esters. Using a chloroamination workflow established here, S–S bond-containing structures such as elemental sulfur or diaryl disulfides can be transformed into imidothionyl or sulfinimidoyl chlorides, which are easily esterified or amidated. Thus, chloramines serve as a versatile [N] and [Cl^+^] source, and by using them in the context reported here, we advance the set of mild synthetic methods as the latest toolbox member to cover even more of the azasulfur(iv) and (vi) chemical space.

## Abstract 2

Starting from *N*,*N*-dichloramines and S_8_ or disulfides, azasulfur(iv) chlorides are prepared, which are easily derivatised by esterification or amidation. The resulting products can be converted to a variety of complex sulfur(vi) compounds.

## Graphical abstract

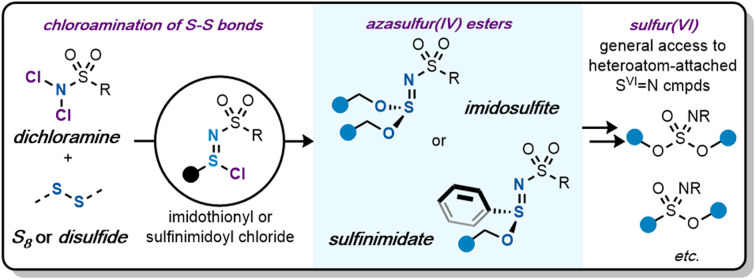

## Introduction

**Scheme 1:**
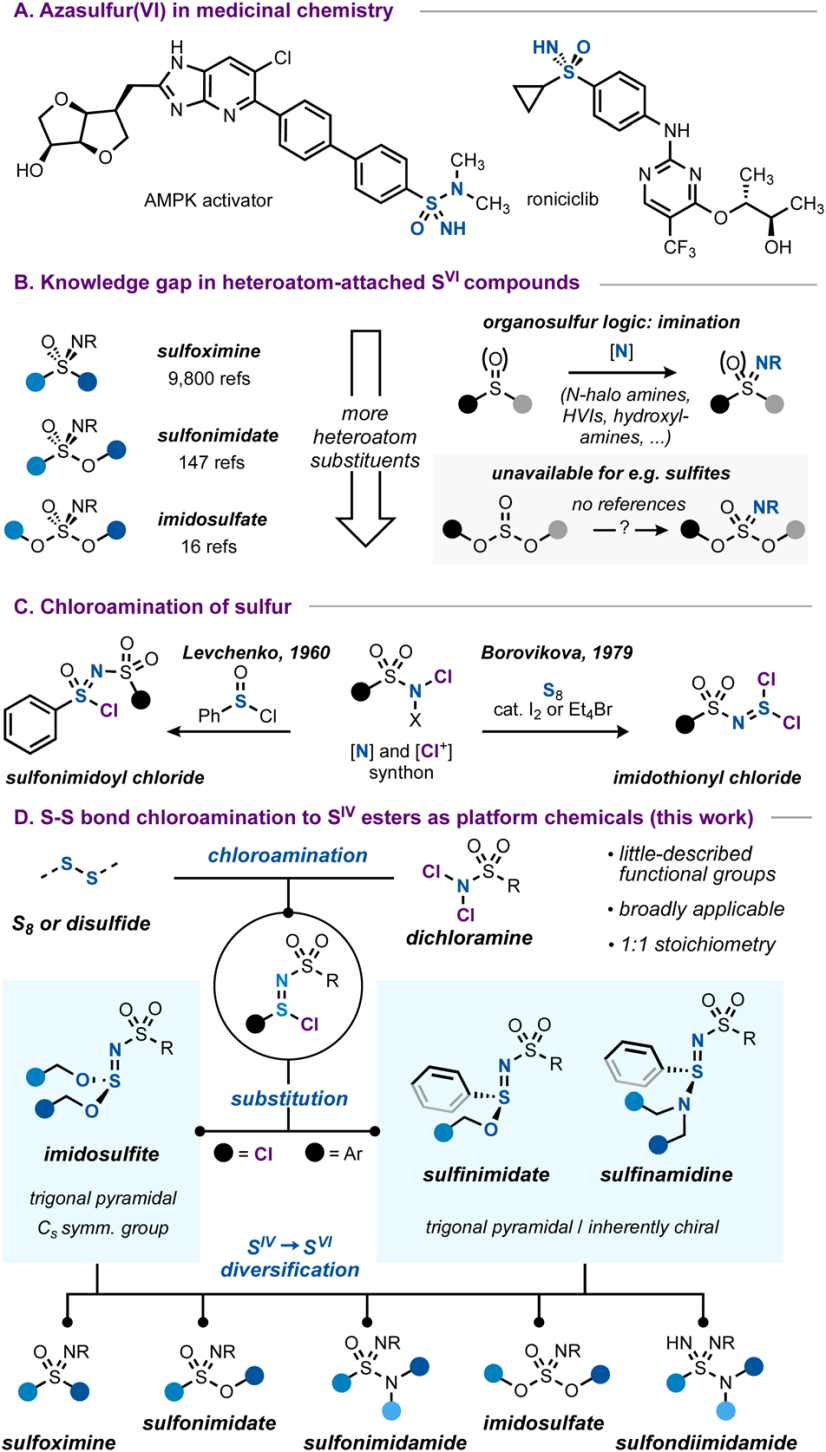
(A) Azasulfur-containing bioactive molecules. (B) Connection between the carbon-bound azasulfur(vi) literature and all-heteroatom azasulfur(vi) species. (C) Precedents in the literature on the chloroamination of sulfur to form S=N bonds. (D) This work.

Azasulfur(vi) structures, with their tetrahedral geometry and formal S=N double bond, represent a class of sulfur compounds that is gaining increasing attention.^1^ While they can be considered as variations of the more common sulfonyl unit, the nitrogen atom in the sulfonimidoyl core represents an additional point of possible functionalization and imparts considerable physicochemical differences due to its relative basicity and H-bonding ability.^2^ Combined with good hydrolytic stability, azasulfur(vi) groups such as sulfoximines or sulfonimidamides are increasingly investigated as viable options in medicinal chemistry (Scheme 1A).^2*a*,3^

While carbon-bound azasulfur(vi) structures such as sulfoximines are synthetically well-studied, the case changes when more heteroatoms are introduced. For example, considering the sulfonimidate functional group, the number of references is almost seventy-fold smaller compared to those on the topic of sulfoximines, decreasing even further for all-heteroatom imidosulfates (Scheme 1B).^4^ The number of synthetic entries to these imidosulfur species is accordingly much smaller, and the typical ‘imination’ routes^1*d*,*f*^ that work for sulfides or sulfoxides (*e.g.* transition metal-catalyzed^5^ or uncatalyzed processes with hydroxylamines,^6^ hypervalent iodinanes^7^ or *N*-halo amides^8^ as the nitrogen source) are much less translatable to the case of heteroatom-substituted substrates such as sulfinate or sulfite esters.^9,10^

A complement to this gap in the imination toolbox is therefore needed, enabling a broad access to azasulfur derivatives with non-carbon substituents. The chloroamination reaction of S–S containing species poses a solution to this lack of synthetic routes.

The chloroamination approach to azasulfur preparation finds precedence in the work of Levchenko, who used chloramine T to prepare sulfonimidoyl chlorides (Scheme 1C).^11^ Later, the same authors reported that chloroamination of elemental sulfur led to imidothionyl chlorides, reactive azasulfur(iv) chemicals with bis(electrophile) potential.^12^ Imidothionyl halides have been known since the 1960s^13^ and important work by Shreeve,^14^ Tisnes,^15^ Mews,^16^ and especially by many scientists at the Ukrainian N.A.S.^12,17^ has greatly expanded the known realm of azasulfur(iv) mono- and dihalides and their applications. Intriguingly, these studies have largely remained limited to the synthesis of S^IV^ compounds, and it seems that little investigation has been made into their usage as general routes to azasulfur(vi) compounds.^18^

In this work, we aim to develop a broadly applicable chloroamination of sulfur and disulfides, as a general means to obtain heteroatom-attached azasulfur compounds. We reasoned that dichloramines, as stable and often crystalline N–Cl compounds, can serve ideally as an integrated [Cl^+^] and nitrogen source, capable of being inserted by low-oxidation-state sulfur species.^19^ The resulting sulfur(iv) chlorides with the ‘sulfinimidoyl’ core can then be transformed into imidosulfites,^14*a*,*b*,15,17*b*^ sulfinimidates^6*a*,20^ or sulfinamidines.^6*a*,8*d*,20*d*,21^ Such compounds are stable, but little-described, and their facile preparation from commercial starting materials contrasts the typical routes to, for example, sulfinimidates, which commonly start from pre-synthesized sulfenamides or sulfinylamines.^22^ A variety of these S=N derivatives is prepared to explore the synthetic breadth of these functional groups. Ultimately, we demonstrate how azasulfur(iv) compounds can serve as a strategic choice for platform chemicals, enabling access to all classes of heteroatom-attached azasulfur(vi) species.

## Results and discussion

We started by focusing our attention on imidothionyl chlorides. Reasoning that these structures are isoelectronic to thionyl chloride, we surmised that they could serve as precursors to imidosulfite esters, which in turn could be transformed into downstream S^VI^ all-heteroatom derivatives. To this end, we set out to optimize the chloroamination of elemental sulfur using a commercial dichloroamine, *N*,*N*-dichlorotoluenesulfonamide (dichloramine T, 1).^12^ By trapping the formed tosylimidothionyl chloride with the preformed disodium salt of neopentylene glycol, various conditions were tested to attain the highest amount of the ester product 3v (Table 1). It was found that the TBAB-catalyzed reaction between TsNCl_2_ and elemental sulfur in a 1 : 1 ratio in 1,2-DCE was ideal, resulting in an initial 76% yield of 3v after 1 h (Table 1, entry 1). Other chlorinated or aromatic solvents (Table 1, entry 2) or phase-transfer catalysts (Table 1, entry 3) as well as a shortening of the reaction times to only 5 min proved less effective (Table 1, entry 4). Desiccants did not improve the yield (Table 1, entry 5). In the absence of light, however, the yield of 3v increased to 81%. The same outcome resulted when the diol was simply added to the imidothionyl chloride mixture along with Et_3_N in the same solvent (Table 1, entry 7). The latter conditions constitute the final, optimized conditions.

**Table 1:** Optimization of the chloroamination of elemental sulfur and subsequent esterification

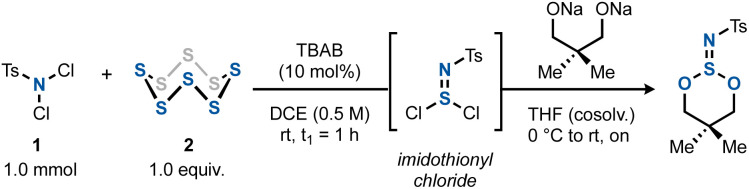		
Entry	Deviation from standard conditions	Yield of 3v^a^ (%)
1	None	76
2	Toluene or CHCl_3_ as solvent	52–69
3	Other Br^−^ or I^−^ catalysts	<63
4	*t*_1_ = 5 min	63
5	Added desiccants	<43
6	In the dark	81
7	Diol (1.1 equiv.) + Et_3_N (2.2 equiv.) in DCE for 30 min	**81** b

a ^1^H NMR yield relative to the internal standard.

b After chromatography.

**Scheme 2:**
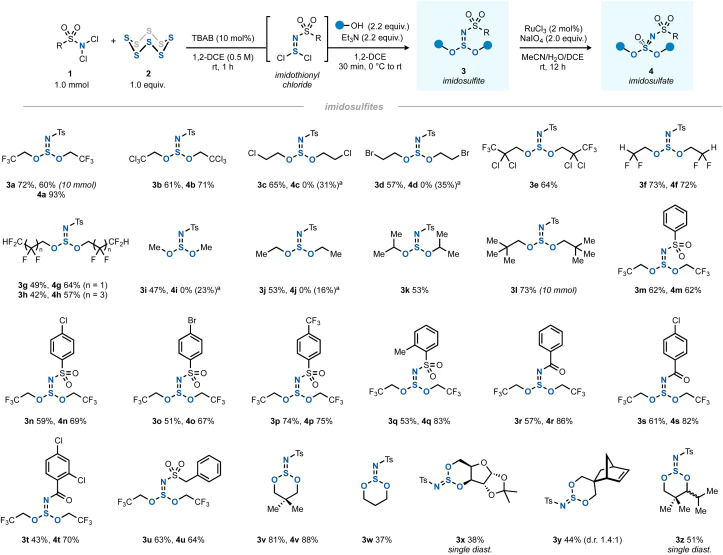
The scope of synthesis of imidosulfites 3 (1.0 mmol scale) and imidosulfates 4 (0.2 mmol scale). ^*a*^The desired imidosulfate was not observed. Instead, *N*-tosyl-*N*,*O*-dialkylsulfamate was isolated (yield between parentheses), see ESI Sections 2.11 and 2.12† for details.

The optimal two-step one-pot method was then investigated as a general route to various imidosulfites (Scheme 2, products 3) and imidosulfate esters (products 4). The latter S(vi) compounds were obtained cleanly by a RuCl_3_/NaIO_4_-mediated oxidation step.^23,24^ Being inspired by Fox's work, where fluoroalkyl sulfite esters had shown a remarkable stability,^25,26^ the investigation of the alcohol scope of the reaction focused initially on halogenated alcohols. Indeed, polyhalogenated alcohols gave good yields of the imidosulfites 3a–h, which underwent the subsequent oxidation efficiently in most cases. In comparison, methanol, ethanol and isopropanol gave mediocre yields of products 3i–k. *tert*-Butanol did not react.^27^ We assume that the enhanced stability of polyhalogenated alkyl (and by extension, neopentyl) S^IV^ esters is due to an increased hydrophobicity, greater steric shielding of the sulfur core, the absence of β-protons, or an interplay of these factors. A side note should be added on the oxidation of less halogenated alkyl imidosulfite esters (3c, 3d, 3i, and 3j), where the corresponding imidosulfates were not formed. Instead, they gave rise to different oxidation products, which we believe to be the respective *N*-tosyl-*N*,*O*-dialkylsulfamates (see ESI Sections 2.11 and 2.12†).^28^ It is conceivable that the corresponding imidosulfate oxidation products 4 were formed initially, but, being good alkylating agents (comparable to, for example, Me_2_SO_4_), they became engaged in a rearrangement to form isomeric *N*-alkylated sulfamates. A similar *O*-to-*N* transfer has been reported for *O*-alkylsulfonimidates by Maricich.^29^ Interestingly, the other imidosulfites, bearing alkyl groups with a higher degree of halogenation, did not show such side reactivity. In addition, for bulky 3e and 3k neither the desired products 4 nor the rearranged side-products were obtained.

Moving away from *N*-Ts, several other arenesulfonyl (3m–q) and benzoyl (3r–t) protecting groups as well as an aliphatic sulfonyl group (3u) worked well. In all cases, the oxidation yields were good (4m–u). Several cyclic derivatives were prepared by using 1,3-diols as bis(nucleophiles) (3v–z). Five-membered cyclic sulfites (*e.g.* from ethylene glycol) could not be isolated, possibly due to their well-documented hydrolytic instability.^30^

Due to the trigonal pyramidal geometry of the imidosulfite moiety, the sulfur atom acts as a rigid stereocenter in the six-membered ring. Derivatives with a distal carbon stereocenter such as 3y therefore gave rise to a mixture of diastereomers. When the stereogenic centers were closer together, a single diastereomer was obtained (3x and 3z). The case of 3z indicates that the ^*i*^Pr-attached carbon stereocenter most likely dictates the orientation of the azasulfur bond. While usually a *cis*-1,3-disubstituted cyclohexane with both substituents in the equatorial position is the more favourable geometry, a different situation was found for cyclic imidosulfites. As previously described by Tisnes, the anomeric effect (lone pair–lone pair repulsion between heteroatoms) favors the imido nitrogen in the axial position of the ring.^15*a*^ Indeed, calculations on the DFT level (B3LYP/def2-TZVPP//B3LYP/def2-SVP) indicated a 3.7 kcal mol^−1^ free energy benefit for *trans*-3z relative to *cis*-3z, which was roughly between the values for the steric strain that the ^*i*^Pr and ^*t*^Bu groups experience in the axial position of monosubstituted cyclohexanes.^31^

**Scheme 3:**
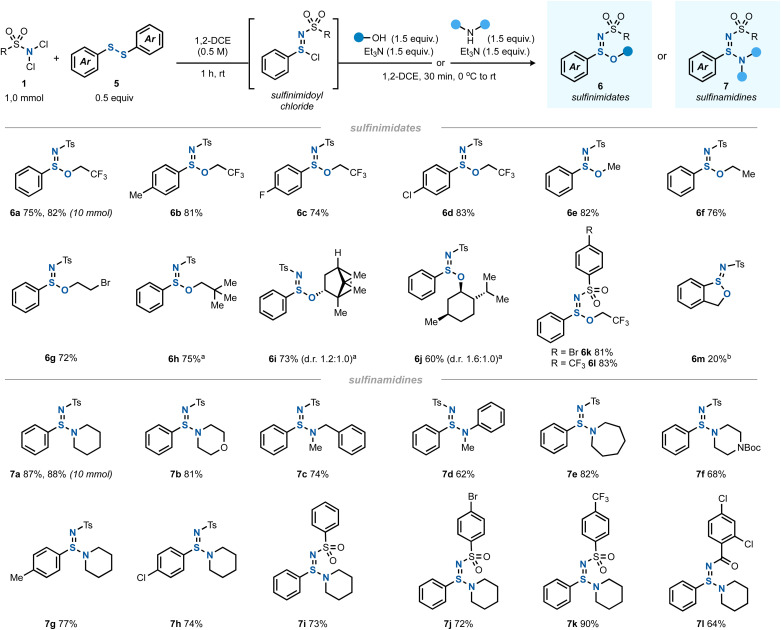
The scope of synthesis of sulfinimidates 6 (1.0 mmol scale) and sulfinamidines 7 (1.0 mmol scale). ^*a*^Use of 2.2 equiv. of alcohol and 2.2 equiv. of Et_3_N instead of the indicated quantities. ^*b*^Use of 2-mercaptobenzyl alcohol as both the sulfur source and alcohol. See the ESI† for all details.

Having thoroughly investigated the imidothionyl chloride synthesis by a formal chloroamination of elemental sulfur, we hypothesized that this chemistry could be extended to other types of S–S bonds. Indeed, when using a diaryl disulfide as the sulfur source instead of S_8_ and a 2 : 1 ratio of dichloramine 1 to disulfide 5, a clean conversion to the corresponding sulfinimidoyl chloride was found (Scheme 3).^32^ Interestingly, this reaction proceeded catalyst-free without a diminished yield.

The formed sulfinimidoyl chlorides could be reacted – in analogy to imidothionyl chlorides – with an alcohol/Et_3_N mixture to give sulfinimidate esters 6 (Scheme 3). With trifluoroethanol, various diaryl disulfides gave rise to the desired products 6a–d. A limitation was found for dialkyl disulfides (see ESI Section 2.13†).

Other primary alcohols afforded the corresponding sulfinimidate esters (6e–h) in good yields, too. Interestingly, halogenated and other aliphatic alcohols reacted equally well and the yields of the resulting sulfinimidate esters 6 were comparable. The chiral secondary alcohols (+)-borneol (6i) and (−)-menthol (6j) led to nonequal mixtures of diastereomers, indicating a stereoinduction to some degree by the existing chiral center. Other *N*,*N*-dichlorosulfonamides underwent the reaction equally well to give 6k and 6l. In an intramolecular variant, 2-mercaptobenzyl alcohol was cyclized to give benzoxathiolane 6m albeit in only 20% yield.

Apart from alcohols, amines also served as capable nucleophiles to trap the *in situ* generated sulfinimidoyl chlorides giving sulfinamidines 7 (Scheme 3). A variety of secondary amines underwent the reaction in good yields, including cyclic (to give 7a, 7b, 7e, and 7f), acyclic (leading to 7c) and aromatic amines (providing 7d). The variation of the disulfide or dichloramine reaction partners posed no problem and sulfinamidines 7g and 7h as well as 7i–l were obtained in good yields.

**Scheme 4:**
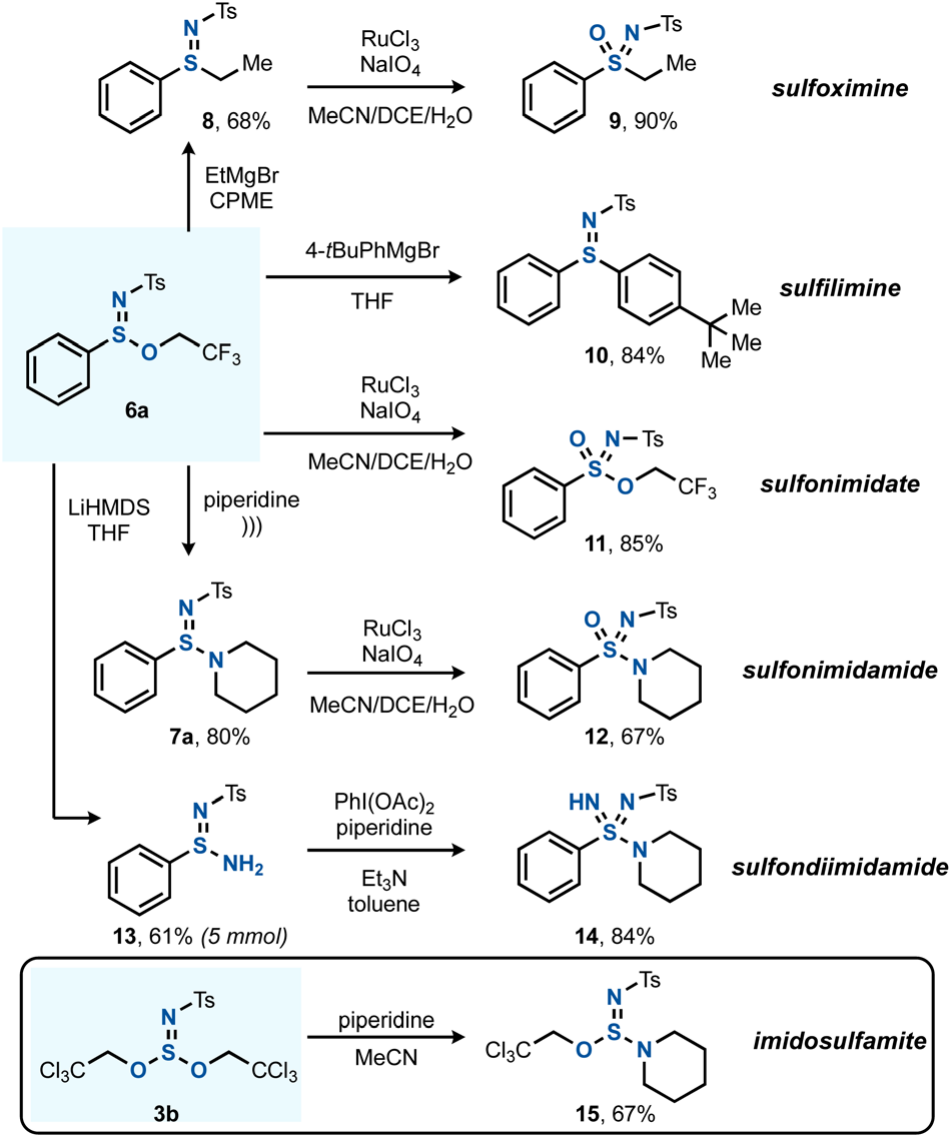
Diversification to various S^IV^ and S^VI^ functionalities. See the ESI† for full experimental details.

The next goal was to apply the S^IV^ esters and amines formed by the chloroamination method to the preparation of complex azasulfur(vi) derivatives (Scheme 4). Sulfinimidate 6a was taken as a platform to explore various post-transformations, such as the C–S bond forming substitution reaction with EtMgBr to give arylalkyl sulfilimine 8,^20*c*^ which could then be oxidized by a known Ru-catalyzed protocol to sulfoximine 9.^23^ The reaction of 6a with 4-*tert*-butylphenylmagnesium bromide gave rise to diaryl sulfilimine 10. The direct oxidation of 6a yielded sulfonimidate 11, and a sonochemical method that is known for the transamidation of sulfinate esters was successful for the preparation of 7a.^33^ In an alternative derivatization, the free sulfinamidine 13 could be obtained from the reaction of 6a with LiHMDS.^34^ Applying 13 in a subsequent S–N bond forming reaction using Willis' PhI(OAc)_2_-mediated synthesis gave sulfondiimidamide 14.^35^ In addition, also imidosulfite ester 3b could undergo (mono)substitution, giving imidosulfamite 15 after reaction with piperidine. An analogous reaction between 3b and PhONa remained unsuccessful.

## Conclusions

In conclusion, this work details a general workflow for synthesizing *N*-protected azasulfur(iv) esters and amides. From a lack of routes to heteroatom-attached structures with a ‘sulfinimidoyl’ (S=NR) core, the chloroamination of S–S bonds was established to address the need for a general entry to these underdeveloped compound classes. The reactive intermediates formed, either imidothionyl or sulfinimidoyl chlorides, function as versatile electrophiles that could be efficiently trapped with alcohols or amines as nucleophiles. A special role is withheld for halogenated alcohols, which impart high stability to the class of imidosulfite esters. This method has the advantages of using an ideal stoichiometry between commercial sulfur sources (elemental S or disulfides), and stable dichloramines as both oxidant and nitrogen sources. Importantly, the azasulfur(iv) derivatives obtained here are shown to be strategic platform chemicals to a wide variety of azasulfur(vi) compounds, further enabling the increasing trend toward their investigation as novel pharmacophores.

## Data availability

Raw NMR data are available from the corresponding author upon request.

## Author contributions

J. D. designed the study, P. W. and J. D. conducted the experiments on the synthesis of starting materials, optimization of conditions, substrate exploration, and product transformation. B. J. S. performed the DFT NMR calculations. J. D. wrote the initial version of the manuscript, and P. W. drafted the ESI.† C. B. directed the project and prepared the final draft of the manuscript. All authors have given approval to the final version of the manuscript.

## Conflicts of interest

The authors declare no conflict of interest.

## Supplementary Material

SC-015-D4SC00500G-s001

## References

[cit1] Mäder P., Kattner L. (2020). J. Med. Chem..

[cit2] Chinthakindi P. K., Naicker T., Thota N., Govender T., Kruger H. G., Arvidsson P. I. (2017). Angew. Chem., Int. Ed..

[cit3] Izzo F., Schäfer M., Lienau P., Ganzer U., Stockman R., Lücking U. (2018). Chem.–Eur. J..

[cit4] Based on a SciFinder search, 2023

[cit5] García Mancheño O., Bolm C. (2007). Chem.–Eur. J..

[cit6] Andresini M., Spennacchio M., Romanazzi G., Ciriaco F., Clarkson G., Degennaro L., Luisi R. (2020). Org. Lett..

[cit7] Luisi R., Bull J. A. (2023). Molecules.

[cit8] Marzinzik A. L., Sharpless K. B. (2001). J. Org. Chem..

[cit9] Two examples were found for imination reactions on sulfinate esters as starting materials (see ref. 10) and no examples were found for organic sulfites

[cit10] Tota A., St John-Campbell S., Briggs E. L., Estévez G. O., Afonso M., Degennaro L., Luisi R., Bull J. A. (2018). Org. Lett..

[cit11] Levchenko E. S., Derkach N. Y., Kirsanov A. V. (1960). Zh. Obshch. Khim..

[cit12] Borovikova G. S., Levchenko E. S., Borovik E. I. (1979). Zh. Org. Khim..

[cit13] Smith W. C., Tullock C. W., Smith R. D., Engelhardt V. A. (1960). J. Am. Chem. Soc..

[cit14] Abe T., Shreeve J. M. (1980). Inorg. Chem..

[cit15] Tisnes P., Picard C., Bastide J. D., Zedde C., Cazaux L., Maroni P., Trinquier G. (1983). Spectrochim. Acta, Part A.

[cit16] Mews R., Glemser O. (1972). Inorg. Chem..

[cit17] Levchenko E. S., Sheinkman I. E. (1966). Zh. Obshch. Khim..

[cit18] Maryanoff B. E., Costanzo M. J., Nortey S. O., Greco M. N., Shank R. P., Schupsky J. J., Ortegon M. P., Vaught J. L. (1998). J. Med. Chem..

[cit19] Koval' I. V. (2001). Russ. J. Org. Chem..

[cit20] Tsuzuki S., Kano T. (2023). Angew. Chem., Int. Ed..

[cit21] Ferry A., Billard T., Langlois B. R., Bacqué E. (2008). J. Org. Chem..

[cit22] Andresini M., Colella M., Degennaro L., Luisi R. (2023). Org. Biomol. Chem..

[cit23] Veale H. S., Levin J., Swern D. (1978). Tetrahedron Lett..

[cit24] Other S(iv) → S(vi) oxidation methods were screened, involving mCPBA, oxone, KMnO_4_, SelectFluor, peroxysulfonate, OsO_4_ or MeReO_3_ as the oxidizing species, but proved ineffective

[cit25] De Marco R. A., Kovacina T. A., Fox W. B. (1975). J. Fluorine Chem..

[cit26] As an example of hydrolytic and thermal stability, Fox reports that bis(trifluoroethyl) sulfite remained completely intact when subjected to water at 125 °C for 16 h, in contrast to dialkyl sulfites which decompose fully at lower temperatures

[cit27] Other failed nucleophiles include phenol, HOBt, morpholine, and aniline, which may be due to either product stability or the destruction of TsN = SCl_2_ into unknown compounds (see ESI Section 2.13†)

[cit28] The characterization data match very well with the expected spectral data of the proposed sulfamate products. While no alternative chemical synthesis towards these structures was successful, we additionally performed DFT-level predictions of the ^1^H and ^13^C NMR chemical shifts, and the high similarity between predicted and experimental NMR spectra further corroborated the identity of the sulfamate product (see ESI Section 2.12†)

[cit29] Maricich T. J., Jourdenais R. A., Albright T. A. (1973). J. Am. Chem. Soc..

[cit30] Bristow P. A., Tillett J. G., Wiggins D. E. (1968). J. Chem. Soc. B.

[cit31] Klein D. R. , Organic Chemistry, Wiley, Hoboken, NJ, 4th edn, 2021

[cit32] Levchenko E. S., Budnik L. V., Dubinina T. N. (1978). Zh. Org. Khim..

[cit33] García Ruano J. L., Parra A., Marzo L., Yuste F., Mastranzo V. M. (2011). Tetrahedron.

[cit34] García Ruano J. L., Alemán J., Belén Cid M., Parra A. (2005). Org. Lett..

[cit35] Zhang Z.-X., Bell C., Ding M., Willis M. C. (2022). J. Am. Chem. Soc..

