# Supplementary material for: Azasulfur(iv) derivatives of sulfite and sulfinate esters by formal S–S bond insertion of dichloramines
Source: Chem Sci. 2024 Mar 5;15(14):5333–9. doi: 10.1039/d4sc00500g (PMC10988629; doi:10.1039/d4sc00500g)

Electronic Supplementary Material (ESI) for Chemical Science.
This journal is © The Royal Society of Chemistry 2024



**Supporting Information**

for

# Azasulfur(IV) derivatives of sulfite and sulfinate esters by formal S–S bond insertion of dichloramines

Peng Wu,‡[a] Joachim Demaerel,‡[a,b] Benjamin J. Statham[a] and Carsten Bolm*[a]

[a] *Institute of Organic Chemistry, RWTH Aachen University, Landoltweg 1, 52074 Aachen, Germany; *E-mail: Carsten.Bolm@oc.rwth-aachen.de*

[b] *Sustainable Chemistry for Metals and Molecules, Dept. of Chemistry, KU Leuven, Celestijnenlaan 200F box 2404, 3001 Leuven, Belgium*

‡ These authors contributed equally.

## Table of Contents

1. General information S2
2. Experimental section S2
   2.1 General procedure for the preparation of dichloroamines S2
   2.2 Optimization of the imidosulfite synthesis S2
   2.3 General procedure for the preparation of imidosulfites S4
   2.4 General procedure for the preparation of sulfinimidates and sulfinamidines S4
   2.5 General procedure for the preparation of S(VI) with $NaIO_4$/$RuCl_3$ S5
   2.6 Procedure for the preparation of *N*-Ts sulfilimines with Grignard reagents S5
   2.7 Procedure for the preparation of **7a** using ultrasound S6
   2.8 Procedure for the preparation of $NH_2$-sulfinamidine **13** S6
   2.9 Procedure for the preparation of sulfondiimidamide **14** with $PhI(OAc)_2$ S7
   2.10 Procedure for the preparation of imidosulfamite **15** S7
   2.11 Note on the formation of side products during the imidosulfite oxidation S7
   2.12 NMR chemical shift predictions S8
   2.13 The summary of failed examples S13
3. Characterization data S14
4. References S45
5. NMR spectra S48

## 1. General information

Unless otherwise noted, the materials were purchased from commercial suppliers and used without further purification. All the solvents were treated according to general methods. The reactions were monitored by thin layer chromatography (TLC) with aluminium sheets silica gel 60 F254 from Merck, and flash column chromatography purifications were performed using silica gel 60 (40-63 μm) from Merck. $^1$H,$^{13}$C{$^1$H}, and $^{19}$F NMR spectra were recorded with an Agilent VNMRS 600 Agilent or VNMRS 400 in deuterated solvents. Chemical shifts (*δ*) are reported in parts per million (ppm) and spin-spin coupling constants (*J*) are given in Hz, Multiplicities are recorded as: s = singlet, d = doublet, t = triplet, q = quartet, dd = doublet of doublets, br = broad, m = multiplet. The IR spectra were recorded with a PerkinElmer Spectrum 100 spectrometer with an attached UATR device Diamond KRS-5. All IR data were collected by attenuated total reflectance (ATR) and wavenumbers n are given in cm$^{-1}$. Mass spectra were recorded with a Finnigan SSQ Finnigan 7000 spectrometer (EI, 70 eV). High resolution mass spectra (HRMS) were recorded on a Thermo Scientific LTQ Orbitrap XL spectrometer.

## 2. Experimental section

### 2.1 General procedure for the preparation of dichloroamines

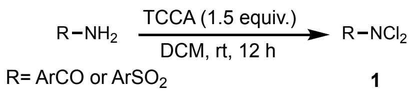


The procedure was based on a literature protocol:[S1] A mixture of primary sulfonamide or primary amide (10 mmol), TCCA (trichloroisocyanuric acid) (15 mmol), and DCM (40 mL) in a 100 mL round bottom flask was stirred at room temperature for 12 h. Upon completion, the flask was transferred to a 0 °C ice bath for 10 min, then, the corresponding dichloroamine **1** was obtained after filtration at 0 °C and concentration.

### 2.2 Optimization of the imidosulfite synthesis

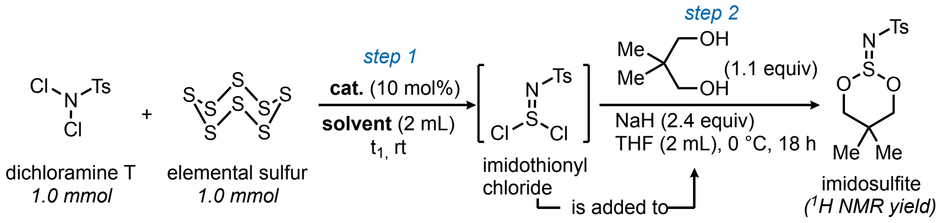

A 10 mL reaction tube was charged with dichloramine T (240 mg, 1.0 mmol), elemental sulfur (sublimed grade, 32 mg, 1.0 mmol), and a dry solvent (2 mL). This suspension was bubbled with $N_2$ for 5 min. Then, the catalyst (0.1 mmol) was added, and the reaction was stirred under $N_2$ at room temperature for time $\mathbf{t_1}$. In a second, separate tube, neopentyl glycol (115 mg, 1.1 equiv) was dissolved in dry THF (2 mL), and to this was added NaH (60% in oil, 100 mg, 2.5 equiv). After stirring under $N_2$ for 15 min at room temp., this tube was brought into an ice bath and when the reaction time $t_1$ had passed, the imidothionyl chloride solution (the content of tube 1) was added to the THF solution over 10 min, while stirring at 0 °C. The reaction mixture was allowed to warm to room temperature overnight. Next day, the internal standard was added, and a sample of the reaction was dissolved in $CDCl_3$, filtered to remove solids and analyzed by $^1H$ NMR spectroscopy.

**Table S1** Optimization of the imidothionyl chloride (step 1) and imidosulfute (step 2) formation.

| Entry | Solvent | Catalyst | $t_1$ | Notes | NMR yield [%][a] |
|---|---|---|---|---|---|
| 1 | PhH | TBAB | 1 h | | 49 |
| 2 | toluene | TBAB | 1 h | | 52 |
| 3 | PhF | TBAB | 1 h | | 30 |
| 4 | 1,2-DCE | TBAB | 1 h | | **76** |
| 5 | DCM | TBAB | 1 h | | 8 |
| 6 | $CHCl_3$ | TBAB | 1 h | | 69 |
| 7 | 1,2-DCE | CTAB | 1 h | | 63 |
| 8 | 1,2-DCE | TEAB | 1 h | | 41 |
| 9 | 1,2-DCE | $BnEt_3NBr$ | 1 h | | 42 |
| 10 | 1,2-DCE | TBAI | 1 h | | 39 |
| 11 | 1,2-DCE | $I_2$ | 1 h | | 35 |
| 12 | 1,2-DCE | DABCO | 1 h | | 43 |
| 13 | 1,2-DCE | TBAB | 5 min | | 63 |
| 14 | 1,2-DCE | TBAB | 3 h | | 76 |
| 15 | 1,2-DCE | TBAB | 1 h | 1 mol% of cat. | 53 |
| 16 | 1,2-DCE | TBAB | 1 h | 20 mol% of cat. | 60 |
| 17 | 1,2-DCE | TBAB | 1 h | $CaCl_2$ added | 43 |
| 18 | 1,2-DCE | TBAB | 1 h | 4Å MS added | 34 |
| 19 | 1,2-DCE | TBAB | 1 h | step 1 @ 40°C | 75 |
| 20 | 1,2-DCE | TBAB | 1 h | in the dark | **81** |
| 21 | 1,2-DCE | TBAB | 1 h | **step 2**: diol (1.1 equiv) + $Et_3N$ (2.2 equiv) in 1,2-DCE as solvent for 30 min | **81** (isol.) |

[a] The quantification was done by $^1H$ NMR spectroscopy, using 4-fluoroanisole as internal standard. TBAB = tetra-*n*-butylammonium bromide, CTAB = cetyl trimethylammonium bromide, TEAB = tetraethylammonium bromide, TBAI = tetra-*n*-butylammonium iodide, DABCO = 1,4-diazabicyclo[2.2.2]octane, MS = molecular sieves.

## 2.3 General procedure for the preparation of imidosulfites

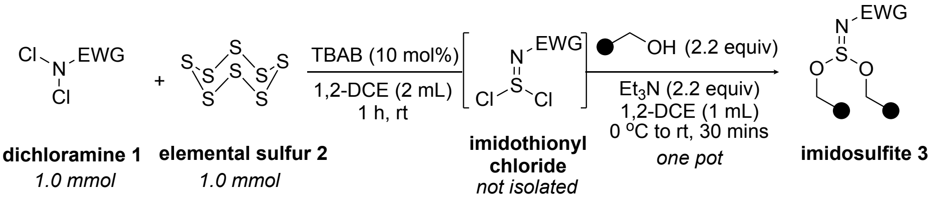


A 20 mL reaction tube was wrapped with aluminium foil and charged with dichloramine (1.0 mmol), elemental sulfur (sublimed grade, 32 mg, 1.0 mmol), and dry 1,2-DCE (2 mL). This suspension was bubbled with $N_2$ for 5 min, and then, TBAB (32 mg, 0.1 mmol) was added, and the reaction was stirred under $N_2$ at room temperature for 1 h. The tube was located in a 0 °C ice bath, and a pre-made solution of the alcohol (2.2 mmol), $Et_3N$ (306 µL, 2.2 mmol) in 1,2-DCE (1 mL) was added to the reaction mixture over the course of 10 min. The mixture was stirred under $N_2$ and allowed to warm to room temperature for 30 mins. DCM (7 mL) and water (7 mL) was added to dilute the mixture. The organic layer was separated, dried over $Mg_2SO_4$, and evaporated under reduced pressure. The product was purified by flash column chromatography to provide **3**.

<u>NOTE</u>: The synthesis worked consistently with batches of elemental sulfur that explicitly mentioned '*sublimed*'. We had trouble to obtain the same results with other grades of sulfur.

## 2.4 General procedure for the preparation of sulfinimidates and sulfinamidines

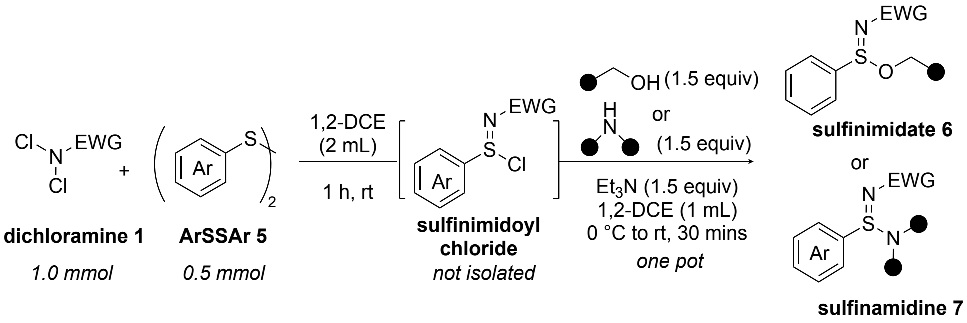


To a 20 mL reaction tube was added dichloramine **1** (1.0 mmol), diaryl disulfide **5** (0.5 mmol), and 1,2-DCE (2 mL). Then, the mixture was stirred at room temperature for 1 h. The tube was then located in a 0 °C ice bath, and a pre-made solution of the alcohol or the secondary amine (1.5 mmol), $Et_3N$ (209 µL, 1.5 mmol) in 1,2-DCE (1 mL) was added to the reaction mixture over the course of 10 min. The mixture was allowed to warm to room temperature for 30 mins. DCM (7 mL) and water (7 mL) was added to dilute the mixture. The organic layer was separated, dried over $Mg_2SO_4$ and evaporated under reduced pressure. The product was purified by flash column chromatography to provide sulfinimidate **6** or sulfinamidine **7**.

## 2.5 General procedure for the preparation of S(VI) with $NaIO_4/RuCl_3$

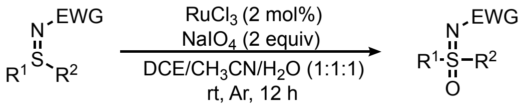


The procedure was based on a literature protocol:[S2] A mixture of S(IV) (**3**, **8**, **6a**, or **7a**) (0.2 mmol), $RuCl_3$ (0.8 mg, 2 mol%) and $NaIO_4$ (85.5 mg, 0.4 mmol), deionized water (0.5 mL), acetonitrile (0.5 mL), and DCE (0.5 mL) was placed in a 10 mL Schlenk tube, and the solution was bubbled with $N_2$ for 5 min. Then, the mixture was stirred at room temperature for 12 h. Upon completion, DCM (10 mL) and water (10 mL) was added to dilute the mixture, and the organic phase was separated, dried over $Mg_2SO_4$ and evaporated under reduced pressure. The product was purified by flash column chromatography to obtain the product **4**, **9**, **11**, or **12**.

## 2.6 Procedure for the preparation of *N*-Ts sulfilimines with Grignard reagents

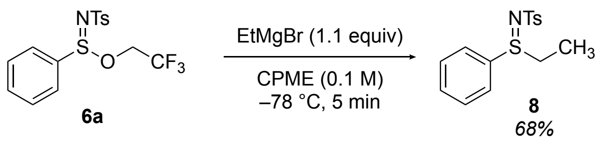


The procedure was based on a literature protocol:[S3] In an oven-dried 8 mL reaction vial was placed trifluoroethyl sulfinimidate **6a** (94 mg, 0.250 mmol), which was then brought under $N_2$ atmosphere and dissolved in dry CPME (2.5 mL). Then, the tube was cooled in a –78 °C dry ice/acetone bath and stirred, and under $N_2$ atm was added EtMgBr (3.0 M in $Et_2O$, 92 μL, 0.275 mmol, 1.1 equiv) in one go. After 5 min at –78 °C, the mixture was checked via TLC (hex/THF 5:5) to show the disappearance of the starting material and the appearance of the product spot at 0.2. The reaction mixture was quenched with few drops of MeOH and partitioned with saturated $NH_4Cl$ solution (2 mL). The organic layer was separated, and the aqueous layer was extracted two times with THF (2 mL), all organic layers were combined, dried over $MgSO_4$, filtered and concentrated. Crude mixture was then subjected to automated silica gel column chromatography (hex/THF 7:3→3:7) to furnish product **8** as a yellow solid (57 mg, 68% yield).

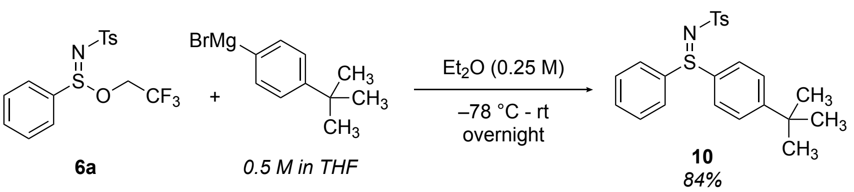


An oven-dried 10 mL reaction tube was charged with trifluoroethyl sulfinimidate **6a** (94 mg, 0.250 mmol), which was brought under $N_2$ atmosphere and dissolved in dry $Et_2O$ (1 mL). Then, the tube was cooled to –78 °C in a dry ice/acetone bath and

stirred, and under $N_2$ atm was added 4-*tert*-butylphenylmagnesium bromide (0.5 M in THF, 550 μL, 1.1 equiv) dropwise over the course of 1 min. The tube was then transferred to a 0 °C ice bath and stirred while allowing to warm to room temp overnight. Next day, the reaction mixture was partitioned between satd. aq. $NH_4Cl$ and MTBE, the organic layer was separated, and the aqueous layer was extracted two more times with MTBE. All organic layers were combined, dried over $MgSO_4$, filtered and concentrated. The crude mixture was then subjected to automated silica gel column chromatography (hex/EtOAc 7:3) to furnish product **10** as a colorless solid (86 mg, 84% yield).

## 2.7 Procedure for the preparation of 7a using ultrasound

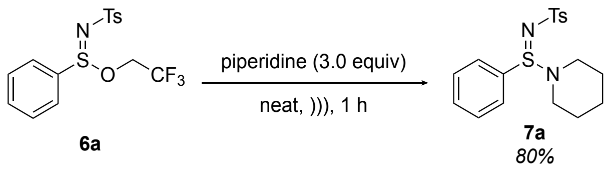


The procedure was based on a literature protocol:[S4] A 10 mL reaction tube was charged with trifluoroethyl sulfinimidate **6a** (94 mg, 0.250 mmol), and to this was added piperidine (74 μL, 0.75 mmol, 3.0 equiv). The tube was closed and placed in a 40 kHz ultrasound bath for 1 h. TLC analysis (in hex/DCM/THF 5:4:1, necessary to distinguish starting material from product) showed complete conversion of starting material to a single spot. The mixture was loaded directly onto a column for separation using automated silica gel column chromatography (hex/EtOAc 8:2) to furnish product **7a** as a colorless solid (72 mg, 80% yield). The NMR spectra were in accordance with the characterization reported above (see sulfinamidine scope).

## 2.8 Procedure for the preparation of $NH_2$-sulfinamidine 13

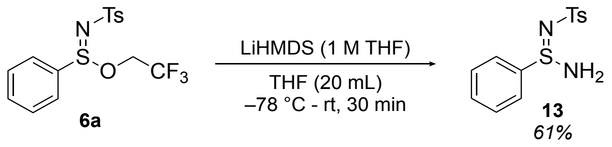


The procedure was based on a literature protocol:[S5] In an 100 mL round bottom flask was brought trifluoroethyl sulfinimidate **6a** (1.89 g, 5 mmol) and dry THF (20 mL). The flask was then cooled in a –78 °C dry ice/acetone bath and LiHMDS (1 M in THF, 7.5 mL, 7.5 mmol, 1.5 equiv) was added over the course of 10 min. The flask was then brought out of the bath and stirred while allowing to warm to room temperature. Checking by TLC after 30 min confirmed that the starting material was fully consumed. The reaction mixture was quenched with satd. aq. $NH_4Cl$ (30 mL) and reacted for another 30 mins, the organic layer was separated, and the aqueous layer was extracted with ethyl acetate (2 x 20 mL). The combined organic layers were dried over $MgSO_4$, filtered and concentrated. The product was recrystallized in *n*-

pentane/EtOAc (1/1) to give a white solid (0.89 g, 61% yield).

## 2.9 Procedure for the preparation of sulfondiimidamide 14 with $PhI(OAc)_2$

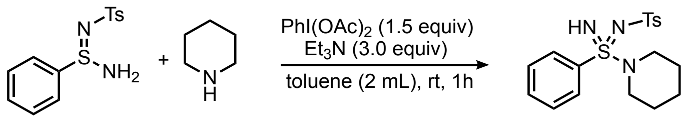


**13** (0.2 mmol) 2.0 equiv **14** *84%*

The procedure was based on a literature protocol:[S6] A mixture of benzenesulfinimidamide **13** (58.8 mg, 0.2 mmol), piperidine (40 μL, 0.4 mmol), $PhI(OAc)_2$ (96.6 mg, 0.3 mmol), $Et_3N$ (83.6 μL, 0.6 mmol), and toluene (2 mL) in a 10 mL sealed vial was stirred at room temperature for 1 h. Upon completion, the mixture was concentrated, and the product was purified by flash column chromatography (*n*-pentane/EtOAc=4:1) to provide product **14** as a colorless oil (63.0 mg, 84%).

## 2.10 Procedure for the preparation of imidosulfamite 15

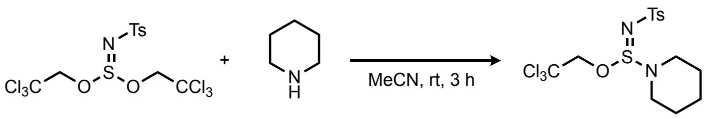


**3b** (0.1 mmol) 2.0 equiv **15** *67%*

A mixture of imidosulfite **3b** (49.5 mg, 0.1 mmol), piperidine (20 μL, 0.2 mmol), and MeCN (1 mL) in a 10 mL sealed vial was stirred at room temperature for 3 h. Upon completion, the mixture was concentrated, and the product was purified by flash column chromatography (*n*-pentane/EtOAc=7:1) to provide product **15** as a colorless oil (29.0 mg, 67%).

## 2.11 Note on the formation of side products during the oxidation of imidosulfites

For dialkyl imidosulfites **3c**, **3d**, **3i**, and **3j**, the formation of the expected imidosulfate **4** did not occur. Instead, the NMR analysis suggested an alternative product that we presume being the *N*-alkylation product, i.e. sulfamate **4'**.

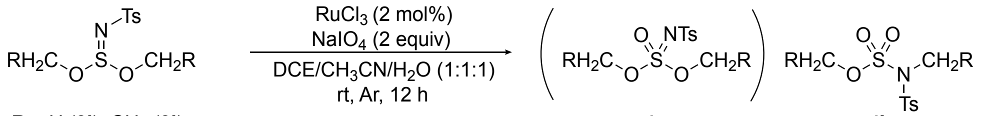


R = H (**3i**), $CH_3$ (**3j**), $CH_2Cl$ (**3c**), $CH_2Br$ (**3d**)

**4** *expected, not observed*

**4'** *sulfamate (presumed)*

The characterization reported below (in Section 3) is for these sulfamate products (**4c'**, **4d'**, **4i'**, and **4j'**) and appears in good accordance with the expected spectral data. Alternative ways to synthesize the side product, to confirm its identity, have failed.

## 2.12 NMR chemical shift predictions

### 2.12.1 Structural assignment—Procedure utilized in the DFT NMR calculations

To verify that the obtained experimental NMR spectrum corresponded to the proposed structure of sulfamate **4i'** and not to the imidosulfate **4i**, the NMR spectra of the two isomers were calculated. The following procedure was followed in both calculations. A default conformer search run was conducted within the CREST program (version 2.11.2, as a utility/driver program for the xtb program of version 6.4.0)[S7] utilizing the GFN2-xTB method[S8,S9] in combination with the ALPB solvation model,[S10] accounting for an implicit chloroform solvation. All conformers obtained by CREST within the default 6 kcal/mol energy window were subsequently subjected to energetic sorting. The energetic sorting comprised in the first step (Part 1.1) single point calculations at the r2SCAN-3c/def2-mTZVP[S11] level of theory (within ORCA 5.0.3)[S12] for all conformers within the 6 kcal/mol Crest window, whereby the chloroform solvent was taken into account via the SMD model[S13] (within ORCA 5.0.3)[S14]. The resolution of identity approximation for the Coulomb integrals[S15] was employed. For the SCF convergence thresholds and the integrations grid, the ORCA 5.0.3 defaults were utilized (NormalSCF keyword for the single-point energies and defgrid2 keyword for the integration grid). The single point energy from the DFT calculation and the free energy of solvation were added for each conformer to obtain a free energy without thermostatistical contributions $g_{\mathrm{Part1}}$[S14]. All conformers with 'free energies' $g_{\mathrm{Part1}}$ within 3.50 kcal/mol of the lowest conformer were considered for the next step (Part 1.2)[S14]. In part 1.2, single point hessian calculations[S16] (within xtb 6.4.0) were performed with the GFN2-xTB method for all eligible conformers, whereby the chloroform solvent was taken into account with the ALPB(GFN2-xTB) model (within xtb 6.4.0)[S14]. The thermostatistical free energy contributions $g_{1,\mathrm{mRRHO}}$ obtained from the single point hessian calculations were added to $g_{\mathrm{Part1}}$ to obtain a true free energy $G_{\mathrm{Part1}}$[S14]. All conformers with free energies $G_{\mathrm{Part1}}$ within 3.50 kcal/mol of the lowest conformer were considered for the next step (Part 2)[S14]. In part 2, the geometries of all eligible conformers were optimized at the r2SCAN-3c/def2-mTZVPP level of theory (within ORCA 5.0.3), whereby the chloroform solvent was treated with the SMD model (within ORCA 5.0.3) model[S14]. The resolution of identity approximation was employed for the Coulomb integrals and the defgrid2 setting was utilized for the integration grid. Furthermore, the SCF convergence thresholds were increased to the TightSCF setting in the geometry optimizations. Subsequently, single point hessian calculations (within xtb 6.4.0) were performed with the GFN2-xTB method for all optimized geometries, whereby the chloroform solvent was taken into account with the ALPB(GFN2-xTB) solvation model (within xtb 6.4.0) to obtain thermostatistical free energy contributions. The thermostatistical free energy contributions $g_{2,\mathrm{mRRHO}}$

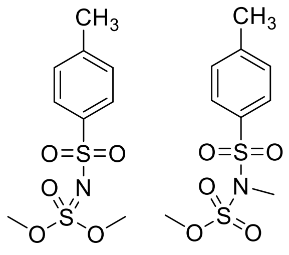

**4i** **4i'**

obtained from the single point hessian calculations were added to $g_{\mathrm{Part2}}$ to obtain a true free energy $G_{\mathrm{Part2}}$[S14]. The optimized geometries and the energies of the optimized structures were utilized to identify conformers and rotamers via the CREGEN sorting routine in the CREST program, which identifies conformers and rotamers on the basis of energies, cartesian root mean square displacements and rotational constants. If several rotamers existed for a given conformer, the conformer structure with the lowest free energy was chosen. The lowest free energy conformer was considered for the NMR part of the calculation. In the NMR part, the eligible conformer from part 2 was re-optimized at the B3LYP/6-31+G(d,p)[S17,S18] level of theory (within Gaussian 09, Revision E.01),[S19] whereby the chloroform solvent was treated with the SMD solvation model (within Gaussian 09, Revision E.01). Default integration grids were employed in the geometry optimization. In the sulfamate case, an accurate hessian was calculated at the beginning of the geometry optimization to reach proper convergence of the geometry. The obtainment of stationary points was confirmed by frequency calculations in both cases (zero imaginary frequencies). The optimized geometries were subsequently employed in GIAO-NMR[S20] calculations at the mPW1PW91/6-311+G(2d,p)[S18g–S18i,S21,S22] level of theory (within Gaussian 09, Revision E.01), whereby the SMD solvation model (within Gaussian 09, Revision E.01) was employed to account for the chloroform solvent. Calculated isotropic shielding constants were converted to chemical shifts by employing the method appropriate scaling factors ($^{13}$C: slope = –1.0512, intercept = 186.2627; $^{1}$H: slope = -1.0843, intercept = 31.8389) from Tantillo and co-workers.[S23]

### 2.12.2 Structural assignment—Comparison of the $^{13}$C chemical shifts

For the structural assignment, the carbon chemical shifts were found to be quite decisive and are, hence, shown for the two isomers in Tables S2 and S3 together with the experimentally determined values.

When comparing the experimentally determined and the computed values, it becomes evident that the $^{13}$C chemical shift of 58.8 ppm is missing for the imidosulfate isomer **4i**, whereas in the sulfamate structure **4i’** the shift can be assigned to the nitrogen bound methyl group. The calculated and experimentally determined shifts are in very good agreement for the sulfamate structure.

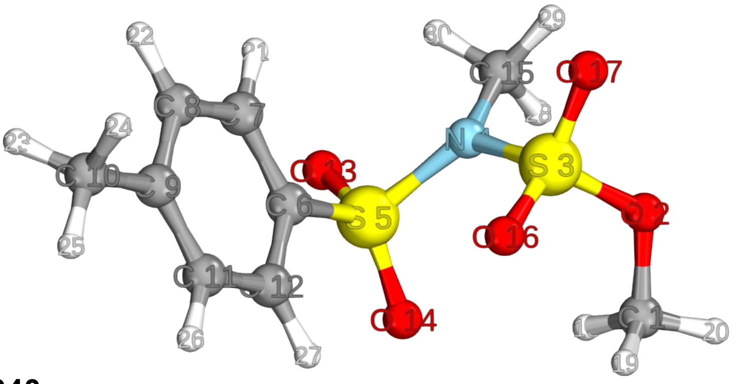


**RMSD = 1.9640 ppm**

**Figure S1.** Geometry of the lowest free energy conformer of the sulfamate isomer **4i’** optimized at the B3LYP/6-31+G(d,p) level of theory in SMD chloroform solvation.

**Table S2.** Calculated and experimentally determined $^{13}C$ chemical shifts of the sulfamate isomer **4i'**.

| Nucleus | Experimental $^{13}C$ chemical shifts sulfamate [ppm] | Calcd. $^{13}C$ chemical shifts sulfamate [ppm] | Deviation [ppm] |
|---|---|---|---|
| C10 | 21.7 | 21.1 | 0.6 |
| C15 | 36.0 | 33.1 | 2.9 |
| C1 | 58.8 | 58.0 | 0.8 |
| C7, C12 | 128.3 | 127.6 | 0.7 |
| C8, C11 | 129.8 | 128.4 | 1.4 |
| C6 | 134.8 | 138.4 | –3.6 |
| C9 | 145.5 | 147.0 | –1.5 |

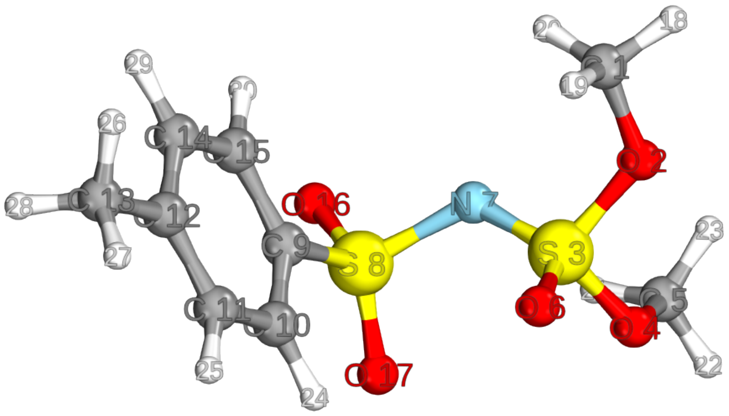

**Figure S2.** Geometry of the lowest free energy conformer of the imidosulfate isomer **4i** optimized at the B3LYP/6-31+G(d,p) level of theory in SMD chloroform solvation.

**Table S3.** Calculated $^{13}C$ chemical shifts of the imidosulfate isomer **4i**.

| Nucleus | Calcd. $^{13}C$ chemical shift imidosulfate [ppm] |
|---|---|
| C13 | 21.0 |
| C1, C5 | 59.1 |
| C10, C15 | 125.7 |
| C11, C14 | 127.4 |
| C9 | 140.1 |
| C12 | 145.6 |

### 2.12.3 Structural assignment—Comparison of the $^{1}H$ chemical shifts

The calculated and experimentally determined $^{1}H$ chemical shifts are also in very good agreement for the sulfamate structure.

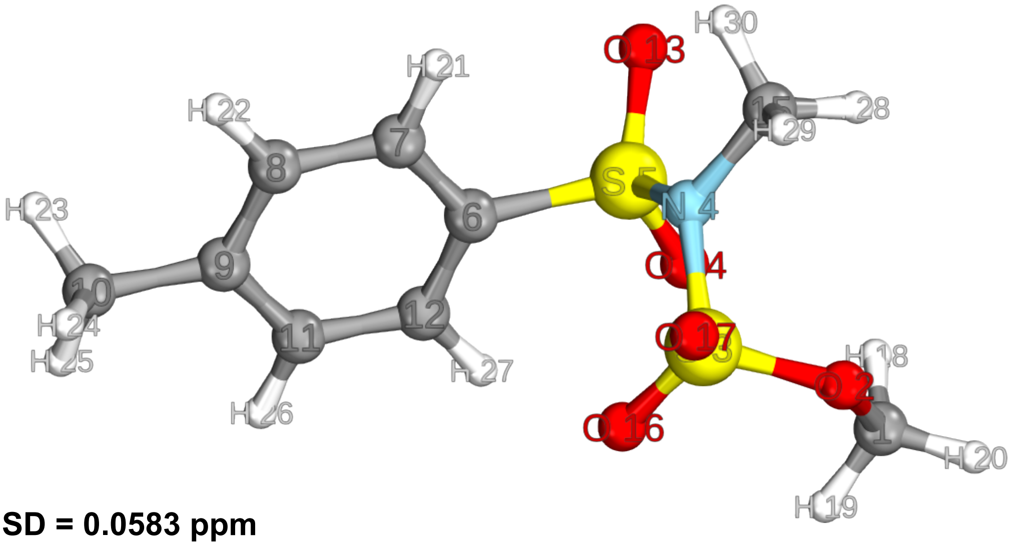


**RMSD = 0.0583 ppm**

**Figure S3.** Geometry of the lowest free energy conformer of the sulfamate isomer **4i'** optimized at the B3LYP/6-31+G(d,p) level of theory in SMD chloroform solvation.

**Table S4.** Calculated and experimentally determined $^{1}H$ chemical shifts of the sulfamate isomer **4i'**.

| Nucleus | Experimental $^{1}H$ chemical shifts sulfamate [ppm] | Calcd. $^{1}H$ chemical shifts sulfamate [ppm] |
|---|---|---|
| H23, H24, H25 | 2.46 | 2.49 |
| H28, H29, H30 | 3.32 | 3.30 |
| H18, H19, H20 | 4.03 | 4.03 |
| H22, H26 | 7.36 | 7.36 |
| H21, H27 | 7.86 | 7.73 |

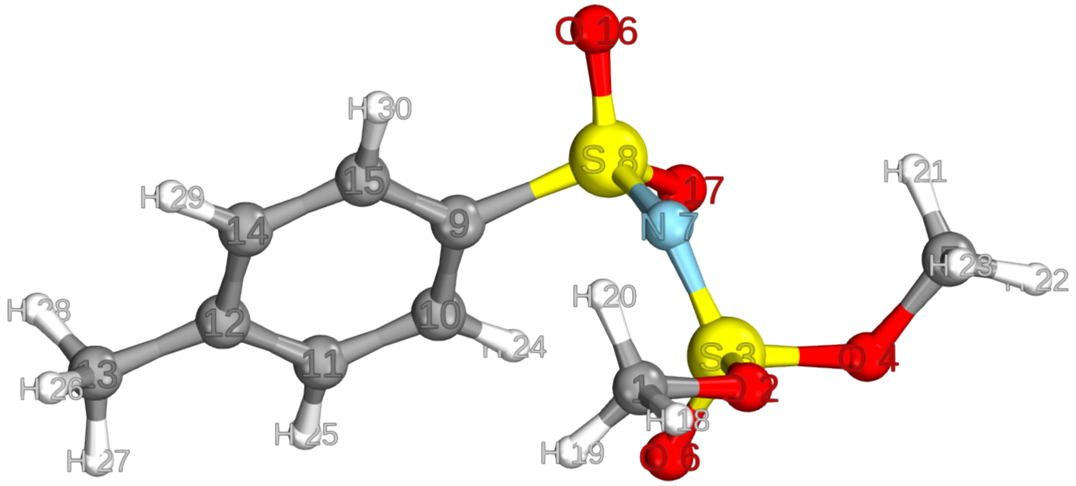


**Figure S4.** Geometry of the lowest free energy conformer of the imidosulfate isomer **4i** optimized at the B3LYP/6-31+G(d,p) level of theory in SMD chloroform solvation.

**Table S5.** Calculated $^{1}H$ chemical shifts of the imidosulfate isomer **4i**.

| **Nucleus** | **Calcd. $^{1}$H chemical shifts imidosulfate [ppm]** |
|---|---|
| H26, H27, H28 | 2.50 |
| H18, H19, H20, H21, H22, H23 | 3.89 |
| H25, H29 | 7.36 |
| H24, H30 | 7.77 |

## 2.13 The summary of failed examples

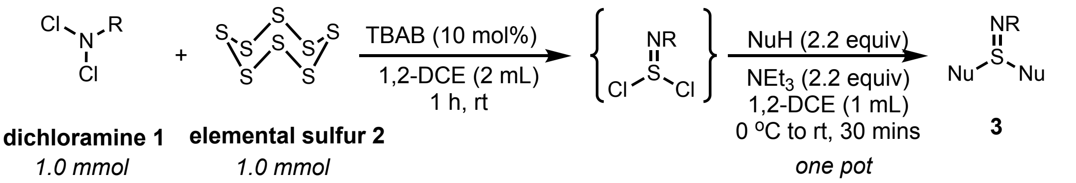


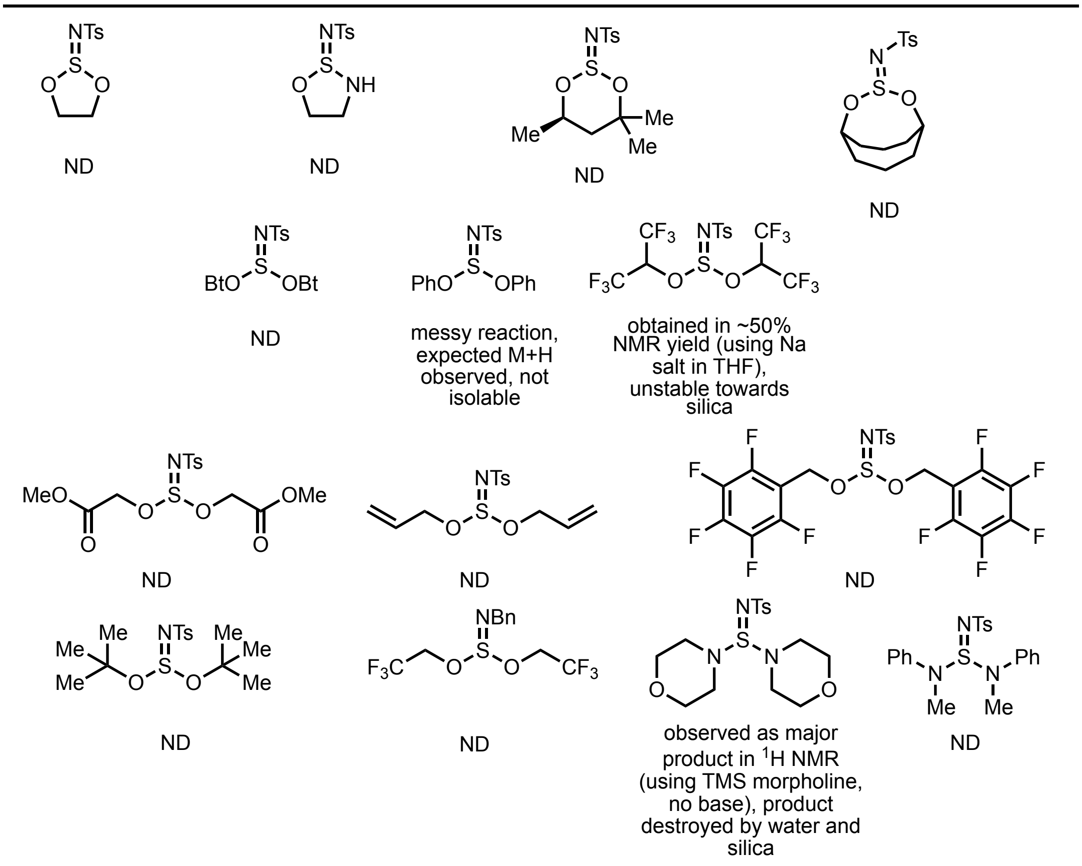


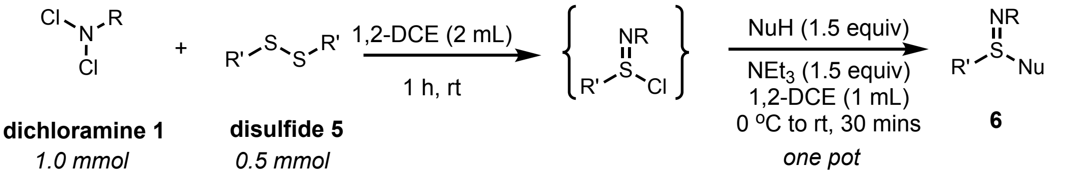


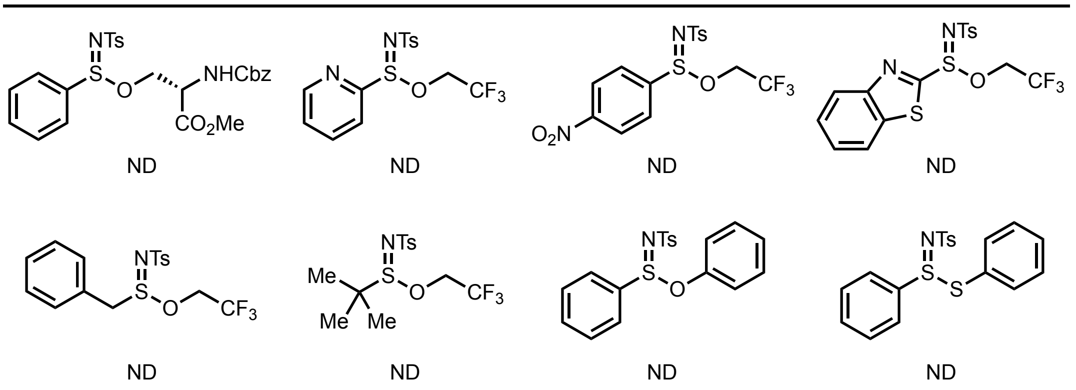

## 3. Characterization Data

### [(4-Bromophenyl) sulfonyl] chlorimidous chloride (1d)

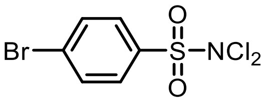


Following the general procedure afforded the product as a white solid (2.2 g, 73% yield). **m.p.**: 111.0-112.3 °C. **$^1$H NMR** (600 MHz, $CDCl_3$) δ 7.95 (d, *J* = 8.4 Hz, 1H), 7.83 (d, *J* = 8.5 Hz, 1H). **$^{13}$C{$^1$H} NMR** (151 MHz, $CDCl_3$) δ 132.7, 132.7, 132.0, 127.8. **IR** (ATR): ν = 3091, 1735, 1567, 1374, 1170, 1062, 770, 773, 700. **MS** (EI, 70 eV): *m/z* (%) = 303.3 (2), 221.0 (72), 219.0 (68), 203.0 (8), 157.0 (85), 155.0 (100), 76.2 (78), 74.2 (28), 50.2 (61). **HRMS** (EI) *m/z:* [M-2Cl+2H] Calcd for $C_6H_6O_2NSBr$ 234.9303; Found, 234.9301.

### {[4-(Trifluoromethyl) phenyl] sulfonyl} chlorimidous chloride (1e)

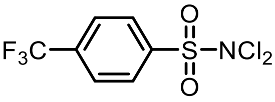


Following the general procedure afforded the product as a white solid (2.0 g, 68% yield). **m.p.**: 83.2-86.6 °C. **$^1$H NMR** (600 MHz, $CDCl_3$) δ 8.25 (d, *J* = 8.1 Hz, 1H), 7.96 (d, *J* = 8.3 Hz, 1H). **$^{13}$C{$^1$H} NMR** (151 MHz, $CDCl_3$) δ 137.4 (q, *J* = 33.4 Hz), 132.6, 132.1, 126.4 (q, *J* = 3.8 Hz), 122.8 (q, *J* = 273.3 Hz). **$^{19}$F NMR** (565 MHz, $CDCl_3$) δ -63.43. **IR** (ATR): ν = 3109, 1743, 1405, 1379, 1314, 1169, 1104, 1057, 790, 707. **MS** (EI, 70 eV): *m/z* (%) = 209.2 (19), 145.1 (100), 95.2 (19), 75.1 (19), 63.2 (4), 50.2 (15). **HRMS** (EI) *m/z:* [M-2Cl+2H] Calcd for $C_7H_6F_3O_2NS$ 225.0071; Found, 225.0069.

### (4-Chlorobenzoyl) chlorimidous chloride (1h)

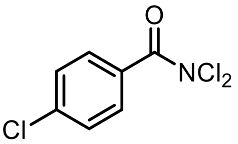


Following the general procedure afforded the product as a yellow solid (1.8 g, 81% yield). **m.p.**: 53.7-56.2 °C. **$^1$H NMR** (600 MHz, $CDCl_3$) δ 7.79 – 7.71 (m, 2H), 7.49 – 7.43 (m, 2H). **$^{13}$C{$^1$H} NMR** (151 MHz, $CDCl_3$) δ 173.3, 139.9, 131.3, 129.0, 126.9. **IR** (ATR): ν = 3158, 3096, 2090, 1703, 1588, 1398, 1221, 1088, 1005, 838, 743. **MS** (EI, 70 eV): *m/z* (%) = 141.1 (34), 139.1 (100), 113.1 (15), 111.1 (44), 75.1 (47), 63.1 (11), 50.2 (34). **HRMS** (EI) *m/z:* [M-2Cl] Calcd for $C_7H_4ONCl$ 152.9981; Found, 152.9976.

**(2,4-Dichlorobenzoyl) chlorimidous chloride (1i)**

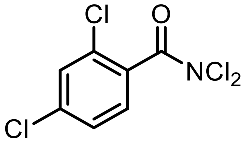

Following the general procedure afforded the product as a white solid (1.9 g, 74% yield). **m.p.**: 59.2-61.2 °C. **$^1$H NMR** (600 MHz, $CDCl_3$) δ 7.51 – 7.49 (m, 1H), 7.37 – 7.34 (m, 2H). **$^{13}$C{$^1$H} NMR** (151 MHz, $CDCl_3$) δ 169.6, 138.0, 132.9, 130.3, 129.9, 129.1, 127.4. **IR** (ATR): ν = 3078, 1697, 1582, 1375, 1238, 1100, 1046, 830,761. **MS** (EI, 70 eV): *m/z* (%) = 177.1 (10), 175.1 (100), 147.1 (14), 145.1 (27), 109.1 (25), 84.1 (13), 74.1 (30), 49.2 (15). **HRMS** (EI) *m/z:* [M-2Cl] Calcd for $C_7H_3ONCl_2$ 186.9592; Found, 186.9588.

**Bis(2,2,2-trifluoroethyl) tosylsulfurimidite (3a)**

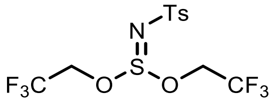

Following the general procedure afforded the product as a white solid (208.1 mg, 72% yield) and (2.41g, 60% yield, 10 mmol scale). **m.p.**: 73.4-76.5 °C. **$^1$H NMR** (600 MHz, $CDCl_3$) δ 7.79 (d, *J* = 8.1 Hz, 2H), 7.32 (d, *J* = 8.1 Hz, 2H), 4.42 (qq, *J* = 12.4, 7.8 Hz, 4H), 2.43 (s, 3H). **$^{13}$C{$^1$H} NMR** (151 MHz, $CDCl_3$) δ 144.1, 138.8, 129.8, 126.4, 121.9 (q, *J* = 277.5 Hz), 61.3 (q, *J* = 38.6 Hz), 21.5. **$^{19}$F NMR** (565 MHz, $CDCl_3$) δ –73.40 (t, *J* = 7.6 Hz). **IR** (ATR): ν = 3356, 3259, 2159, 1454, 1405, 1269, 1149, 1078, 981, 773, 669. **MS** (EI, 70 eV): *m/z* (%) = 400.1 (22), 398.8 (100), 302.0 (6), 299.9 (71), 297.7 (15), 291.8 (6), 155.0 (88), 91.1 (42). **HRMS** (ESI) *m/z:* $[M+Na]^+$ Calcd for $C_{11}H_{11}O_4NF_6NaS_2^+$ 421.9926; Found, 421.9913.

**Bis(2,2,2-trichloroethyl) tosylsulfurimidite (3b)**

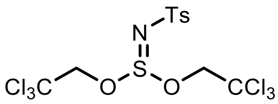

Following the general procedure afforded the product as a white solid (303.4 mg, 61% yield). **m.p.**: 73.2-76.1 °C. **$^1$H NMR** (600 MHz, $CDCl_3$) δ 7.83 (d, *J* = 8.1 Hz, 2H), 7.29 (d, *J* = 8.0 Hz, 2H), 4.74 (d, *J* = 11.7 Hz, 2H), 4.59 (d, *J* = 11.7 Hz, 2H), 2.41 (s, 3H). **$^{13}$C{$^1$H} NMR** (151 MHz, $CDCl_3$) δ 143.8, 139.1, 129.6, 126.5, 93.5, 75.0, 21.5. **IR** (ATR): ν = 2923, 1445, 1304, 1148, 1091, 960, 824, 723. **MS** (EI, 70 eV): *m/z* (%) = 349.7 (6), 268.8 (27), 266.9 (34), 218.4 (33), 217.8 (64), 155.0 (100), 91.1 (54), 65.9 (13). **HRMS** (ESI) *m/z:* $[M+Na]^+$ Calcd for $C_{11}H_{11}O_4NCl_6NaS_2^+$ 517.8153; Found, 517.8145.

**Bis(2-chloroethyl) tosylsulfurimidite (3c)**

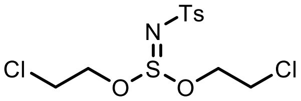

Following the general procedure afforded the product as a colorless oil (234.2 mg, 65% yield). **$^1$H NMR** (600 MHz, $CDCl_3$) δ 7.78 (d, *J* = 8.1 Hz, 2H), 7.29 (d, *J* = 8.1 Hz, 2H), 4.42 – 4.36 (m, 2H), 4.34 – 4.28 (m, 2H), 3.74 – 3.59 (m, 4H), 2.40 (s, 3H). **$^{13}C\{^1H\}$ NMR** (151 MHz, $CDCl_3$) δ 143.1, 139.8, 129.4, 126.0, 65.9, 41.3, 21.3. **IR** (ATR): ν = 2959, 1915, 1597, 1447, 1303, 1151, 989, 666. **MS** (EI, 70 eV): *m/z* (%) = 298.9 (5), 297.0 (12), 279.9 (23), 218.0 (11), 171.1 (43), 155.0 (100), 91.1 (58), 66.0 (15), 64.3 (13). **HRMS** (ESI) *m/z:* $[M+Na]^+$ Calcd for $C_{11}H_{15}O_4NCl_2NaS_2^+$ 381.9712; Found, 381.9708.

**Bis(2-bromoethyl) tosylsulfurimidite (3d)**

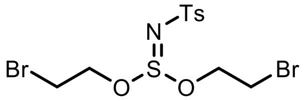


Following the general procedure afforded the product as a colorless oil (254.0 mg, 57% yield). **$^1$H NMR** (600 MHz, $CDCl_3$) δ 7.86 (d, *J* = 8.3 Hz, 2H), 7.36 (d, *J* = 8.1 Hz, 2H), 4.56 – 4.48 (m, 2H), 4.47 – 4.40 (m, 2H), 3.60 – 3.51 (m, 4H), 2.48 (s, 3H). **$^{13}C\{^1H\}$ NMR** (151 MHz, $CDCl_3$) δ 143.1, 139.8, 129.5, 126.1, 65.6, 28.0, 21.4. **IR** (ATR): ν = 2963, 1597, 1451, 1295, 1082, 921, 726. **MS** (EI, 70 eV): *m/z* (%) = 325.8 (3), 291.8 (5), 171.0 (50), 155.0 (100), 109.0 (21), 106.9 (28), 91.1 (67), 65.9 (17), 54.5 (4). **HRMS** (ESI) *m/z:* $[M+Na]^+$ Calcd for $C_{11}H_{15}O_4NBr_2NaS_2^+$ 469.8701; Found, 469.8695.

**Bis(2,2-dichloro-3,3,3-trifluoropropyl) tosylsulfurimidite (3e)**

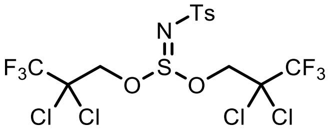


Following the general procedure afforded the product as a white solid (357.9 mg, 64% yield). **m.p.**: 73.4-76.8 °C. **$^1$H NMR** (600 MHz, $CDCl_3$) δ 7.90 – 7.71 (m, 2H), 7.31 (d, *J* = 7.9 Hz, 2H), 4.64 (d, *J* = 12.1 Hz, 2H), 4.49 (d, *J* = 12.1 Hz, 2H), 2.42 (s, 3H). **$^{13}C\{^1H\}$ NMR** (151 MHz, $CDCl_3$) δ 144.1, 139.0, 121.2 (q, *J* = 283.5 Hz), 80.3 (q, *J* = 35.9 Hz), 67.7, 21.5. **$^{19}$F NMR** (565 MHz, $CDCl_3$) δ –76.85 – –77.04 (m). **IR** (ATR): ν = 2978, 2161, 1597, 1452, 1323, 1271, 1184, 1155, 1084, 970, 889, 749, 705, 669. **MS** (EI, 70 eV): *m/z* (%) = 565.1 (30), 564.1 (20), 562.6 (28), 385.9 (6), 383.7 (48), 381.7 (55), 378.6 (19), 217.9 (19), 154.9 (100), 91.0 (48), 65.8 (11). **HRMS** (ESI) *m/z:* $[M+H]^+$ Calcd for $C_{13}H_{11}O_4NCl_4F_6NaS_2^+$ 585.8680; Found, 585.8668.

**Bis(2,2-difluoroethyl) tosylsulfurimidite (3f)**

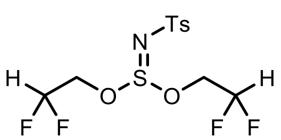


Following the general procedure afforded the product as a colorless oil (266.4 mg, 73% yield). **$^1$H NMR** (600 MHz, $CDCl_3$) δ 7.86 – 7.63 (m, 2H), 7.29 (dd, *J* = 8.4, 2.2

Hz, 2H), 5.89 (tt, *J* = 54.3, 3.6 Hz, 2H), 4.32 – 4.15 (m, 4H), 2.39 (s, 3H). **$^{13}$C{$^{1}$H} NMR** (151 MHz, $CDCl_3$) δ 143.8, 139.3, 129.7, 126.3, 111.1 (t, *J* = 243.3 Hz), 63.3 (t, *J* = 29.7 Hz), 21.5. **$^{19}$F NMR** (565 MHz, $CDCl_3$) δ –125.52 (ddt, *J* = 54.0, 26.5, 13.2 Hz). **IR** (ATR): ν = 2981, 2160, 1598, 1406, 1331, 1158, 995, 904, 810, 663. **MS** (EI, 70 eV): *m/z* (%) = 363.1 (1), 314.9 (11), 271.9 (23), 208.0 (7), 155.0 (100), 91.1 (38), 65.9 (13), 54.6 (5). **HRMS** (ESI) *m/z:* $[M+Na]^+$ Calcd for $C_{11}H_{13}O_4NF_4NaS_2^+$ 386.0114; Found, 386.0112.

**Bis(2,2,3,3-tetrafluoropropyl) tosylsulfurimidite (3g)**

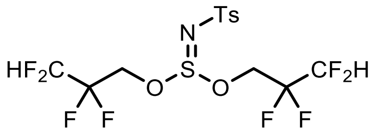


Following the general procedure afforded the product as a colorless oil (228.7 mg, 49% yield). **$^{1}$H NMR** (600 MHz, $CDCl_3$) δ 7.81 – 7.61 (m, 2H), 7.26 (d, *J* = 8.1 Hz, 2H), 5.83 (tt, *J* = 52.7, 3.7 Hz, 2H), 4.40 (h, *J* = 12.5 Hz, 4H), 2.35 (s, 3H). **$^{13}$C{$^{1}$H} NMR** (151 MHz, $CDCl_3$) δ 144.0, 138.9, 129.6, 126.1, 113.3 (tt, *J* = 251.8, 28.2 Hz), 108.8 (tt, *J* = 250.0, 35.5 Hz), 60.7 (t, *J* = 29.1 Hz), 21.1. **$^{19}$F NMR** (564 MHz, $CDCl_3$) δ –123.60 – –123.80 (m, 2F), -137.71 (dd, *J* = 52.9, 7.1 Hz, 2F). **IR** (ATR): ν = 2971, 2162, 1598, 1449, 1321, 1101, 983, 775, 666. **MS** (EI, 70 eV): *m/z* (%) = 462.9 (24), 334.0 (7), 331.9 (92), 325.5 (8), 155.0 (100), 91.1 (42), 65.9 (8), 54.6 (100). **HRMS** (ESI) *m/z:* $[M+Na]^+$ Calcd for $C_{13}H_{13}O_4NF_8NaS_2^+$ 486.0051; Found, 486.0039.

**Bis(2,2,3,3,4,4,5,5-octafluoropentyl) tosylsulfurimidite (3h)**

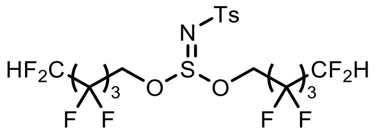


Following the general procedure afforded the product as a colorless oil (280.5 mg, 42% yield). **$^{1}$H NMR** (600 MHz, $CDCl_3$) δ 7.8 (d, *J* = 8.4 Hz, 2H), 7.3 (d, *J* = 8.1 Hz, 2H), 6.0 (tt, *J* = 51.9, 5.2 Hz, 2H), 4.5 (q, *J* = 13.0 Hz, 2H), 4.5 (q, *J* = 13.1 Hz, 2H), 2.4 (s, 3H). **$^{13}$C{$^{1}$H} NMR** (151 MHz, $CDCl_3$) δ 144.2, 138.7, 129.7, 126.4, 113.8 (tt, *J* = 259.0, 31.2 Hz), 110.9 – 109.6 (m), 107.5 (tt, *J* = 254.8, 31.5 Hz), 60.7 (t, *J* = 26.8 Hz), 21.4. **$^{19}$F NMR** (376 MHz, $CDCl_3$) δ –119.5 (t, *J* = 12.7 Hz, 2F), –125.0 (t, *J* = 8.6 Hz, 2F), –129.8 (dt, *J* = 12.7, 6.4 Hz, 2F), –137.3 (dt, *J* = 52.1, 7.7 Hz, 2F). **IR** (ATR): ν = 2971, 2324, 1599, 1403, 1326, 1161, 1127, 993, 778, 665. **MS** (EI, 70 eV): *m/z* (%) = 663.1 (1), 434.1 (6), 433.2 (13), 431.8 (100), 155.0 (88), 91.1 (29), 66.0 (5). **HRMS** (ESI) *m/z:* $[M+Na]^+$ Calcd for $C_{17}H_{13}O_4NF_{16}NaS_2^+$ 685.9923; Found, 685.9897.

**Dimethyl tosylsulfurimidite (3i)**

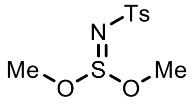

Following the general procedure afforded the product as a white solid (122.7 mg, 47% yield). **m.p.**: 59.1-61.2 °C. **$^1$H NMR** (600 MHz, $CDCl_3$) δ 7.76 (d, *J* = 8.1 Hz, 2H), 7.25 (d, *J* = 8.0 Hz, 2H), 3.70 (s, 6H), 2.37 (s, 3H). **$^{13}$C{$^1$H} NMR** (151 MHz, $CDCl_3$) δ 142.7, 140.3, 129.3, 126.0, 52.1, 21.3. **IR** (ATR): ν = 2953, 1449, 1297, 1080, 942, 730. **MS** (EI, 70 eV): *m/z* (%) = 263.0 (40), 232.0 (88), 230.3 (10), 225.4 (6), 155.0 (100), 91.1 (87), 66.0 (21), 50.5 (12). **HRMS** (ESI) *m/z:* $[M+Na]^+$ Calcd for $C_9H_{13}O_4NNaS_2^+$ 286.0178; Found, 286.0175.

**Diethyl tosylsulfurimidite (3j)**

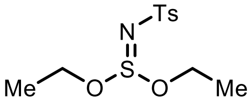

Following the general procedure afforded the product as a colorless oil (155.1 mg, 53% yield). **$^1$H NMR** (600 MHz, $CDCl_3$) δ 7.76 (d, *J* = 8.2 Hz, 2H), 7.24 (d, *J* = 8.0 Hz, 2H), 4.23 – 4.14 (m, 2H), 4.12 – 4.04 (m, 2H), 2.37 (s, 3H), 1.27 (t, *J* = 7.1 Hz, 6H). **$^{13}$C{$^1$H} NMR** (151 MHz, $CDCl_3$) δ 142.5, 140.6, 129.3, 126.0, 62.8, 21.3, 14.8. **IR** (ATR): ν = 2984, 1446, 1308, 1150, 989, 877, 664. **MS** (EI, 70 eV): *m/z* (%) = 292.1 (1), 246.0 (6), 218.0 (38), 198.0 (10), 171.0 (20), 155.0 (100), 92.1 (17), 91.1 (57), 65.9 (17), 54.5 (5). **HRMS** (ESI) *m/z:* $[M+Na]^+$ Calcd for $C_{11}H_{17}O_4NNaS_2^+$ 314.0491; Found, 314.0484.

**Diisopropyl tosylsulfurimidite (3k)**

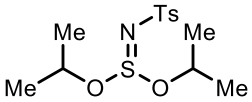

Following the general procedure afforded the product as a colorless oil (169.8 mg, 53% yield). **$^1$H NMR** (600 MHz, $CDCl_3$) δ 7.78 (d, *J* = 8.3 Hz, 2H), 7.25 (d, *J* = 8.0 Hz, 2H), 4.89 (hept, *J* = 6.2 Hz, 2H), 2.38 (s,3H), 1.32 (d, *J* = 6.3 Hz, 6H), 1.24 (d, *J* = 6.2 Hz, 6H). **$^{13}$C{$^1$H} NMR** (151 MHz, $CDCl_3$) δ 142.4, 140.9, 129.3, 126.1, 73.7, 23.2, 23.0, 21.4. **IR** (ATR): ν = 2982, 2105, 1380, 1308, 1149, 898, 666. **MS** (EI, 70 eV): *m/z* (%) = 319.8 (7), 277.9 (16), 259.9 (16), 258.4 (14), 218.1 (65), 216.9 (57), 154.9 (100), 106.0 (13), 91.0 (40), 65.8 (12), 48.3 (51). **HRMS** (ESI) *m/z:* $[M+Na]^+$ Calcd for $C_{13}H_{21}O_4NNaS_2^+$ 342.0804; Found, 342.0804.

**Dineopentyl tosylsulfurimidite (3l)**

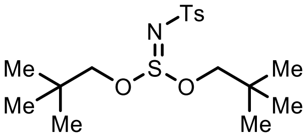

The general procedure was followed on a tenfold scale, using neopentyl alcohol afforded the product as a tan solid (2.74 g, 73% yield). **TLC** Rf 0.5 in hex/EtOAc 7:3. **m.p.**: 40-42°C. **$^1$H NMR** (400 MHz, $CDCl_3$) δ 7.84 – 7.76 (m, 2H), 7.29 – 7.22 (m, 2H), 3.78 (d, *J* = 9.3 Hz, 2H), 3.64 (d, *J* = 9.3 Hz, 2H), 2.39 (s, 3H), 0.90 (s, 18H). **$^{13}$C{$^1$H} NMR** (101 MHz, $CDCl_3$) δ 142.5, 141.0, 129.3, 126.2, 75.4, 31.8, 26.2,

21.5. **IR** (ATR): ν = 2970 (m, C-H stretch), 1598 (w, C=C stretch), 1476 (m, $CH_3$ bend), 1318 (s, O=S=O asymm stretch), 1152 (vs, O=S=O symm stretch), 1062 (s, S=N-S asymm stretch). **HRMS** (ESI) *m/z:* $[M+H]^+$ Calcd for $C_{17}H_{30}NO_4S_2^+$ 376.1616; Found, 376.1607.

**Bis(2,2,2-trifluoroethyl) (phenylsulfonyl) sulfurimidite (3m)**

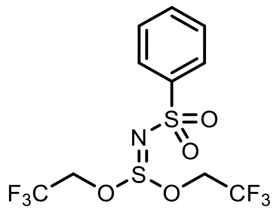


Following the general procedure afforded the product as a white solid (240.1 mg, 62% yield). **m.p.**: 55.1-57.8 °C. **$^1$H NMR** (600 MHz, $CDCl_3$) δ 7.95 – 7.89 (m, 2H), 7.66 – 7.56 (m, 1H), 7.54 –7.50 (m, 2H), 4.51 – 4.33 (m, 4H). **$^{13}C\{^1H\}$ NMR** (151 MHz, $CDCl_3$) δ 141.6, 133.1, 129.2, 126.3, 121.9 (q, *J* = 277.8 Hz), 61.3 (q, *J* = 38.3 Hz). **$^{19}$F NMR** (564 MHz, $CDCl_3$) δ –73.48 (t, *J* = 7.9 Hz). **IR** (ATR): ν = 3345, 3253, 1730, 1446, 1280, 1157, 1084, 982, 957, 750, 685. **MS** (EI, 70 eV): *m/z* (%) = 384.8 (8), 285.8 (22), 156.9 (7), 140.9 (73), 93.0 (5), 77.0 (100), 54.5 (40), 50.5 (15). **HRMS** (ESI) *m/z:* $[M+Na]^+$ Calcd for $C_{10}H_9O_4NF_6NaS_2^+$ 407.9769; Found, 407.9760.

**Bis(2,2,2-trifluoroethyl) [(4-chlorophenyl) sulfonyl] sulfurimidite (3n)**

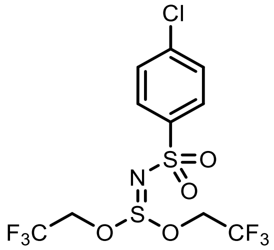


Following the general procedure afforded the product as a white solid (197.7 mg, 59% yield). **m.p.**: 70.4-72.6 °C. **$^1$H NMR** (600 MHz, $CDCl_3$) δ 7.85 (d, *J* = 8.6 Hz, 2H), 7.50 (d, *J* = 8.5 Hz, 2H), 4.52 – 4.39 (m, 4H). **$^{13}C\{^1H\}$ NMR** (151 MHz, $CDCl_3$) δ 140.1, 139.7, 129.5, 127.9, 121.8 (q, *J* = 277.4 Hz), 61.6 (q, *J* = 38.7, 38.2 Hz). **$^{19}$F NMR** (565 MHz, $CDCl_3$) δ –73.38 (t, *J* = 8.0 Hz). **IR** (ATR): ν = 2923, 2160, 1584, 1405, 1271, 1153, 1080, 984, 797, 775, 665. **MS** (EI, 70 eV): *m/z* (%) = 400.1 (22), 398.8 (100), 302.0 (6), 299.9 (71), 297.7 (15), 291.8 (6), 155.0 (88), 91.1 (42). **HRMS** (ESI) *m/z:* $[M+Na]^+$ Calcd for $C_{10}H_8O_4NClF_6NaS_2^+$ 441.9380; Found, 441.9373.

**Bis(2,2,2-trifluoroethyl) [(4-bromophenyl) sulfonyl] sulfurimidite (3o)**

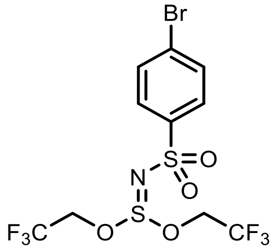

Following the general procedure afforded the product as a white solid (235.4 mg, 51% yield). **m.p.**: 80.0-89.1°C. **$^1$H NMR** (600 MHz, $CDCl_3$) δ 7.83 – 7.74 (m, 2H), 7.69 – 7.57 (m, 2H), 4.52 – 4.37 (m, 4H). **$^{13}$C{$^1$H} NMR** (151 MHz, $CDCl_3$) δ 140.6, 132.5, 128.2, 127.9, 121.8 (q, *J* = 278.4 Hz), 61.6 (q, *J* = 38.2 Hz). **$^{19}$F NMR** (565 MHz, $CDCl_3$) δ –73.37 (t, *J* = 7.8 Hz). **IR** (ATR): ν = 2924, 2161, 1575, 1455, 1406, 1269, 1150, 1079, 980, 793, 772, 736, 664. **MS** (EI, 70 eV): *m/z* (%) = 462.7 (87), 365.7 (100), 363.7 (88), 361.9 (16), 291.7 (7), 220.8 (100), 218.8 (94), 215.1 (11), 156.9 (47), 154.9 (445), 76.1 (21), 75.1 (20), 53.9 (17), 50.6 (23). **HRMS** (ESI) *m/z:* $[M+Na]^+$ Calcd for $C_{10}H_8O_4NBrF_6NaS_2^+$ 485.8875; Found, 485.8868.

**Bis(2,2,2-trifluoroethyl) {[4-(trifluoromethyl) phenyl] sulfonyl} sulfurimidite (3p)**

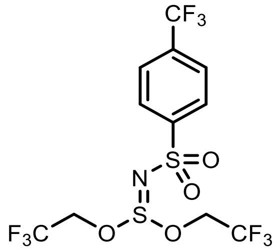

Following the general procedure afforded the product as a white solid (335.8 mg, 74% yield). **m.p.**: 62.0-63.9 °C. **$^1$H NMR** (600 MHz, $CDCl_3$) δ 8.04 (d, *J* = 8.2 Hz, 2H), 7.79 (d, *J* = 8.2 Hz, 2H), 4.81 – 3.00 (m, 4H). **$^{13}$C{$^1$H} NMR** (151 MHz, $CDCl_3$) δ 145.1, 134.8 (q, *J* = 33.0 Hz), 127.0, 126.4 (q, *J* = 3.5 Hz), 125.2 (q, *J* = 191.5 Hz), 121.8 (q, *J* = 278.4 Hz), 61.7 (q, *J* = 38.4 Hz). **$^{19}$F NMR** (376 MHz, $CDCl_3$) δ -63.29 (3F), -73.45 (t, *J* = 7.8 Hz, 6F). **IR** (ATR): ν = 2923, 2184, 1407, 1319, 1272, 1152, 1093, 983, 774, 711, 664. **MS** (EI, 70 eV): *m/z* (%) = 353.8 (18), 288.9 (12), 209.0 (56), 146.1 (11), 145.0 (100), 125.0 (7), 95.1 (8), 83.2 (8), 50.6 (38). **HRMS** (ESI) *m/z:* $[M+Na]^+$ Calcd for $C_{11}H_8O_4NF_9NaS_2^+$ 475.9643; Found, 475.9632.

**Bis(2,2,2-trifluoroethyl) (*o*-tolylsulfonyl) sulfurimidite (3q)**

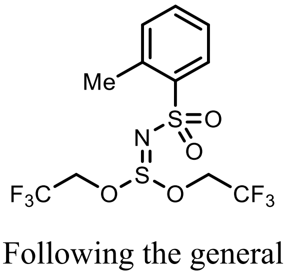

Following the general procedure afforded the product as a white solid (272.9 mg, 53%

yield). **m.p.**: 43.1-45.2 °C. **$^{1}$H NMR** (600 MHz, $CDCl_3$) δ 7.98 (dd, *J* = 8.2, 1.4 Hz, 1H), 7.46 (td, *J* = 7.5, 1.3 Hz, 1H), 7.31 (d, *J* = 7.4 Hz, 2H), 4.51-4.31 (m, 4H), 2.67 (s, 3H). **$^{13}$C{$^{1}$H} NMR** (151 MHz, $CDCl_3$) δ 139.9, 137.2, 133.2, 132.4, 127.6, 126.2, 121.9 (d, *J* = 277.4 Hz), 61.4 (q, *J* = 38.6, 38.1 Hz), 20.1. **$^{19}$F NMR** (565 MHz, $CDCl_3$) δ –73.57(t, *J* = 7.6 Hz). **IR** (ATR): ν = 3016, 2973, 2169, 1410, 1406, 1297, 1273, 1159, 1099, 990, 761, 738, 693. **MS** (EI, 70 eV): *m/z* (%) = 398.8 (14), 302.3 (13), 301.7 (27), 299.6 (100), 288.4 (18), 252.9 (11), 236.0 (15), 235.5 (18), 234.8 (14), 155.0 (31), 153.9 (41), 137.0 (34), 136.0 (51), 91.1 (66), 90.0 (41), 65.9 (19), 50.5 (9). **HRMS** (ESI) *m/z:* $[M+Na]^+$ Calcd for $C_{11}H_{11}O_4NF_6NaS_2^+$ 421.9926; Found, 421.9915.

**Bis(2,2,2-trifluoroethyl) benzoylsulfurimidite (3r)**

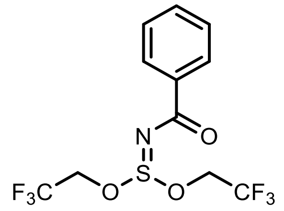


Following the general procedure afforded the product as a colorless oil (199.7 mg, 57% yield). **$^{1}$H NMR** (600 MHz, $CDCl_3$) δ 8.25 – 8.02 (m, 2H), 7.57 (td, *J* = 7.3, 1.4 Hz, 1H), 7.45 (t, *J* = 7.8 Hz, 2H), 4.74 – 4.63 (m, 2H), 4.62 – 4.50 (m, 2H). **$^{13}$C{$^{1}$H} NMR** (151 MHz, $CDCl_3$) δ 174.8, 133.8, 133.1, 129.8, 128.3, 122.3 (q, *J* = 277.4 Hz), 61.8 (q, *J* = 38.5 Hz). **$^{19}$F NMR** (565 MHz, $CDCl_3$) δ –73.53 (t, *J* = 8.0 Hz). **IR** (ATR): ν = 2972, 1628, 1270, 1164, 1005, 959, 711. **MS** (EI, 70 eV): *m/z* (%) = 348.9 (6), 250.9 (4), 105.1 (100), 77.2 (16). **HRMS** (ESI) *m/z:* $[M+Na]^+$ Calcd for $C_{11}H_9O_3NF_6NaS^+$ 372.0100; Found, 372.0090.

**Bis(2,2,2-trifluoroethyl) (4-chlorobenzoyl) sulfurimidite (3s)**

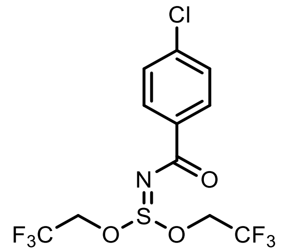


Following the general procedure afforded the product as a colorless oil (234.6 mg, 61% yield). **$^{1}$H NMR** (600 MHz, $CDCl_3$) δ 8.15 – 7.95 (m, 2H), 7.52 – 7.33 (m, 2H), 4.71 – 4.61 (m, 2H), 4.60 – 4.52 (m, 2H). **$^{13}$C{$^{1}$H} NMR** (151 MHz, $CDCl_3$) δ 173.8, 139.5, 132.3, 131.1, 128.6, 122.2 (q, *J* = 278.4 Hz), 61.9(q, *J* = 37.7 Hz). **$^{19}$F NMR** (565 MHz, $CDCl_3$) δ –73.60 (t, *J* = 7.9 Hz). **IR** (ATR): ν = 3367, 2924, 2171, 1609, 1566, 1402, 1308, 1265, 1158, 1000, 814, 727. **MS** (EI, 70 eV): *m/z* (%) = 382.8 (17), 380.4 (7), 141.0 (23), 138.9 (100), 110.9 (17), 75.1 (7), 53.9 (5). **HRMS** (ESI) *m/z:* $[M+Na]^+$ Calcd for $C_{11}H_8O_3NClF_6NaS^+$ 405.9710; Found, 405.9701.

**Bis(2,2,2-trifluoroethyl) (2,4-dichlorobenzoyl) sulfurimidite (3t)**

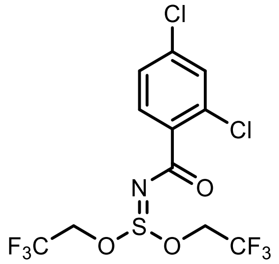


Following the general procedure afforded the product as a colorless oil (178.9 mg, 43% yield). **$^1$H NMR** (600 MHz, $CDCl_3$) δ 7.87 (d, *J* = 8.4 Hz, 1H), 7.46 (d, *J* = 2.0 Hz, 1H), 7.29 (dd, *J* = 8.5, 2.0 Hz, 1H), 4.75 – 4.64 (m, 2H), 4.62 – 4.52 (m, 2H). **$^{13}$C{$^1$H} NMR** (151 MHz, $CDCl_3$) δ 173.4, 138.1, 134.4, 132.8, 131.9, 131.0, 127.0, 122.1 (q, *J* = 277.4 Hz), 62.2 (q, *J* = 37.7 Hz). **$^{19}$F NMR** (565 MHz, $CDCl_3$) δ –73.55 (t, *J* = 7.8 Hz). **IR** (ATR): ν = 2973, 1629, 1585, 1404, 1377, 1274, 1163, 1003, 959, 765. **MS** (EI, 70 eV): *m/z* (%) = 418.3 (28), 416.8 (65), 271.9 (7), 176.9 (12), 172.9 (100), 145.0 (10), 109.0 (5), 74.1 (3). **HRMS** (ESI) *m/z:* $[M+H]^+$ Calcd for $C_{11}H_8O_3NCl_2F_6S^+$ 417.9501; Found, 417.9508.

**Bis(2,2,2-trifluoroethyl) (benzylsulfonyl) sulfurimidite (3u)**

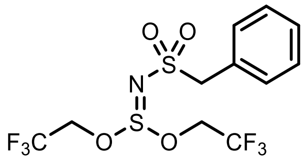


Following the general procedure afforded the product as a white solid (251.6 mg, 63% yield). **m.p.**: 41.1-44.7 °C. **$^1$H NMR** (600 MHz, $CDCl_3$) δ 7.46-7.34 (m, 5H), 4.38 (s, 3H), 4.35-4.20 (m, 4H). **$^{13}$C{$^1$H} NMR** (151 MHz, $CDCl_3$) δ 130.9, 129.1, 128.8, 121.9 (q, *J* = 277.6 Hz), 61.19 (q, *J* = 38.2 Hz), 61.15. **$^{19}$F NMR** (565 MHz, $CDCl_3$) δ –73.29 (t, *J* = 7.7 Hz). **IR** (ATR): ν = 2986, 1451, 1404, 1275, 1166, 1139, 991, 957, 790, 697. **MS** (EI, 70 eV): *m/z* (%) = 399.2 (1), 398.6 (5), 235.9 (21), 91.0 (100), 65.9 (8), 50.6 (5). **HRMS** (ESI) *m/z:* $[M+Na]^+$ Calcd for $C_{11}H_{11}O_4NF_6NaS_2^+$ 421.9926; Found, 421.9910.

***N*-(5,5-Dimethyl-1,3,2λ$^4$-dioxathian-2-ylidene)-4-methylbenzenesulfonamide (3v)**

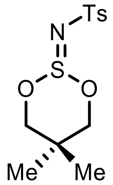


The general procedure was followed using 2,2-dimethyl-1,3-propanediol (CAS 20031-21-4, 1.1 mmol), afforded the product as a yellow solid (245.2 mg, 81% yield). **m.p.**: 120-122.2 °C. **$^1$H NMR** (600 MHz, $CDCl_3$) δ 7.81 (d, *J* = 8.3 Hz, 2H), 7.31 – 7.26 (m, 2H), 4.87 (d, *J* = 10.9 Hz, 2H), 3.58 (dt, *J* = 11.1, 1.2 Hz, 2H), 2.43 (s, 3H), 1.30 (s, 3H), 0.87 (s, 3H). **$^{13}$C{$^1$H} NMR** (151 MHz, $CDCl_3$) δ 142.6, 140.6, 129.4, 126.1, 69.3, 31.9, 22.6, 22.2, 21.5. **MS** (EI, 70 eV): *m/z* (%) = 279.0 (18), 278.0 (42),

214.0 (16), 157.0 (6), 138.0 (100), 136.0 (87), 110.0 (100), 111.0 (20), 77.1 (20), 51.2 (20). **IR** (ATR): ν = 2961, 1450, 1294, 1150, 1024, 945, 816, 735, 660. **HRMS** (ESI) *m/z:* $[M+Na]^+$ Calcd for $C_{12}H_{17}O_4NNaS_2^+$ 326.0491; Found, 326.0475.

***N*-(1,3,2 λ⁴-Dioxathian-2-ylidene)-4-methylbenzenesulfonamide (3w)**

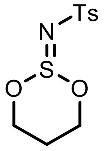


The general procedure was followed using 1,3-propanediol (CAS 504-63-2, 1.1 mmol), afforded the product as a yellow solid (102.8 mg, 37% yield). **m.p.**:65.6-67.8 °C. **$^1$H NMR** (600 MHz, $CDCl_3$) δ 7.78 (d, *J* = 8.3 Hz, 2H), 7.26 (d, *J* = 8.2 Hz, 2H), 5.19 – 5.12 (m, 2H), 4.02 – 3.98 (m, 2H), 2.63 – 2.53 (m, 1H), 2.39 (s, 3H), 1.70 – 1.63 (m, 1H). **$^{13}$C{$^1$H} NMR** (151 MHz, $CDCl_3$) δ 142.6, 140.6, 129.4, 126.1, 60.3, 25.4, 21.4. **MS** (EI, 70 eV): *m/z* (%) = 276.1 (18), 275.0 (8), 213.1 (16), 155.0 (65), 91.2 (100), 65.2 (32). **IR** (ATR): ν = 3356, 3261, 1596, 1292, 1149, 1090 1048, 989, 815, 661. **HRMS** (ESI) *m/z:* $[M+H]^+$ Calcd for $C_{10}H_{14}O_4NS_2^+$ 276.0359; Found, 276.0371.

***N*-[(4a*R*, 5a*R*, 8a*R*, 8b*S*)-7,7-Dimethyltetrahydro-4H 2λ⁴[1,3] dioxolo[4',5':4,5] furo[3,2-*d*][1,3,2]dioxathiin-2-ylidene]-4-methylbenzenesulfonamide (3x)**

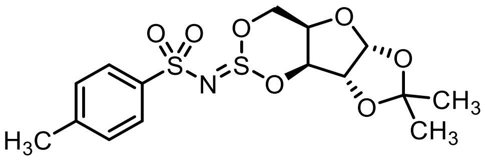


The general procedure was followed using 1,2-*O*-isopropylidene-α-D-xylofuranose (CAS 20031-21-4, 1.1 mmol), affording the product as a colorless solid (148 mg, 38% yield) and as a single diastereomer. **TLC** $R_f$ 0.6 in hex/EtOAc 5:5. **m.p.**: 148-150°C. **$^1$H NMR** (400 MHz, $CDCl_3$) δ 7.78 (d, *J* = 8.4 Hz, 2H), 7.28 (d, *J* = 8.4 Hz, 2H), 5.98 (d, *J* = 3.7 Hz, 1H), 5.16 – 5.06 (m, 2H), 4.59 (d, *J* = 3.8 Hz, 1H), 4.26 (d, *J* = 12.9 Hz, 1H), 4.16 – 4.13 (m, 1H), 2.41 (s, 3H), 1.45 (s, 3H), 1.31 (s, 3H). **$^{13}$C{$^1$H} NMR** (101 MHz, $CDCl_3$) δ 143.0, 139.9, 129.4, 125.9, 112.6, 104.4, 82.9, 71.7, 70.3, 58.8, 26.3, 25.9, 21.3. **IR** (ATR): ν = 2985, 2948 (w, C-H stretch), 1596 (w, C=C stretch), 1379 (m, $CH_3$ bend), 1310 (s, O=S=O asymm stretch), 1148 (s, C-O stretch), 1074 (s, S=N-S asymm stretch), 994 (vs, N=S stretch). **HRMS** (ESI) *m/z:* $[M+H]^+$ Calcd for $C_{15}H_{20}NO_7S_2^+$ 390.0676; Found, 390.0673.

**4-Methyl-*N*-{(1*R*,2*S*,4*R*)-2λ[4]-spiro[bicyclo[2.2.1]heptane-2,5'-[1,3,2]dioxathian]-5-en-2'-ylidene}benzenesulfonamide (3y)**

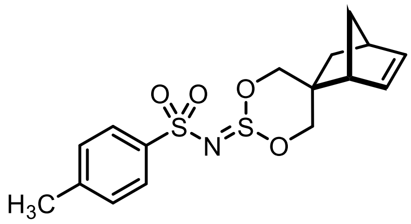

The general procedure was followed using 5-norbornene-2,2-dimethanol (CAS 6707-12-6, 1.1 mmol), affording the product as a colorless solid (154 mg, 44% yield) as a 58:42 mixture of diastereomers. **TLC** Rf 0.2 in hex/EtOAc 7:3. **m.p.**: 123-126°C. **$^1$H NMR** (400 MHz, $CDCl_3$) δ 7.78 (dd, *J* = 8.5, 2.3 Hz, 2H), 7.30 – 7.23 (m, 2H), 6.28 (dd, *J* = 5.7, 3.0 Hz, 0.5H), 6.19 (dm, *J* = 15.5, 1H), 5.93 (dd, *J* = 5.7, 3.0 Hz, 0.5H), 5.20 (d, *J* = 11.3 Hz, 0.5H), 5.14 (d, *J* = 11.0 Hz, 0.5H), 4.96 (d, *J* = 10.9 Hz, 0.5H), 4.90 (d, *J* = 11.3 Hz, 0.5H), 3.86 – 3.80 (m, 1H), 3.57 (dd, *J* = 11.3, 2.8 Hz, 0.5H), 3.44 (dd, *J* = 10.9, 2.5 Hz, 0.5H), 3.24 (s, 0.5H), 2.98 (s, 0.5H), 2.83 (s, 0.5H), 2.52 (s, 0.5H), 2.40 (s, 3H), 1.92 (dd, *J* = 12.5, 3.7 Hz, 0.5H), 1.62 (d, *J* = 10.5 Hz, 0.5H), 1.52 – 1.41 (m, 1.5H), 1.35 (d, *J* = 12.7 Hz, 0.5H), 1.29 – 1.21 (m, 0.5H), 1.18 (dd, *J* = 12.8, 3.6 Hz, 0.5H), 0.64 (dd, *J* = 12.8, 2.7 Hz, 0.5H). **$^{13}$C{$^1$H} NMR** (101 MHz, $CDCl_3$) δ 142.4, 140.4, 139.0, 138.2, 134.1, 131.3, 129.2, 125.8, 125.8, 70.3, 68.9, 67.6, 66.6, 47.3, 46.8, 46.4, 44.7, 43.4, 43.1, 42.7, 41.4, 34.8, 34.2, 21.2. **IR** (ATR): ν = 2954, 2875 (w, C-H stretch), 1596 (w, C=C stretch), 1453 (w, $CH_3$ bend), 1285 (m, O=S=O asymm stretch), 1144 (s, C-O stretch), 1030 (s, S=N-S asymm stretch), 933 (vs, C-O stretch). **HRMS** (ESI) *m/z:* $[M+H]^+$ Calcd for $C_{16}H_{20}NO_4S_2^+$ 354.0828; Found, 354.0827.

***N*-(4-Isopropyl-5,5-dimethyl-1,3,2λ[4]-dioxathian-2-ylidene)-4-methylbenzene-sulfonamide (3z)**

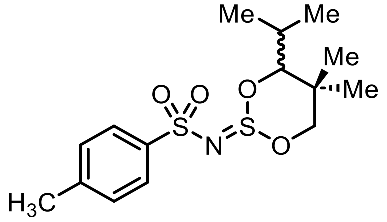

The general procedure was followed using 2,2,4-trimethylpentane-1,3-diol (CAS 144-19-4, 1.1 mmol), affording the product as a colorless solid (177 mg, 51% yield) and as a single diastereomer. **TLC** Rf 0.25 in hex/EtOAc 7:3. **m.p.**: 101.5-103.5°C. **$^1$H NMR** (400 MHz, $CDCl_3$) δ 7.78 (d, J = 8.4 Hz, 2H), 7.25 (d, J = 8.3 Hz, 2H), 4.84 – 4.75 (m, 2H), 3.41 (d, J = 11.3 Hz, 1H), 2.39 (s, 3H), 1.97 (hepd, J = 6.8, 4.7 Hz, 1H), 1.21 (s, 3H), 0.89 – 0.83 (m, 9H). **$^{13}$C{$^1$H} NMR** (101 MHz, $CDCl_3$) δ 142.5, 140.9, 129.4, 126.1, 83.8, 71.2, 36.4, 28.3, 22.9, 22.0, 21.4, 19.0, 18.0. **IR** (ATR): ν = 2952, 2913 (w, C-H stretch), 1600 (w, C=C stretch), 1464 (w, $CH_3$ bend), 1284 (s, O=S=O asymm stretch), 1143 (s, C-S stretch), 1039 (s, S=N-S asymm stretch), 947 (vs, C-O stretch). **HRMS** (ESI) *m/z:* [M+Na]+ Calcd for $C_{15}H_{23}NNaO_4S_2^+$ 368.0961; Found, 368.0963.

**Bis(2,2,2-trifluoroethyl) tosylsulfurimidate (4a)**

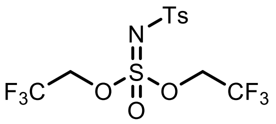

Following the general procedure afforded the product as a white solid (77.1 mg, 93% yield). **m.p.**: 81.2-82.7 °C. **$^{1}$H NMR** (600 MHz, $CDCl_3$) δ 7.84 (d, *J* = 8.4 Hz, 2H), 7.34 (d, *J* = 8.1 Hz, 2H), 4.66 (q, *J* = 7.5 Hz, 4H), 2.44 (s, 3H). **$^{13}$C{$^{1}$H} NMR** (151 MHz, $CDCl_3$) δ 144.7, 137.9, 129.7, 127.0, 120.9 (q, *J* = 277.4 Hz), 68.3 (q, *J* = 39.8 Hz), 21.6. **$^{19}$F NMR** (565 MHz, $CDCl_3$) δ –73.61 (t, *J* = 7.6 Hz). **IR** (ATR): ν = 3035, 1598, 1418, 1344, 1274, 1161, 1135, 989, 887, 750, 663. **MS** (EI, 70 eV): *m/z* (%) = 414.9 (67), 398.9 (5), 351.0 (10), 310.0 (10), 307.9 (100), 155.0 (33), 91.1 (34), 65.9 (8). **HRMS** (ESI) *m/z:* $[M+Na]^+$ Calcd for $C_{11}H_{11}O_5NF_6NaS_2^+$ 437.9875; Found, 437.9858.

**Bis(2,2,2-trichloroethyl) tosylsulfurimidate (4b)**

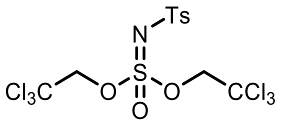

Following the general procedure afforded the product as a white solid (72.8 mg, 71% yield). **m.p.**: 82.1-84.3 °C. **$^{1}$H NMR** (600 MHz, $CDCl_3$) δ 7.89 (d, *J* = 6.2 Hz, 2H), 7.33 (d, *J* = 7.8 Hz, 2H), 4.89 (s, 4H), 2.44 (s, 3H). **$^{13}$C{$^{1}$H} NMR** (151 MHz, $CDCl_3$) δ 144.4, 138.2, 129.7, 127.1, 91.6, 80.8, 21.6. **IR** (ATR): ν = 2962, 1598, 1444, 1328, 1265, 1141, 1002, 886, 786, 712. **MS** (EI, 70 eV): *m/z* (%) = 510.0 (1), 365.7 (3), 234.0 (15), 233.1 (100), 155.0 (92), 130.9 (6), 91.1 (23), 65.9 (4). **HRMS** (ESI) *m/z:* $[M+Na]^+$ Calcd for $C_{11}H_{11}O_5NCl_6NaS_2^+$ 533.8102; Found, 533.8094.

**1-[(2-Chloroethoxysulfonyl)(2-chloroethyl)aminosulfonyl]-4-methylbenzene (4c')**

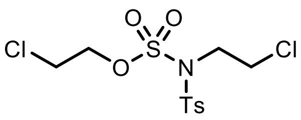

Following the general procedure afforded the above product (see note, section 2.11) as a colorless oil (23.3 mg, 31% yield). **$^{1}$H NMR** (600 MHz, $CDCl_3$) δ 7.87 (d, *J* = 8.3 Hz, 2H), 7.37 (d, *J* = 8.1 Hz, 2H), 4.64 (t, *J* = 5.7 Hz, 2H), 4.05 (t, *J* = 7.4 Hz, 2H), 3.88 – 3.67 (m, 4H), 2.46 (s, 3H). **$^{13}$C{$^{1}$H} NMR** (151 MHz, $CDCl_3$) δ 146.0, 135.2, 129.9, 128.4, 72.5, 50.6, 40.6, 40.6, 21.7. **IR** (ATR): ν = 2969, 2922, 2172, 1731, 1594, 1446, 1403, 1355, 1161, 1083, 919, 858, 775. **MS** (EI, 70 eV): *m/z* (%) = 376.7 (13), 374.9 (14), 327.4 (50), 325.9 (75), 313.9 (9), 155.0 (100), 91.1 (35), 64.3 (3). **HRMS** (ESI) *m/z:* $[M+Na]^+$ Calcd for $C_{11}H_{15}O_5NCl_2NaS_2^+$ 397.9661; Found, 397.9652.

**1-[(2-Bromoethoxysulfonyl)(2-bromoethyl)aminosulfonyl]-4-methylbenzene (4d')**

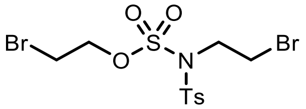


Following the general procedure afforded the above product (see note, section 2.11) as a colorless oil (32.7 mg, 35% yield). **$^{1}$H NMR** (600 MHz, $CDCl_3$) δ 7.86 (d, *J* = 8.4 Hz, 2H), 7.37 (d, *J* = 8.1 Hz, 2H), 4.67 (t, *J* = 6.2 Hz, 2H), 4.31 – 3.95 (m, 2H), 3.71 – 3.37 (m, 4H), 2.47 (s, 3H). **$^{13}$C{$^{1}$H} NMR** (151 MHz, $CDCl_3$) δ 146.0, 135.1, 130.0, 128.4, 72.1, 50.6, 27.5, 26.9, 21.7. **IR** (ATR): ν = 2962, 2922, 1730, 1594, 1444, 1402, 1355, 1160, 1070, 851, 717, 672. **MS** (EI, 70 eV): *m/z* (%) = 464.8 (7), 462.8 (4), 371.8 (84), 369.9 (81), 367.1 (17), 291.9 (9), 155.0 (100), 91.1 (48), 65.9 (9)**. HRMS** (ESI) *m/z:* $[M+Na]^+$ Calcd for $C_{11}H_{15}O_5NBr_2NaS_2^+$ 485.8651; Found, 485.8642.

**Bis(2,2-difluoroethyl) tosylsulfurimidate (4f)**

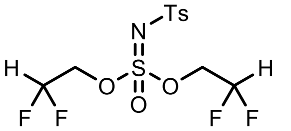


Following the general procedure afforded the product as a colorless oil (55.3 mg, 72% yield). **$^{1}$H NMR** (600 MHz, $CDCl_3$) δ 7.80 (d, *J* = 8.1 Hz, 2H), 7.30 (d, *J* = 8.1 Hz, 2H), 6.01 (tt, *J* = 53.8, 3.6 Hz, 2H), 4.48 (td, *J* = 12.9, 3.6 Hz, 4H), 2.40 (s, 3H). **$^{13}$C{$^{1}$H} NMR** (151 MHz, $CDCl_3$) δ 144.4, 138.1, 129.6, 126.7, 110.9 (t, *J* = 243.4 Hz), 70.4 (t, *J* = 30.3 Hz), 21.3. **$^{19}$F NMR** (565 MHz, $CDCl_3$) δ –126.20 (td, *J* = 12.4, 3.2 Hz), –126.29 (td, *J* = 12.7, 3.7 Hz). **IR** (ATR): ν = 2981, 1598, 1406, 1331, 1158, 1087, 996, 905, 810, 663. **MS** (EI, 70 eV): *m/z* (%) = 378.9 (33), 314.9 (9), 271.9 (21), 155.0 (100), 91.1 (43), 65.9 (16). **HRMS** (ESI) *m/z:* $[M+Na]^+$ Calcd for $C_{11}H_{13}O_5NF_4NaS_2^+$ 402.0064; Found, 402.0050.

**Bis(2,2,3,3-tetrafluoropropyl) tosylsulfurimidate (4g)**

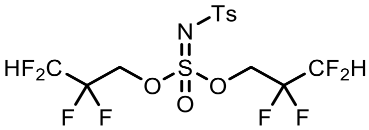


Following the general procedure afforded the product as a colorless oil (61.5 mg, 64% yield). **$^{1}$H NMR** (600 MHz, $CDCl_3$) δ 7.82 (d, *J* = 8.4 Hz, 2H), 7.32 (d, *J* = 8.1 Hz, 2H), 5.91 (tt, *J* = 52.7, 3.5 Hz, 2H), 4.79 – 4.57 (m, 4H), 2.42 (s, 3H). **$^{13}$C{$^{1}$H} NMR** (151 MHz, $CDCl_3$) δ 144.7, 138.0, 129.7, 126.9, 112.6 (tt, *J* = 252.7, 29.0 Hz), 110.7 – 106.5 (m), 68.0 (t, *J* = 30.1 Hz), 21.4. **$^{19}$F NMR** (564 MHz, $CDCl_3$) δ –123.48 – –123.71 (m, 2F), –137.28 (dd, *J* = 52.6, 25.6 Hz, 2F). **IR** (ATR): ν = 3038, 2169, 1596, 1341, 1228, 1109, 990, 947, 842, 744, 668. **MS** (EI, 70 eV): *m/z* (%) = 478.9 (20), 414.9 (26), 371.9 (100), 347.9 (9), 257.9 (24), 155.1 (40), 91.1 (40), 54.7 (18). **HRMS** (ESI) *m/z:* $[M+Na]^+$ Calcd for $C_{13}H_{13}O_5NF_8NaS_2^+$ 501.0000; Found, 501.9981.

**Bis(2,2,3,3,4,4,5,5-octafluoropentyl) tosylsulfurimidate (4h)**

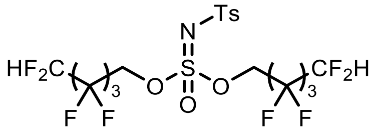

Following the general procedure afforded the product as a colorless oil (77.6 mg, 57% yield). **$^{1}$H NMR** (600 MHz, $CDCl_3$) δ 7.84 (d, *J* = 8.3 Hz, 2H), 7.33 (d, *J* = 8.0 Hz, 2H), 6.04 (tt, *J* = 51.8, 5.1 Hz, 2H), 4.75 (t, *J* = 12.6 Hz, 4H), 2.43 (s, 3H). **$^{13}$C NMR** (151 MHz, $CDCl_3$) δ 144.8, 137.9, 129.7, 127.0, 113.0 (tt, *J* = 260.3, 31.8 Hz), 110.9 – 109.6 (m), 107.5 (tt, *J* = 254.5, 31.5 Hz), 67.9 (t, *J* = 27.7 Hz), 21.4. **$^{19}$F NMR** (564 MHz, $CDCl_3$) δ –119.8 (t, *J* = 12.1 Hz, 2F), –125.0 (t, *J* = 9.0 Hz, 2F), –129.6 (td, *J* = 9.2, 4.5 Hz, 2F), –134.6 – –140.6 (m, 2F). **IR** (ATR): ν = 2983, 1599, 1406, 1342, 1164, 1128, 1011, 807, 664. **MS** (EI, 70 eV): *m/z* (%) = 678.9 (12), 574.4 (14), 571.6 (17), 357.8 (33), 218.0 (15), 155.1 (100), 145.0 (25), 126.0 (24), 91.2 (77), 66.0 (14), 54.8 (24). **HRMS** (ESI) *m/z:* $[M+Na]^+$ Calcd for $C_{17}H_{13}O_5NF_{16}NaS_2^+$ 701.9872; Found, 701.9846.

**1-[(Methoxysulfonyl)(methyl)aminosulfonyl]-4-methylbenzene (4i')**

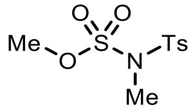

Following the general procedure afforded the above product (see note, section 2.11 and 2.12) as a white solid (12.8 mg, 23% yield). The desired imidosulfate product (**4i**) was not observed. **m.p.**: 101.0-103.6 °C. **$^{1}$H NMR** (600 MHz, $CDCl_3$) δ 7.86 (d, *J* = 8.2 Hz, 2H), 7.36 (d, *J* = 8.1 Hz, 2H), 4.03 (s, 3H), 3.32 (s, 3H), 2.46 (s, 3H). **$^{13}$C{$^{1}$H} NMR** (151 MHz, $CDCl_3$) δ 145.5, 134.8, 129.8, 128.3, 58.8, 36.0, 21.7. **IR** (ATR): ν = 3276, 2922, 2853, 1731, 1595, 1447, 1393, 1358, 1163, 993, 841, 793, 684. **MS** (EI, 70 eV): *m/z* (%) = 278.9 (7), 155.0 (47), 107.1 (11), 91.1 (100), 65.9 (23), 54.6 (6), 45.3 (9). **HRMS** (ESI) *m/z:* $[M+Na]^+$ Calcd for $C_9H_{13}O_5NNaS_2^+$ 302.0127; Found, 302.0121.

**1-[(Ethoxysulfonyl)(ethyl)aminosulfonyl]-4-methylbenzene (4j')**

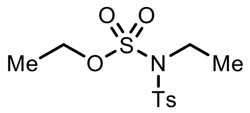

Following the general procedure afforded the above product (see note, section 2.11) as a colorless oil (10.1 mg, 16% yield). **$^{1}$H NMR** (600 MHz, $CDCl_3$) δ 7.87 (d, *J* = 8.4 Hz, 2H), 7.34 (d, *J* = 8.1 Hz, 2H), 4.46 (q, *J* = 7.1 Hz, 2H), 3.83 (q, *J* = 7.1 Hz, 2H), 2.45 (s, 3H), 1.44 (t, *J* = 7.1 Hz, 3H), 1.36 (t, *J* = 7.0 Hz, 3H). **$^{13}$C{$^{1}$H} NMR** (151 MHz, $CDCl_3$) δ 145.2, 136.1, 129.7, 128.2, 70.0, 46.1, 21.7, 15.3, 14.6. **IR** (ATR): ν = 2985, 2923, 1731, 1597, 1450, 1390, 1167, 999, 931, 876, 758, 689. **MS** (EI, 70 eV): *m/z* (%) = 307.0 (32), 292.0 (23), 215.0 (6), 155.0 (100), 91.1 (53), 46.7 (3). **HRMS** (ESI) *m/z:* $[M+Na]^+$ Calcd for $C_{11}H_{17}O_5NNaS_2^+$ 330.0440; Found, 330.0433.

**Bis(2,2,2-trifluoroethyl) (phenylsulfonyl) sulfurimidate (4m)**

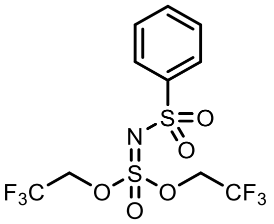


Following the general procedure afforded the product as a colorless oil (49.5 mg, 62% yield). **$^{1}$H NMR** (600 MHz, $CDCl_3$) δ 8.17 – 7.94 (m, 2H), 7.65 (t, *J* = 7.4 Hz, 1H), 7.55 (t, *J* = 7.7 Hz, 2H), 4.67 (q, *J* = 7.5 Hz, 4H). **$^{13}$C{$^{1}$H} NMR** (151 MHz, $CDCl_3$) δ 140.7, 133.6, 129.1, 127.0, 120.9 (d, *J* = 277.5 Hz), 68.4 (q, *J* = 39.8 Hz), 29.7. **$^{19}$F NMR** (565 MHz, $CDCl_3$) δ –73.57 (t, *J* = 7.2 Hz). **IR** (ATR): ν = 2918, 2850, 1449, 1414, 1341, 1280, 1166, 1003, 961, 843, 741. **MS** (EI, 70 eV): *m/z* (%) = 400.9 (26), 336.9 (12), 307.9 (100), 141.0 (35), 91.1 (11), 77.2 (47), 54.6 (23). **HRMS** (ESI) *m/z:* $[M+Na]^+$ Calcd for $C_{10}H_9O_5NF_6NaS_2^+$ 423.9719; Found, 423.9714.

**Bis(2,2,2-trifluoroethyl) [(4-chlorophenyl) sulfonyl] sulfurimidate (4n)**

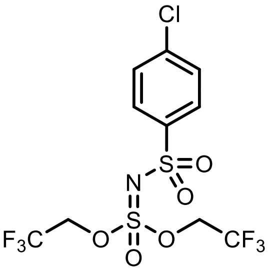


Following the general procedure afforded the product as a white solid (59.8 mg, 69% yield). **m.p.**: 55.5-56.8 °C. **$^{1}$H NMR** (600 MHz, $CDCl_3$) δ 7.90 (d, *J* = 8.6 Hz, 2H), 7.53 (d, *J* = 8.6 Hz, 2H), 4.69 (q, *J* = 7.4 Hz, 4H). **$^{13}$C{$^{1}$H} NMR** (151 MHz, $CDCl_3$) δ 140.3, 139.2, 129.5, 128.5, 120.9 (q, *J* = 277.9 Hz), 68.5 (q, *J* = 39.9 Hz), 29.7. **$^{19}$F NMR** (565 MHz, $CDCl_3$) δ –73.54 (t, *J* = 7.5 Hz). **IR** (ATR): ν = 2920, 2852, 1585, 1421, 1339, 1274, 1162, 1136, 999, 967, 888, 766. **MS** (EI, 70 eV): *m/z* (%) = 434.5 (6), 307.7 (26), 177.0 (7), 174.8 (26), 111.0 (73), 83.0 (100), 75.0 (79), 53.9 (68). **HRMS** (ESI) *m/z:* $[M+Na]^+$ Calcd for $C_{10}H_8O_5NClF_6NaS_2^+$ 457.9329; Found, 457.9318.

**Bis(2,2,2-trifluoroethyl) [(4-bromophenyl) sulfonyl] sulfurimidate (4o)**

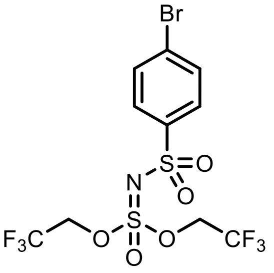


Following the general procedure afforded the product as a white solid (64.3 mg, 67% yield). **m.p.**: 68.4-69.3 °C. **$^{1}$H NMR** (600 MHz, $CDCl_3$) δ 7.82 (d, *J* = 8.6 Hz, 2H),

7.69 (d, $J$ = 8.6 Hz, 2H), 4.69 (q, $J$ = 7.4 Hz, 4H). **$^{13}$C{$^{1}$H} NMR** (151 MHz, $CDCl_3$) δ 139.7, 132.5, 128.8, 128.6, 120.9 (q, $J$ = 277.4 Hz), 68.6 (q, $J$ = 39.7 Hz), 29.7. **$^{19}$F NMR** (565 MHz, $CDCl_3$) δ –73.53 (t, $J$ = 7.2 Hz). **IR** (ATR): ν = 2922, 2853, 1575, 1421, 1348, 1287, 1167, 1132, 1019, 989, 887, 760. **MS** (EI, 70 eV): *m/z* (%) = 480.9 (36), 480.1 (15), 478.7 (40), 414.8 (8), 307.9 (100), 220.9 (9), 156.8 (9), 83.0 (7). **HRMS** (ESI) *m/z:* $[M+Na]^+$ Calcd for $C_{10}H_8O_5NBrF_6NaS_2^+$ 501.8824; Found, 501.8813

**Bis(2,2,2-trifluoroethyl) {[4-(trifluoromethyl) phenyl] sulfonyl} sulfurimidate (4p)**

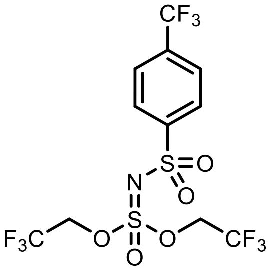

Following the general procedure afforded the product as a white solid (70.1 mg, 75% yield). **m.p.**: 62.9-64.1 °C. **$^{1}$H NMR** (600 MHz, $CDCl_3$) δ 8.09 (d, $J$ = 8.3 Hz, 2H), 7.82 (d, $J$ = 8.3 Hz, 2H), 4.70 (q, $J$ = 7.4 Hz, 4H). **$^{13}$C{$^{1}$H} NMR** (151 MHz, $CDCl_3$) δ 144.1, 135.28 (q, $J$ = 33.8 Hz), 127.6, 126.4 (q, $J$ = 3.7 Hz), 122.9 (q, $J$ = 215.7 Hz), 119.0 (q, $J$ = 278.5 Hz), 68.7 (q, $J$ = 39.8). **$^{19}$F NMR** (376 MHz, $CDCl_3$) δ –63.35 (3F), –73.63 (t, $J$ = 7.6 Hz, 6F). **IR** (ATR): ν = 2921, 2852, 1731, 1408, 1334, 1280, 1167, 1135, 1026, 989, 961, 841, 710. **MS** (EI, 70 eV): *m/z* (%) = 309.0 (13), 307.8 (100), 271.9 (14), 209.1 (32), 159.0 (21), 145.0 (93), 126.1 (10), 83.2 (32), 50.7(5). **HRMS** (ESI) *m/z:* $[M+Na]^+$ Calcd for $C_{11}H_8O_5NF_9NaS_2^+$ 491.9592; Found, 491.9583.

**Bis(2,2,2-trifluoroethyl) (*o*-tolylsulfonyl) sulfurimidate (4q)**

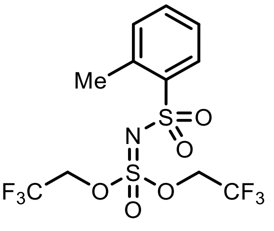

Following the general procedure afforded the product as a white solid (68.7 mg, 83% yield). **m.p.**: 44.7-46.8 °C. **$^{1}$H NMR** (600 MHz, $CDCl_3$) δ 8.08 – 7.89 (m, 1H), 7.51 (td, $J$ = 7.5, 1.4 Hz, 1H), 7.35 (d, $J$ = 7.6 Hz, 2H), 4.67 (qd, $J$ = 7.5, 1.4 Hz, 4H), 2.70 (s, 3H). **$^{13}$C{$^{1}$H} NMR** (151 MHz, $CDCl_3$) δ 139.0, 137.7, 133.6, 132.6, 128.5, 126.2, 120.9 (q, $J$ = 277.3 Hz), 68.3 (q, $J$ = 39.8 Hz), 20.1. **$^{19}$F NMR** (565 MHz, $CDCl_3$) δ –73.62 (t, $J$ = 7.7 Hz). **IR** (ATR): ν =3043, 2981, 1449, 1419, 1337, 1279, 1140, 1003, 963, 843, 739. **MS** (EI, 70 eV): *m/z* (%) = 414.9 (67), 351.0 (19), 350.0 (39), 307.8 (6), 155.0 (23),137.0 (31), 90.0 (100), 78.1 (21), 65.9 (20), 54.5 (11). **HRMS** (ESI) *m/z:* $[M+Na]^+$ Calcd for $C_{11}H_{11}O_5NF_6NaS_2^+$ 437.9875; Found, 437.9859.

**Bis(2,2,2-trifluoroethyl) benzoylsulfurimidate (4r)**

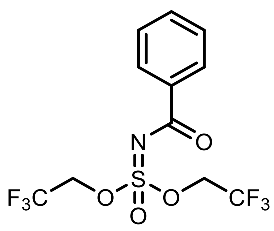


Following the general procedure afforded the product as a colorless oil (62.9 mg, 86% yield). **$^{1}$H NMR** (600 MHz, $CDCl_3$) δ 8.11 – 7.98 (m, 2H), 7.66 – 7.51 (m, 1H), 7.45 (t, *J* = 7.6 Hz, 2H), 4.79 (p, *J* = 7.8 Hz, 4H). **$^{13}$C{$^{1}$H} NMR** (151 MHz, $CDCl_3$) δ 170.4, 133.5, 133.3, 129.9, 128.4, 121.3 (q, *J* = 277.4 Hz), 67.9 (q, *J* = 39.3 Hz). **$^{19}$F NMR** (565 MHz, $CDCl_3$) δ –73.65 (t, *J* = 7.6 Hz). **IR** (ATR): ν = 2921, 1662, 1412, 1334, 1269, 1167, 1015, 959, 848, 708. **MS** (EI, 70 eV): *m/z* (%) = 364.9 (16), 287.9 (8), 169.1 (7), 105.0 (100), 83.0 (24), 77.1 (43), 54.4 (47). **HRMS** (ESI) *m/z:* $[M+Na]^+$ Calcd for $C_{11}H_9O_4NF_6NaS^+$ 388.0049; Found, 388.0037.

**Bis(2,2,2-trifluoroethyl) (4-chlorobenzoyl) sulfurimidate (4s)**

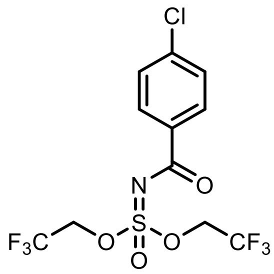


Following the general procedure afforded the product as a white solid (65.2 mg, 82% yield). **m.p.**: 41.2-43.8 °C. **$^{1}$H NMR** (600 MHz, $CDCl_3$) δ 8.00 (d, *J* = 8.6 Hz, 2H), 7.41 (d, *J* = 8.6 Hz, 2H), 4.78 (p, *J* = 7.4 Hz, 4H). **$^{13}$C{$^{1}$H} NMR** (151 MHz, $CDCl_3$) δ 169.4, 140.0, 131.8, 131.2, 128.7, 121.3 (q, *J* = 278.4 Hz), 68.0 (q, *J* = 38.9 Hz). **$^{19}$F NMR** (565 MHz, $CDCl_3$) δ –73.68 (t, *J* = 7.4 Hz). **IR** (ATR): ν = 2988, 1654, 1519, 1403, 1275, 1156, 1010, 956, 876, 837, 761. **MS** (EI, 70 eV): *m/z* (%) = 400.7 (10), 398.8 (23), 287.8 (9), 201.0 (11), 140.9 (36), 138.9 (100), 111.0 (19), 83.0 (16), 75.0 (12), 53.9 (6). **HRMS** (ESI) *m/z:* $[M+Na]^+$ Calcd for $C_{11}H_8O_4NClF_6NaS^+$ 421.9659; Found, 421.9648.

**Bis(2,2,2-trifluoroethyl) (2,4-dichlorobenzoyl) sulfurimidate (4t)**

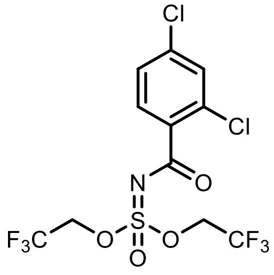


Following the general procedure afforded the product as a colorless oil (60.3 mg, 70% yield). **$^{1}$H NMR** (600 MHz, $CDCl_3$) δ 7.87 (d, *J* = 8.4 Hz, 1H), 7.47 (d, *J* = 2.0 Hz, 1H), 7.30 (dd, *J* = 8.4, 2.0 Hz, 1H), 4.93 – 4.70 (m, 4H). **$^{13}$C{$^{1}$H} NMR** (151 MHz,

$CDCl_3$) δ 168.6, 138.6, 134.7, 133.0, 131.2, 131.2, 127.1, 121.2 (q, *J* = 277.4 Hz), 68.2 (q, *J* = 39.2 Hz). **$^{19}$F NMR** (565 MHz, $CDCl_3$) δ –73.66 (t, *J* = 7.4 Hz). **IR** (ATR): ν = 2985, 1670, 1584, 1412, 1334, 1276, 1166, 1011, 959, 840, 768. **MS** (EI, 70 eV): *m/z* (%) = 299.9 (2), 287.9 (9), 178.0 (12), 174.9 (71), 172.9 (100), 147.0 (8), 109.0 (6), 83.1 (20). **HRMS** (ESI) *m/z:* $[M+Na]^+$ Calcd for $C_{11}H_7O_4NCl_2F_6NaS^+$ 455.9269; Found, 455.9255.

**Bis(2,2,2-trifluoroethyl) (benzylsulfonyl) sulfurimidate (4u)**

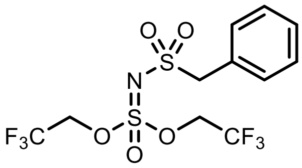

Following the general procedure afforded the product as a colorless oil (53.5 mg, 64% yield). **m.p.**: 51.2-53.4 °C. **$^1$H NMR** (600 MHz, $CDCl_3$) δ 7.66 – 7.34 (m, 5H), 4.55 – 4.45 (m, 4H), 4.44 (s, 2H). **$^{13}$C{$^1$H} NMR** (151 MHz, $CDCl_3$) δ 130.9, 129.2, 128.8, 128.3, 120.9 (q, *J* = 277.3 Hz), 68.3 (q, *J* = 39.6 Hz), 61.6. **$^{19}$F NMR** (565 MHz, $CDCl_3$) δ –73.70 (t, *J* = 7.5 Hz). **IR** (ATR): ν = 3035, 2987, 1450, 1419, 1332, 1281, 1150, 1006, 960, 854, 784, 698. **HRMS** (ESI) *m/z:* $[M+Na]^+$ Calcd for $C_{11}H_{11}O_5NF_6NaS_2^+$ 437.9875; Found, 437.9859.

***N*-(5,5-Dimethyl-2-oxido-1,3,2λ$^6$-dioxathian-2-ylidene)-4-methylbenzenesulfonamide (4v)**

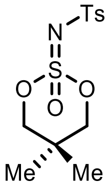

Following the general procedure afforded the product as a white solid (55.9 mg, 88% yield). **m.p.**: 110.3-112.3 °C. **$^1$H NMR** (600 MHz, $CDCl_3$) δ 7.83 (d, *J* = 8.0 Hz, 2H), 7.28 (d, *J* = 8.0 Hz, 2H), 4.58 (d, *J* = 11.0 Hz, 2H), 4.37 (d, *J* = 11.1 Hz, 2H), 2.40 (s, 3H), 1.18 (s, 3H), 1.13 (s, 3H). **$^{13}$C{$^1$H} NMR** (151 MHz, $CDCl_3$) δ 143.6, 139.0, 129.3, 126.8, 83.5, 30.7, 21.5, 21.2, 20.2. **IR** (ATR): ν = 2924, 1596, 1459, 1377, 1299, 1150, 1090, 938, 824, 715, 661. **MS** (EI, 70 eV): *m/z* (%) = 319.3 (75), 318.8 (43), 234.0 (5), 171.0 (7), 155.0 (100), 91.1 (66), 69.3 (54), 58.6 (21). **HRMS** (ESI) *m/z:* $[M+Na]^+$ Calcd for $C_{11}H_{17}O_5NNaS_2^+$ 342.0440; Found, 342.0433.

**4-Methyl-*N*-[phenyl(2,2,2-trifluoroethoxy)-λ$^4$-sulfanylidene]benzenesulfonamide (6a)**

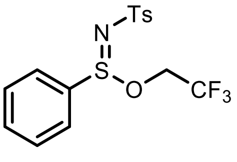

Following the general procedure afforded the product as a white solid (281.5 mg, 75%

yield) and (3.09 g, 82% yield, 10 mmol scale). **m.p.**: 116.3-117.8 °C **$^1$H NMR** (600 MHz, $CDCl_3$) δ 7.91 – 7.84 (m, 4H), 7.71 – 7.66 (m, 1H), 7.65 – 7.59 (m, 2H), 7.32 (d, *J* = 8.1 Hz, 2H), 4.34 – 4.24 (m, 1H), 3.91 – 3.82 (m, 1H), 2.43 (s, 3H). **$^{13}C\{^1H\}$ NMR** (151 MHz, $CDCl_3$) δ 143.2, 139.5, 134.5, 129.9, 129.6, 127.5, 126.5, 122.6 (q, *J* = 278.0 Hz), 61.0 (q, *J* = 37.1 Hz), 21.5. **$^{19}F$ NMR** (565 MHz, $CDCl_3$) δ –73.13 (t, *J* = 8.2 Hz). **IR** (ATR): ν = 3065, 2956, 1447, 1406, 1279, 1150, 1037, 990, 910, 716, 670. **MS** (EI, 70 eV): *m/z* (%) = 378.4 (19), 376.9 (70), 373.2 (13), 277.9 (75), 232.0 (30), 221.9 (100), 214.1 (31), 198.9 (20), 154.9 (28), 139.0 (90), 125.0 (14), 122.9 (17), 91.0 (78), 77.0 (28), 65.9 (18), 54.5 (20). **HRMS** (ESI) *m/z:* $[M+Na]^+$ Calcd for $C_{15}H_{14}O_3NF_3NaS_2^+$ 400.0259; Found, 400.0252.

**4-Methyl-*N*-[(4-methylphenyl)(2,2,2-trifluoroethoxy)-λ$^4$-sulfanylidene]benzenesulfonamide (6b)**

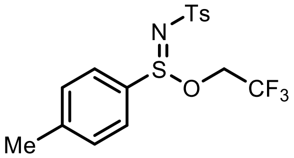


Following the general procedure afforded the product as a colorless oil (315.6 mg, 81% yield). **$^1$H NMR** (600 MHz, $CDCl_3$) δ 7.85 (d, *J* = 8.1 Hz, 2H), 7.71 (d, *J* = 8.2 Hz, 2H), 7.38 (d, *J* = 8.2 Hz, 2H), 7.28 (d, *J* = 8.0 Hz, 2H), 4.27 – 4.17 (m, 1H), 3.86 – 3.76 (m, 1H), 2.43 (s, 3H), 2.39 (s, 3H). **$^{13}C\{^1H\}$ NMR** (151 MHz, $CDCl_3$) δ 145.3, 143.0, 139.5, 131.0, 130.5, 129.4, 127.2, 126.3, 122.5 (q, *J* = 277.9 Hz), 60.3 (q, *J* = 37.0 Hz), 21.4, 21.3. **$^{19}F$ NMR** (565 MHz, $CDCl_3$) δ –73.1 (t, *J* = 8.1 Hz). **IR** (ATR): ν = 3357, 3259, 1596, 1403, 1281, 1152, 1036, 993, 811, 668. **MS** (EI, 70 eV): *m/z* (%) = 391.1 (1), 236.1 (6), 155.1 (8), 139.0 (51), 137.0 (15), 91.0 (100), 89.1 (12), 77.1 (16), 65.9 (62), 64.2 (17), 54.5 (29), 45.3 (40). **HRMS** (ESI) *m/z:* $[M+Na]^+$ Calcd for $C_{16}H_{16}O_3NF_3NaS_2^+$ 414.0416; Found, 414.0408.

**4-Methyl-*N*-[(4-fluorophenyl)(2,2,2-trifluoroethoxy)-λ$^4$-sulfanylidene]benzenesulfonamide (6c)**

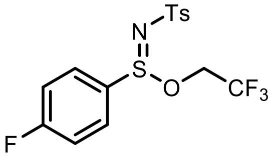


Following the general procedure afforded the product as a colorless oil (293.5 mg, 74% yield). **$^1$H NMR** (600 MHz, $CDCl_3$) δ 7.91 – 7.83 (m, 4H), 7.32 – 7.26 (m, 4H), 4.34 – 4.22 (m, 1H), 3.96 – 3.84 (m, 1H), 2.40 (s, 3H). **$^{13}C\{^1H\}$ NMR** (151 MHz, $CDCl_3$) δ 165.9 (d, *J* = 257.7 Hz), 143.2, 139.2, 130.0 (d, *J* = 9.8 Hz), 129.5, 126.3, 122.4 (q, *J* = 278.0 Hz), 117.3 (d, *J* = 23.0 Hz), 60.9 (q, *J* = 37.0 Hz), 21.3. **$^{19}F$ NMR** (564 MHz, $CDCl_3$) δ -73.19 (t, *J* = 8.1 Hz), –102.55 – –102.62 (m). **IR** (ATR): ν = 3102, 1590, 1491, 1280, 1152, 992, 840, 776, 728. **MS** (EI, 70 eV): *m/z* (%) = 394.9 (44), 296.1 (100), 232.2 (27), 155.2 (29), 139.2 (91), 91.2 (99), 65.3 (33), 51.3 (7). **HRMS** (ESI) *m/z:* $[M+Na]^+$ Calcd for $C_{15}H_{13}O_3NF_4NaS_2^+$ 418.0165; Found, 418.0166.

**4-Methyl-*N*-[(4-chlorophenyl)(2,2,2-trifluoroethoxy)-λ$^4$-sulfanylidene]benzenesulfonamide (6d)**

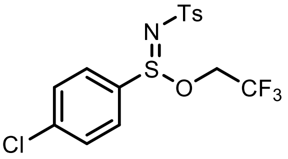


Following the general procedure afforded the product as a white solid (341.4 mg, 83% yield). **m.p.**: 113.8-115.2 °C. **$^1$H NMR** (600 MHz, $CDCl_3$) δ 7.83 (d, *J* = 8.3 Hz, 2H), 7.77 (d, *J* = 8.7 Hz, 2H), 7.54 (d, *J* = 8.7 Hz, 2H), 7.28 (d, *J* = 8.0 Hz, 2H), 4.32 – 4.22 (m, 1H), 3.93 – 3.82 (m, 1H), 2.39 (s, 3H). **$^{13}$C{$^1$H} NMR** (151 MHz, $CDCl_3$) δ 143.3, 140.9, 139.2, 132.9, 130.2, 129.6, 128.7, 126.4, 122.4 (q, *J* = 278.0 Hz), 61.2 (q, *J* = 37.0 Hz), 21.4. **$^{19}$F NMR** (565 MHz, $CDCl_3$) δ –73.12 (t, *J* = 8.2 Hz). **IR** (ATR): ν = 3090, 2956, 1574, 1474, 1396, 1279, 1152, 1037, 992, 815, 776, 669. **MS** (EI, 70 eV): *m/z* (%) = 410.2 (9), 311.9 (30), 265.9 (13), 255.9 (49), 249.6 (31), 158.0 (18), 154.9 (31), 138.9 (100), 110.9 (14), 91.1 (78), 65.9 (16). **HRMS** (ESI) *m/z:* $[M+Na]^+$ Calcd for $C_{15}H_{13}O_3NClF_3NaS_2^+$ 433.9870; Found, 433.9860.

***N*-[Methoxy(phenyl)-λ$^4$-sulfanylidene]-4-methylbenzenesulfonamide (6e)**

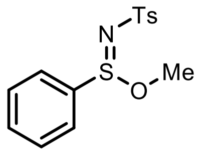


Following the general procedure afforded the product as a colorless oil (252.1 mg, 82% yield). **$^1$H NMR** (600 MHz, $CDCl_3$) δ 7.81 (d, *J* = 8.3 Hz, 2H), 7.77 – 7.73 (m, 2H), 7.60 – 7.55 (m, 1H), 7.53 – 7.47 (m, 2H), 7.23 (d, *J* = 8.0 Hz, 2H), 3.30 (s, 3H), 2.34 (s, 3H). **$^{13}$C{$^1$H} NMR** (151 MHz, $CDCl_3$) δ 142.4, 140.2, 134.4, 133.2, 129.5, 129.2, 127.3, 126.2, 50.1, 21.3. **MS** (EI, 70 eV): *m/z* (%) = 309.1 (17), 278.1 (27), 232.1 (17), 214.1 (16), 155.1 (28), 154.1 (100), 139.1 (60), 123.0 (13), 91.2 (92), 65.2 (36), 51.2 (17). **HRMS** (ESI) *m/z:* $[M+Na]^+$ Calcd for $C_{14}H_{15}O_3NNaS_2^+$ 332.0386; Found, 332.0375.

***N*-[Ethoxy(phenyl)-λ$^4$-sulfanylidene]-4-methylbenzenesulfonamide (6f)**

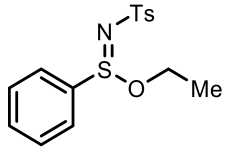


Following the general procedure afforded the product as a colorless oil (246.0 mg, 76% yield). **$^1$H NMR** (600 MHz, $CDCl_3$) δ 7.85 – 7.79 (m, 2H), 7.78 – 7.74 (m, 2H), 7.62 – 7.54 (m, 1H), 7.54 – 7.48 (m, 2H), 7.27 – 7.19 (m, 2H), 3.98 (dq, *J* = 9.8, 7.1 Hz, 1H), 3.55 (dq, *J* = 9.8, 7.0 Hz, 1H), 2.36 (s, 3H), 1.08 (t, *J* = 7.1 Hz, 3H). **$^{13}$C{$^1$H} NMR** (151 MHz, $CDCl_3$) δ 142.3, 140.4, 135.4, 133.0, 129.5, 129.2, 127.2, 126.2, 61.7, 21.3, 14.6. **IR** (ATR): ν = 2984, 1597, 1445, 1388, 1298, 1146, 1088, 1020, 987, 870, 665. **MS** (EI, 70 eV): *m/z* (%) = 323.1 (2), 168.0 (12), 154.9 (13), 140.0 (23),

92.1 (19), 91.0 (100), 77.1 (80), 77.1 (16), 65.9 (69), 54.5 (94), 45.3 (38). **HRMS** (ESI) *m/z:* $[M+Na]^+$ Calcd for $C_{15}H_{17}O_3NNaS_2^+$ 346.0542; Found, 346.0535.

### *N*-[2-Bromoethoxy(phenyl)-λ⁴-sulfanylidene]-4-methylbenzenesulfonamide (6g)

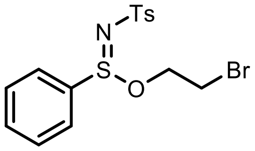


Following the general procedure afforded the product as a colorless oil (290.1 mg, 72% yield).**$^1$H NMR** (600 MHz, $CDCl_3$) δ 7.88 – 7.82 (m, 4H), 7.66 – 7.61 (m, 1H), 7.60 – 7.54 (m, 2H), 7.29 (d, *J* = 8.0 Hz, 2H), 4.28 – 4.20 (m, 1H), 3.82 – 3.72 (m, 1H), 3.34 – 3.22 (m, 2H), 2.40 (s, 3H). **$^{13}$C{$^1$H} NMR** (151 MHz, $CDCl_3$) δ 142.7, 140.0, 135.0, 133.4, 129.6, 129.4, 127.3, 126.3, 64.3, 28.3, 21.4. **IR** (ATR): ν = 3062, 2926, 1596, 1446, 1290, 1146, 1087, 1015, 928, 811, 666. **MS** (EI, 70 eV): *m/z* (%) = 401.0 (7), 399.6 (3), 278.0 (28), 277.1 (20), 247.7 (16), 214.0 (24), 165.9 (12), 154.9 (54), 139.0 (57), 125.0 (30), 108.9 (37), 107.0 (28), 91.1 (100), 77.1 (37), 65.9 (25), 54.5 (23). **HRMS** (ESI) *m/z:* $[M+Na]^+$ Calcd for $C_{15}H_{16}O_3NBrNaS_2^+$ 423.9647; Found, 423.9640.

### *N*-[(2,2-Dimethylpropoxy)(phenyl)-λ⁴-sulfanylidene]-4-methylbenzenesulfon-amide (6h)

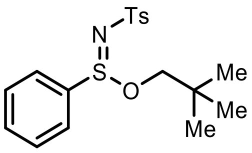


Following the general procedure using 2.2 equiv of neopentyl alcohol and 2.2 equiv of $NEt_3$ afforded the product as a colorless solid (274 mg, 75% yield). **TLC** Rf 0.30 in hex/EtOAc 7:3. **m.p.**: 79-81°C. **$^1$H NMR** (400 MHz, $CDCl_3$) δ 7.89 – 7.84 (m, 2H), 7.82 – 7.78 (m, 2H), 7.64 – 7.51 (m, 3H), 7.29 – 7.23 (m, 2H), 3.59 (d, *J* = 9.0 Hz, 1H), 3.04 (d, *J* = 9.0 Hz, 1H), 2.39 (s, 3H), 0.78 (s, 9H). **$^{13}$C{$^1$H} NMR** (101 MHz, $CDCl_3$) δ 142.4, 140.7, 135.6, 133.0, 129.5, 129.4, 127.5, 126.5, 74.1, 31.6, 26.2, 21.4. **IR** (ATR): ν = 2959 (m, CH stretch), 1597 (w, C=C stretch), 1447 (m, $CH_2$ bend), 1311 (vs, O=S=O bend), 1148 (vs, C-S stretch), 1088 (O=S=O symm. stretch), 1009 (vs, N=S-N asymm. stretch). **HRMS** (ESI) *m/z:* $[M+Na]^+$ Calcd for $C_{18}H_{23}NNaO_3S_2^+$ 388.1012 Found 388.1002.

### 4-Methyl-*N*-[phenyl({[(1*S*,2*R*,4*S*)-1,7,7-trimethylbicyclo[2.2.1]heptan-2-yl]oxy})-λ⁴-sulfanylidene]benzenesulfonamide (6i)

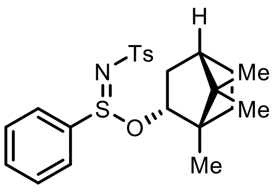


Following the general procedure using 2.2 equiv of (+)-borneol (CAS 464-43-7) and

2.2 equiv of $NEt_3$ on a 0.5 mmol scale afforded the product as a waxy solid (158 mg, 73% yield), in a 54:46 mixture of diastereoisomers. **TLC** Rf 0.35 in hex/EtOAc 7:3. **m.p.**: 88-90°C. **$^1$H NMR** (400 MHz, $CDCl_3$) δ 7.91 – 7.78 (m, 4H), 7.64 – 7.53 (m, 3H), 7.30 – 7.25 (m, 2H), 4.74 (ddd, *J* = 10.1, 3.5, 2.1 Hz, 0.5H), 4.37 (ddd, *J* = 9.6, 3.2, 2.2 Hz, 0.5H), 2.41 (s, 3H), 2.39 – 2.31 (m, 0.5H), 1.95 – 1.86 (m, 0.5H), 1.87 – 1.76 (m, 1H), 1.76 – 1.65 (m, 1.5H), 1.59 (t, *J* = 4.5 Hz, 1H), 1.32 – 1.13 (m, 2.5H), 0.98 (dd, *J* = 14.0, 3.4 Hz, 0.5H), 0.92 (s, 1.5H), 0.86 (s, 1.5H), 0.82 (s, 3H), 0.74 (s, 1.5H), 0.58 (s, 1.5H). **$^{13}$C{$^1$H} NMR** (101 MHz, $CDCl_3$) δ 142.2, 142.1, 140.8, 140.7, 137.0, 136.6, 132.88, 132.87, 129.35, 129.28, 129.2, 127.2, 126.9, 126.2, 126.1, 86.5, 85.5, 49.9, 49.6, 47.74, 47.65, 44.7, 44.6, 37.0, 36.4, 27.7, 27.6, 26.5, 26.4, 21.3, 19.50, 19.45, 18.6, 18.5, 13.2, 12.7. **IR** (ATR): ν = 2954 (m, CH stretch), 1597 (w, C=C stretch), 1446 (m, $CH_2$ bend), 1299 (s, O=S=O asymm. stretch), 1145 (vs, C-S stretch), 1088 (s, O=S=O symm. stretch). **HRMS** (ESI) *m/z:* $[M+Na]^+$ Calcd for $C_{23}H_{29}NNaO_3S_2^+$ 454.1481; Found, 454.1480.

***N*-({[(1*R*,5*R*)-2-Isopropyl-5-methylcyclohexyl]oxy}(phenyl)-λ$^4$-sulfanylidene)-4-methylbenzenesulfonamide (6j)**

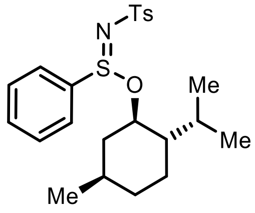


Following the general procedure using 2.2 equiv of (–)-menthol (CAS 2216-51-5) and 2.2 equiv of $NEt_3$ afforded the product as a colorless oil (261 mg, 60% yield), in a 62:38 mixture of diastereoisomers. **TLC** Rf 0.4 in hex/EtOAc 7:3. **$^1$H NMR** (600 MHz, $CDCl_3$) δ 7.81 – 7.73 (m, 2H), 7.70 – 7.63 (m, 2H), 7.49 – 7.37 (m, 3H), 7.18 – 7.10 (m, 2H), 4.24 (td, *J* = 10.8, 4.4 Hz, 0.4H), 4.16 (td, *J* = 10.7, 4.3 Hz, 0.6H), 2.41 – 2.32 (m, 0.6H), 2.26 (d, J = 4.1 Hz, 3H), 2.01 – 1.90 (m, 1H), 1.87 – 1.80 (m, 0.4H), 1.62 – 1.50 (m, 1.7H), 1.44 – 1.35 (m, 0.6H), 1.32 – 1.19 (m, 1.5H), 1.05 (q, *J* = 11.8 Hz, 0.6H), 0.98 – 0.89 (m, 1.4H), 0.89 – 0.80 (m, 4.4H), 0.78 – 0.69 (m, 4H), 0.62 (d, *J* = 6.9 Hz, 1.8H). **$^{13}$C{$^1$H} NMR** (151 MHz, $CDCl_3$) δ 141.91, 141.88, 140.7, 140.5, 137.7, 136.9, 132.6, 132.5, 129.12, 129.09, 128.97, 128.96, 126.6, 126.2, 125.9, 83.4, 81.0, 48.1, 47.6, 42.0, 41.3, 33.5, 33.3, 31.4, 31.1, 25.2, 24.9, 22.9, 22.5, 21.7, 21.4, 21.03, 21.02, 20.6, 20.5, 15.5, 15.3. **IR** (ATR): ν = 2954, 2869 (m, CH stretch), 1598 (w, C=C stretch), 1446 (m, $CH_2$ bend), 1299 (s, O=S=O asymm. stretch), 1145 (vs, C-S stretch), 1089 (O=S=O symm. stretch). **HRMS** (ESI) *m/z:* $[M+H]^+$ Calcd for $C_{23}H_{32}NO_3S_2^+$ 434.1818; Found 434.1817.

**4-Bromo-*N*-[phenyl(2,2,2-trifluoroethoxy)-λ⁴-sulfanylidene]benzenesulfonamide (6k)**

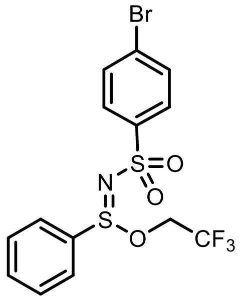

Following the general procedure afforded the product as a white solid (358.1 mg, 81% yield). **m.p.**: 146.7-148.2 °C. **$^{1}$H NMR** (600 MHz, $CDCl_3$) δ 7.87 – 7.79 (m, 4H), 7.71 – 7.66 (m, 1H), 7.65 – 7.58 (m, 4H), 4.33 (dq, *J* = 12.3, 8.1 Hz, 1H), 3.87 (dq, *J* = 12.3, 8.1 Hz, 1H). **$^{13}$C{$^{1}$H} NMR** (151 MHz, $CDCl_3$) δ 141.4, 134.3, 134.1, 132.2, 130.0, 128.1, 127.4, 127.3, 122.5 (q, *J* = 277.9 Hz), 61.1 (q, *J* = 37.1 Hz). **$^{19}$F NMR** (565 MHz, $CDCl_3$) δ –73.09 (t, *J* = 8.2 Hz). **IR** (ATR): ν = 3069, 2955, 1574, 1473, 1447, 1389, 1276, 1153, 1037, 989, 789, 735. **MS** (EI, 70 eV): *m/z* (%) = 442.6 (34), 440.8 (26), 343.7 (87), 297.8 (42), 295.7 (49), 222.0 (100), 202.9 (45), 199.0 (25), 123.0 (13), 77.1 (10), 54.7 (7). **HRMS** (ESI) *m/z:* $[M+Na]^+$ Calcd for $C_{14}H_{11}O_3NBrF_3NaS_2^+$ 463.9208; Found, 463.9201.

***N*-[Phenyl(2,2,2-trifluoroethoxy)-λ⁴-sulfanylidene]-4-(trifluoromethyl)benzene-sulfonamide (6l)**

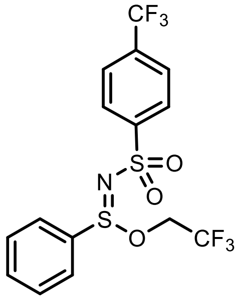

Following the general procedure afforded the product as a white solid (356.4 mg, 83% yield). **m.p.**: 129.7-132.5 °C. **$^{1}$H NMR** (600 MHz, $CDCl_3$) δ 8.11 (d, *J* = 8.2 Hz, 2H), 7.85 (d, *J* = 7.6 Hz, 2H), 7.77 (d, *J* = 8.3 Hz, 1H), 7.72 – 7.68 (m, 2H), 7.62 (t, *J* = 7.9 Hz, 2H), 4.39 – 4.29 (m, 1H), 3.92 – 3.82 (m, 1H). **$^{13}$C{$^{1}$H} NMR** (151 MHz, $CDCl_3$) δ 145.8, 134.4, 134.2 (q, *J* = 33.0 Hz), 134.0, 130.1, 127.4, 127.1, 126.2 (q, *J* = 3.8 Hz), 123.3 (q, *J* = 278.0 Hz), 122.4 (q, *J* = 278.0 Hz), 61.2 (q, *J* = 37.1 Hz). **$^{19}$F NMR** (564 MHz, $CDCl_3$) δ –63.16 (3F), –73.19 (t, *J* = 8.0 Hz, 3F). **IR** (ATR): ν = 3070, 1807, 1447, 1404, 1321, 1281, 1156, 1038, 990, 714. **MS** (EI, 70 eV): *m/z* (%) = 432.1 (6), 431.4 (1), 430.8 (25), 332.9 (20), 331.9 (100), 285.9 (65), 283.3 (11), 222.1 (77), 198.9 (17), 192.9 (30),145.0 (21), 123.0 (16), 77.1 (10), 54.5 (8). **HRMS** (ESI) *m/z:* $[M+Na]^+$ Calcd for $C_{15}H_{11}O_3NF_6NaS_2^+$ 453.9977; Found, 453.9962.

**$N$-(3$H$-2,1 λ$^4$-Benzoxathiol-1-ylidene)-4-methylbenzenesulfonamide (6m)**

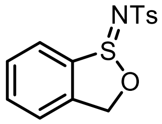


An adapted form of the general procedure was followed: 2-mercaptobenzyl alcohol (CAS 4521-31-7, 140 mg, 1.0 mmol) and $Et_3N$ (306 µL, 2.2 mmol) were dissolved in DCE (2 mL). While stirring at 0 °C, a separate solution of $TsNCl_2$ (240 mg, 1.0 mmol) in DCE (1 mL) was added dropwise to the reaction mixture over 10 min, and the reaction was allowed to warm to room temperature and reacted overnight. The usual purification afforded the product as a colorless solid (60 mg, 20% yield). **TLC** Rf 0.25 in hex/EtOAc 5:5. **m.p.**: 174-176 °C. **$^1$H NMR** (400 MHz, $CDCl_3$) δ 7.75 (d, J = 8.0 Hz, 2H), 7.65 – 7.57 (m, 2H), 7.55 – 7.45 (m, 2H), 7.25 (d, J = 8.0 Hz, 2H), 5.82 (d, J = 13.2 Hz, 1H), 5.46 (d, J = 13.2 Hz, 1H), 2.40 (s, 3H). **$^{13}$C{$^1$H} NMR** (101 MHz, $CDCl_3$) δ 142.6, 140.0, 138.4, 136.5, 132.9, 129.9, 129.3, 126.4, 124.2, 122.1, 21.4. **IR** (ATR): ν = 2968 (w, CH stretch), 1596 (w, C=C stretch), 1451 (w, C=C stretch), 1288 (vs, O=S=O asymm. stretch), 1143 (vs, C-S stretch), 1088 (m, O=S=O symm. stretch), 993 (vs, N=S stretch). **HRMS** (ESI) *m/z:* $[M+H]^+$ Calcd for $C_{14}H_{14}NO_3S_2^+$ 308.0410, Found 308.0412.

**4-Methyl-$N$- [ phenyl (piperidin-1-yl)-λ$^4$-sulfaneylidene] benzenesulfonamide (7a)**

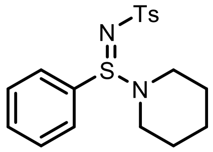


Following the general procedure afforded the product as a white solid (315.7 mg, 87% yield) and (3.18 g, 88% yield, 10 mmol scale). **m.p.**: 116.5-118.6 °C. **$^1$H NMR** (600 MHz, $CDCl_3$) δ 7.84 – 7.78 (m, 2H), 7.73 – 7.67 (m, 2H), 7.51 – 7.43 (m, 3H), 7.20 (d, $J$ = 8.0 Hz, 2H), 3.14 – 3.04 (m, 2H), 2.87 – 2.76 (m, 2H), 2.34 (s, 3H), 1.63 – 1.49 (m, 4H), 1.49 – 1.40 (m, 2H). **$^{13}$C{$^1$H} NMR** (151 MHz, $CDCl_3$) δ 141.5, 134.1, 131.7, 129.3, 129.1, 127.6, 126.1, 47.8, 25.6, 23.2, 21.3. **IR** (ATR): ν = 2947, 2851, 1467, 1442, 1285, 1138, 1088, 976, 905, 760, 660. **MS** (EI, 70 eV): *m/z* (%) = 363.1 (1), 279.2 (41), 277.8 (14), 155.0 (9), 124.1 (35), 91.1 (26), 84.2 (77), 66.0 (11), 57.9 (28), 47.4 (100). **HRMS** (ESI) *m/z:* $[M+H]^+$ Calcd for $C_{18}H_{23}O_2N_2S_2^+$ 363.1195; Found, 363.1193.

**4-Methyl-$N$- [morpholino (phenyl)- λ$^4$-sulfaneylidene] benzenesulfonamide (7b)**

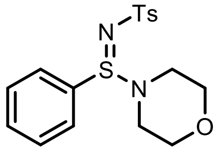


Following the general procedure afforded the product as a white solid (296.3 mg, 81% yield). **m.p.**: 164.5-167.3 °C **$^1$H NMR** (600 MHz, $CDCl_3$) δ 7.84 (d, $J$ = 7.9 Hz, 2H), 7.76 (d, $J$ = 7.5 Hz, 2H), 7.60 – 7.48 (m, 3H), 7.24 (d, $J$ = 7.9 Hz, 2H), 3.81 – 3.60 (m,

4H), 3.20 – 3.13 (m, 2H), 2.90 – 2.79 (m, 2H), 2.38 (s, 3H). **$^{13}$C{$^{1}$H} NMR** (151 MHz, $CDCl_3$) δ 141.9, 141.4, 133.2, 132.2, 129.6, 129.3, 127.8, 126.2, 66.4, 46.5, 21.4. **IR** (ATR): ν = 2919, 2854, 1598, 1446, 1286, 1140, 1108, 1088, 967, 925, 755, 659. **MS** (EI, 70 eV): *m/z* (%) = 279.1 (37), 278.1 (28), 273.0 (45), 195.0 (11), 194.1 (21), 155.1 (17), 124.0 (33), 86.2 (100), 58.8 (61), 47.3 (18). **HRMS** (ESI) *m/z:* $[M+Na]^+$ Calcd for $C_{17}H_{20}O_3N_2NaS_2^+$ 387.0808; Found, 387.0802.

**_N_-{[Benzyl(methyl)amino] (phenyl)-λ$^4$-sulfaneylidene}-4-methylbenzenesulfonamide (7c)**

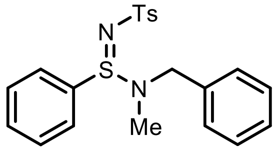


Following the general procedure afforded the product as a colorless oil (295.1 mg, 74% yield). **$^{1}$H NMR** (600 MHz, $CDCl_3$) δ 7.89 (d, *J* = 7.9 Hz, 2H), 7.85 – 7.78 (m, 2H), 7.59 – 7.50 (m, 3H), 7.36 (t, *J* = 7.5 Hz, 2H), 7.34 – 7.30 (m, 1H), 7.29 – 7.23 (m, 4H), 4.34 (d, *J* = 13.8 Hz, 1H), 3.95 (d, *J* = 13.9 Hz, 1H), 2.50 (s, 3H), 2.40 (s, 3H). **$^{13}$C{$^{1}$H} NMR** (151 MHz, $CDCl_3$) δ 141.8, 141.4, 135.6, 134.6, 132.0, 129.5, 129.2, 128.7, 128.7, 128.1, 127.8, 126.3, 56.5, 33.7, 21.4. **IR** (ATR): ν = 2927, 2168, 1597, 1447, 1282, 1141, 1085, 954, 924, 750, 702. **MS** (EI, 70 eV): *m/z* (%) = 280.1 (11), 279.1 (34), 278.1 (48), 214.1 (19), 155.1 (17), 124.1 (70), 120.1 (100), 118.1 (15), 91.2 (69), 77.3 (10), 47.5 (43). **HRMS** (ESI) *m/z:* $[M+H]^+$ Calcd for $C_{21}H_{23}O_2N_2S_2^+$ 399.1195; Found, 399.1192.

**4-Methyl-_N_-{[methyl(phenyl) amino] (phenyl)-λ$^4$-sulfaneylidene} benzenesulfonamide (7d)**

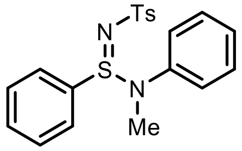


Following the general procedure afforded the product as a yellow oil (239.3 mg, 62% yield). **$^{1}$H NMR** (600 MHz, $CDCl_3$) δ 7.82 – 7.75 (m, 2H), 7.64 (d, *J* = 8.0 Hz, 2H), 7.60 – 7.49 (m, 3H), 7.34 – 7.28 (m, 2H), 7.14 (t, *J* = 7.4 Hz, 1H), 7.11 (d, *J* = 7.7 Hz, 2H), 7.07 (d, *J* = 8.0 Hz, 2H), 2.76 (s, 3H), 2.33 (s, 3H). **$^{13}$C{$^{1}$H} NMR** (151 MHz, $CDCl_3$) δ 144.5, 141.6, 141.1, 134.6, 132.2, 129.6, 129.4, 129.1, 127.4, 126.2, 124.7, 120.5, 31.2, 21.3. **IR** (ATR): ν = 3062, 2925, 1594, 1490, 1446, 1286, 1142, 1086, 965, 749, 663. **MS** (EI, 70 eV): *m/z* (%) = 384.1 (5), 278.9 (65), 277.0 (27), 275.8 (21), 274.9. (21), 232.0 (20), 229.1 (31), 155.1 (14), 124.0 (78), 119.1 (44),109.1 (35), 106.1. (51), 97.1 (70), 77.1 (60), 64.2 (21), 54.5 (100), 49.9 (19), 45.3 (52). **HRMS** (ESI) *m/z:* $[M+H]^+$ Calcd for $C_{20}H_{21}O_2N_2S_2^+$ 385.1039; Found, 385.1035.

***N*-[Azepan-1-yl(phenyl)-λ⁴-sulfaneylidene]-4-methylbenzenesulfonamide (7e)**

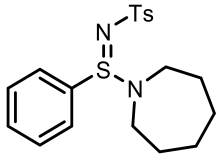

Following the general procedure afforded the product as a white solid (309.4 mg, 82% yield). **m.p.**: 127.7-130.1 °C. **$^1$H NMR** (600 MHz, $CDCl_3$) δ 7.82 (d, *J* = 7.9 Hz, 2H), 7.76 – 7.67 (m, 2H), 7.52 – 7.44 (m, 3H), 7.20 (d, *J* = 7.9 Hz, 2H), 3.34 – 3.23 (m, 2H), 3.06 – 2.95 (m, 2H), 2.34 (s, 3H), 1.62 – 1.47 (m, 6H), 1.50 – 1.33 (m, 2H). **$^{13}$C{$^1$H} NMR** (151 MHz, $CDCl_3$) δ 141.6, 141.4, 134.9, 131.6, 129.2, 129.0, 127.5, 126.1, 49.5, 28.6, 26.9, 21.2. **IR** (ATR): ν = 2928, 2857, 1445, 1383, 1285, 1141, 1086, 943, 763, 657. **MS** (EI, 70 eV): *m/z* (%) = 313.0 (16), 279.0 (49), 158.1 (13), 155.1 (14), 124.1. (40), 98.2 (100), 91.2 (33), 84.2 (32), 57.9 (29), 47.8 (72), 46.9 (94), 45.3(14). **HRMS** (ESI) *m/z:* $[M+H]^+$ Calcd for $C_{19}H_{25}O_2N_2S_2^+$ 377.1352; Found, 377.1349.

***Tert*-butyl 4-(*S*-phenyl-*N*-tosylsulfinimidoyl) piperazine-1-carboxylate (7f)**

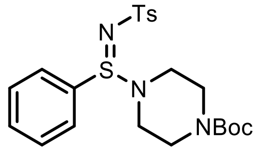

Following the general procedure afforded the product as a white solid (315.9 mg, 68% yield). **m.p.**: 123.0-124.9 °C. **$^1$H NMR** (600 MHz, $CDCl_3$) δ 7.81 (d, *J* = 8.0 Hz, 2H), 7.73 (d, *J* = 7.4 Hz, 2H), 7.56 – 7.48 (m, 3H), 7.22 (d, *J* = 8.0 Hz, 2H), 3.56 – 3.31 (m, 4H), 3.22 – 3.04 (m, 2H), 2.90 – 2.76 (m, 2H), 2.35 (s, 3H), 1.43 (s, 9H). **$^{13}$C{$^1$H} NMR** (151 MHz, $CDCl_3$) δ 154.0, 141.8, 141.1, 133.2, 132.1, 129.5, 129.2, 127.6, 126.1, 80.2, 46.3, 28.2, 21.3. **IR** (ATR): ν = 2976, 2924, 2250, 1669, 1426, 1281, 1251, 1139, 1086, 965, 915, 752, 723. **MS** (EI, 70 eV): *m/z* (%) = 307.1 (8), 279.2 (45), 237.1 (13), 184.1 (21), 129.2 (30), 128.1 (79), 124.1 (62), 91.3 (15), 85.3 (51), 59.5 (100), 46.8 (30). **HRMS** (ESI) *m/z:* $[M+Na]^+$ Calcd for $C_{22}H_{29}O_4N_3NaS_2^+$ 486.1492; Found, 486.1487.

**4-Methyl-*N*-[piperidin-1-yl(*p*-tolyl)- λ⁴-sulfaneylidene] benzenesulfonamide (7g)**

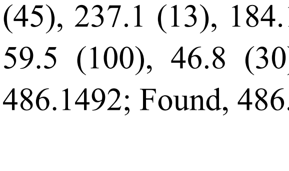

Following the general procedure afforded the product as a white solid (288.1 mg, 77% yield). **m.p.**: 147.9-150.2 °C. **$^1$H NMR** (600 MHz, $CDCl_3$) δ 7.80 (d, *J* = 8.0 Hz, 2H), 7.57 (d, *J* = 8.2 Hz, 2H), 7.26 (d, *J* = 8.2 Hz, 2H), 7.19 (d, *J* = 8.0 Hz, 2H), 3.11 – 3.03 (m, 2H), 2.85 – 2.78 (m, 2H), 2.36 (s, 3H), 2.34 (s, 3H), 1.64 – 1.49 (m, 4H), 1.49 – 1.41 (m, 2H). **$^{13}$C{$^1$H} NMR** (151 MHz, $CDCl_3$) δ 142.4, 141.6, 141.5, 130.9, 130.0, 129.1, 127.5, 126.2, 47.6, 25.7, 23.2, 21.3, 21.2. **IR** (ATR): ν = 2973, 2855, 1492, 1449, 1283, 1139, 1086, 962, 910, 813, 764, 677. **MS** (EI, 70 eV): *m/z* (%) =

377.3 (1), 293.0(46), 291.2 (18), 228.1 (9), 213.0 (9), 138.1 (34), 91.1 (39), 84.2 (58), 58.9 (16), 57.9 (31), 47.4 (100). **HRMS** (ESI) *m/z:* $[M+H]^+$ Calcd for $C_{19}H_{25}O_2N_2S_2^+$ 377.1352; Found, 377.1347.

***N*-[(4-Chlorophenyl)(piperidin-1-yl)-λ⁴-sulfaneylidene]-4-methylbenzenesulfonamide (7h)**

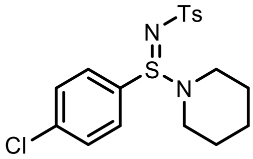


Following the general procedure afforded the product as a white solid (294.0 mg, 74% yield). **m.p.**: 147.9-150.2 °C. **$^1$H NMR** (600 MHz, $CDCl_3$) δ 7.82 (d, *J* = 7.8 Hz, 2H), 7.67 (d, *J* = 8.2 Hz, 2H), 7.47 (d, *J* = 8.2 Hz, 2H), 7.23 (d, *J* = 7.9 Hz, 2H), 3.15 – 3.07 (m, 2H), 2.87 – 2.79 (m, 2H), 2.38 (s, 3H), 1.67 – 1.52 (m, 4H), 1.53 – 1.45 (m, 2H). **$^{13}$C{$^1$H} NMR** (151 MHz, $CDCl_3$) δ 141.7, 141.4, 138.5, 132.8, 129.6, 129.2, 129.1, 126.1, 47.8, 25.7, 23.1, 21.3. **IR** (ATR): ν = 2945, 2856, 1471, 1287, 1142, 1088, 960, 919, 837, 771, 657. **MS** (EI, 70 eV): *m/z* (%) = 314.9 (17), 314.2 (15), 312.8 (53), 248.0 (11), 227.2 (21), 160.1 (20), 158.1 (52), 157.2 (22), 155.0 (25), 91.2 (30), 84.2 (100), 58.0 (16), 47.4 (58). **HRMS** (ESI) *m/z:* $[M+H]^+$ Calcd for $C_{18}H_{22}O_2N_2ClS_2^+$ 397.0806; Found, 397.0800.

***N*-[Phenyl(piperidin-1-yl)- λ⁴-sulfaneylidene] benzenesulfonamide (7i)**

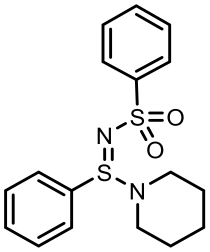


Following the general procedure afforded the product as a white solid (252.9 mg, 73% yield). **m.p.**: 171.1-173.3 °C. **$^1$H NMR** (600 MHz, $CDCl_3$) δ 7.97 – 7.93 (m, 2H), 7.73 – 7.68 (m, 2H), 7.53 – 7.39 (m, 6H), 3.14 – 3.04 (m, 2H), 2.86 – 2.77 (m, 2H), 1.65 – 1.50 (m, 4H), 1.52 – 1.43 (m, 2H). **$^{13}$C{$^1$H} NMR** (151 MHz, $CDCl_3$) δ 144.4, 134.1, 131.8, 131.2, 129.4, 128.6, 127.6, 126.2, 47.8, 25.7, 23.2. **IR** (ATR): ν = 2929, 2859, 2166, 1748, 1443, 1283, 1136, 1085, 957, 919, 756, 687. **MS** (EI, 70 eV): *m/z* (%) = 267.0 (6), 266.1 (10), 264.8 (74), 261.9 (20), 191.9 (8), 124.0 (64), 84.2 (100), 77.2 (24), 57.9 (17),47.4 (55). **HRMS** (ESI) *m/z:* $[M+H]^+$ Calcd for $C_{17}H_{21}O_2N_2S_2^+$ 349.1039; Found, 349.1032.

**4-Bromo-*N*-[phenyl(piperidin-1-yl)- λ⁴-sulfaneylidene] benzenesulfonamide (7j)**

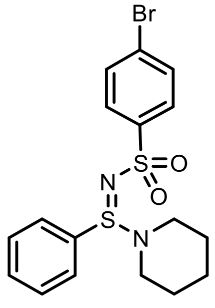


Following the general procedure afforded the product as a white solid (308.2 mg, 72% yield). **m.p.**: 145.0-147.7 °C. **$^1$H NMR** (600 MHz, $CDCl_3$) δ 7.82 – 7.78 (m, 2H), 7.71 – 7.66 (m, 2H), 7.57 – 7.52 (m, 2H), 7.52 – 7.46 (m, 3H), 3.15 – 3.06 (m, 2H), 2.88 – 2.78 (m, 2H), 1.66 – 1.52 (m, 4H), 1.51 – 1.44 (m, 2H). **$^{13}$C{$^1$H} NMR** (151 MHz, $CDCl_3$) δ 143.5, 133.7, 131.9, 131.7, 129.4, 127.8, 127.5, 125.7, 47.8, 25.6, 23.1. **IR** (ATR): ν = 2928, 2853, 1743, 1572, 1444, 1325, 1290, 1137, 1087, 962, 906, 753, 684. **MS** (EI, 70 eV): *m/z* (%) = 344.7 (14), 342.9 (17), 221.0 (9), 219.0 (9), 157.0 (19), 155.0 (18), 124.1 (70), 84.2 (100), 58.0 (16), 47.5 (32). **HRMS** (ESI) *m/z:* $[M+H]^+$ Calcd for $C_{17}H_{20}O_2N_2BrS_2^+$ 427.0144; Found, 427.0138.

***N*-[Phenyl(piperidin-1-yl)-λ⁴-sulfaneylidene]-4-(trifluoromethyl)benzenesulfon-amide (7k)**

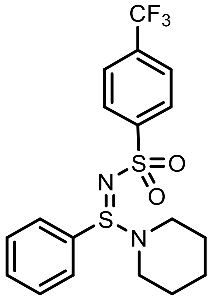


Following the general procedure afforded the product as a white solid (372.9 mg, 90% yield). **m.p.**: 128.6-130.1 °C. **$^1$H NMR** (600 MHz, $CDCl_3$) δ 8.05 (d, *J* = 8.2 Hz, 2H), 7.70 – 7.65 (m, 4H), 7.53 – 7.44 (m, 3H), 3.14 – 3.05 (m, 2H), 2.87 – 2.78 (m, 2H), 1.66 – 1.49 (m, 4H), 1.50 – 1.40 (m, 2H). **$^{13}$C{$^1$H} NMR** (151 MHz, $CDCl_3$) δ 147.9, 133.6, 132.9 (q, *J* = 32.8 Hz), 132.1, 129.6, 127.60, 126.7, 125.8 (q, *J* = 3.8 Hz), 123.5 (q, *J* = 272.6 Hz), 47.9, 25.7, 23.2. **$^{19}$F NMR** (565 MHz, $CDCl_3$) δ –62.92. **IR** (ATR): ν = 2947, 2856, 1447, 1402, 1316, 1152, 1122, 969, 912, 706. **MS** (EI, 70 eV): *m/z* (%) = 417.0 (15), 415.2 (6), 332.8 (100), 327.7 (31), 325.5 (10), 199.0 (13), 193.0 (15), 145.0 (25), 124.0 (68), 84.2 (98), 77.1 (10), 57.8 (28), 47.4 (74). **HRMS** (ESI) *m/z:* $[M+Na]^+$ Calcd for $C_{18}H_{19}O_2N_2F_3NaS_2^+$ 439.0732; Found, 439.0719.

**2,4-Dichloro-*N*-[phenyl(piperidin-1-yl)- λ⁴-sulfaneylidene] benzamide (7l)**

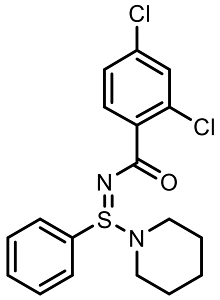

Following the general procedure afforded the product as a colorless oil (244.7 mg, 64% yield). **$^1$H NMR** (600 MHz, $CDCl_3$) δ 7.93 (dd, *J* = 6.7, 3.0 Hz, 2H), 7.76 (d, *J* = 8.3 Hz, 1H), 7.58 – 7.48 (m, 3H), 7.41 (d, *J* = 1.9 Hz, 1H), 7.25 (dd, *J* = 8.4, 2.0 Hz, 1H), 3.29 – 3.21 (m, 2H), 3.16 – 3.07 (m, 2H), 1.69 – 1.59 (m, *J* = 7.7 Hz, 4H), 1.57 – 1.49 (m, 2H). **$^{13}$C{$^1$H} NMR** (151 MHz, $CDCl_3$) δ 177.2, 136.5, 135.1, 134.1, 132.8, 131.5, 131.3, 130.0, 129.2, 128.1, 126.6, 49.1, 26.2, 23.5. **IR** (ATR): ν = 2938, 2853, 1583, 1444, 1304, 1140, 1096, 911, 831, 753, 686. **MS** (EI, 70 eV): *m/z* (%) = 380.9 (1), 298.9 (1), 172.9 (16), 109.0 (8), 84.1 (62), 58.8 (15), 57.8 (34), 54.5 (8), 47.3 (100). **HRMS** (ESI) *m/z:* $[M+H]^+$ Calcd for $C_{18}H_{19}ON_2Cl_2S^+$ 381.0590; Found, 381.0585.

***N*-[Ethyl(phenyl)-λ⁴-sulfaneylidene]-4-methylbenzenesulfonamide (8)**

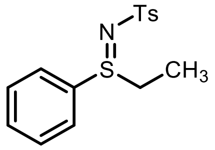

The procedure for the synthesis of sulfilimines using Grignard reagents (see section 2.6) was followed. The crude mixture was separated using automated silica gel column chromatography (hex/THF 7:3→3:7) to furnish product **8** as a yellow solid (57 mg, 68% yield). **TLC** $R_f$ 0.2 in hex/THF 5:5. **m.p.**: 96.5-97.5 °C. **$^1$H NMR** (400 MHz, $CDCl_3$) δ 7.74 (d, J = 8.3 Hz, 2H), 7.67 (d, J = 6.7 Hz, 1H), 7.55 – 7.44 (m, 3H), 7.15 (d, J = 8.0 Hz, 2H), 3.12 – 2.93 (m, 2H), 2.34 (s, 3H), 1.19 (t, J = 7.3 Hz, 3H). **$^{13}$C{$^1$H} NMR** (151 MHz, $CDCl_3$) δ 141.5, 141.3, 134.1, 132.3, 129.8, 129.1, 126.24, 126.20, 47.8, 21.3, 7.4. **HRMS** (ESI) *m/z:* $[M+H]^+$ Calcd for $C_{15}H_{18}NO_2S_2^+$ 308.0779; Found, 308.0773. The characterization is in agreement with the reported values.[S24]

***N*-[Ethyl(oxo)(phenyl)- λ⁶-sulfaneylidene]-4-methylbenzenesulfonamide (9)**

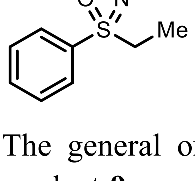

The general oxidation procedure was followed on a 0.08 mmol scale, to furnish product **9** as a colorless solid (23 mg, 90% yield). **TLC** Rf 0.25 in hex/THF 6:4. **$^1$H NMR** (600 MHz, $CDCl_3$) δ 7.96 (dd, J = 8.5, 1.3 Hz, 2H), 7.84 (d, J = 8.3 Hz, 2H),

7.69 (t, J = 7.5 Hz, 1H), 7.62 – 7.57 (m, 2H), 7.24 (d, J = 8.0 Hz, 2H), 3.59 – 3.46 (m, 2H), 2.38 (s, 3H), 1.25 (t, J = 7.4 Hz, 3H). **$^{13}$C{$^1$H} NMR** (151 MHz, $CDCl_3$) δ 142.8, 140.9, 135.9, 134.3, 129.6, 129.2, 128.5, 126.7, 52.9, 21.5, 7.5. **HRMS** (ESI) *m/z:* $[M+H]^+$ Calcd for $C_{15}H_{18}NO_3S_2^+$ 324.0723; Found, 324.0727. Characterization is in agreement with reported values.[S25]

**_N_-{[4-(_Tert_-butyl) phenyl] (phenyl)- λ$^4$-sulfaneylidene}-4-methylbenzenesulfonamide (10)**

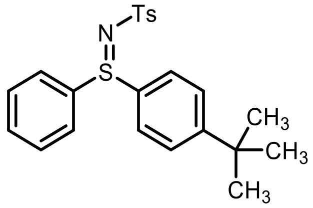


The procedure for the synthesis of sulfilimines using Grignard reagents (see section 2.6) was followed. The crude mixture was separated using automated silica gel column chromatography (hex/EtOAc 7:3) to furnish the product **10** as a colorless solid (86 mg, 84% yield). **TLC** Rf 0.45 in hex/EtOAc 5:5. **m.p.**: 128-130°C. **$^1$H NMR** (400 MHz, $CDCl_3$) δ 7.73 (d, J = 8.3 Hz, 2H), 7.64 – 7.60 (m, 2H), 7.53 – 7.41 (m, 7H), 7.12 (d, J = 7.6 Hz, 1H), 2.33 (s, 3H), 1.28 (s, 9H). **$^{13}$C{$^1$H} NMR** (101 MHz, $CDCl_3$) δ 156.2, 141.4, 141.49, 136.5, 133.2, 132.0, 129.7, 129.0, 127.2, 127.1, 126.9, 126.3, 35.1, 31.0, 21.4. **IR** (ATR): ν = 2960 (w, CH stretch), 1591 (w, C=C stretch), 1445 (w, C=C stretch), 1283 (s, O=S=O asymm. stretch), 1140 (vs, C-S stretch), 1089 (s, O=S=O symm. stretch), 963 (vs, N=S stretch). **HRMS** (ESI) *m/z:* $[M+H]^+$ Calcd for $C_{23}H_{26}NO_2S_2$ 412.1400; Found, 412.1404.

**2,2,2-Trifluoroethyl _N_-tosylbenzenesulfonimidate (11)**

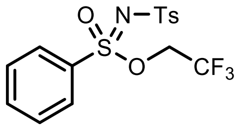


Following the general oxidation procedure afforded the product as a colorless oil (66.9 mg, 85% yield). **$^1$H NMR** (600 MHz, $CDCl_3$) δ 7.86 (d, *J* = 8.2 Hz, 2H), 7.79 (d, *J* = 8.0 Hz, 2H), 7.63 (t, *J* = 7.5 Hz, 1H), 7.48 (t, *J* = 7.8 Hz, 2H), 7.20 (d, *J* = 8.0 Hz, 2H), 4.62 – 4.53 (m, 1H), 4.52 – 4.43 (m, 1H), 2.32 (s, 3H). **$^{13}$C{$^1$H} NMR** (151 MHz, $CDCl_3$) δ 143.7, 139.3, 135.3, 134.5, 129.5, 129.4, 127.7, 126.8, 121.6 (q, *J* = 277.8 Hz), 65.2 (q, *J* = 38.4 Hz), 21.4. **$^{19}$F NMR** (565 MHz, $CDCl_3$) δ –73.48 (t, *J* = 7.9 Hz). **IR** (ATR): ν = 2975, 1448, 1277, 1160, 1123, 1010, 809. **MS** (EI, 70 eV): *m/z* (%) = 393.1 (10), 300.0 (100), 285.8 (24), 245.8 (17), 155.0 (27), 91.1 (13). **HRMS** (ESI) *m/z:* $[M+Na]^+$ Calcd for $C_{15}H_{14}O_3NF_3NaS_2^+$ 416.0209; Found, 416.0187.

**4-Methyl-*N*-(oxo(phenyl)(piperidin-1-yl)-λ$^6$-sulfaneylidene) benzenesulfonamide (12)**

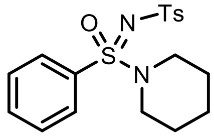


Following the general oxidation procedure afforded the product as a white solid (50.9 mg, 67% yield). **m.p.**: 121.3-122.7 °C. **$^1$H NMR** (600 MHz, $CDCl_3$) δ 7.91 (d, *J* = 7.9 Hz, 2H), 7.88 (d, *J* = 8.1 Hz, 2H), 7.65 – 7.60 (m,1H), 7.54 (t, *J* = 7.7 Hz, 2H), 7.30 – 7.25 (m, 2H), 3.27 – 3.19 (m, 2H), 3.13 – 3.05 (m, 2H), 2.41 (s, 3H), 1.72 – 1.58 (m, 4H), 1.54 – 1.44 (m, 2H). **$^{13}$C{$^1$H} NMR** (151 MHz, $CDCl_3$) δ 142.6, 140.9, 136.1, 133.4, 129.1, 129.1, 127.5, 126.8, 47.0, 25.0, 23.4, 21.5. **IR** (ATR): ν = 2935, 2857, 1444, 1308, 1153, 1086, 921, 736. **MS** (EI, 70 eV): *m/z* (%) = 301.0 (3), 155.1 (22), 84.2 (100), 47.3 (24). **HRMS** (ESI) *m/z:* $[M+Na]^+$ Calcd for $C_{18}H_{22}O_3N_2NaS_2^+$ 401.0964; Found, 401.0944.

**(*Z*)-*N*-[Amino(phenyl)- λ$^4$-sulfaneylidene]-4-methylbenzenesulfonamide (13)**

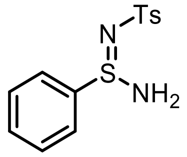


Following the procedure for the synthesis of $NH_2$-sulfinamidine **13** (see section 2.8). The product was recrystallized in *n*-pentane/EtOAc (1/1) to afford the product as a white solid (0.89 g, 61% yield). **TLC** $R_f$ 0.55 in hex/THF 4:6. **m.p.**: 140 °C (decomp.). **$^1$H NMR** (400 MHz, DMSO-$d_6$) δ 7.75 – 7.66 (m, 4H), 7.61 – 7.54 (m, 3H), 7.30 (d, J = 8.0 Hz, 2H), 6.93 (br, 2H), 2.35 (s, 3H). **$^{13}$C{$^1$H} NMR** (101 MHz, DMSO-$d_6$) δ 142.5, 141.1, 139.4, 131.5, 129.4, 129.3, 126.8, 125.8, 21.0. **IR** (ATR): ν = 3355 (w, $NH_2$ stretch), 3218 (w, CH stretch), 1596 (w, $NH_2$ sciss.), 1536 (w, C=C stretch), 1387 (w, C=C stretch), 1271 (s, O=S=O asymm. stretch), 1133 (s, C-S stretch), 1083 (s, O=S=O symm. stretch), 985 (vs, N=S stretch). **HRMS** (ESI) *m/z:* $[M+H]^+$ Calcd for $C_{13}H_{15}N_2O_2S_2^+$ 295.0569; Found, 295.0584.

***N*-[Imino(phenyl)(piperidin-1-yl)-λ$^6$-sulfaneylidene]-4-methylbenzenesulfon-amide (14)**

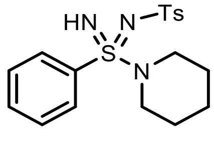


Following the procedure for the synthesis of sulfondiimidamide **14** (see section 2.9) afforded the product as a colorless oil (63.0 mg, 84% yield). **$^1$H NMR** (400 MHz, $CDCl_3$) δ 7.93 – 7.88 (m, 2H), 7.81 – 7.75 (m, 2H), 7.58 – 7.49 (m, 1H), 7.48 – 7.41 (m, 2H), 7.16 (d, *J* = 8.0 Hz, 2H), 3.12 – 2.96 (m, 4H), 2.33 (s, 3H), 1.60 – 1.48 (m, 4H), 1.45 – 1.33 (m, 2H). **$^{13}$C{$^1$H} NMR** (101 MHz, $CDCl_3$) δ 142.1, 141.1, 135.5, 132.8, 129.0, 128.8, 127.1, 126.5, 47.2, 25.2, 23.5, 21.3. **IR** (ATR): ν = 2939, 2854,

1601, 1446, 1285, 1050, 908, 754. **MS** (EI, 70 eV): *m/z* (%) = 378.2 (6), 279.1 (38), 218.2 (10) 155.1 (23), 124.1 (67), 91.1 (43), 84.2 (100), 65.2 (18), 56.2 (18). **HRMS** (ESI) *m/z:* [M+H]$^+$ Calcd for $C_{18}H_{24}N_3O_2S_2^+$ 378.1304; Found, 378.1306.

**2,2,2-Trichloroethyl-*N*-tosylpiperidine-1-sulfinimidate (15)**

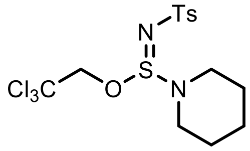


Following the procedure for the synthesis of imidosulfamite (see section 2.10) afforded the product as a colorless oil (29.0 mg, 67% yield). **$^1$H NMR** (600 MHz, $CDCl_3$) δ 7.79 (d, *J* = 8.3 Hz, 2H), 7.26 (d, *J* = 8.1 Hz, 2H), 4.42 (d, *J* = 12.0 Hz, 1H), 4.22 (d, *J* = 12.1 Hz, 1H), 3.52 – 3.43 (m, 2H), 3.30 – 3.20 (m, 2H), 2.39 (s, 3H), 1.66 – 1.55 (m, 6H). **$^{13}$C{$^1$H} NMR** (151 MHz, $CDCl_3$) δ 142.5, 140.6, 129.4, 126.2, 95.3, 75.7, 45.5, 25.2, 23.9, 21.5. **IR** (ATR): ν = 2939, 2858, 1448, 1301, 1149, 990, 816, 723. **MS** (EI, 70 eV): *m/z* (%) = 171.3 (6), 131.3 (16), 91.3 (81) 84.3 (100), 65.4 (45), 55.3 (89), 49.3 (12). **HRMS** (ESI) *m/z:* [M+Na]$^+$ Calcd for $C_{14}H_{19}O_3N_2Cl_3NaS_2^+$ 454.9795; Found, 454.9798.

## 4. References

[S1] L. De Luca, G. Giacomelli and G. Nieddu. *Synlett,* 2005, 223–226.

[S2] J. Wang, M. Frings and C. Bolm, *Angew. Chem. Int. Ed.,* 2013, **52**, 8661–8665.

[S3] M. Andresini, M. Spennacchio, M. Colella, G. Losito, A. Aramini, L. Degennaro and R. Luisi, *Org. Lett.*, 2021, **23**, 6850–6854.

[S4] J. L. García Ruano, A. Parra, L. Marzo, F. Yuste and V. M. Mastranzo, *Tetrahedron,* 2011, **67**, 2905–2910.

[S5] J. L. García Ruano, J. Alemán, M. Belén Cid and A. Parra, *Org. Lett.,* 2005, **7**, 179–182.

[S6] Z.-X. Zhang, Charles. Bell, Ming, Ding and M. C. Willis, *J. Am. Chem. Soc.,* 2022, **144**, 11851−11858.

[S7] (*a*) P. Pracht, F. Bohle and S. Grimme, *Phys. Chem. Chem. Phys.,* 2020, **22**, 7169–7192; (*b*) S. Grimme, *J. Chem. Theory Comput.*, 2019, **15**, 2847–2862.

[S8] (*a*) C. Bannwarth, S. Ehlert and S. Grimme, *J. Chem. Theory Comput.,* 2019, **15**, 1652–1671; (*b*) C. Bannwarth, E. Caldeweyher, S. Ehlert, A. Hansen, P. Pracht, J. Seibert, S. Spicher and S. Grimme, *WIREs Comput. Mol. Sci.,* 2020, **11**, e1493.

[S9] (a) E. Caldeweyher, C. Bannwarth and S. Grimme, *J. Chem. Phys.*, 2017, **147**, 034112; (b) E. Caldeweyher, S. Ehlert, A. Hansen, H. Neugebauer, S. Spicher, C. Bannwarth and S. Grimme, *J. Chem. Phys.*, 2019, **150**, 154122. (c) E. Caldeweyher, J.-M. Mewes, S. Ehlert and S. Grimme, *Phys. Chem. Chem. Phys.*, 2020, **22**, 8499–8512.

[S10] S. Ehlert, M. Stahn, S. Spicher and S. Grimme, *J. Chem. Theory Comput.,* 2021, **17**, 4250–4261.

[S11] (*a*) H. Kruse, S. Grimme, *J. Chem. Phys.*, 2012, **136**, 154101; (*b*) J. W. Furness, A. D. Kaplan, J. Ning, J. P. Perdew and J. Sun, *J. Phys. Chem. Lett.*, 2020, **11**, 8208–8215; (*c*) S. Grimme, A. Hansen, S. Ehlert and J.-M. Mewes, *J. Chem. Phys.,* 2021, **154**, 064103.

[S12] (*a*) F. Neese, *WIREs Comput. Mol. Sci.,* 2012, **2**, 73–78; (*b*) F. Neese, F. Wennmohs, U. Becker and C. Riplinger, *J. Chem. Phys.,* 2020, **152,** 224108; (*c*) F. Neese, *WIREs Comput. Mol. Sci.*, 2022, **12**, e1606.

[S13] A. V. Marenich, C. J. Cramer and D. G. Truhlar, *J. Phys. Chem. B,* 2009, **113**, 6378–6396.

[S14] S. Grimme, F. Bohle, A. Hansen, P. Pracht, S. Spicher, and M. Stahn, *J. Phys. Chem. A*, 2021, **125**, 4039–4054.

[S15] F. Neese, *J. Comput. Chem.*, 2003, **24**, 1740–1747.

[S16] S. Spicher and S. Grimme, *J. Chem. Theory Comput.*, 2021, **17**, 1701–1714.

[S17] A. D. Becke, *J. Chem. Phys.*, 1993, **98**, 5648–5652.

[S18] (*a*) R. Ditchfield, W. J. Hehre and J. A. Pople, *J. Chem. Phys.,* 1971, **54**, 724–728; (*b*) W. J. Hehre, R. Ditchfield and J. A. Pople, *J. Chem. Phys.,* 1972, **56**, 2257–2261; (*c*) P. C. Hariharan and J. A. Pople, *Theor. Chem. Acc.,* 1973, **28**, 213–222; (*d*) P. C. Hariharan and J. A. Pople, *Mol. Phys.,* 1974, **27**, 209–214; (*e*) M. S. Gordon, *Chem. Phys. Lett.,* 1980, **76**, 163–168; (*f*) M. M. Francl, W. J. Pietro, W. J. Hehre, J. S. Binkley, D. J. DeFrees, J. A. Pople and M. S. Gordon, *J. Chem. Phys.,* 1982, **77**, 3654–3665; (*g*) T. Clark, J. Chandrasekhar, G. W. Spitznagel and P. von Ragué Schleyer, *J. Comput. Chem.,* 1983, **4**, 294–301; (*h*) R. C. Binning, Jr. and L. A. Curtiss, *J. Comput. Chem.,* 1990, **11**, 1206–1216; (*i*) J.-P. Blaudeau, M. P. McGrath, L. A. Curtiss and L. Radom, *J. Chem. Phys.,* 1997, **107**, 5016–5021; (*j*) V. A. Rassolov, J. A. Pople, M. A. Ratner and T. L. Windus, *J. Chem. Phys.,* 1998, **109**, 1223–1229; (*k*) V. A. Rassolov, M. A. Ratner, J. A. Pople, P. C. Redfern and L. A. Curtiss, *J. Comput. Chem.,* 2001, **22**, 976–984.

[S19] Gaussian 09, Revision E.01, M. J. Frisch, G. W. Trucks, H. B. Schlegel, G. E. Scuseria, M. A. Robb, J. R. Cheeseman, G. Scalmani, V. Barone, B. Mennucci, G. A. Petersson, H. Nakatsuji, M. Caricato, X. Li, H. P. Hratchian, A. F. Izmaylov, J. Bloino, G. Zheng, J. L. Sonnenberg, M. Hada, M. Ehara, K. Toyota, R. Fukuda, J. Hasegawa, M. Ishida, T. Nakajima, Y. Honda, O. Kitao, H. Nakai, T. Vreven, J. A. Montgomery, Jr., J. E. Peralta, F. Ogliaro, M. Bearpark, J. J. Heyd, E. Brothers, K. N. Kudin, V. N. Staroverov, T. Keith, R. Kobayashi, J. Normand, K. Raghavachari, A. Rendell, J. C. Burant, S. S. Iyengar, J. Tomasi, M. Cossi, N. Rega, J. M. Millam, M. Klene, J. E. Knox, J. B. Cross, V. Bakken, C. Adamo, J. Jaramillo, R. Gomperts, R. E. Stratmann, O. Yazyev, A. J. Austin, R. Cammi, C. Pomelli, J. W. Ochterski, R. L. Martin, K. Morokuma, V. G. Zakrzewski, G. A. Voth, P. Salvador, J. J. Dannenberg, S. Dapprich, A. D. Daniels, O. Farkas, J. B. Foresman, J. V. Ortiz, J. Cioslowski, and D. J. Fox, Gaussian, Inc., Wallingford CT, 2013.

[S20] (*a*) F. London, *J. Phys. Radium*, 1937, **8**, 397–409; (*b*) R. McWeeny, *Phys. Rev.*, 1962, **126**, 1028–1034; (*c*) R. Ditchfield, *Mol. Phys.*, 1974, **27**, 789–807; (*d*) K. Wolinski, J. F. Hinton and P. Pulay, *J. Am. Chem. Soc.*, 1990, **112**, 8251–8260; (*e*) J. Gauss, *Chem. Phys. Lett.*, 1992, **191**, 614–620; (*f*) J. Gauss, *J. Chem. Phys.*, 1993,

**99**, 3629–3643; (*g*) J. Gauss, *Ber. Bunsen-Ges.*, 1995, **99**, 1001–1008; (*h*) J. R. Cheeseman, G. W. Trucks, T. A. Keith and M. J. Frisch, *J. Chem. Phys.*, 1996, **104**, 5497–5509.

[S21] C. Adamo and V. Barone, *J. Chem. Phys.,* 1998, **108**, 664–675.

[S22] (*a*) A. D. McLean and G. S. Chandler, *J. Chem. Phys.,* 1980, **72**, 5639–5648; (*b*) R. Krishnan, J. S. Binkley, R. Seeger and J. A. Pople, *J. Chem. Phys.,* 1980, **72**, 650–654; (*c*) A. J. H. Wachters, *J. Chem. Phys.,* 1970, **52**, 1033–1036; (*d*) P. J. Hay, *J. Chem. Phys.,* 1977, **66**, 4377–4384; (*e*) M. J. Frisch, J. A. Pople and J. S. Binkley, *J. Chem. Phys.,* 1984, **80**, 3265–3269, (*f*) K. Raghavachari and G. W. Trucks, *J. Chem. Phys.,* 1989, **91**, 1062–1065; (*g*) M. P. McGrath and L. Radom, *J. Chem. Phys.,* 1991, **94**, 511–516; (*h*) L. A. Curtiss, M. P. McGrath, J.-P. Blaudeau, N. E. Davis, R. C. Binning, Jr. and L. Radom, *J. Chem. Phys.,* 1995, **103**, 6104–6113.

[S23] M. W. Lodewyk, M. R. Siebert and D. J. Tantillo, *Chem. Rev.,* 2012, **112**, 1839–1862.

[S24] M. Murakami, T. Uchida, B. Saito and T. Katsuki, *Chirality,* 2003, **15**, 116–123.

[S25] W. Zhang and J. Hu, *Adv. Synth. Catal.*, 2010, **352**, 2799–2804.

## 5. NMR Spectra

$^{1}$H NMR spectrum of compound **1d** (600 MHz, $CDCl_3$)

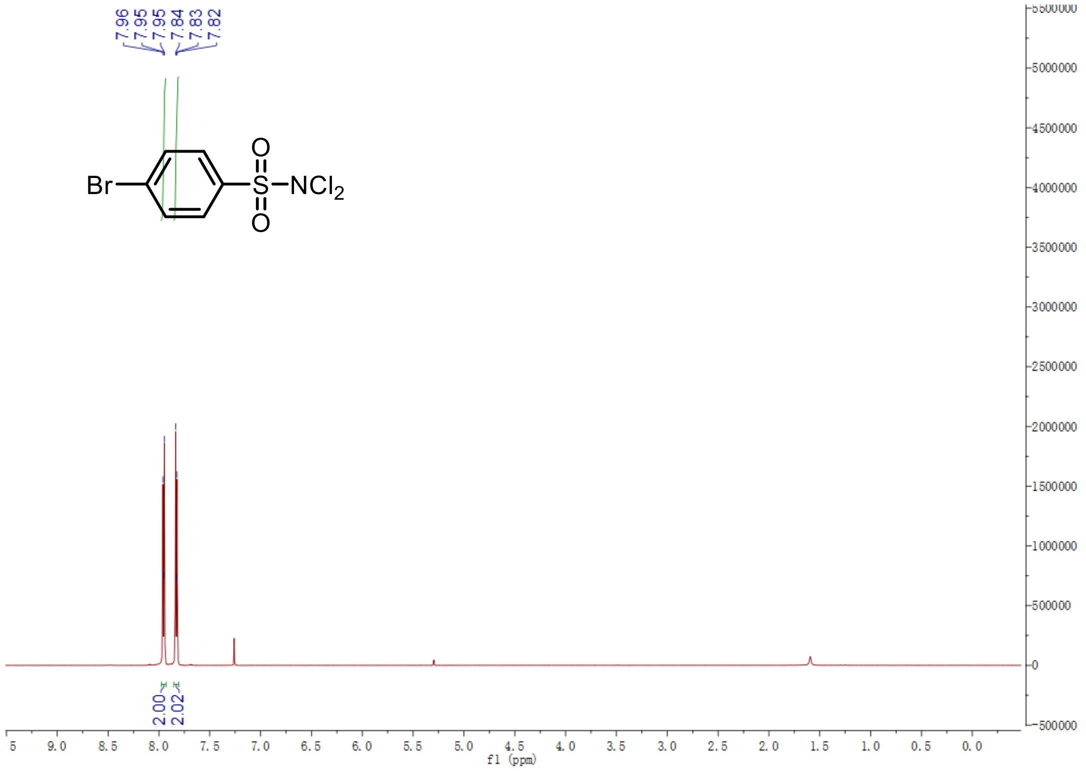


$^{13}$C{$^{1}$H} NMR spectrum of compound **1d** (151 MHz, $CDCl_3$)

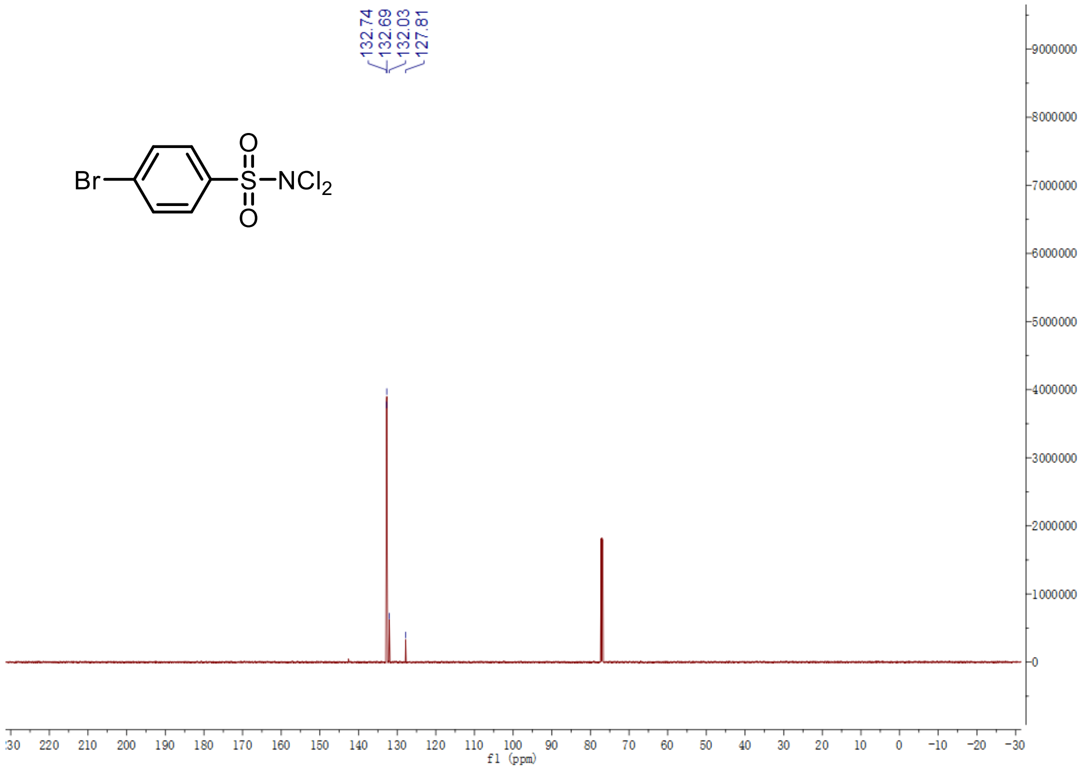

$^{1}H$ NMR spectrum of compound **1e** (600 MHz, $CDCl_3$)

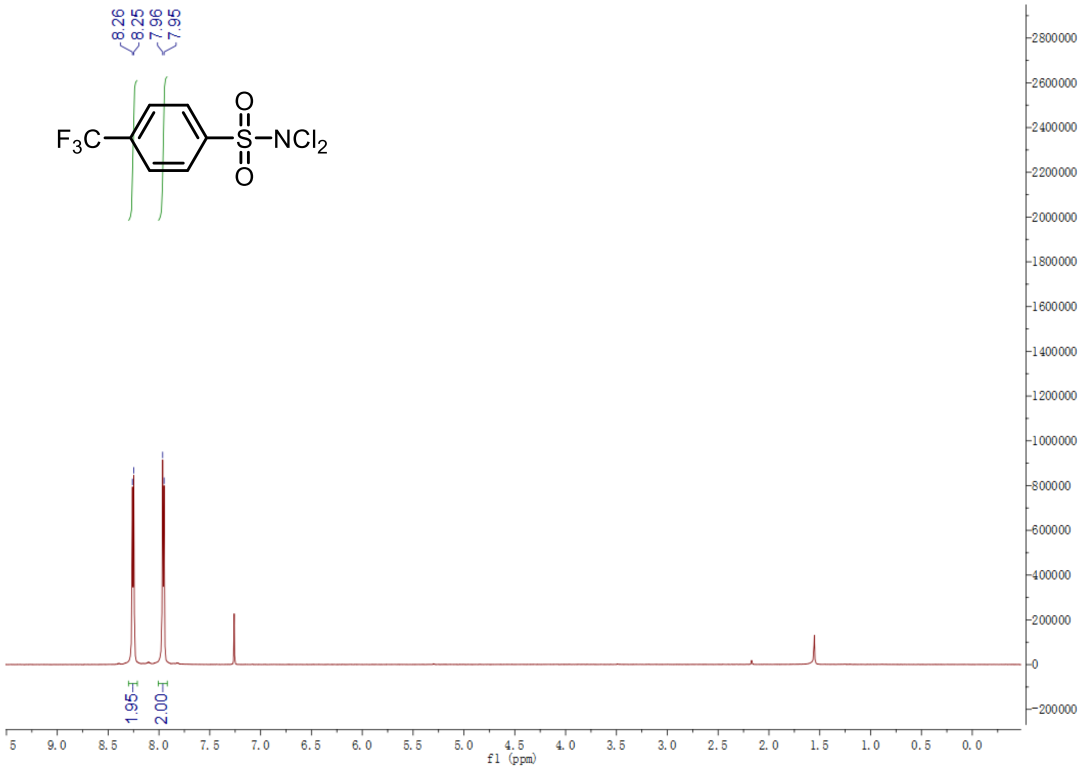


$^{13}C\{^{1}H\}$ NMR spectrum of compound **1e** (151 MHz, $CDCl_3$)

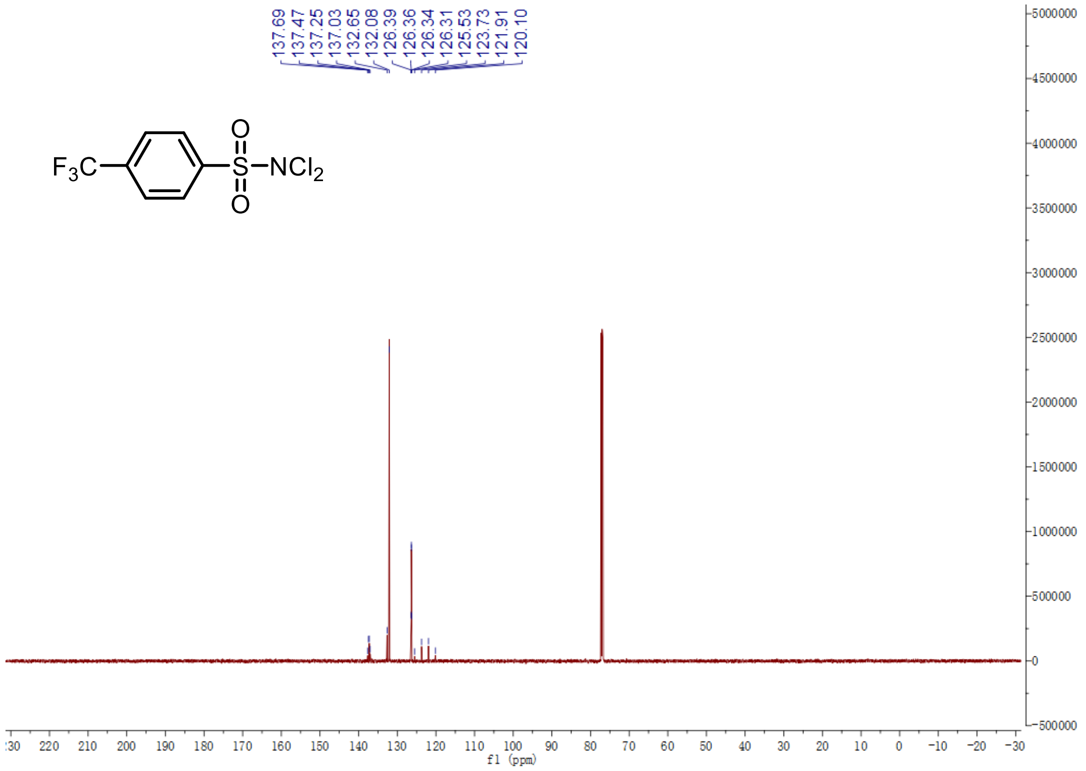

$^{19}$F NMR spectrum of compound **1e** (564 MHz, $CDCl_3$)

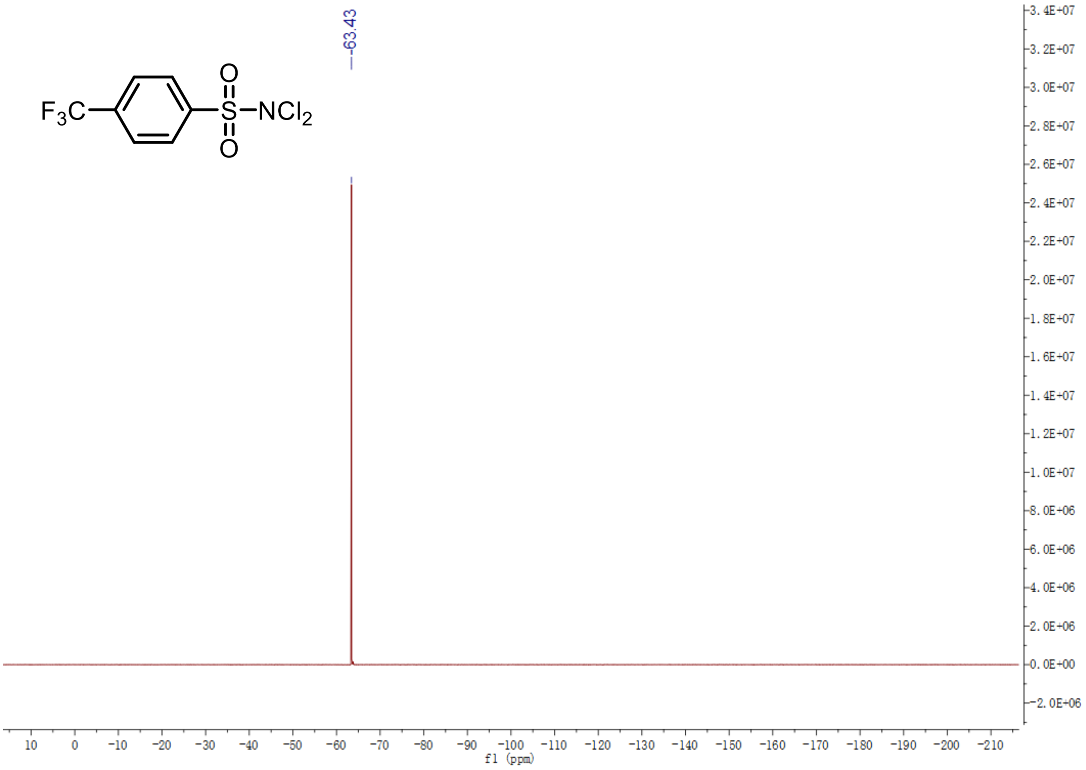


$^{1}$H NMR spectrum of compound **1h** (600 MHz, $CDCl_3$)

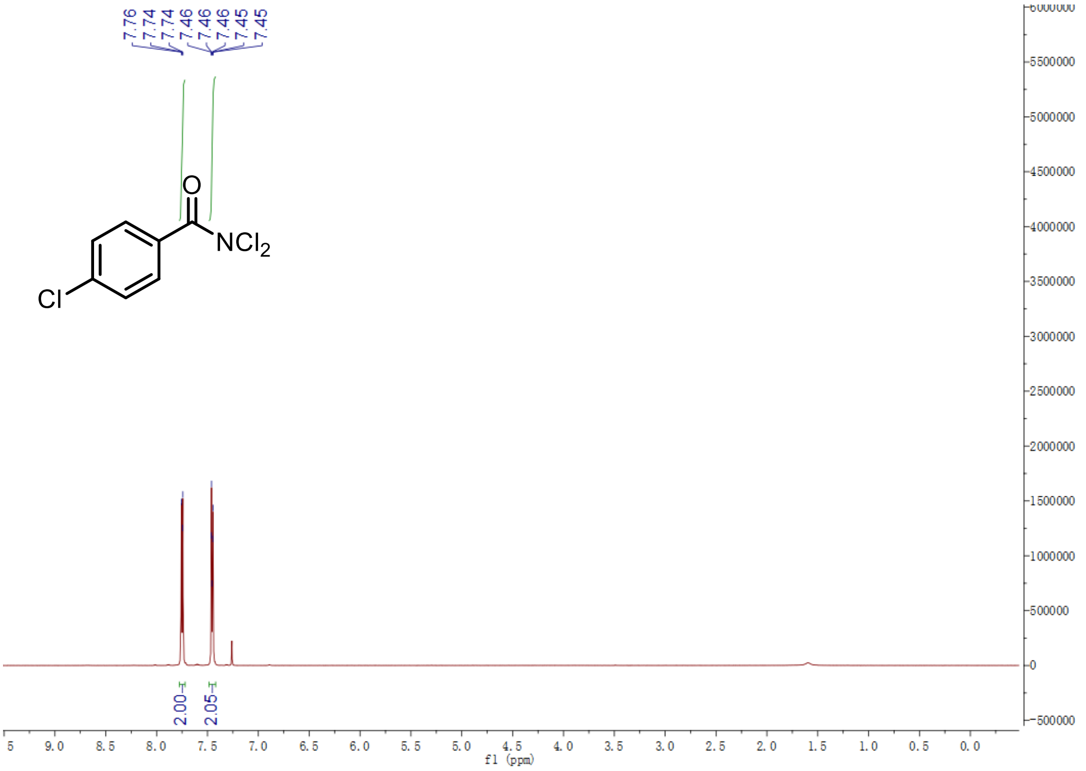

$^{13}C\{^1H\}$ NMR spectrum of compound **1h** (151 MHz, $CDCl_3$)

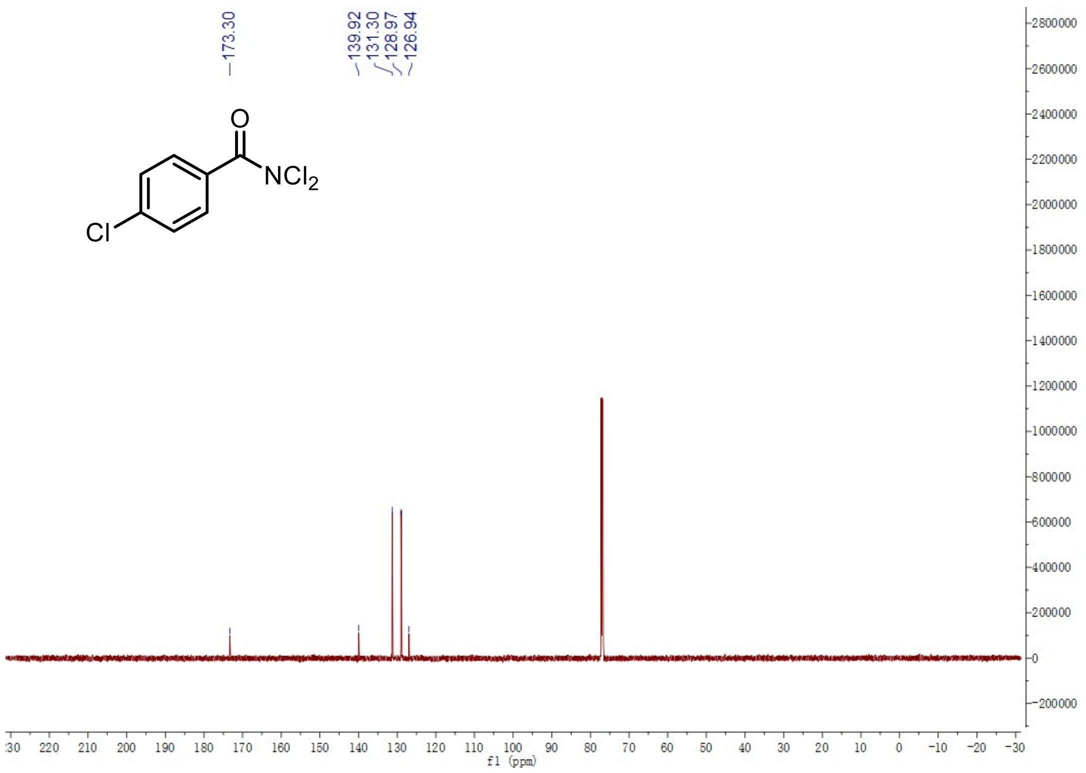


$^1H$ NMR spectrum of compound **1i** (600 MHz, $CDCl_3$)

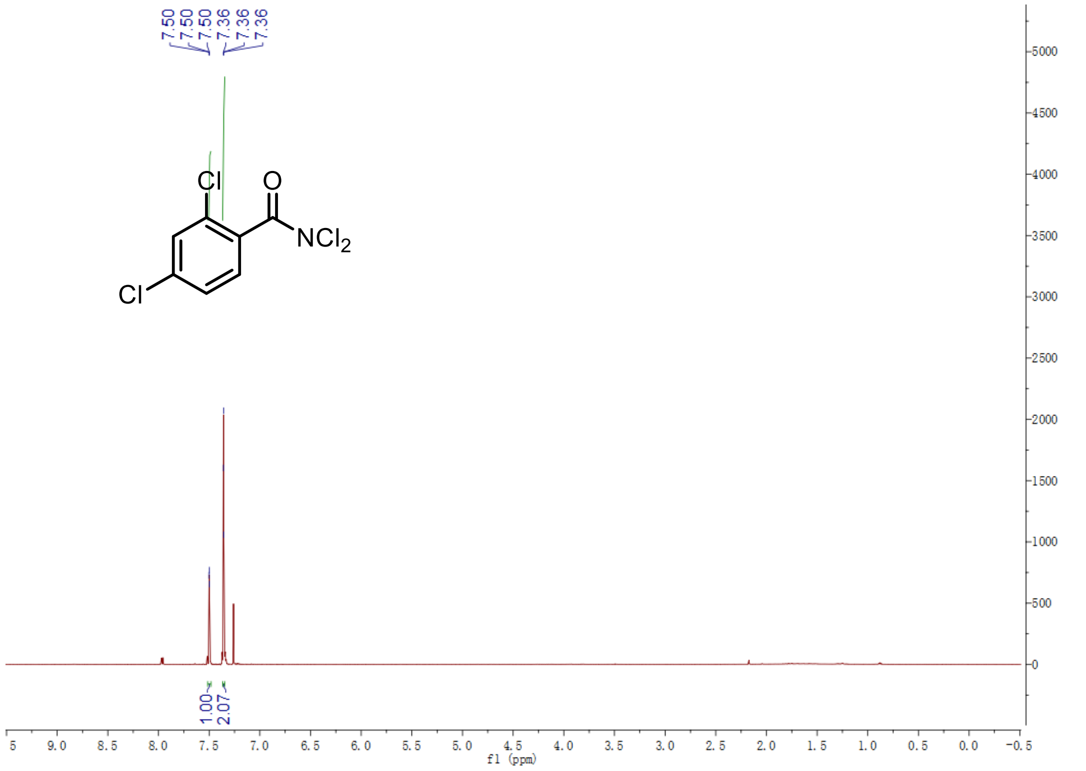

$^{13}C\{^1H\}$ NMR spectrum of compound **1i** (151 MHz, $CDCl_3$)

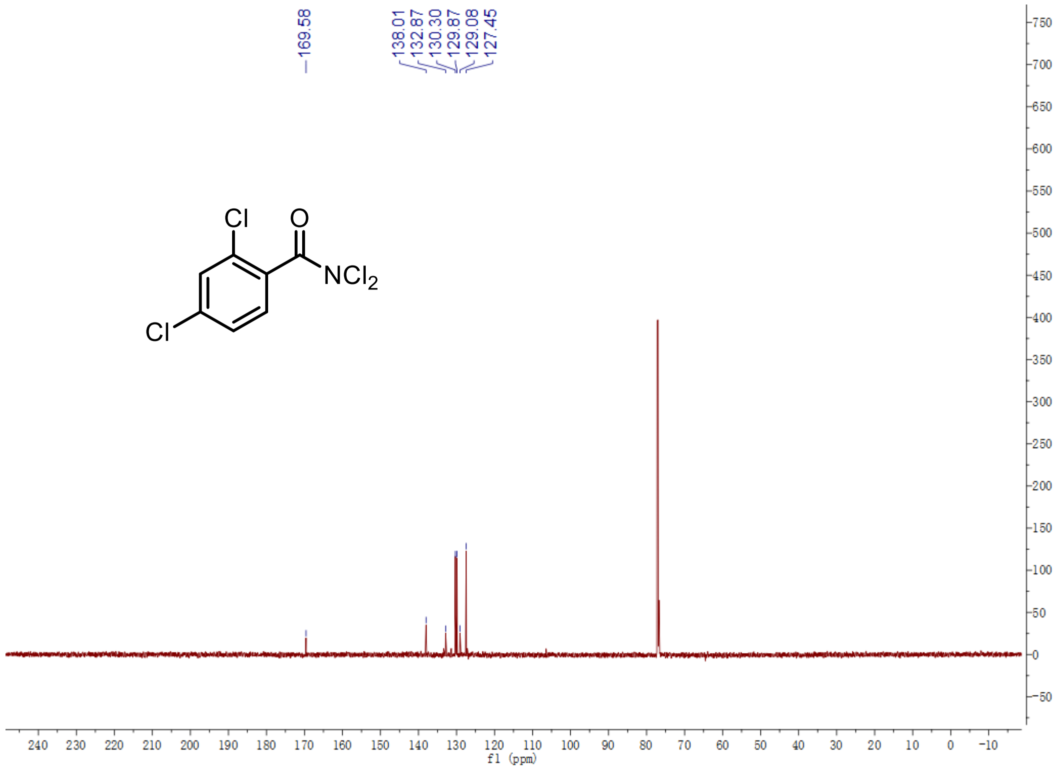

$^1H$ NMR spectrum of compound **3a** (600 MHz, $CDCl_3$)

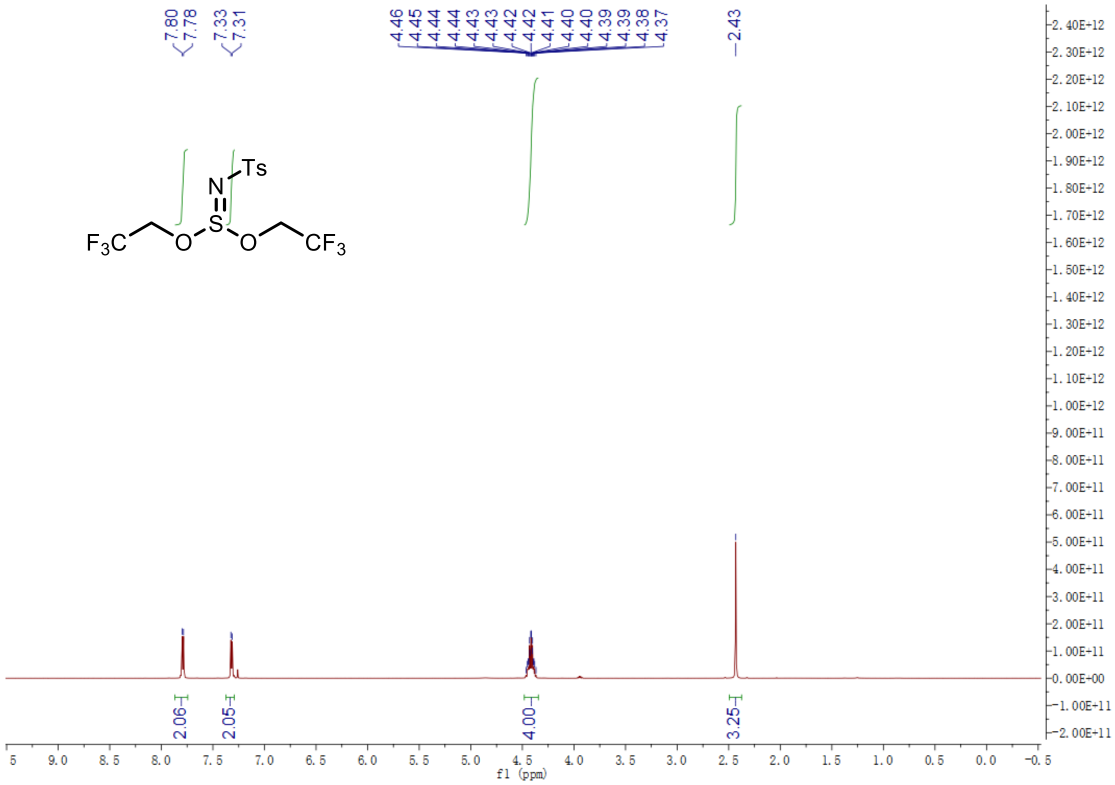

$^{13}C\{^{1}H\}$ NMR spectrum of compound **3a** (151 MHz, $CDCl_3$)

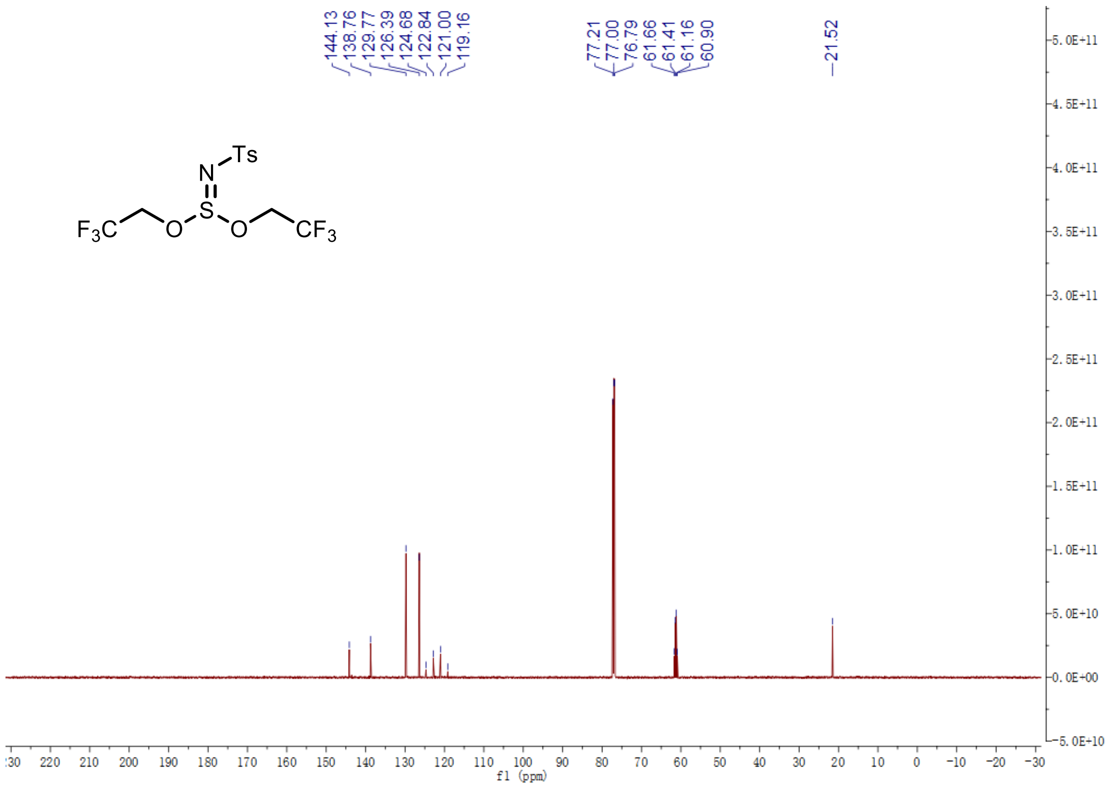


$^{19}F$ NMR spectrum of compound **3a** (564 MHz, $CDCl_3$)

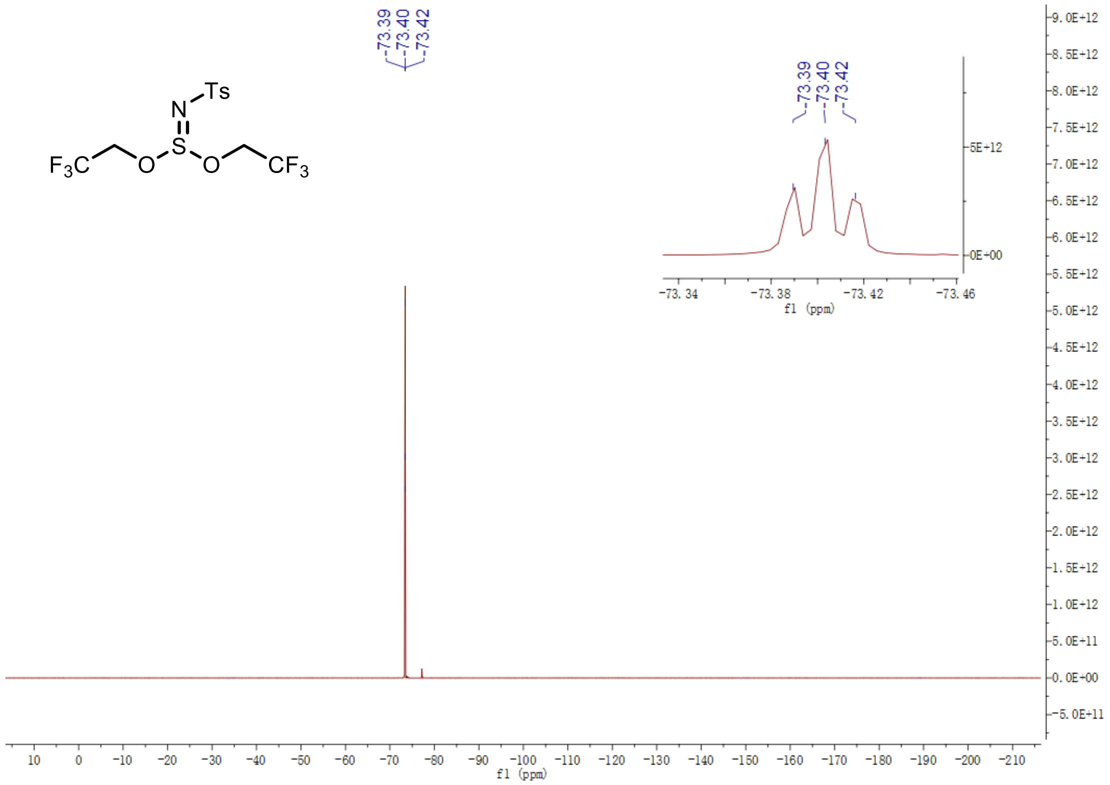

$^{1}H$ NMR spectrum of compound **3b** (600 MHz, $CDCl_3$)

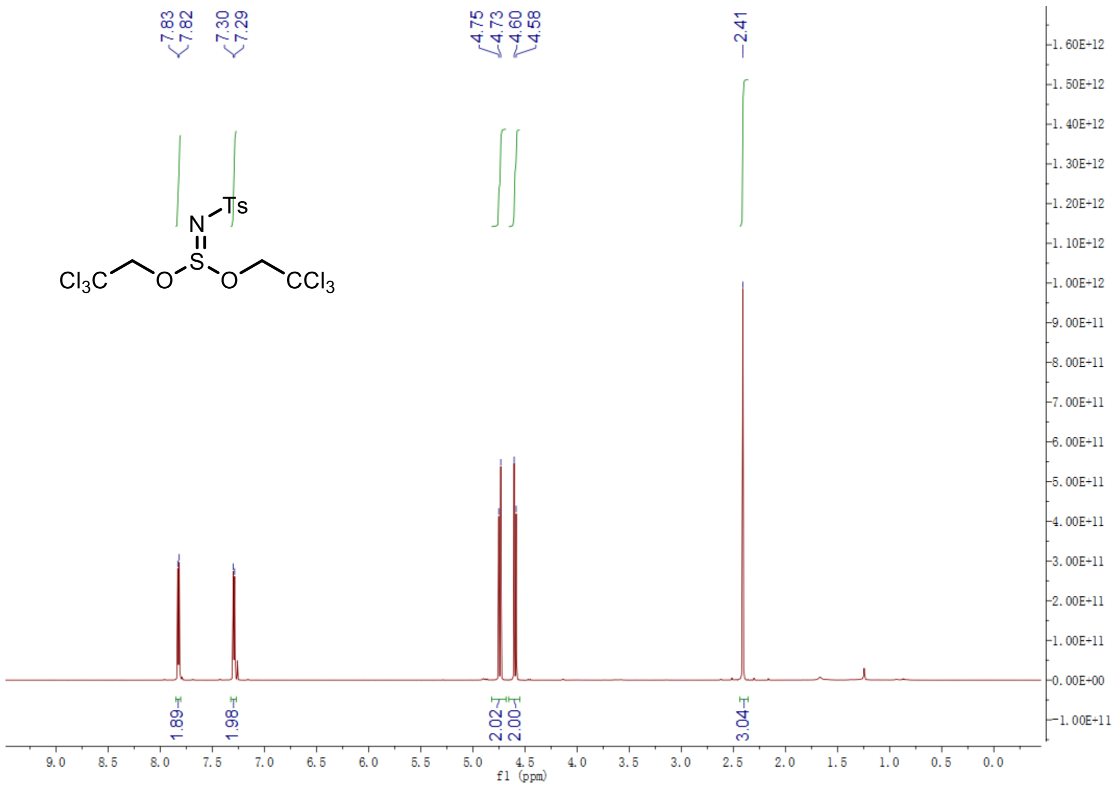


$^{13}C\{^{1}H\}$ NMR spectrum of compound **3b** (151 MHz, $CDCl_3$)

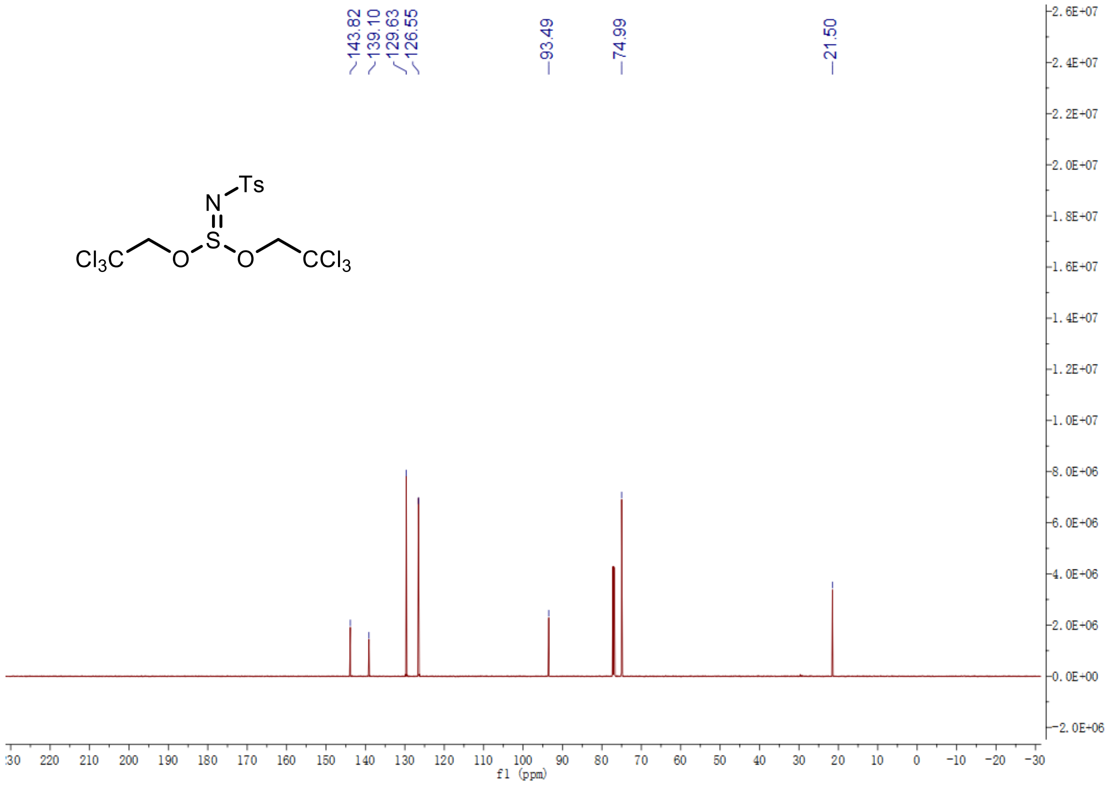

$^{1}$H NMR spectrum of compound **3c** (600 MHz, $CDCl_3$)

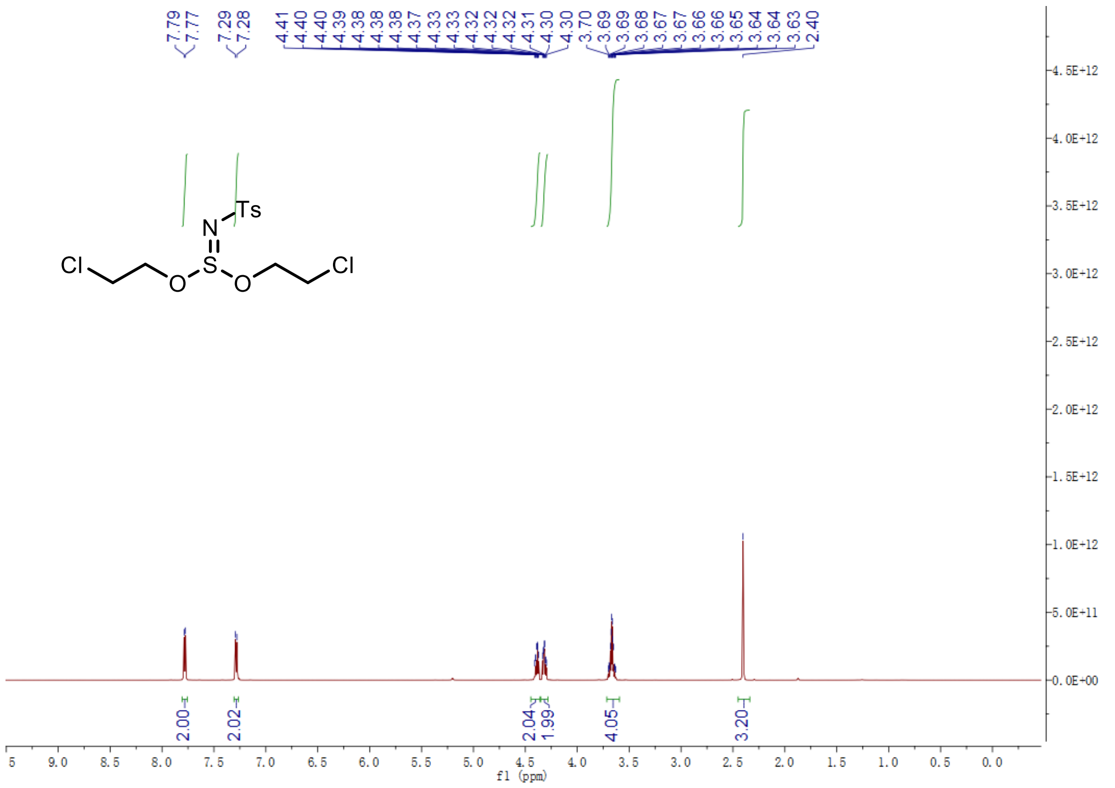

$^{13}$C{$^{1}$H} NMR spectrum of compound **3c** (151 MHz, $CDCl_3$)

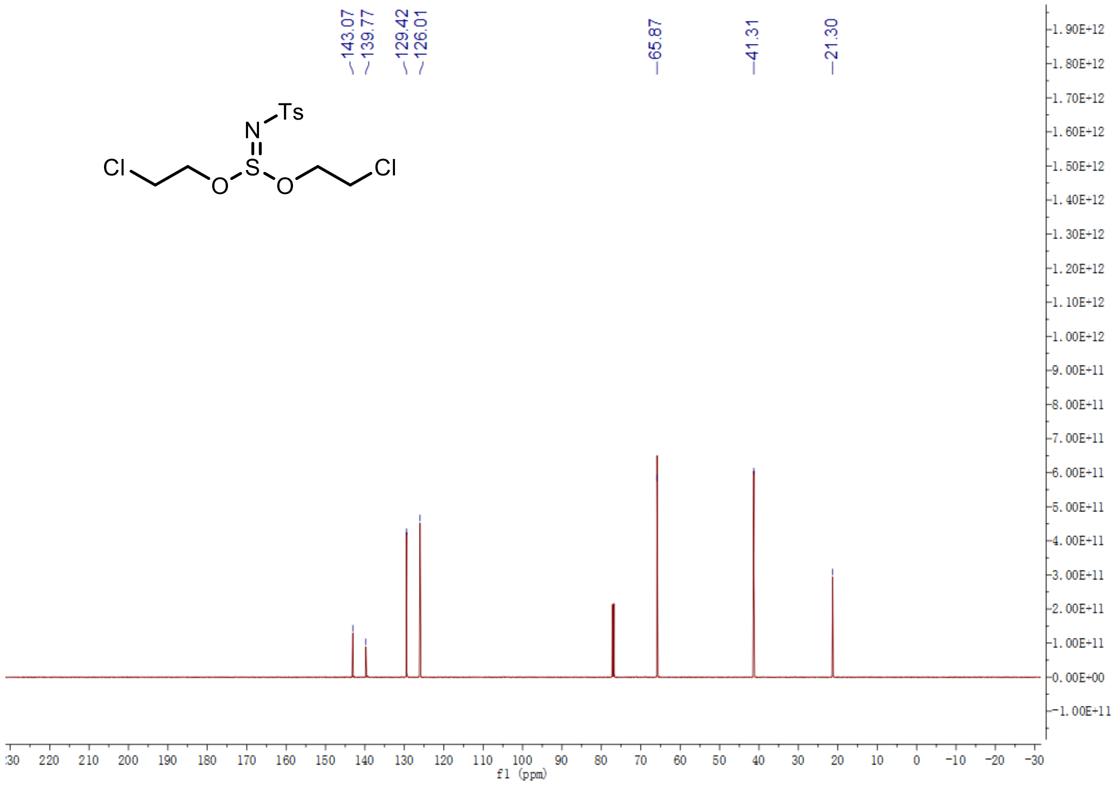

$^{1}$H NMR spectrum of compound **3d** (600 MHz, $CDCl_3$)

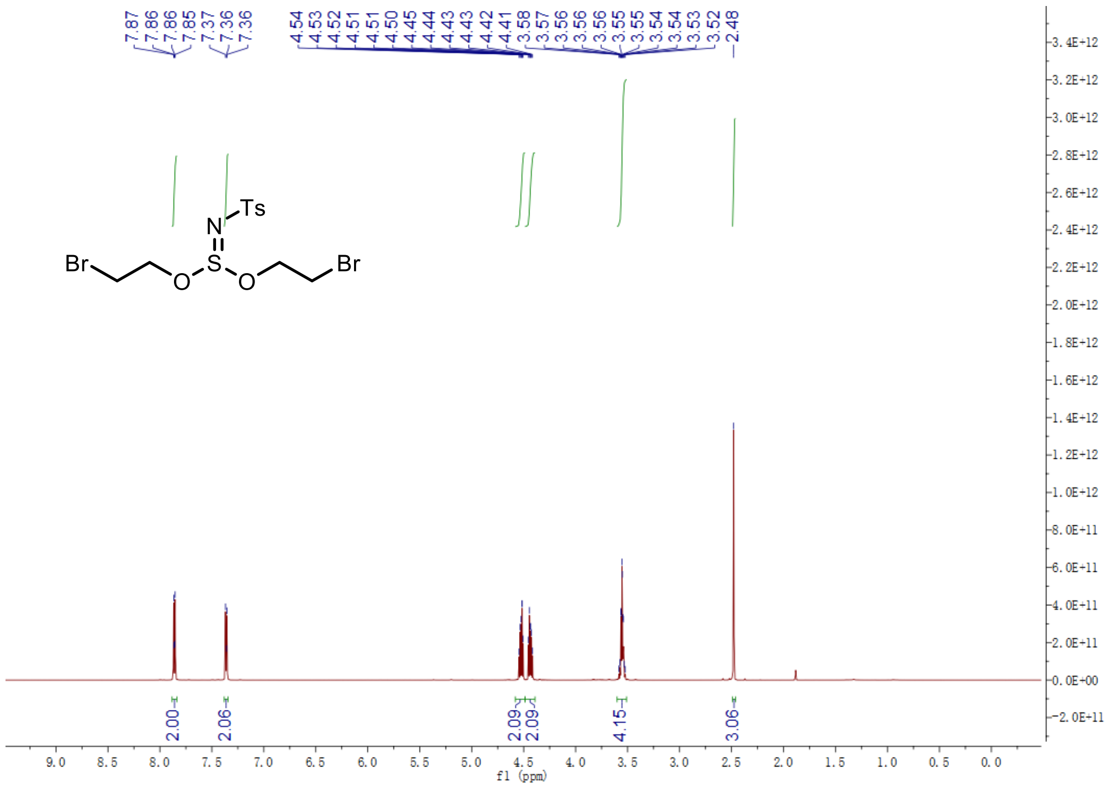

$^{13}$C{$^{1}$H} NMR spectrum of compound **3d** (151 MHz, $CDCl_3$)

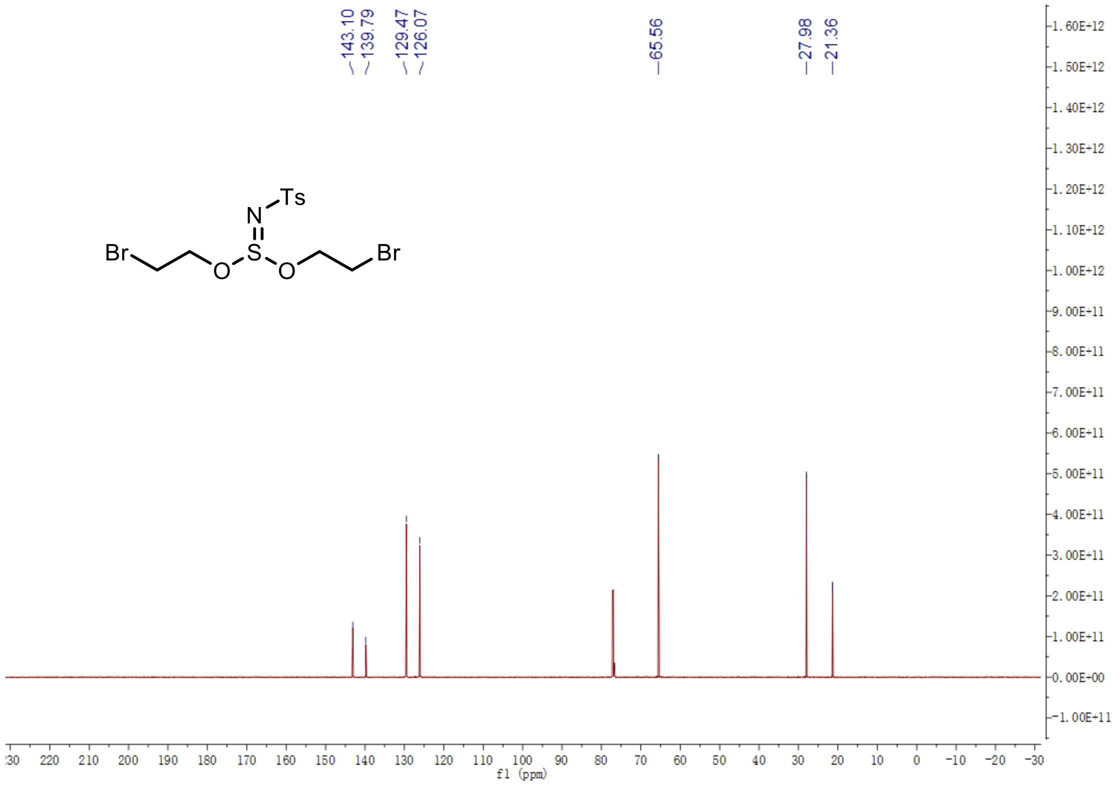

$^{1}$H NMR spectrum of compound **3e** (600 MHz, $CDCl_3$)

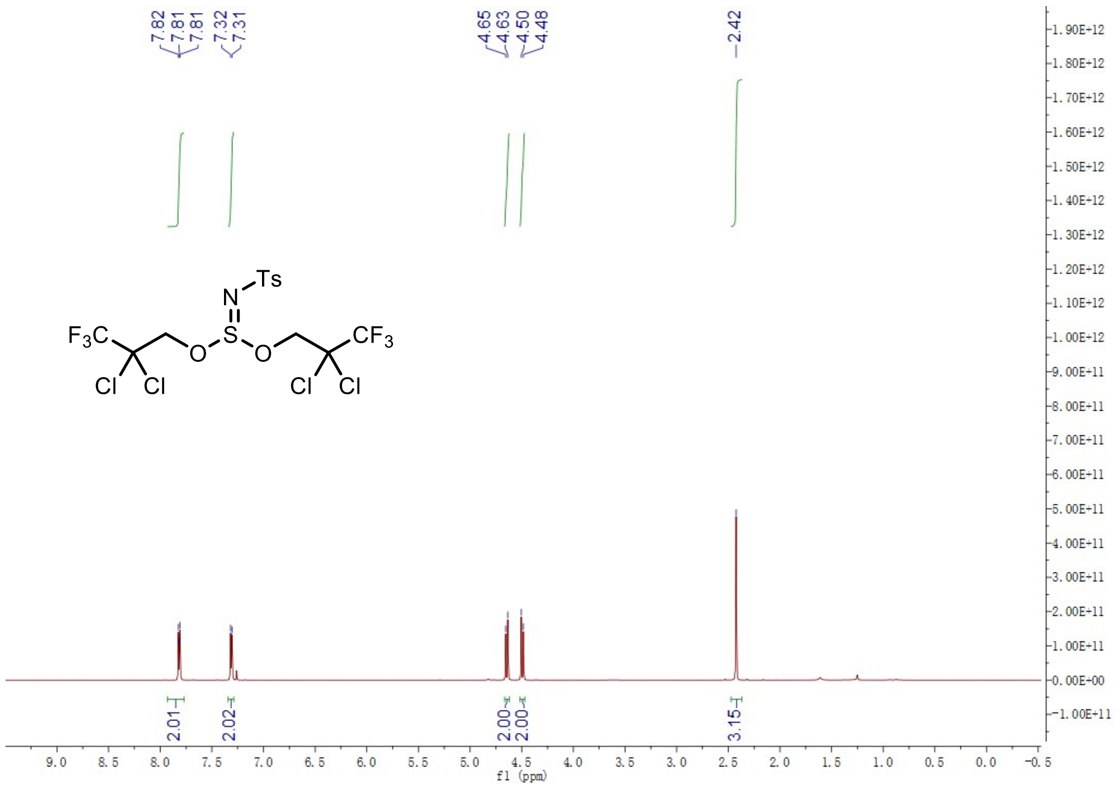


$^{13}$C{$^{1}$H} NMR spectrum of compound **3e** (151 MHz, $CDCl_3$)

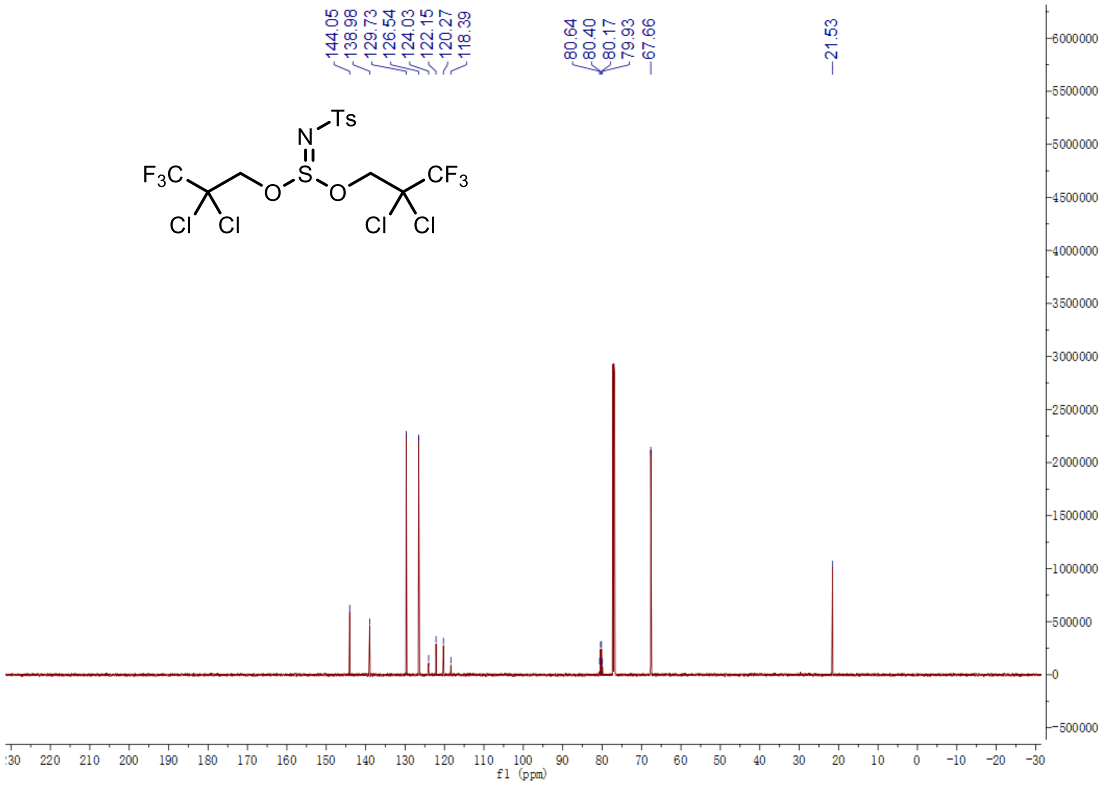

$^{19}$F NMR spectrum of compound **3e** (564 MHz, $CDCl_3$)

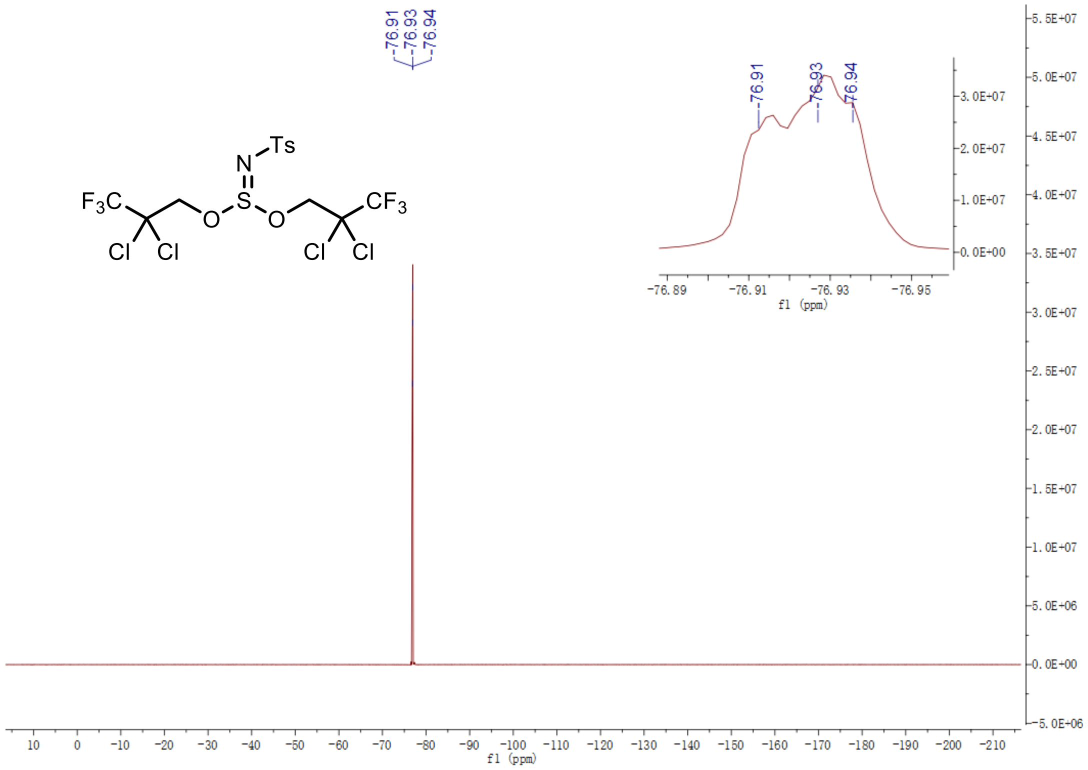


$^{1}$H NMR spectrum of compound **3f** (600 MHz, $CDCl_3$)

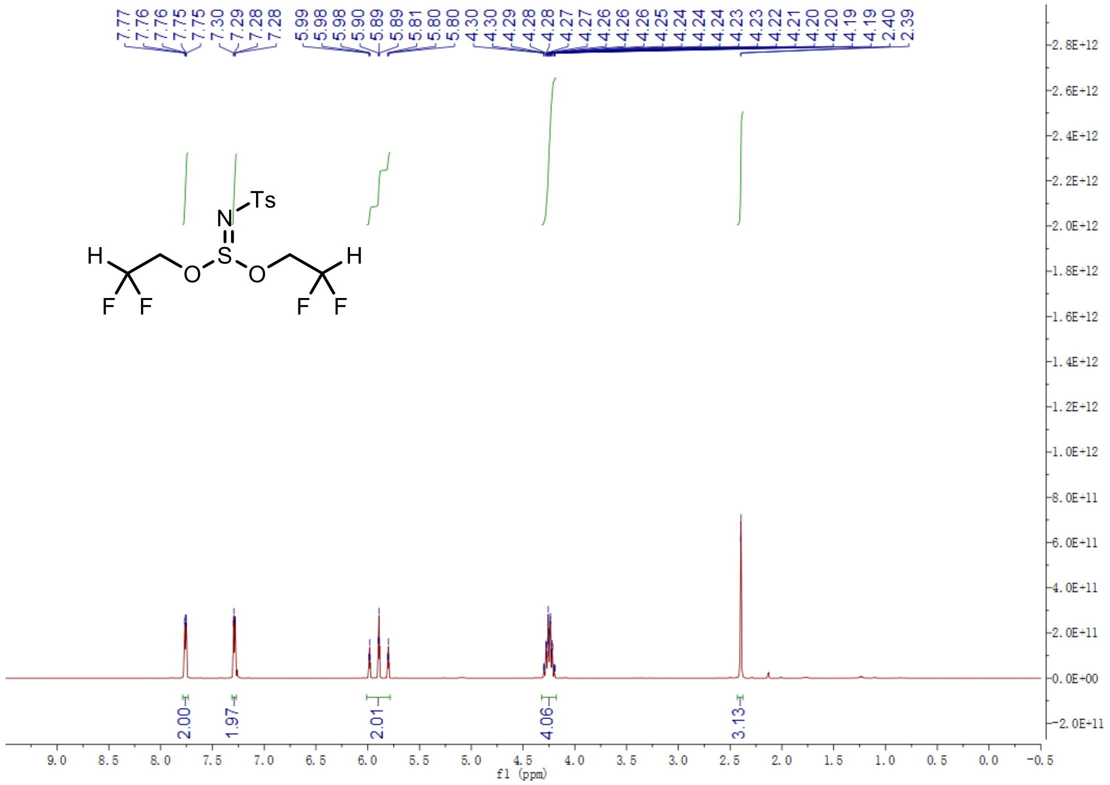

$^{13}C\{^{1}H\}$ NMR spectrum of compound **3f** (151 MHz, $CDCl_3$)

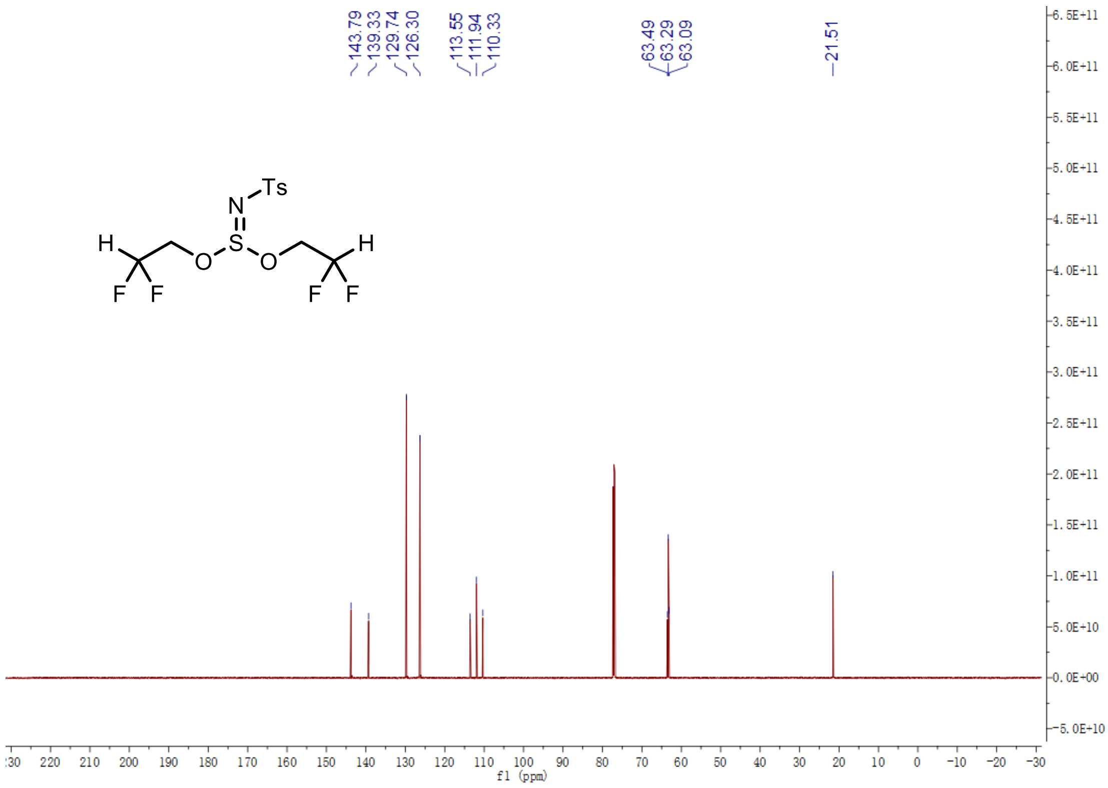


$^{19}F$ NMR spectrum of compound **3f** (564 MHz, $CDCl_3$)

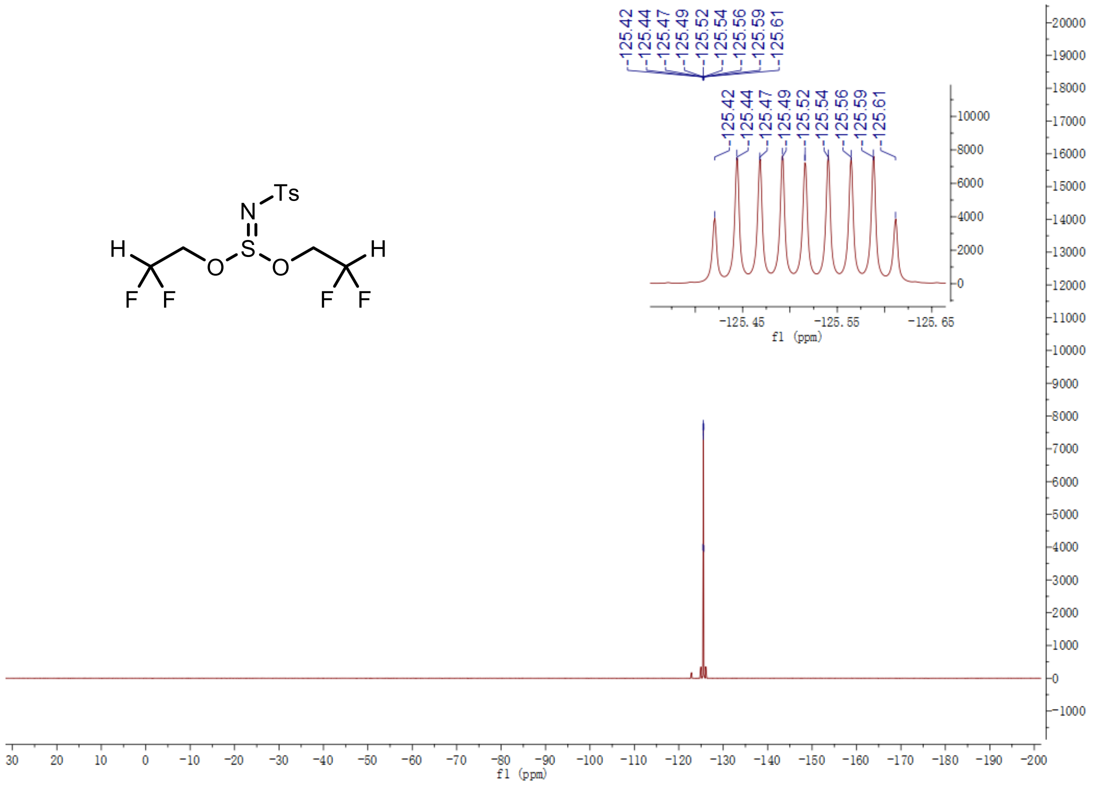

$^{1}$H NMR spectrum of compound **3g** (600 MHz, $CDCl_3$)

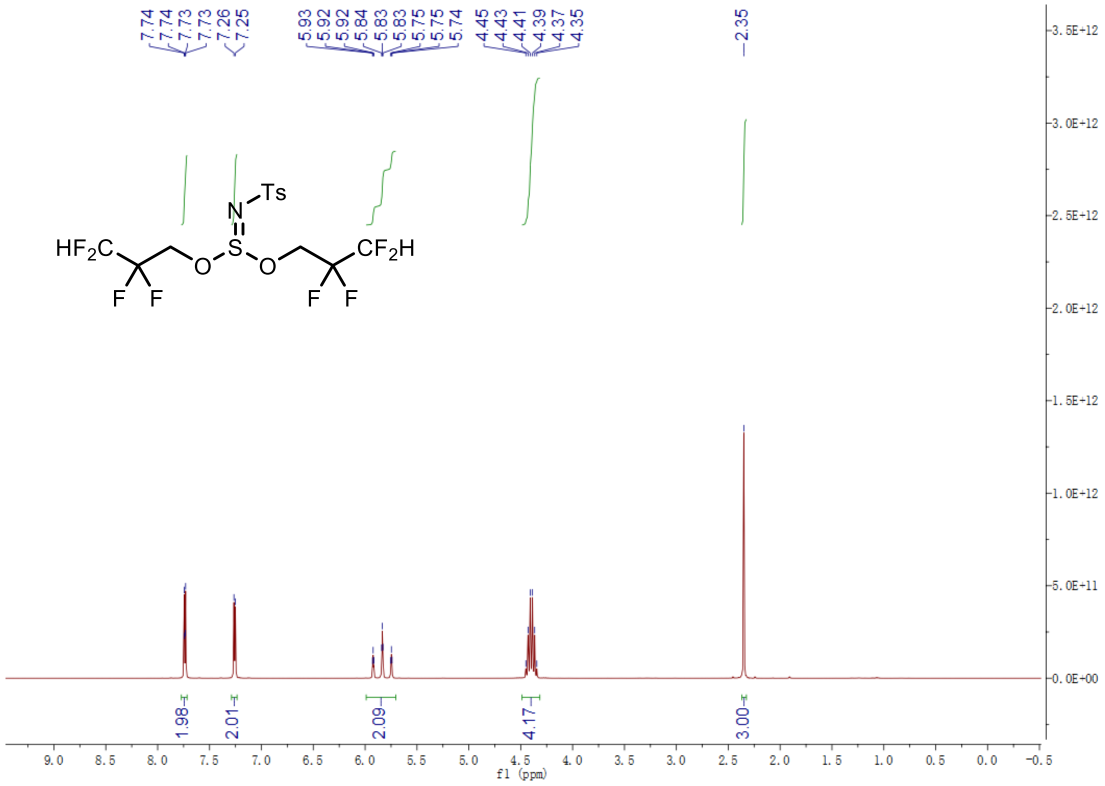


$^{13}$C{$^{1}$H} NMR spectrum of compound **3g** (151 MHz, $CDCl_3$)

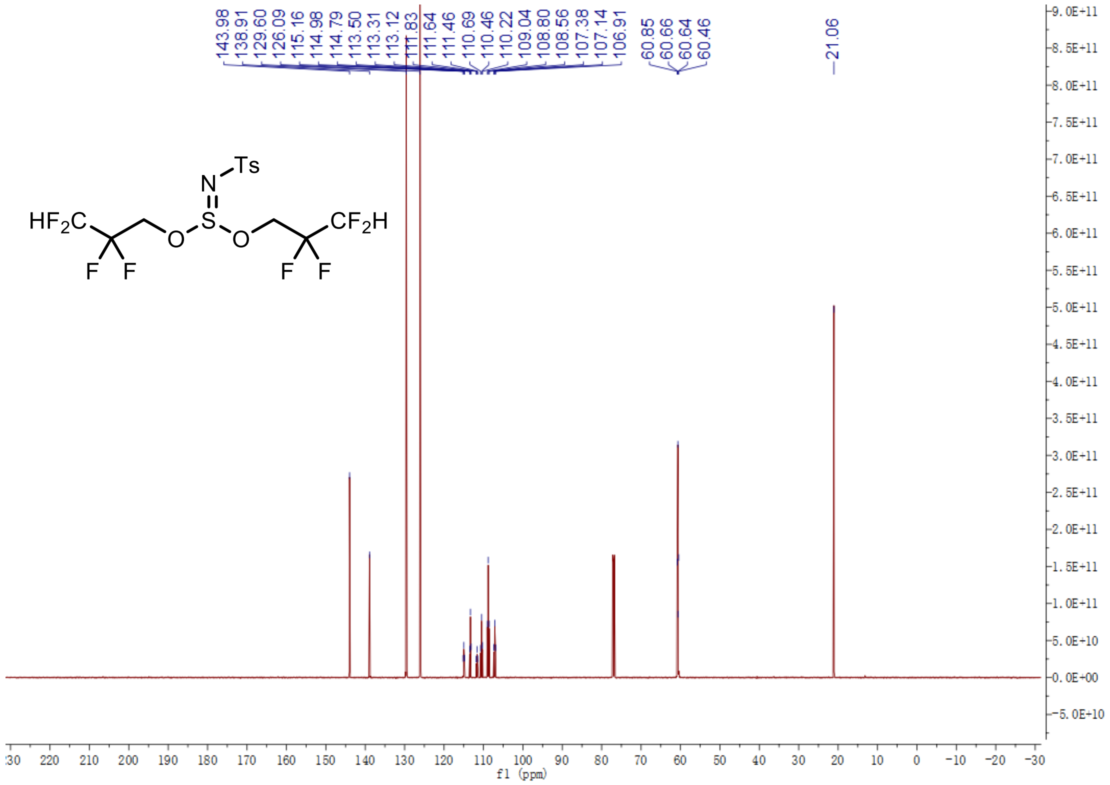

$^{19}F$ NMR spectrum of compound **3g** (564 MHz, $CDCl_3$)

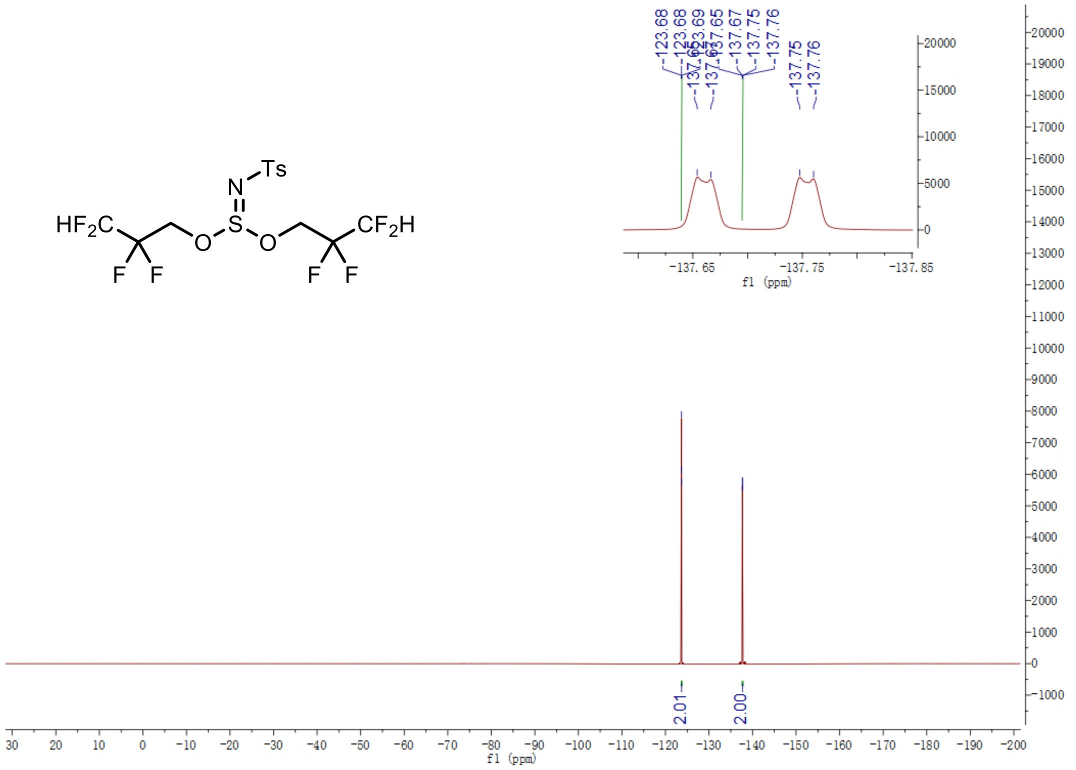

$^{1}H$ NMR spectrum of compound **3h** (600 MHz, $CDCl_3$)

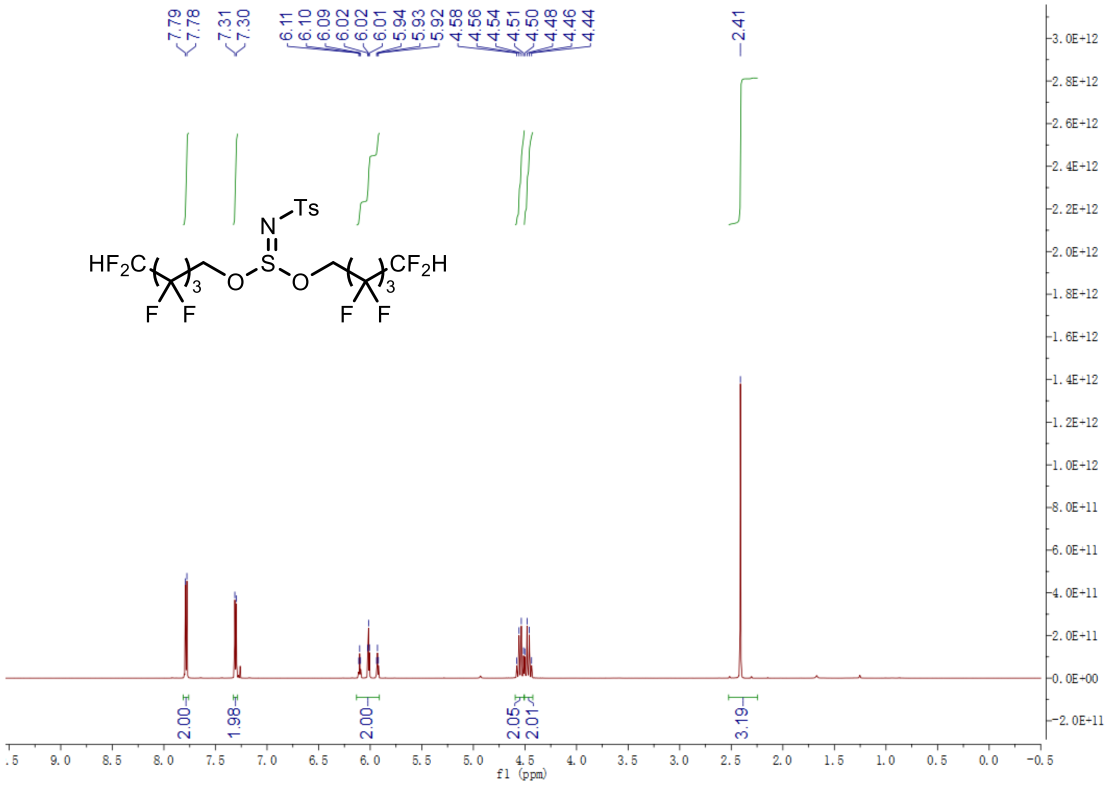

$^{13}C\{^{1}H\}$ NMR spectrum of compound **3h** (151 MHz, $CDCl_3$)

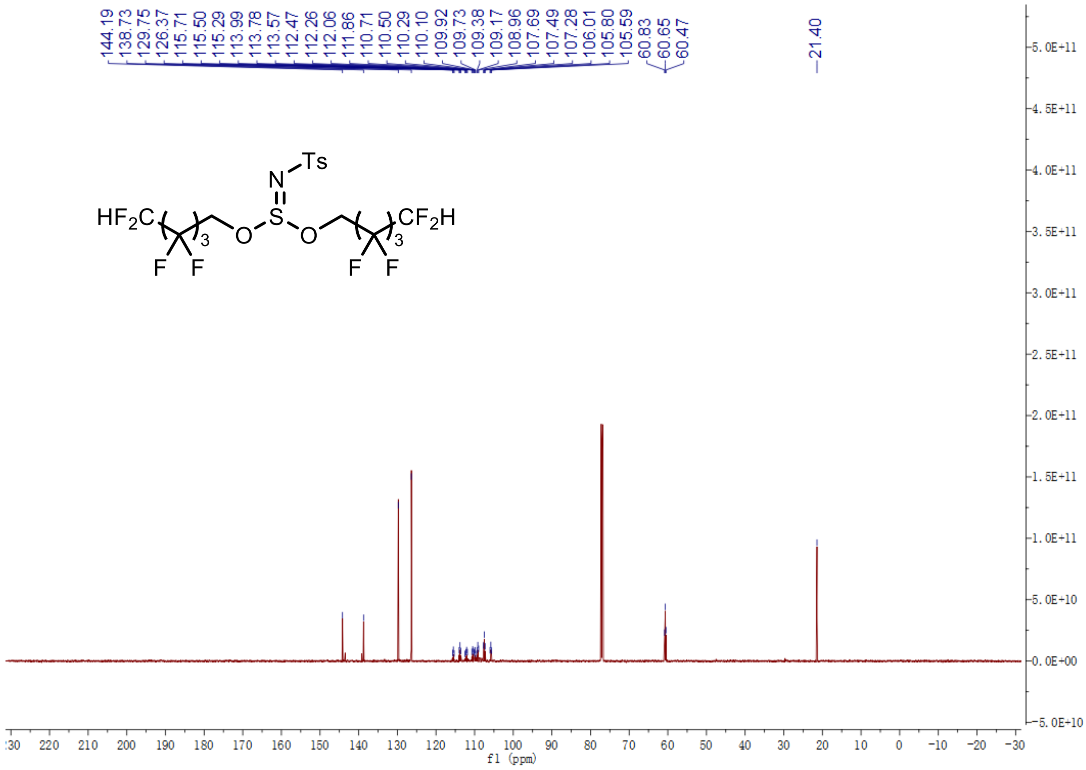

$^{19}F$ NMR spectrum of compound **3h** (564 MHz, $CDCl_3$)

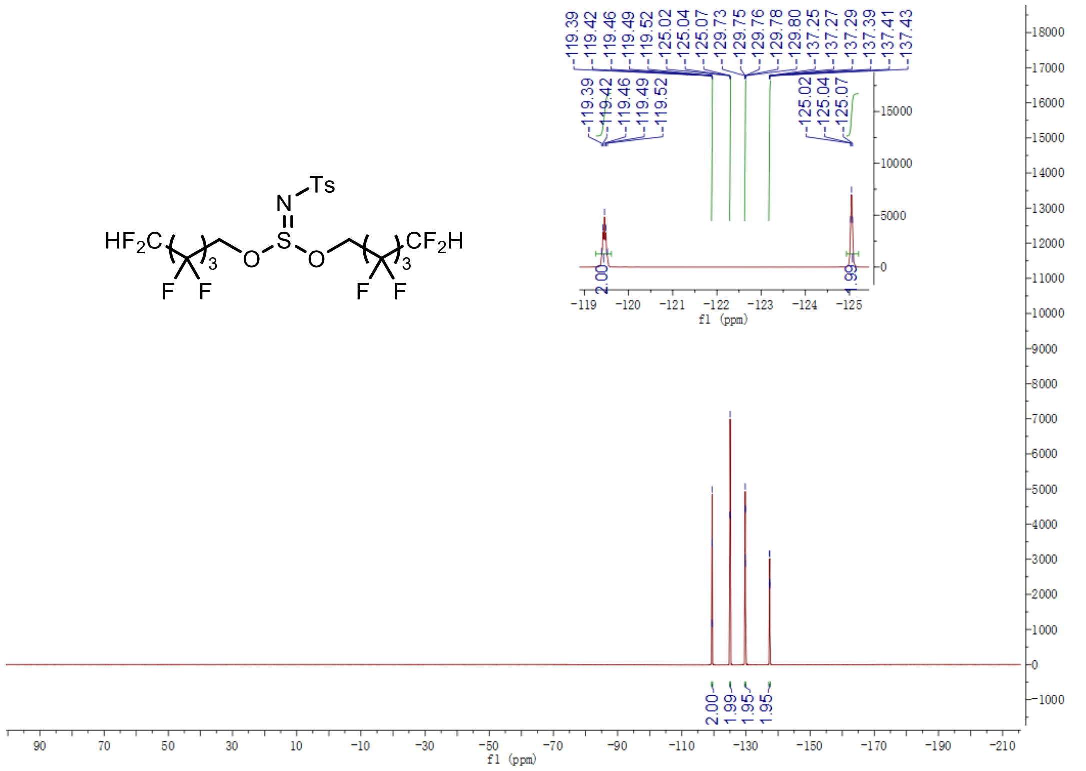

$^{1}$H NMR spectrum of compound **3i** (600 MHz, $CDCl_3$)

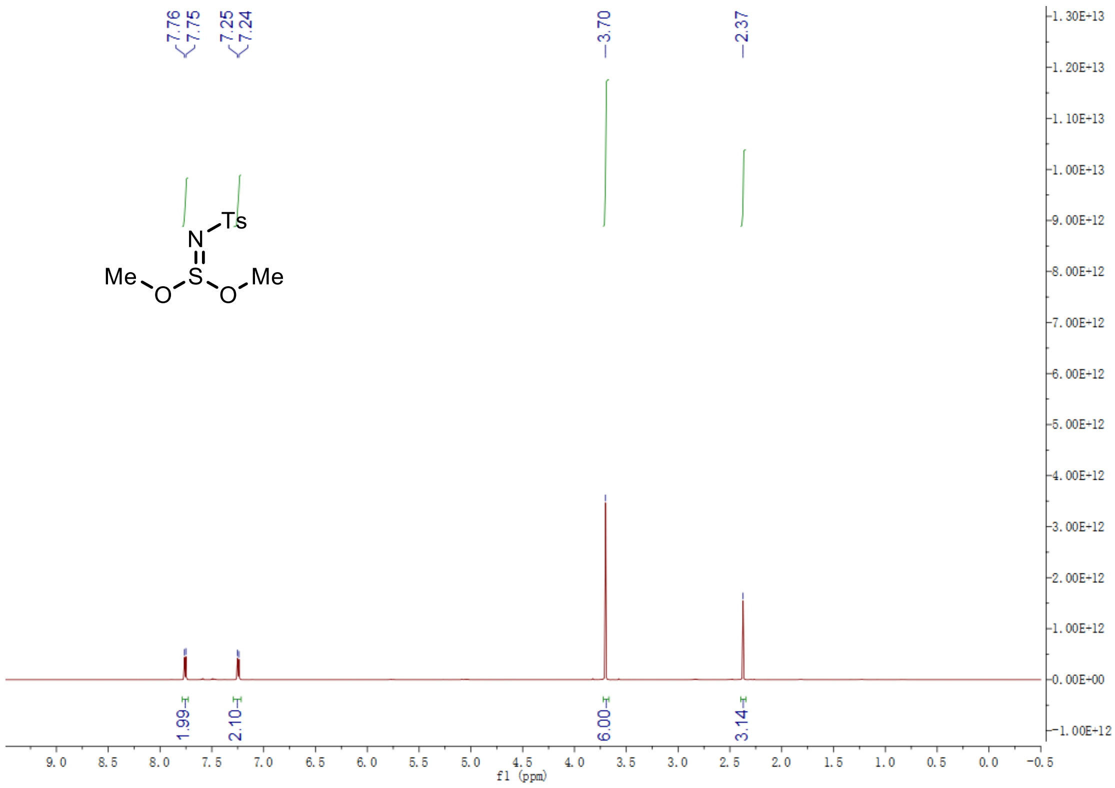


$^{13}$C{$^{1}$H} NMR spectrum of compound **3i** (151 MHz, $CDCl_3$)

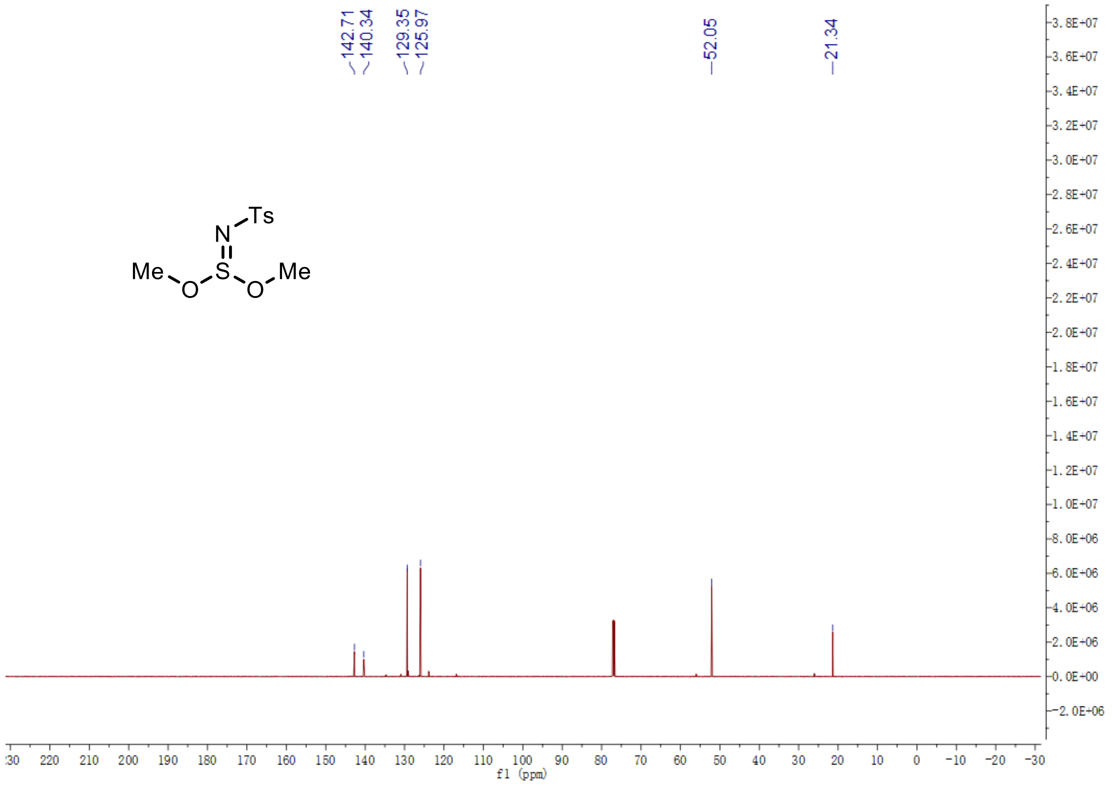

$^{1}$H NMR spectrum of compound **3j** (600 MHz, $CDCl_3$)

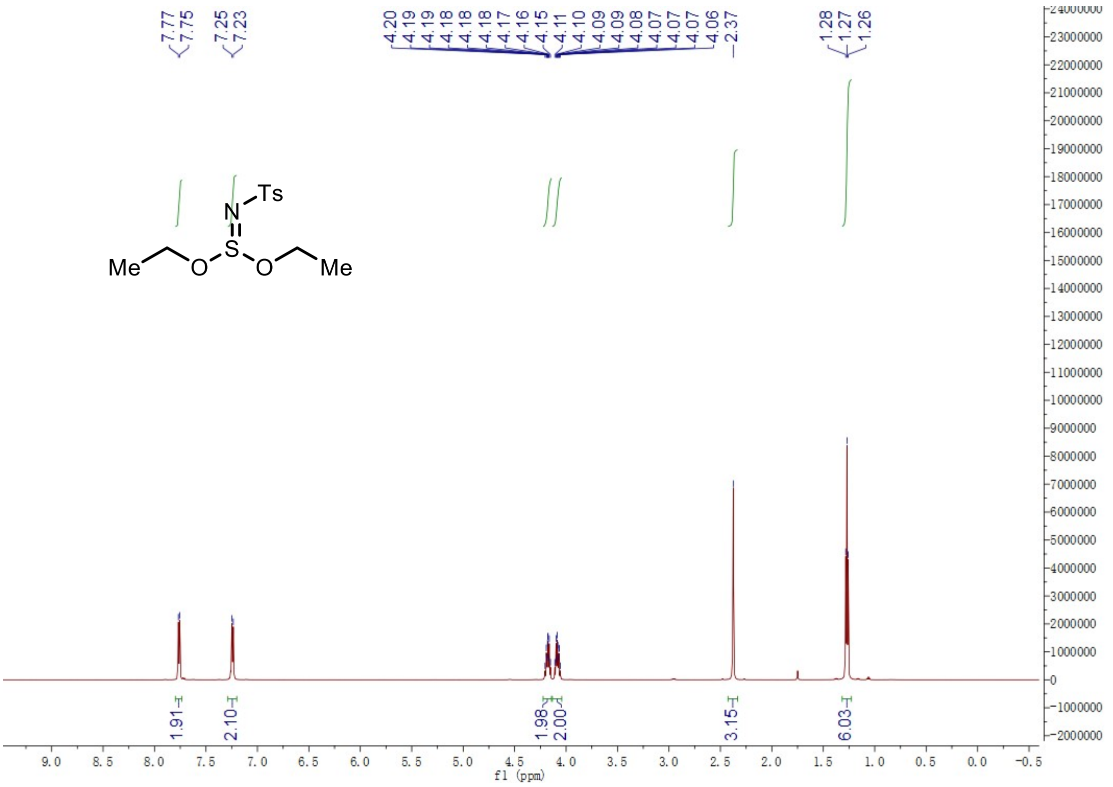


$^{13}$C{$^{1}$H} NMR spectrum of compound **3j** (151 MHz, $CDCl_3$)

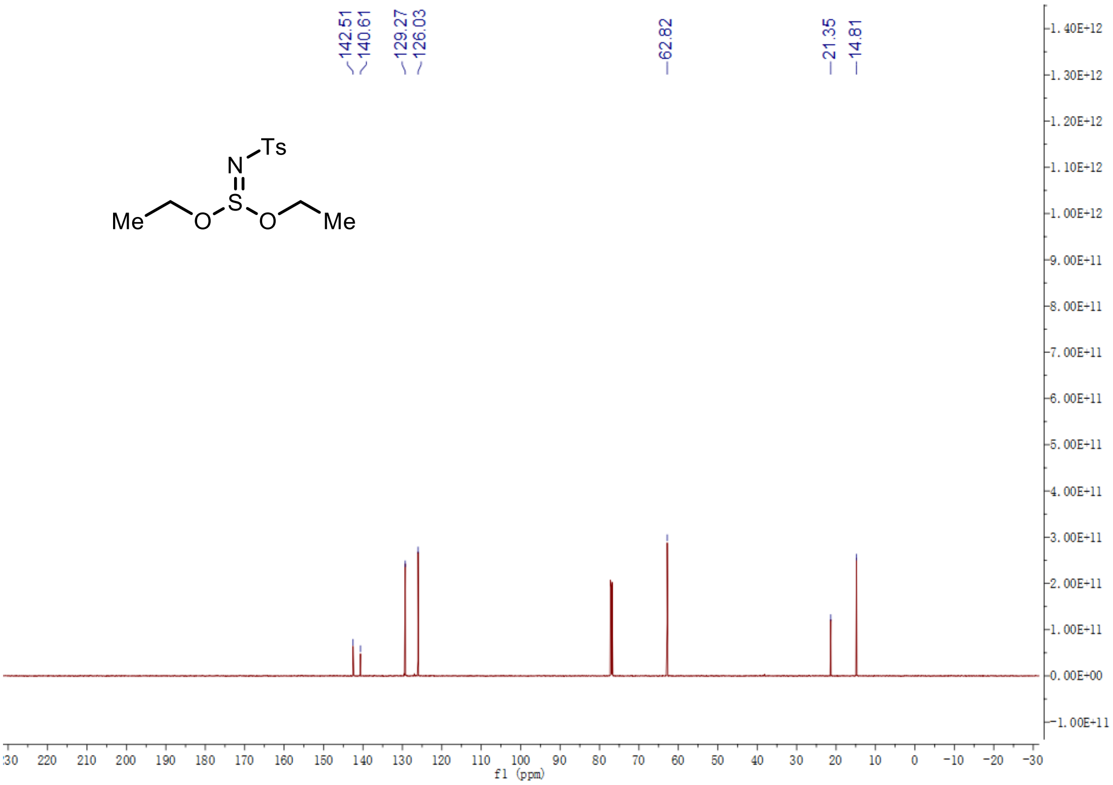

$^{1}$H NMR spectrum of compound **3k** (600 MHz, $CDCl_3$)

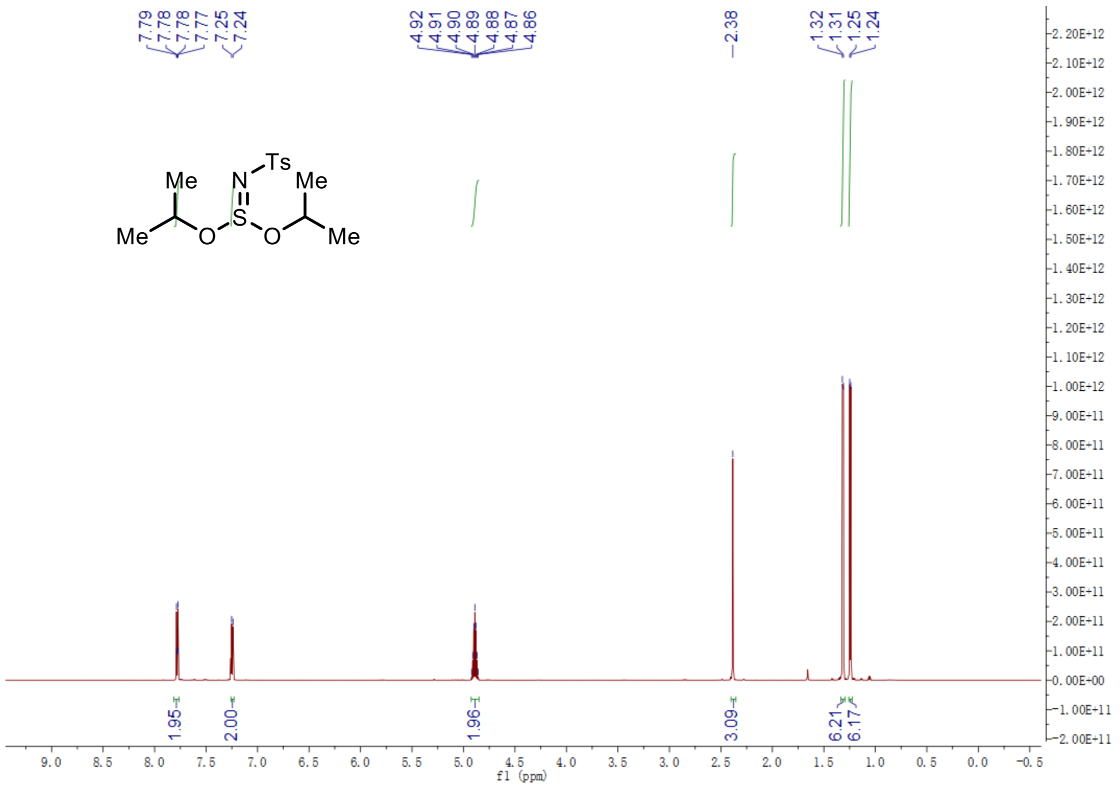


$^{13}$C{$^{1}$H} NMR spectrum of compound **3k** (151 MHz, $CDCl_3$)

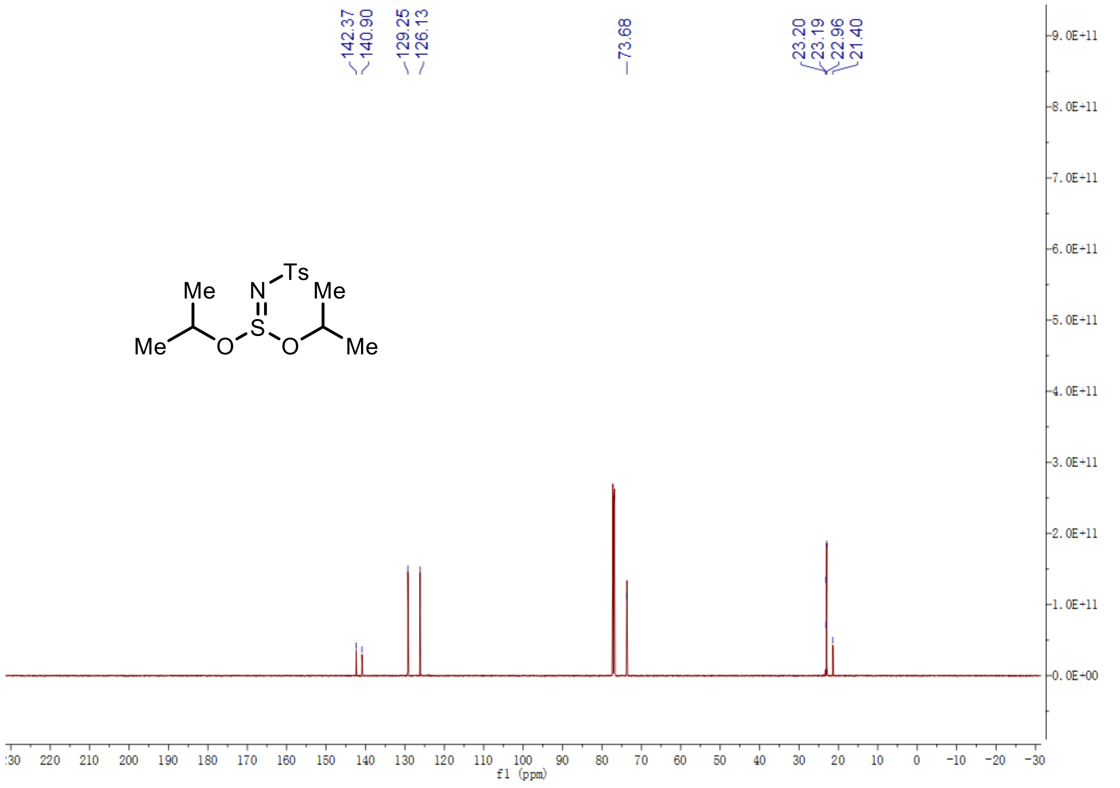

$^{1}$H NMR spectrum of compound **3l** (400 MHz, $CDCl_3$)

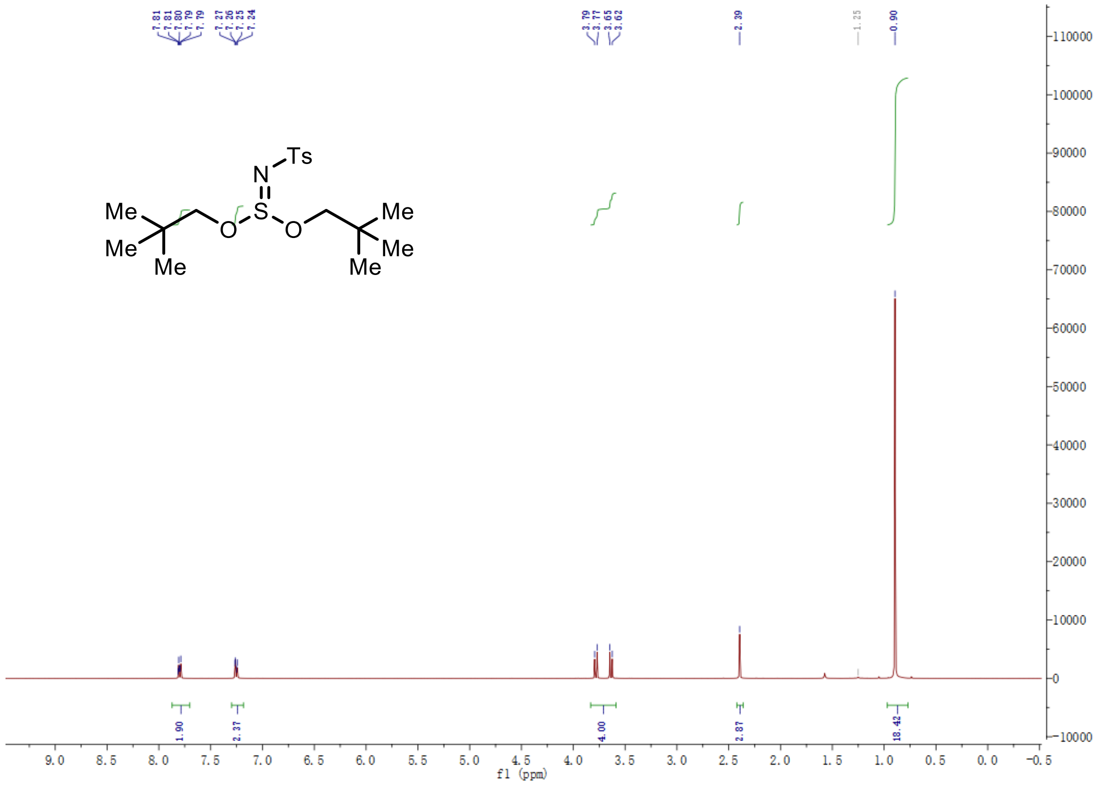


$^{13}C\{^{1}H\}$ NMR spectrum of compound **3l** (101 MHz, $CDCl_3$)

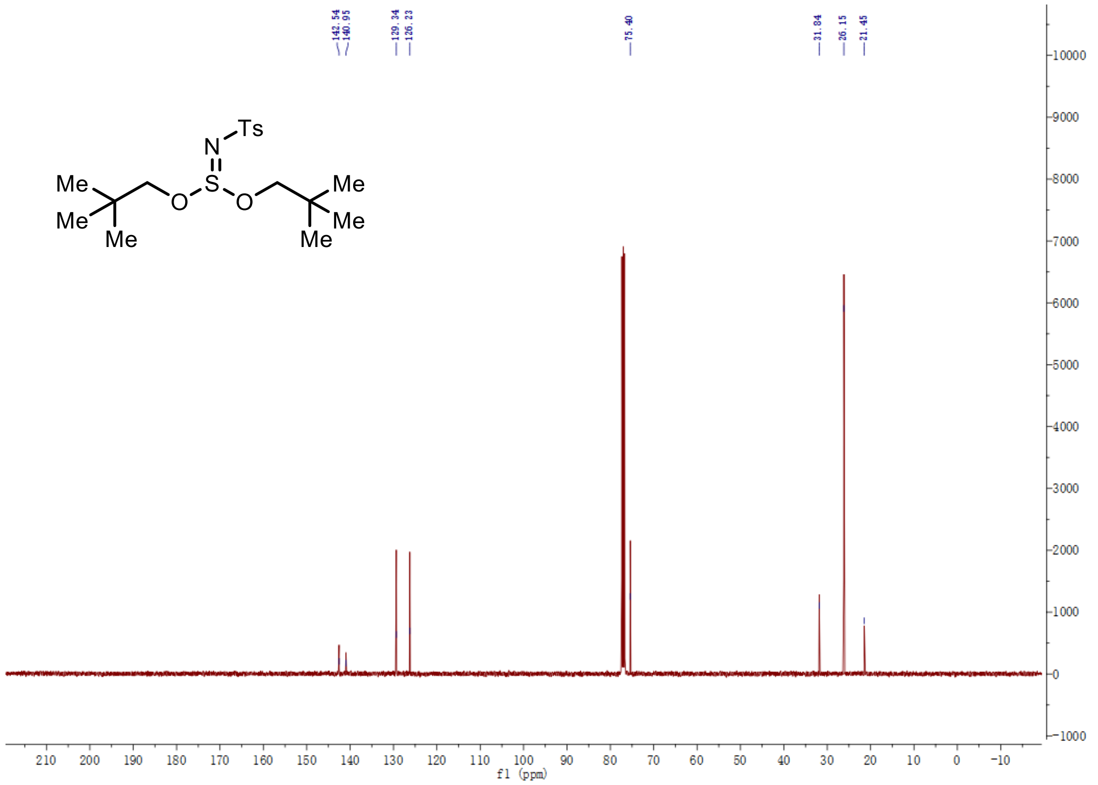

$^{1}$H NMR spectrum of compound **3m** (600 MHz, $CDCl_3$)

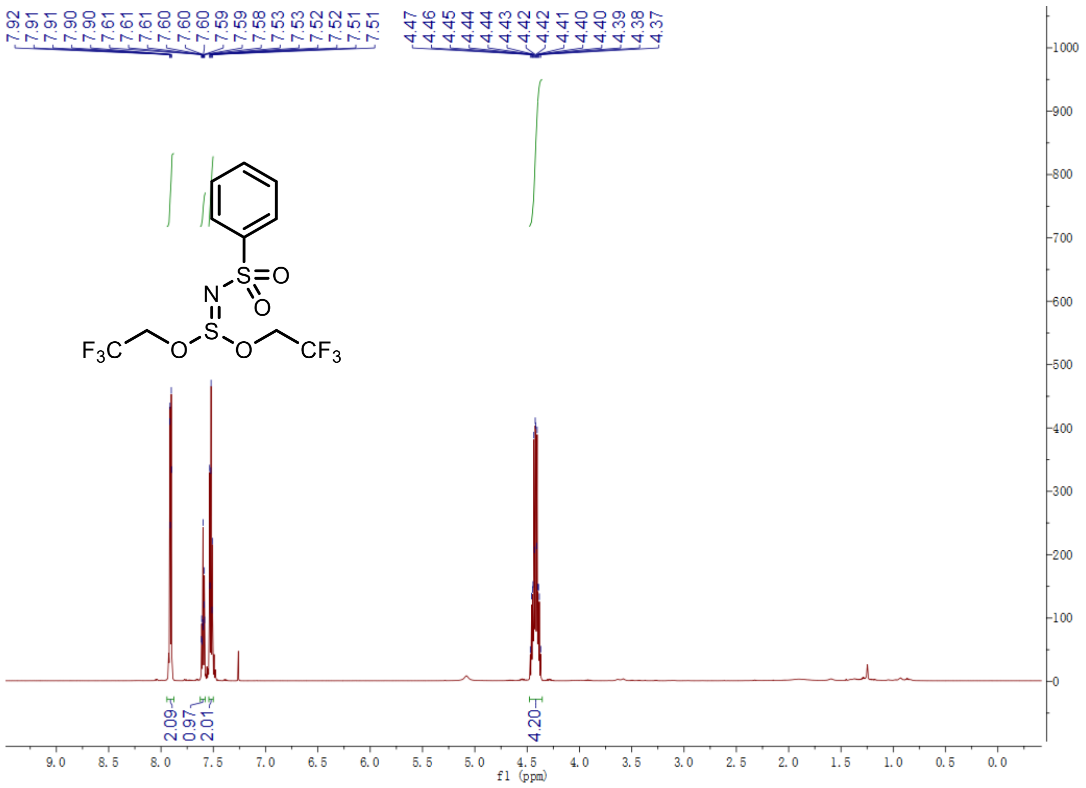


$^{13}$C{$^{1}$H} NMR spectrum of compound **3m** (151 MHz, $CDCl_3$)

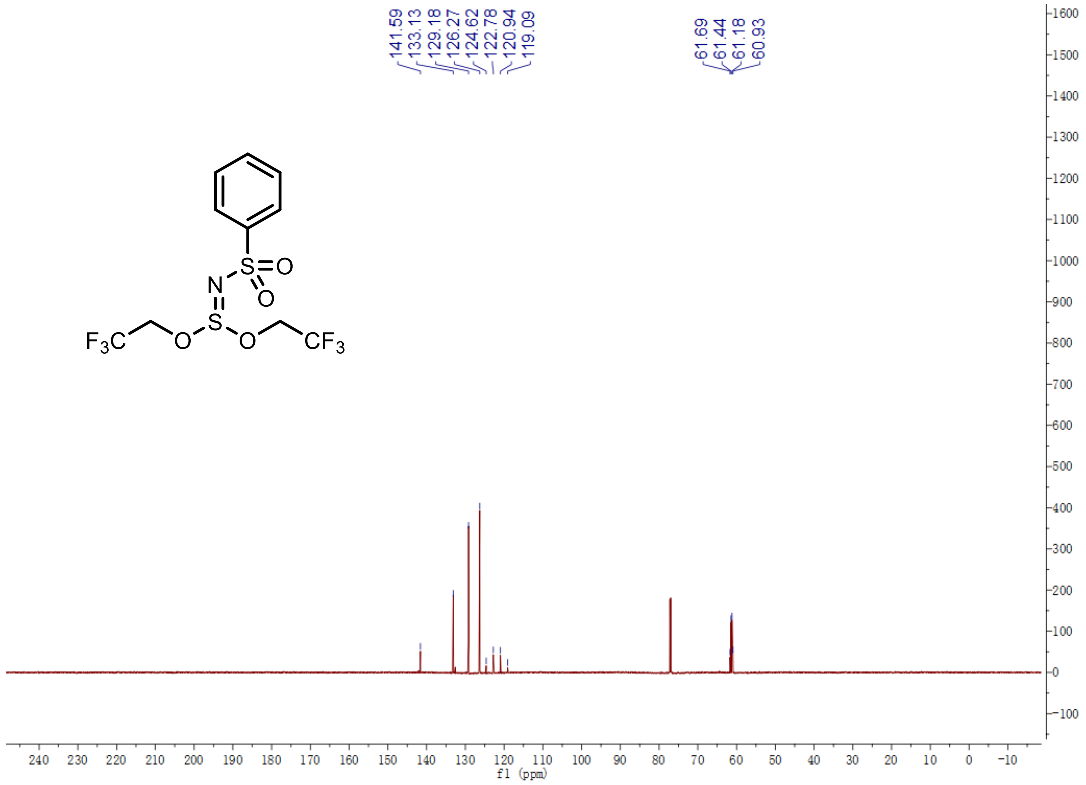

$^{19}F$ NMR spectrum of compound **3m** (564 MHz, $CDCl_3$)

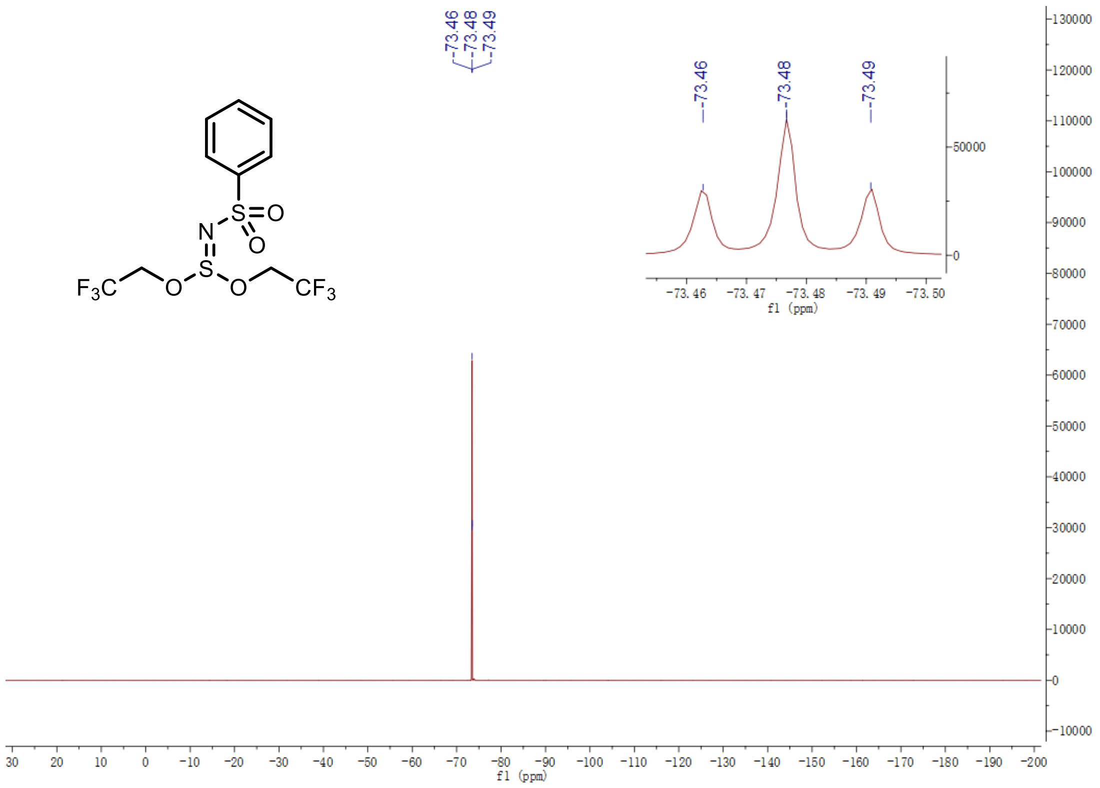

$^{1}H$ NMR spectrum of compound **3n** (600 MHz, $CDCl_3$)

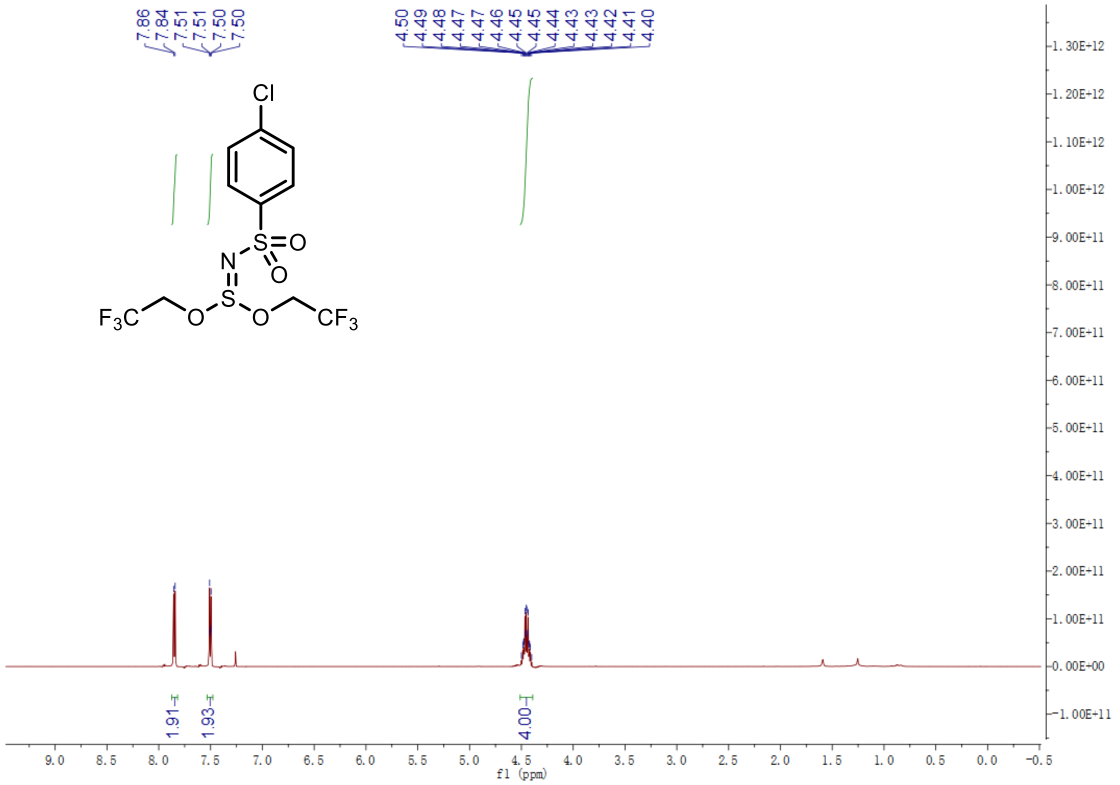

$^{13}C\{^{1}H\}$ NMR spectrum of compound **3n** (151 MHz, $CDCl_3$)

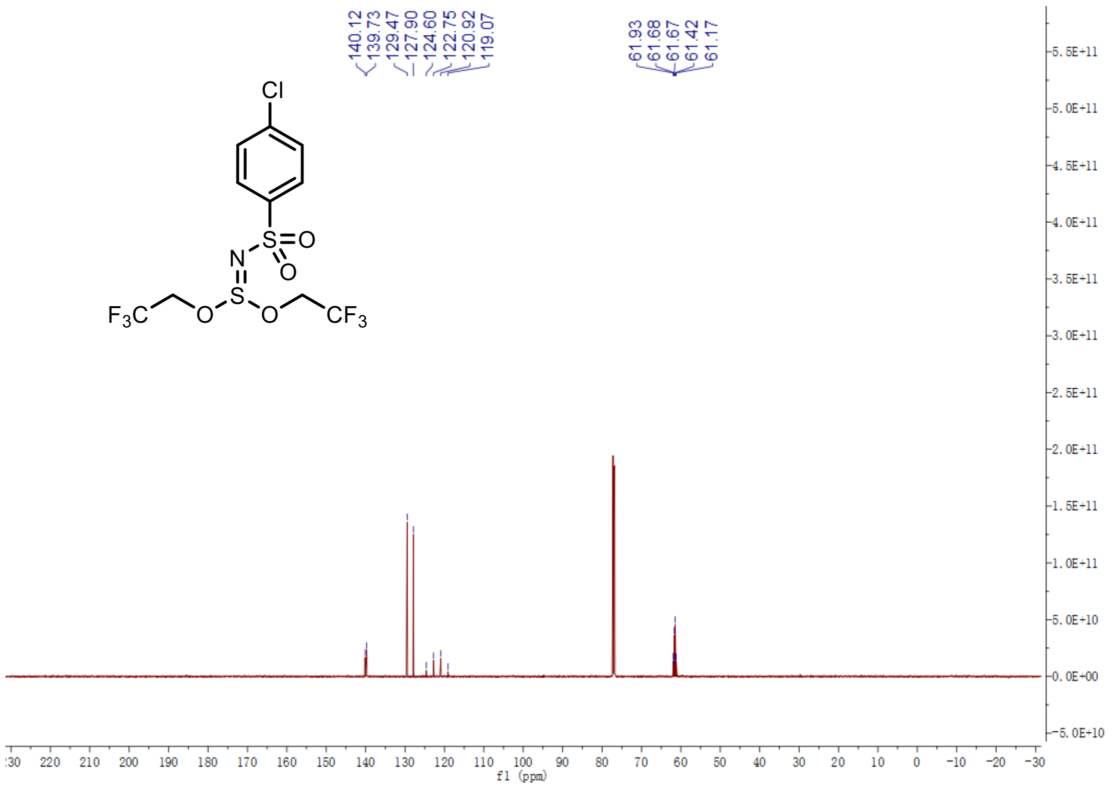

$^{19}F$ NMR spectrum of compound **3n** (564 MHz, $CDCl_3$)

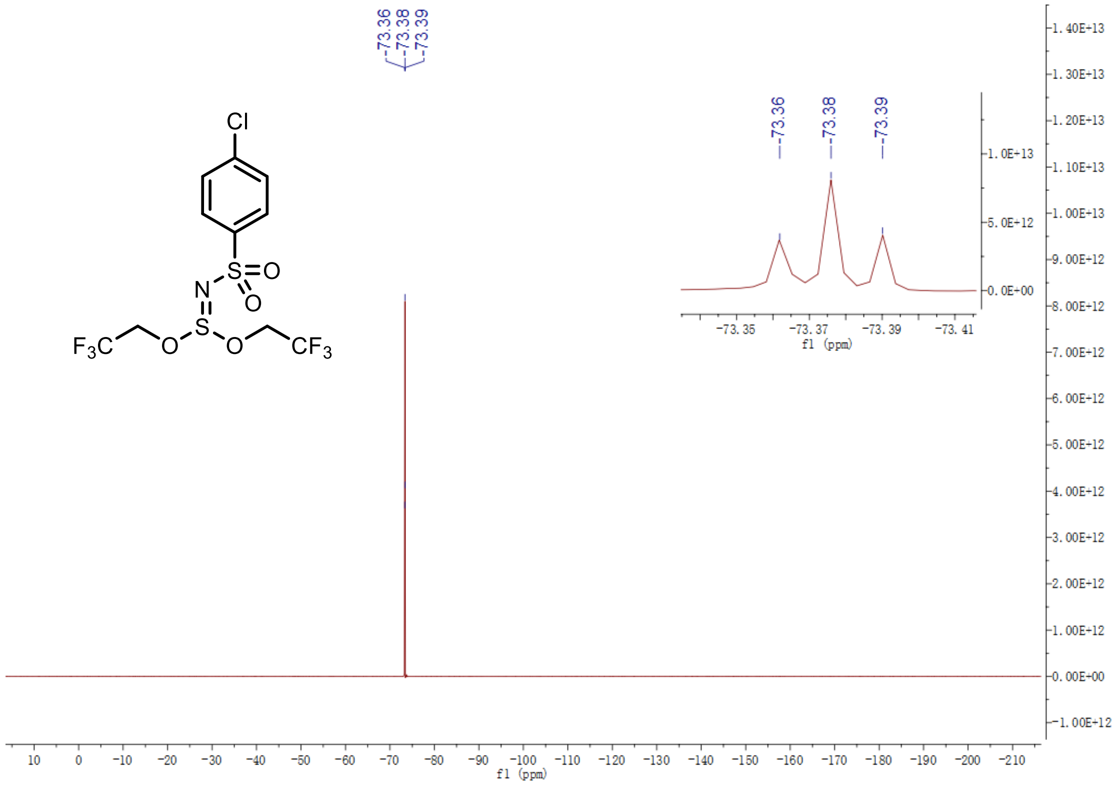

$^{1}H$ NMR spectrum of compound **3o** (600 MHz, $CDCl_3$)

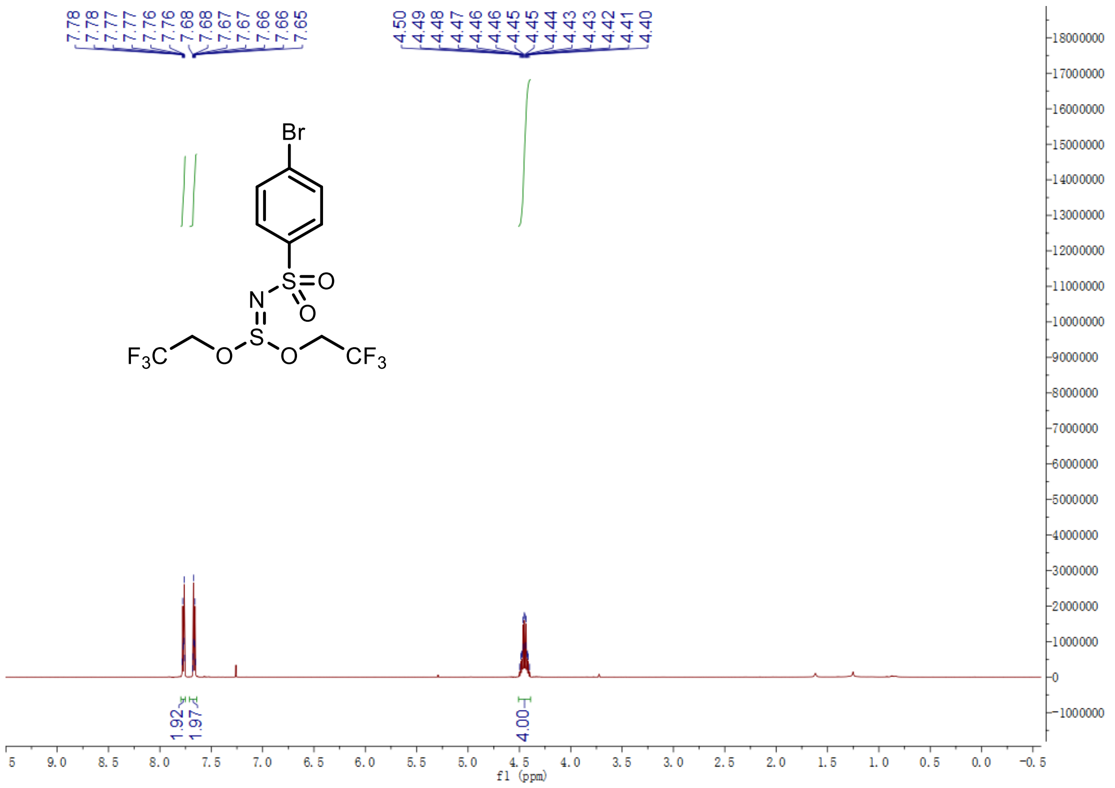


$^{13}C\{^{1}H\}$ NMR spectrum of compound **3o** (151 MHz, $CDCl_3$)

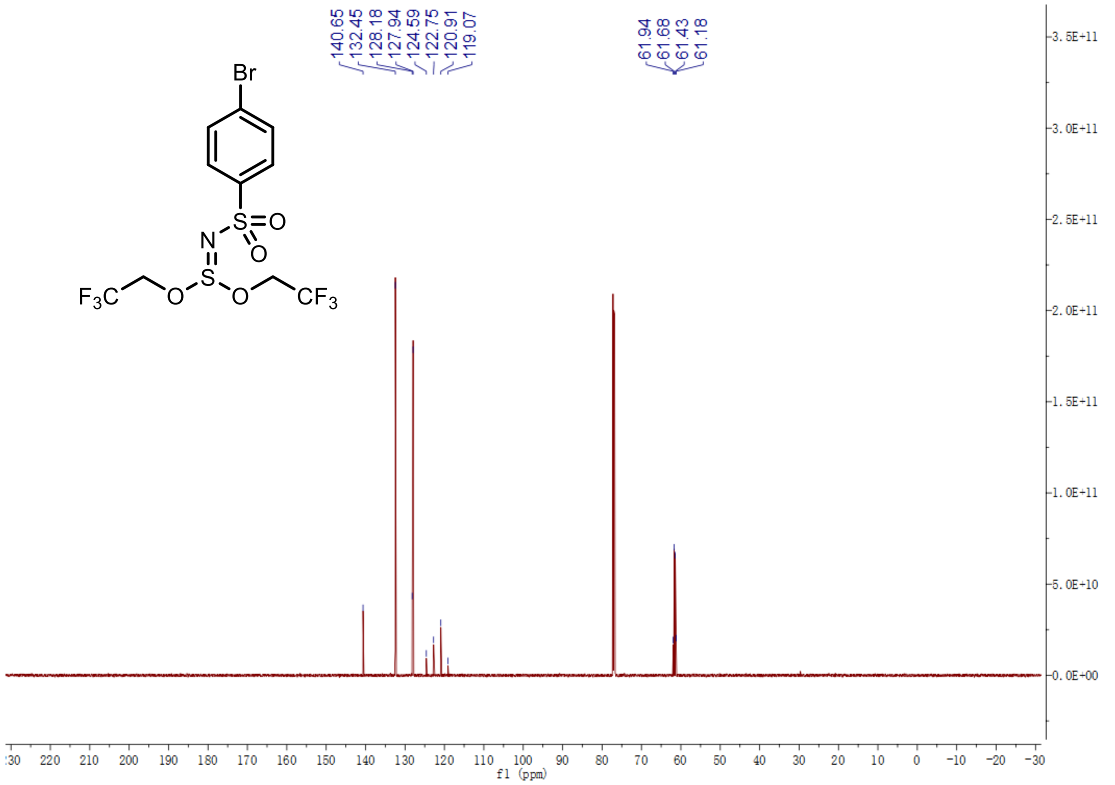

$^{19}$F NMR spectrum of compound **3o** (564 MHz, $CDCl_3$)

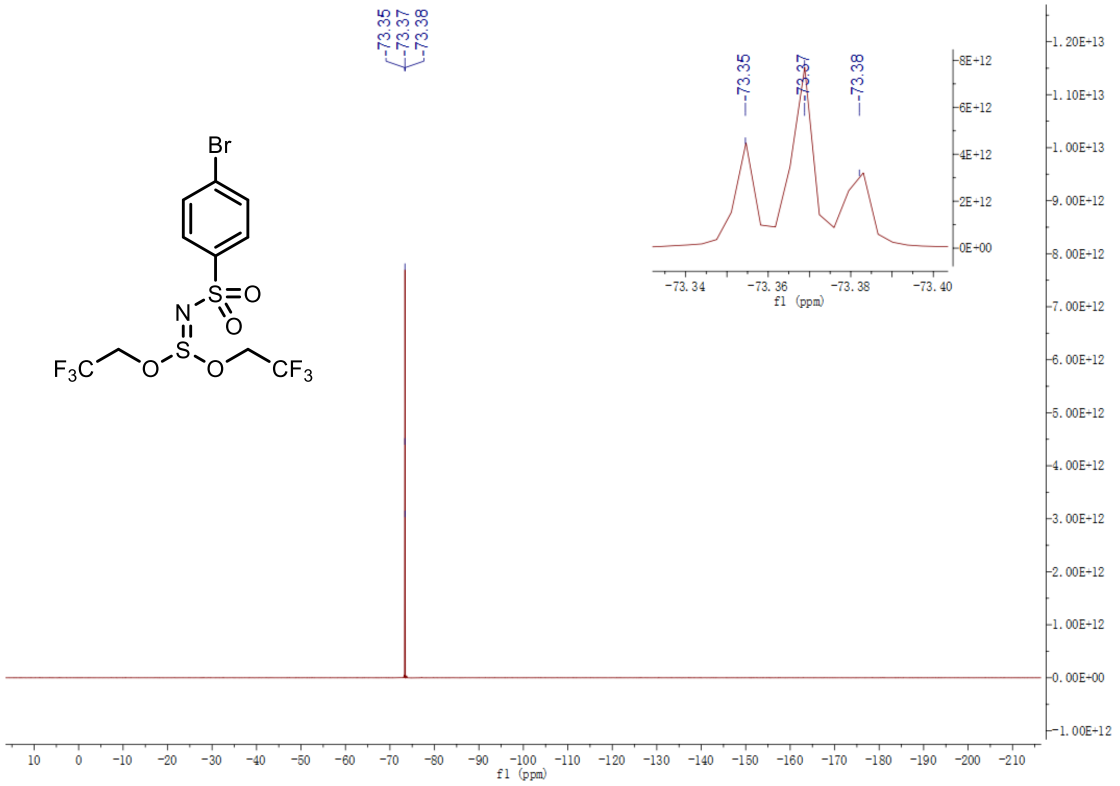


$^{1}$H NMR spectrum of compound **3p** (600 MHz, $CDCl_3$)

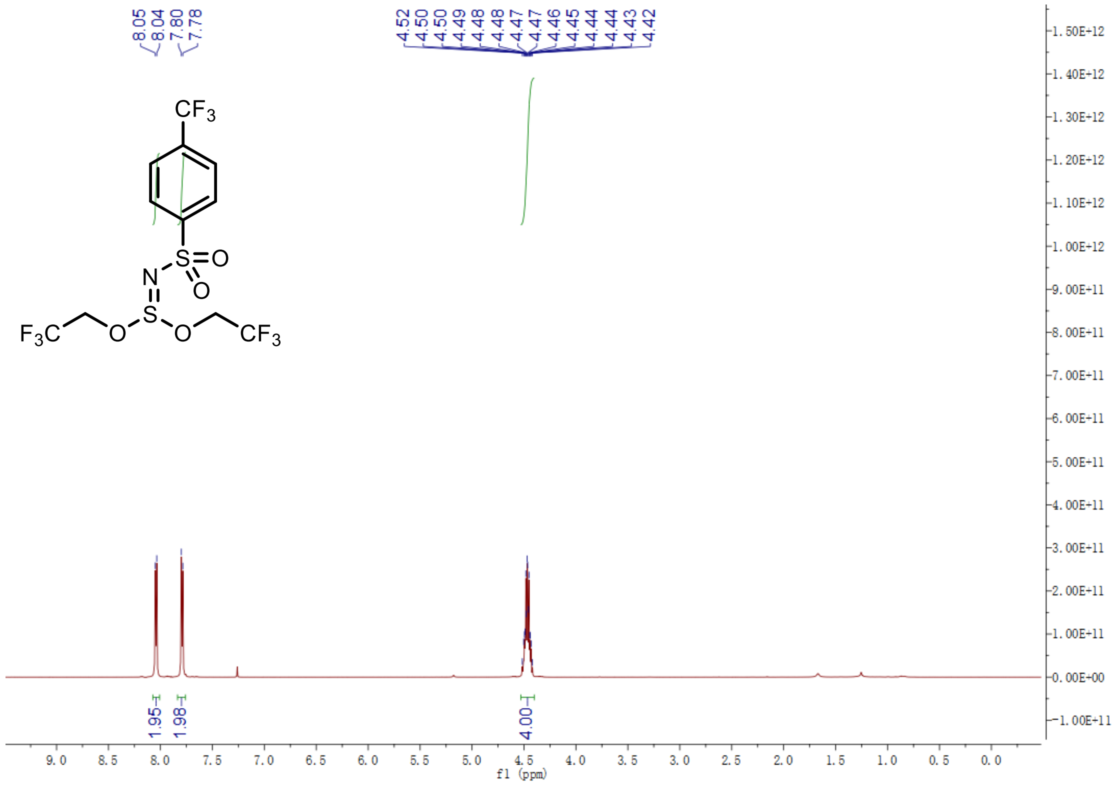

## $^{13}C\{^{1}H\}$ NMR spectrum of compound **3p** (151 MHz, $CDCl_3$)

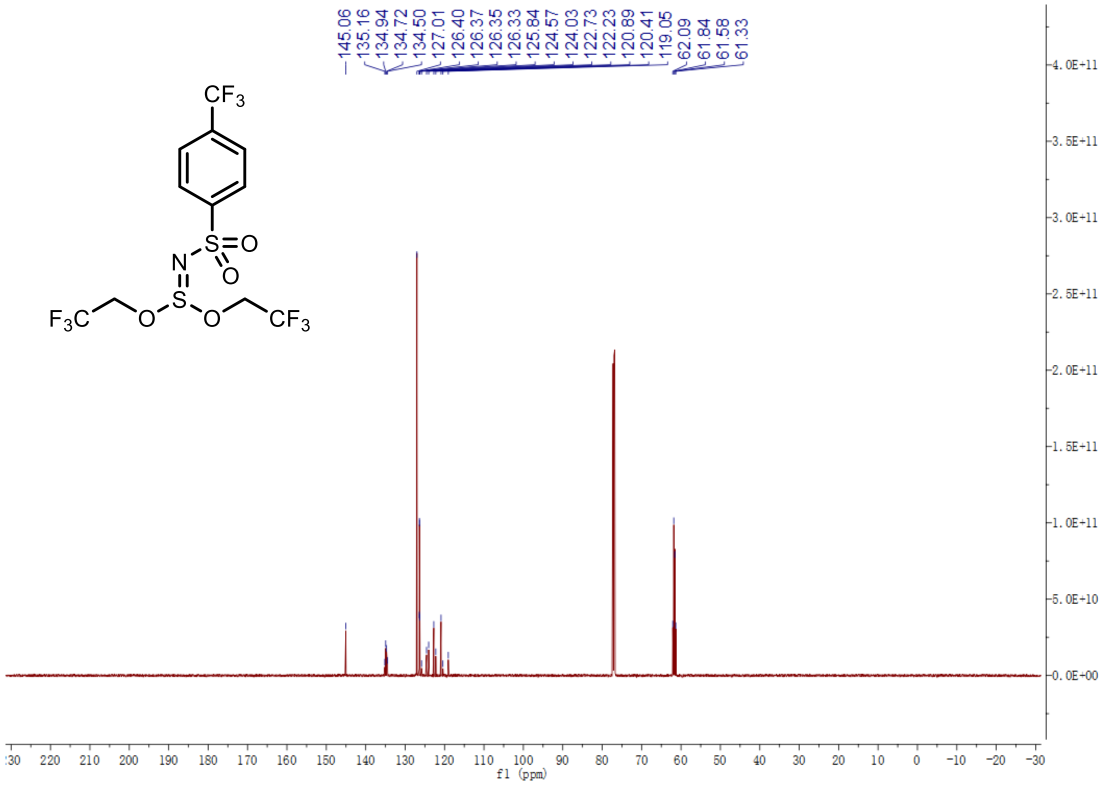


## $^{19}F$ NMR spectrum of compound **3p** (564 MHz, $CDCl_3$)

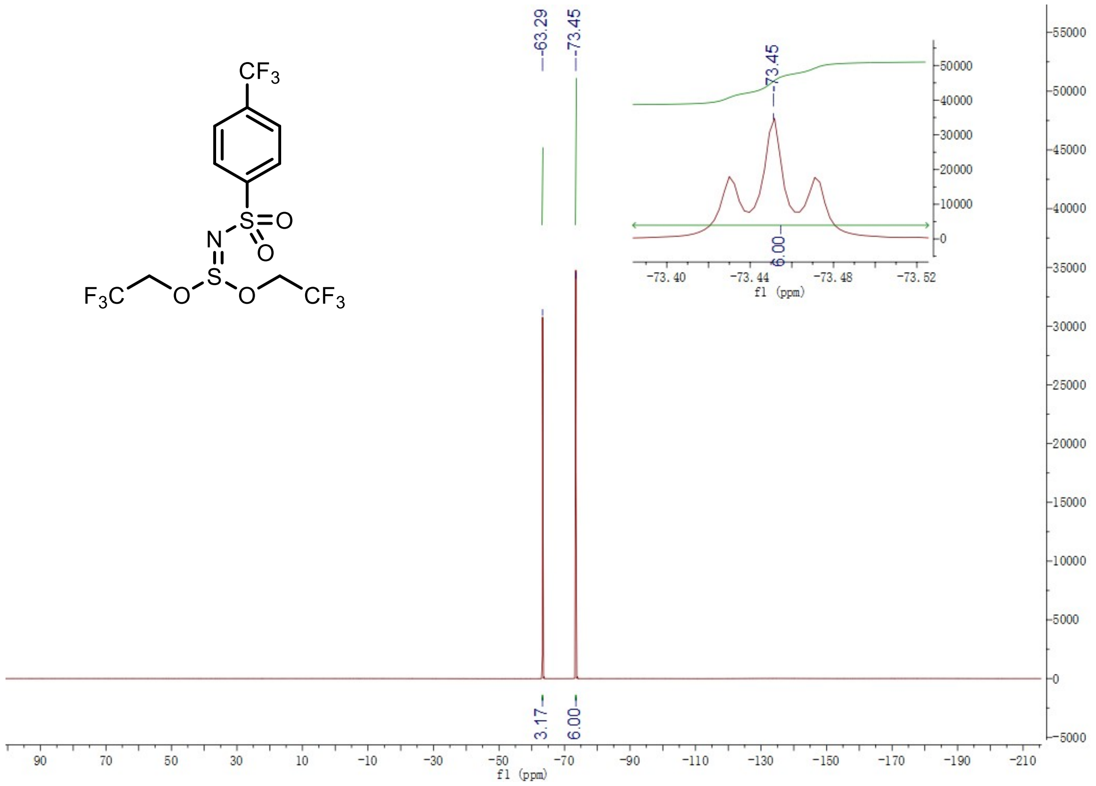

$^{1}$H NMR spectrum of compound **3q** (600 MHz, $CDCl_3$)

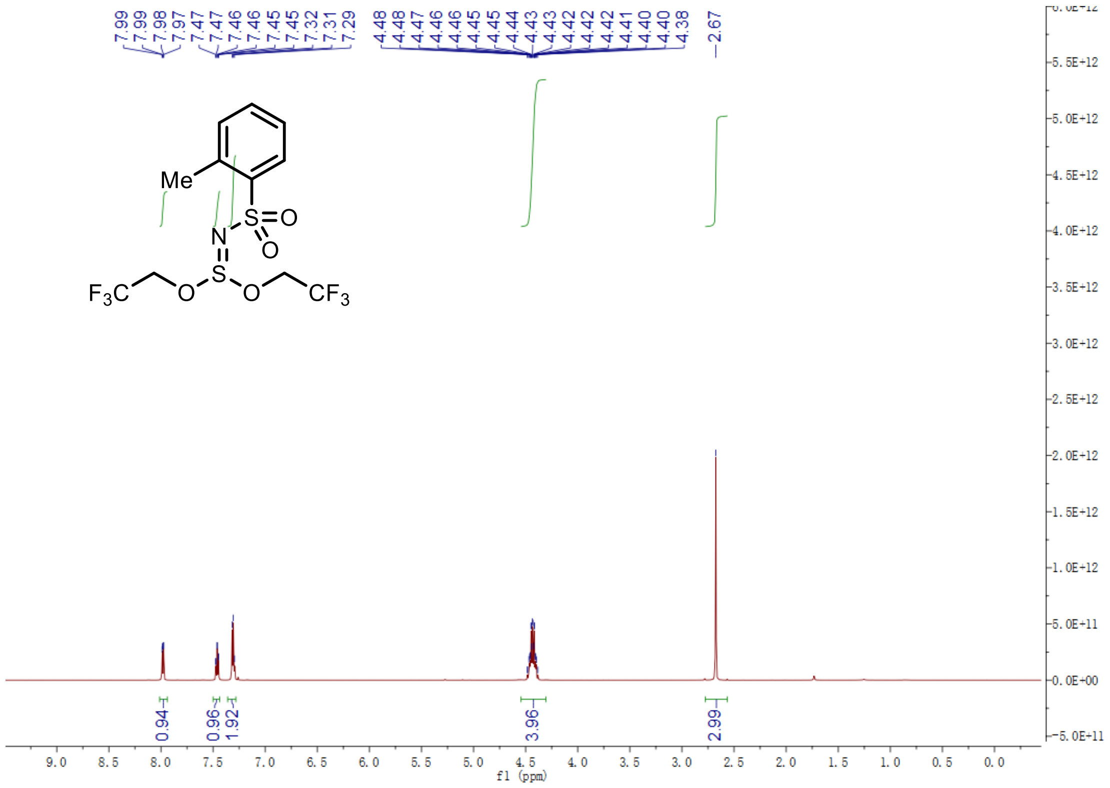


$^{13}$C{$^{1}$H} NMR spectrum of compound **3q** (151 MHz, $CDCl_3$)

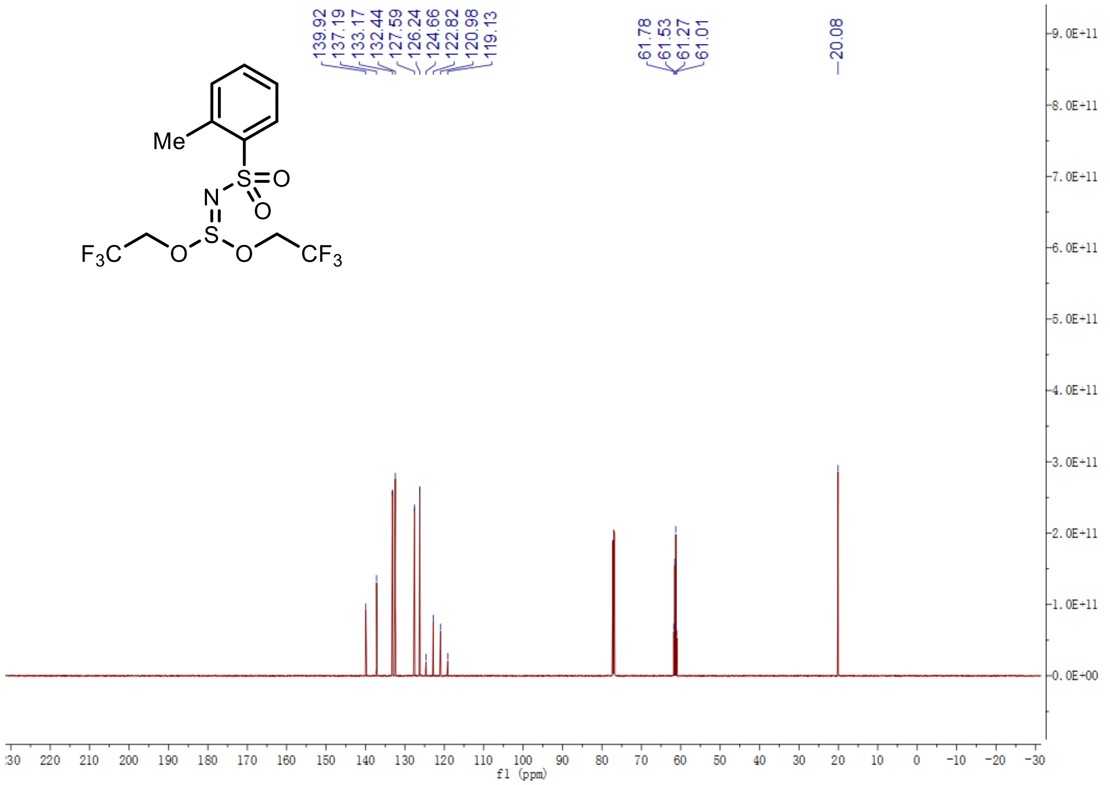

## $^{19}$F NMR spectrum of compound **3q** (564 MHz, $CDCl_3$)

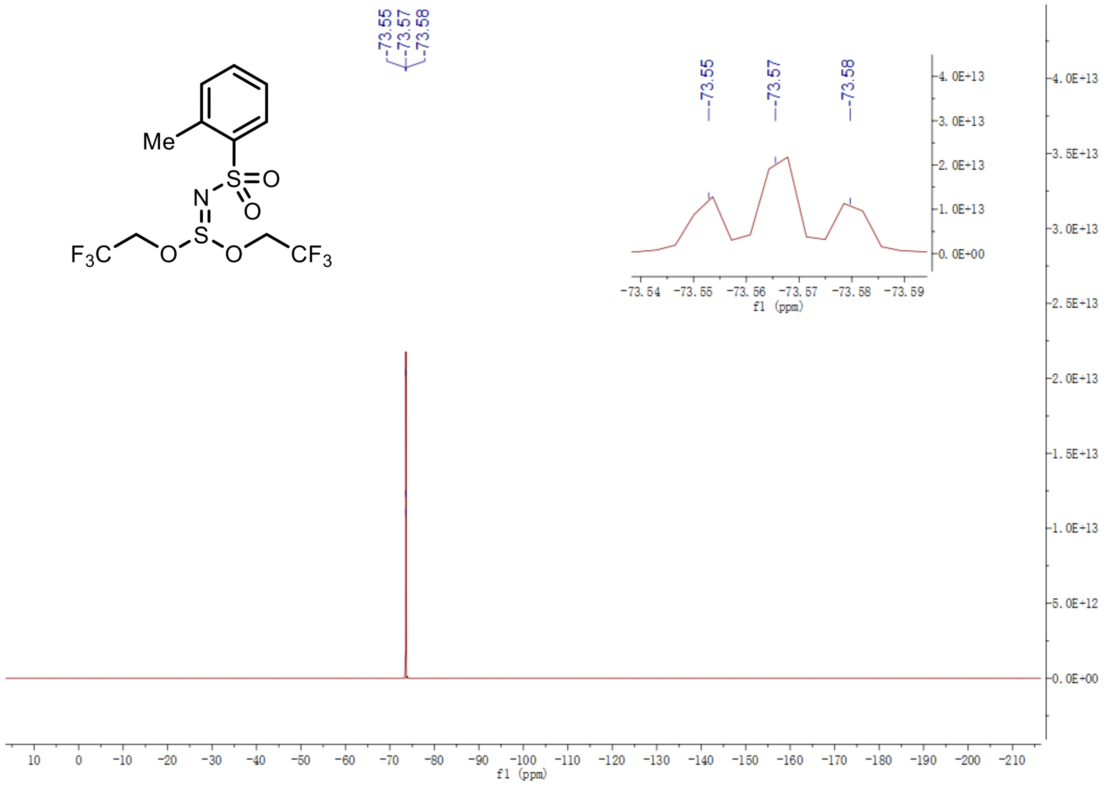


## $^{1}$H NMR spectrum of compound **3r** (600 MHz, $CDCl_3$)

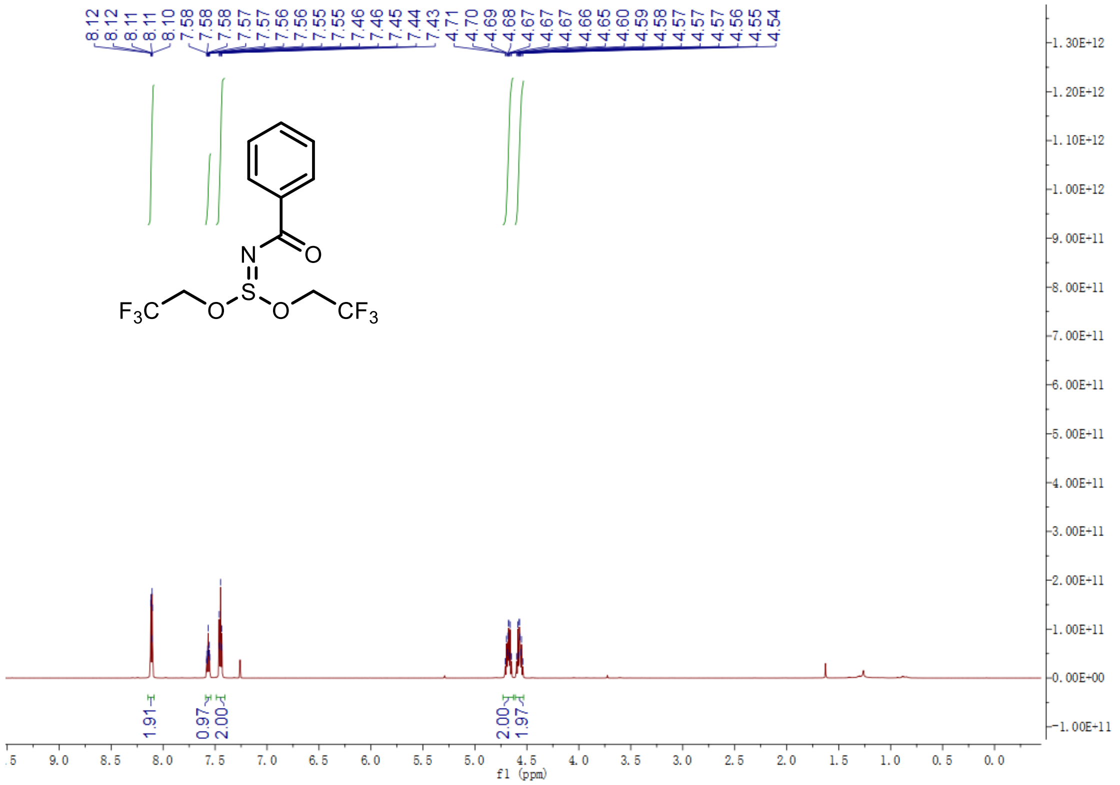

$^{13}C\{^1H\}$ NMR spectrum of compound **3r** (151 MHz, $CDCl_3$)

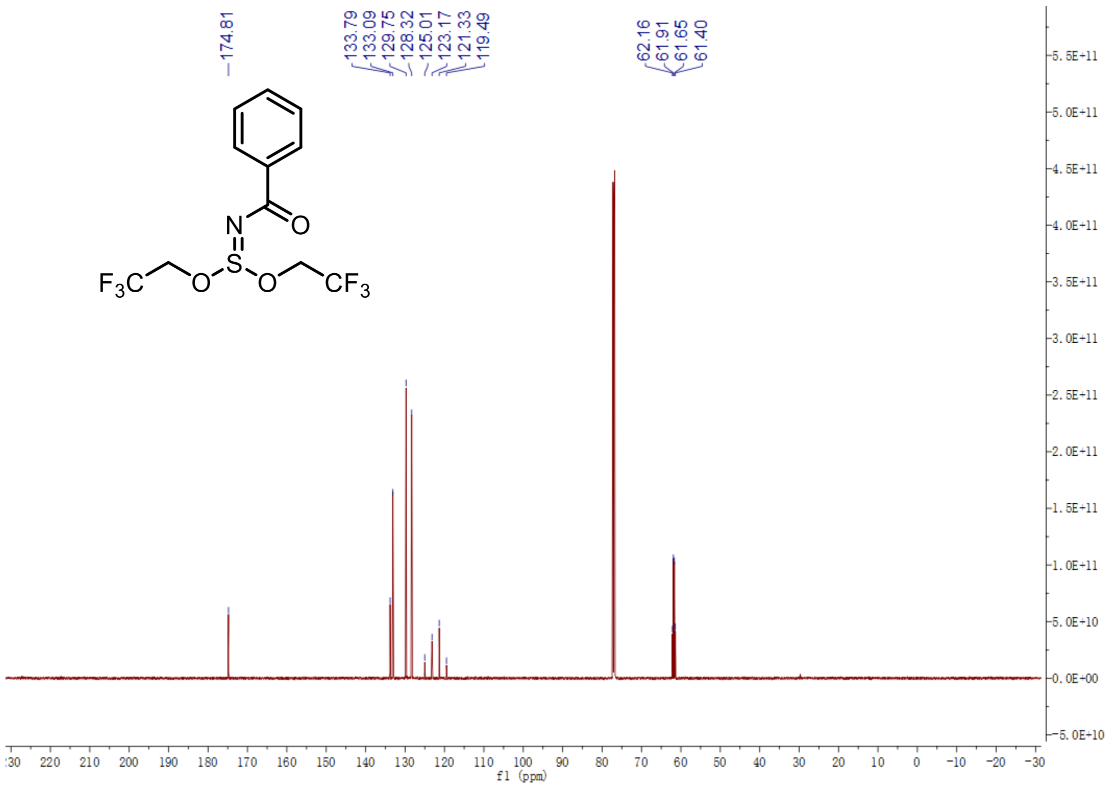


$^{19}F$ NMR spectrum of compound **3r** (564 MHz, $CDCl_3$)

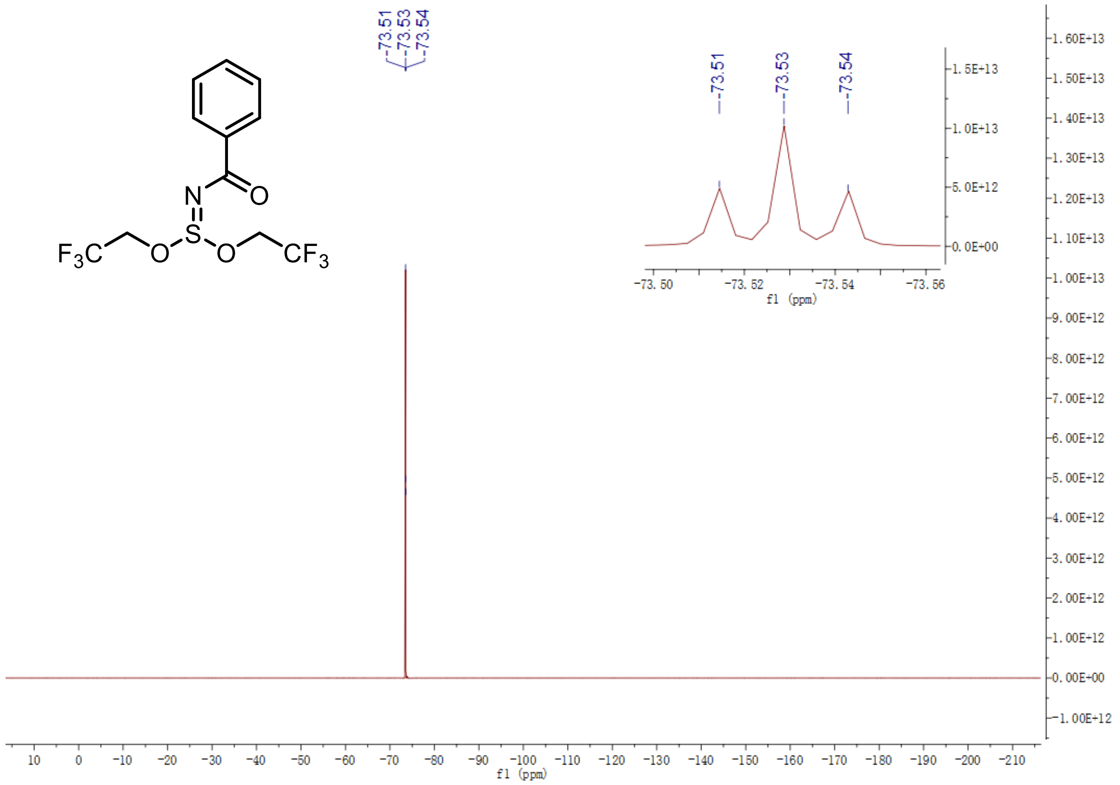

$^{1}$H NMR spectrum of compound **3s** (600 MHz, $CDCl_3$)

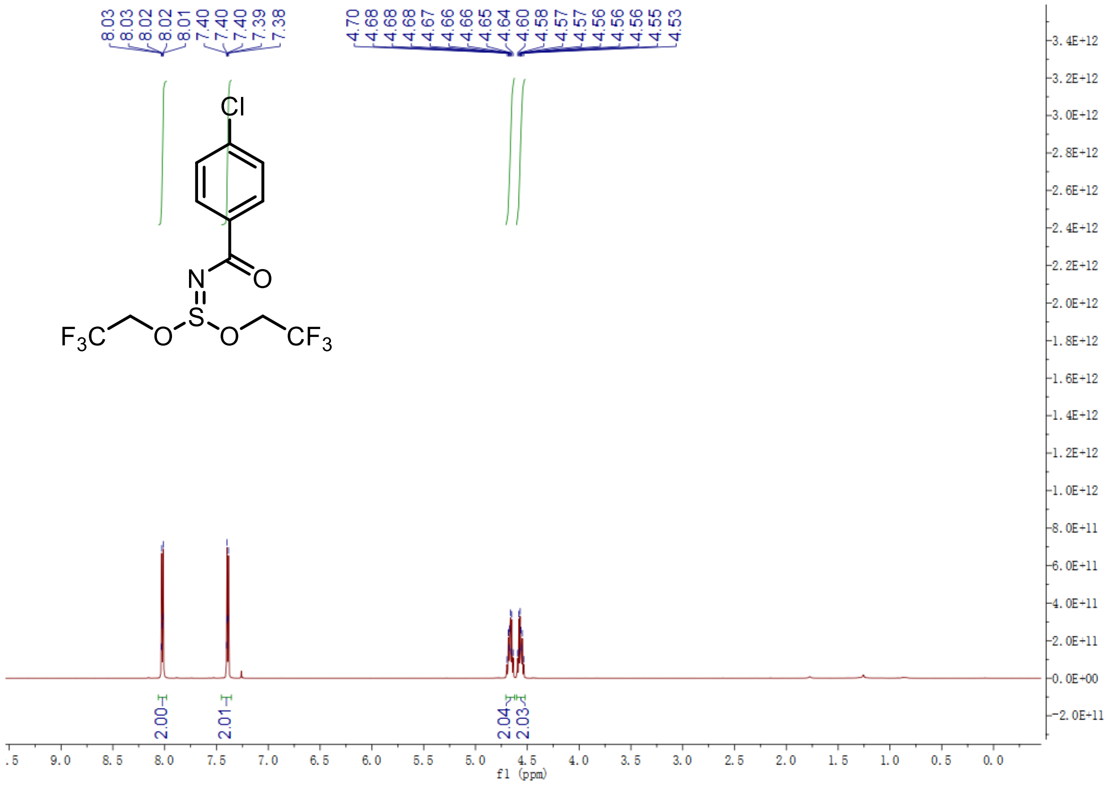


$^{13}$C{$^{1}$H} NMR spectrum of compound **3s** (151 MHz, $CDCl_3$)

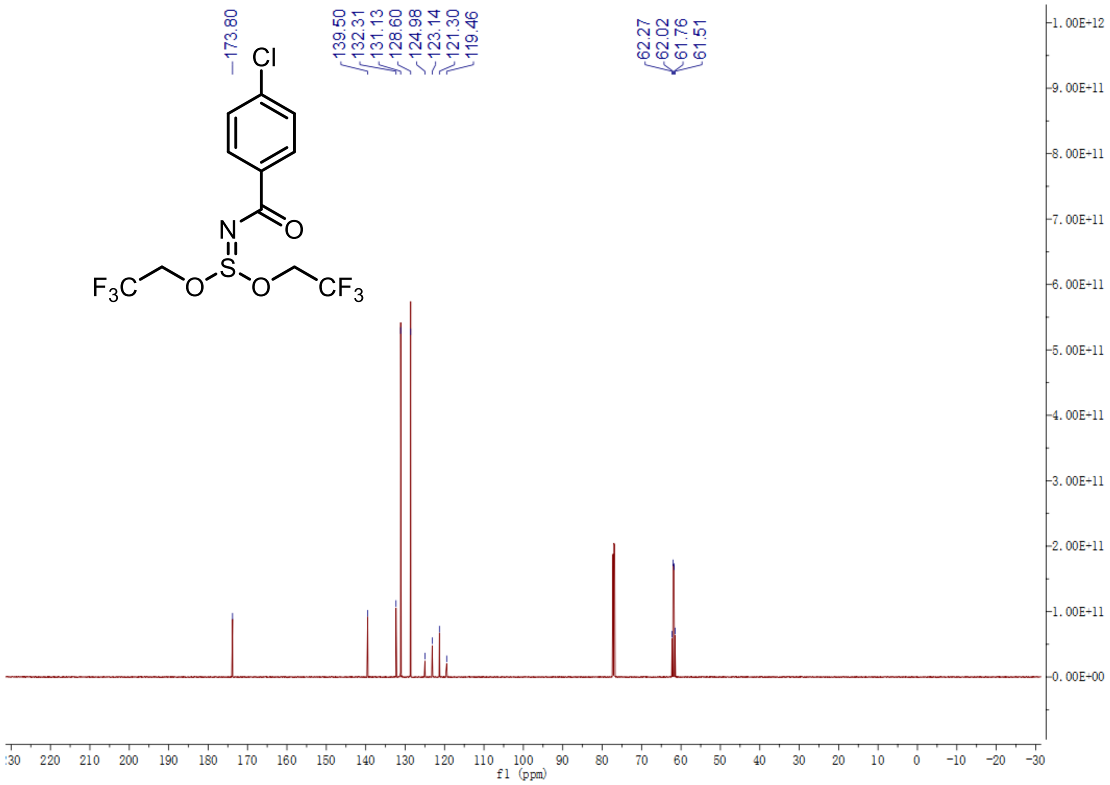

$^{19}$F NMR spectrum of compound **3s** (564 MHz, CDCl$_3$)

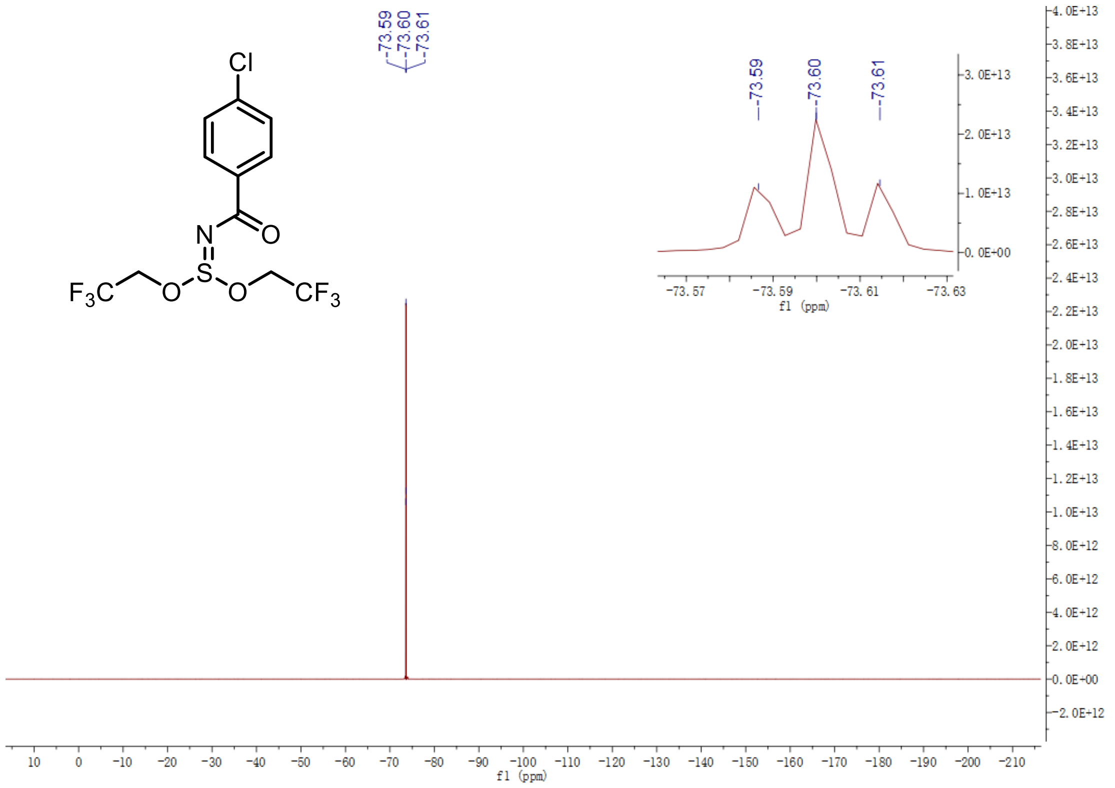


$^{1}$H NMR spectrum of compound **3t** (600 MHz, CDCl$_3$)

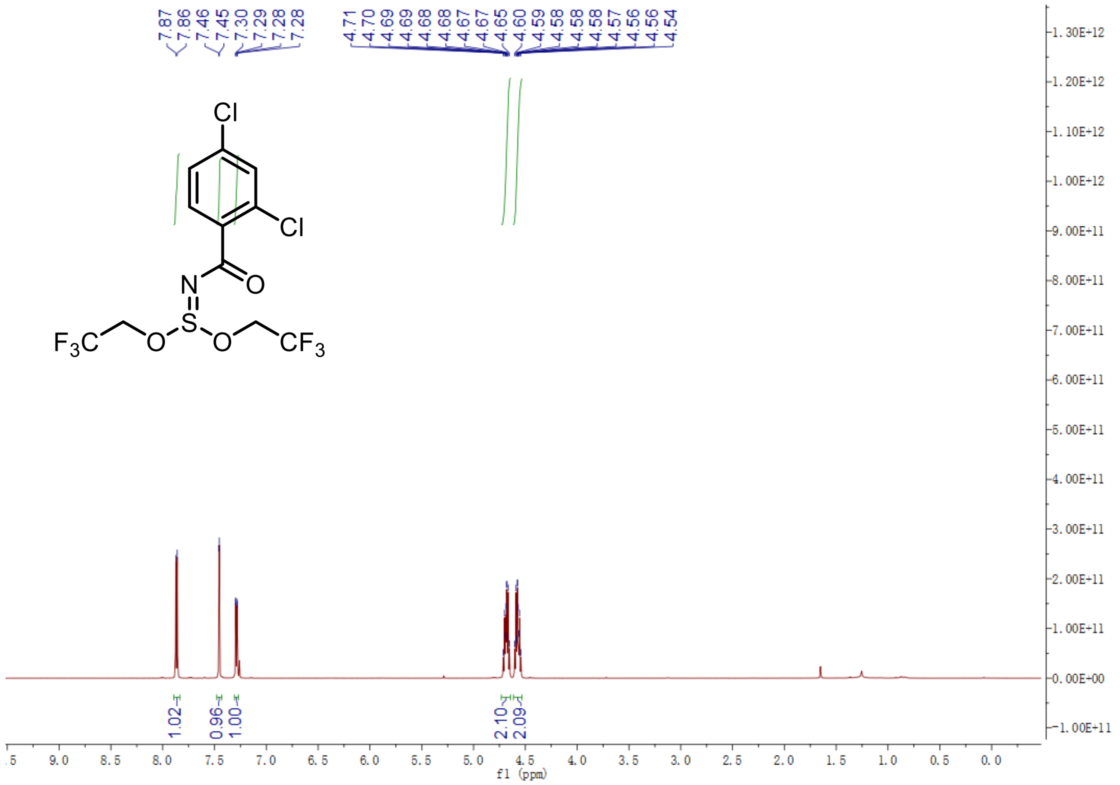

$^{13}C\{^{1}H\}$ NMR spectrum of compound **3t** (151 MHz, $CDCl_3$)

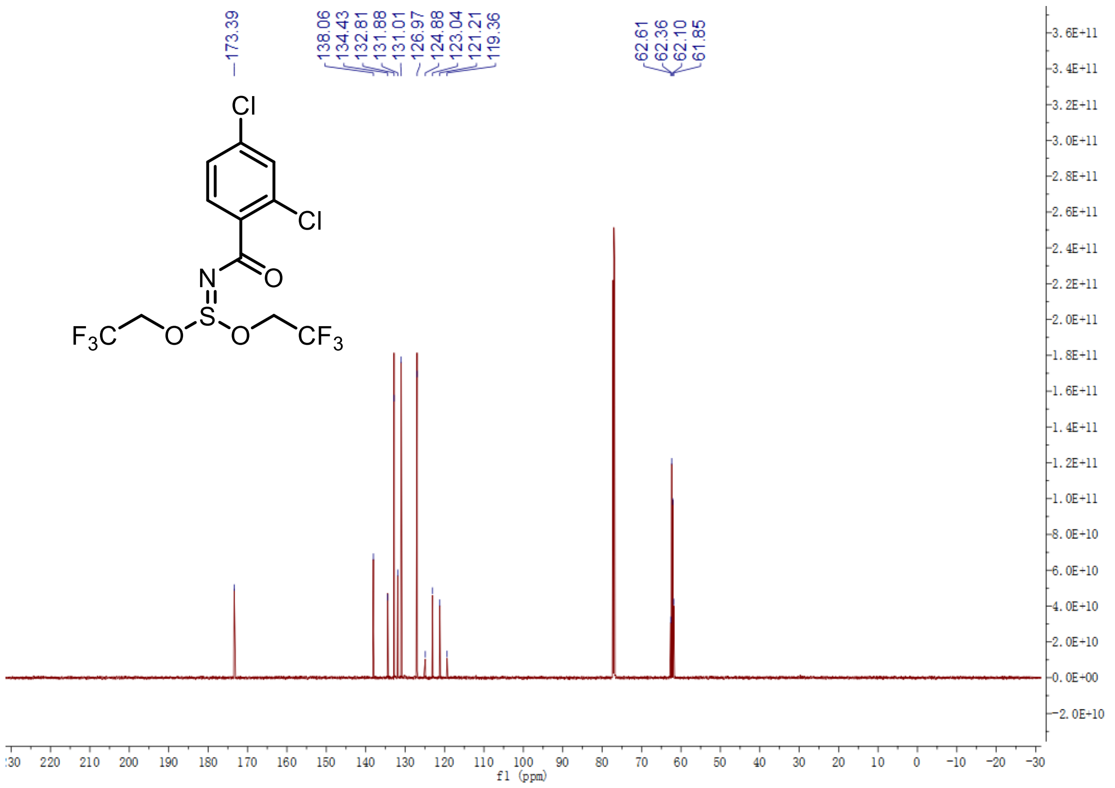


$^{19}F$ NMR spectrum of compound **3t** (564 MHz, $CDCl_3$)

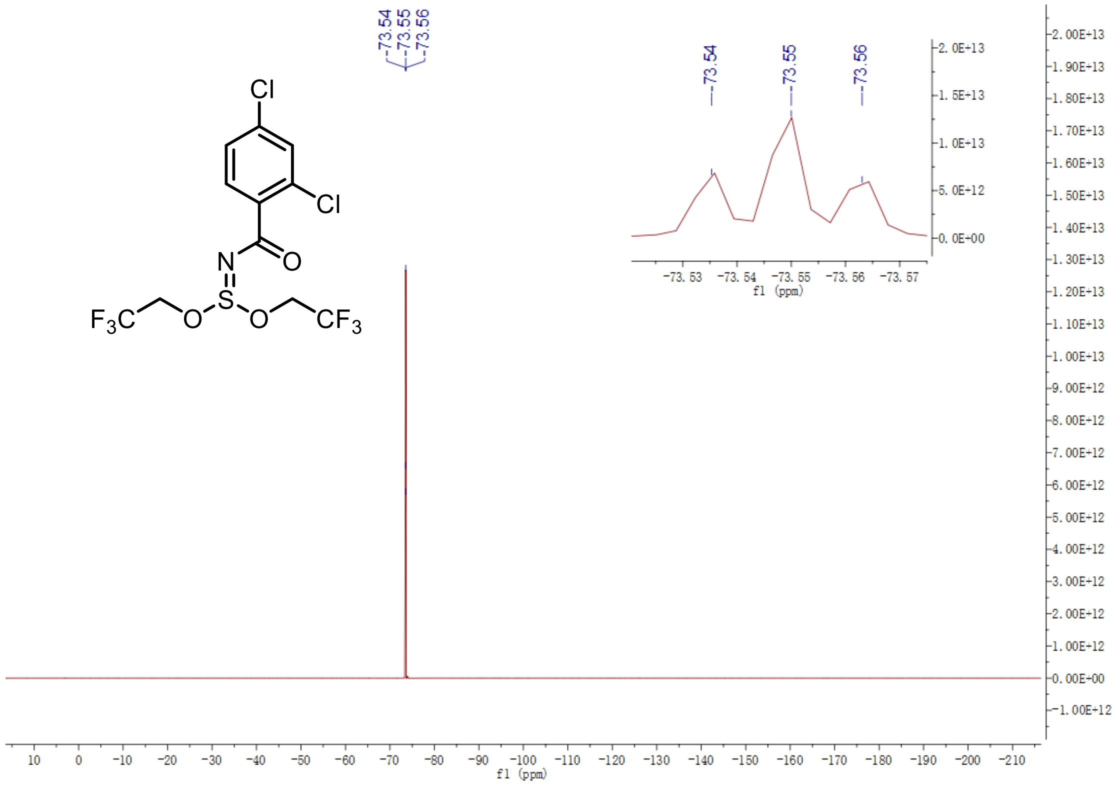

$^{1}$H NMR spectrum of compound **3u** (600 MHz, $CDCl_3$)

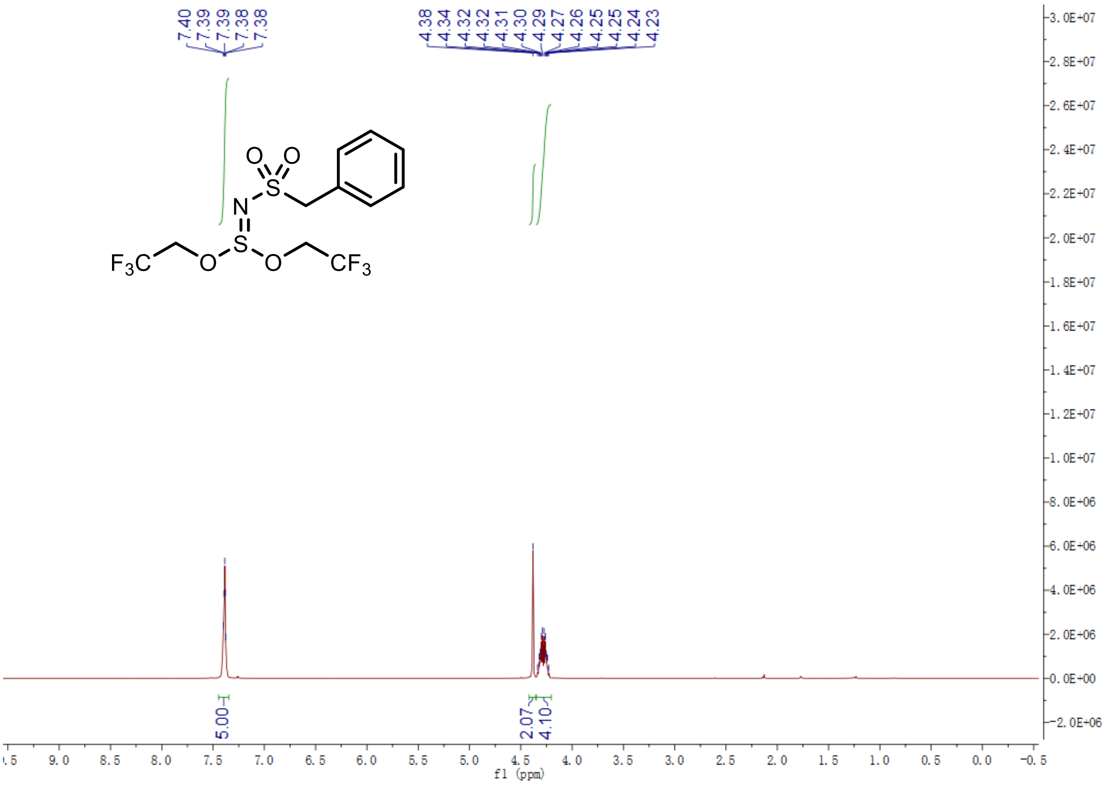


$^{13}$C{$^{1}$H} NMR spectrum of compound **3u** (151 MHz, $CDCl_3$)

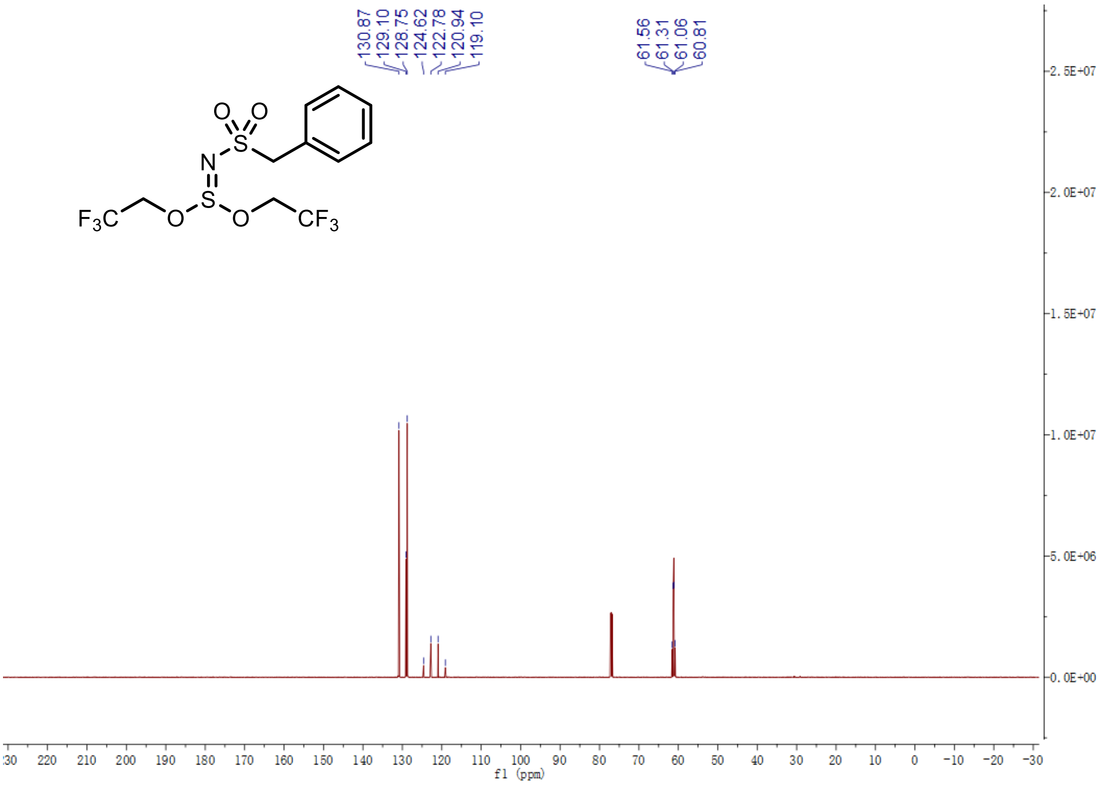

$^{19}$F NMR spectrum of compound **3u** (564 MHz, $CDCl_3$)

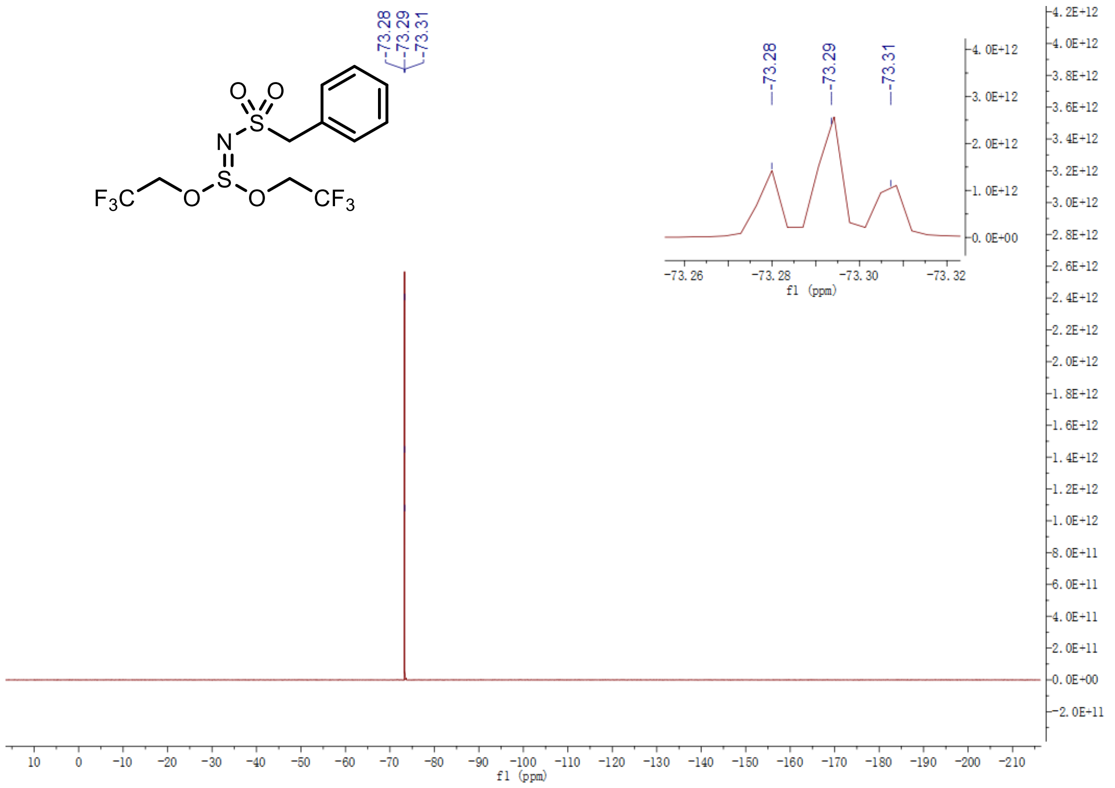

$^{1}$H NMR spectrum of compound **3v** (600 MHz, $CDCl_3$)

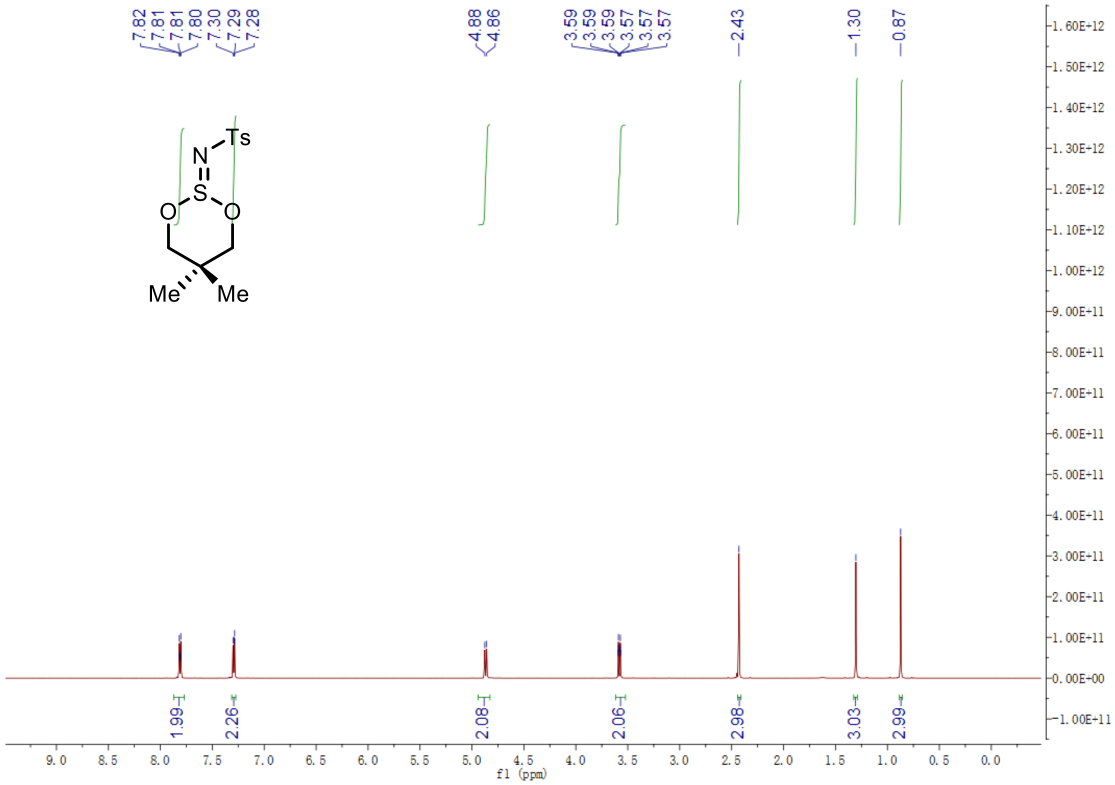

$^{13}C\{^{1}H\}$ NMR spectrum of compound **3v** (151 MHz, $CDCl_3$)

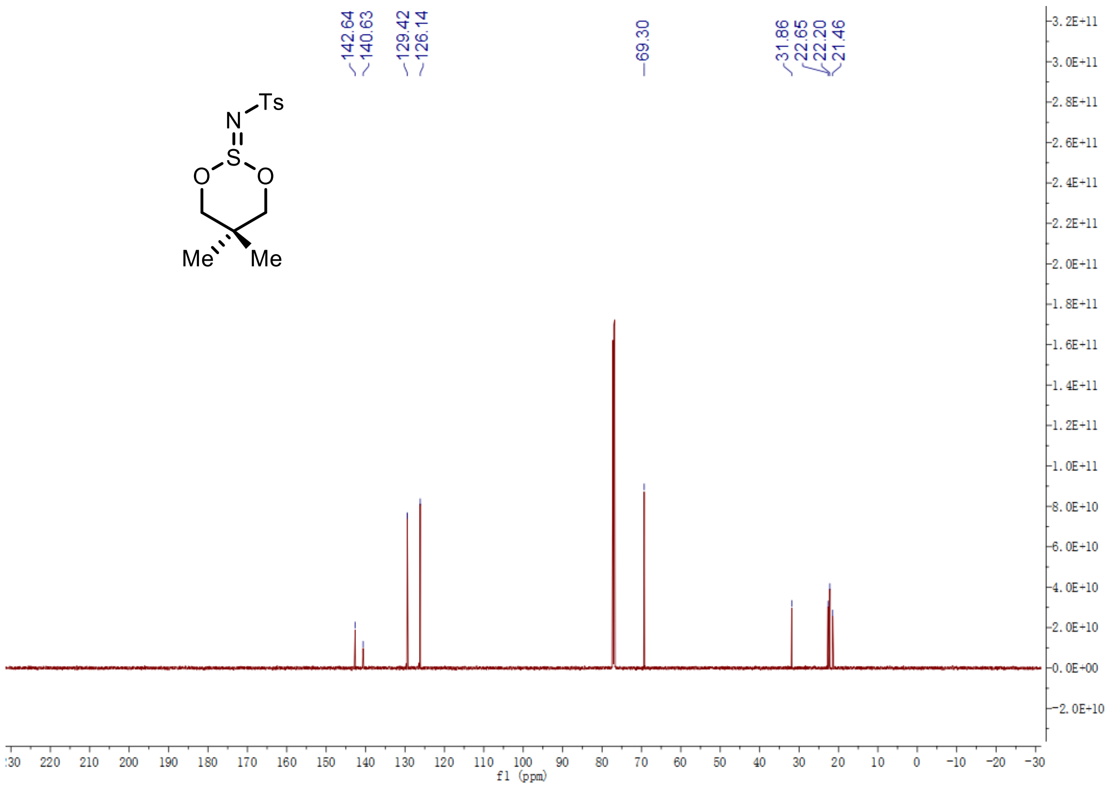

$^{1}H$ NMR spectrum of compound **3w** (600 MHz, $CDCl_3$)

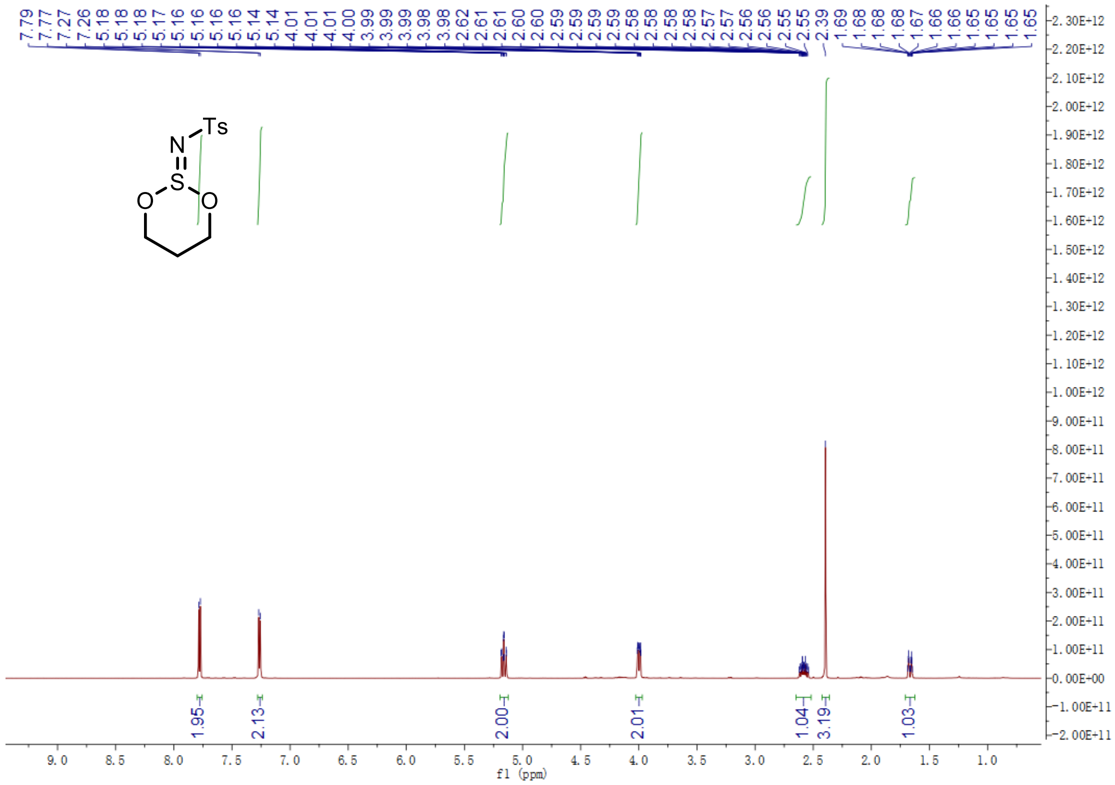

$^{13}C\{^{1}H\}$ NMR spectrum of compound **3w** (151 MHz, $CDCl_3$)

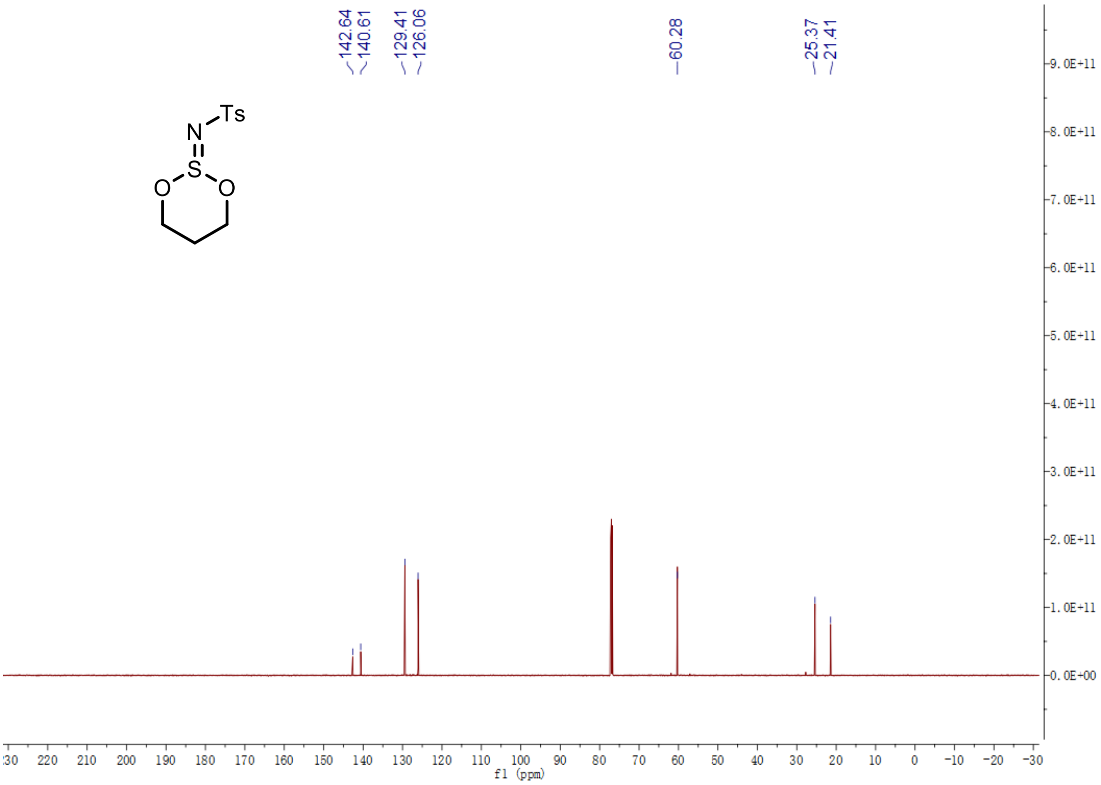

$^{1}H$ NMR spectrum of compound **3x** (400 MHz, $CDCl_3$)

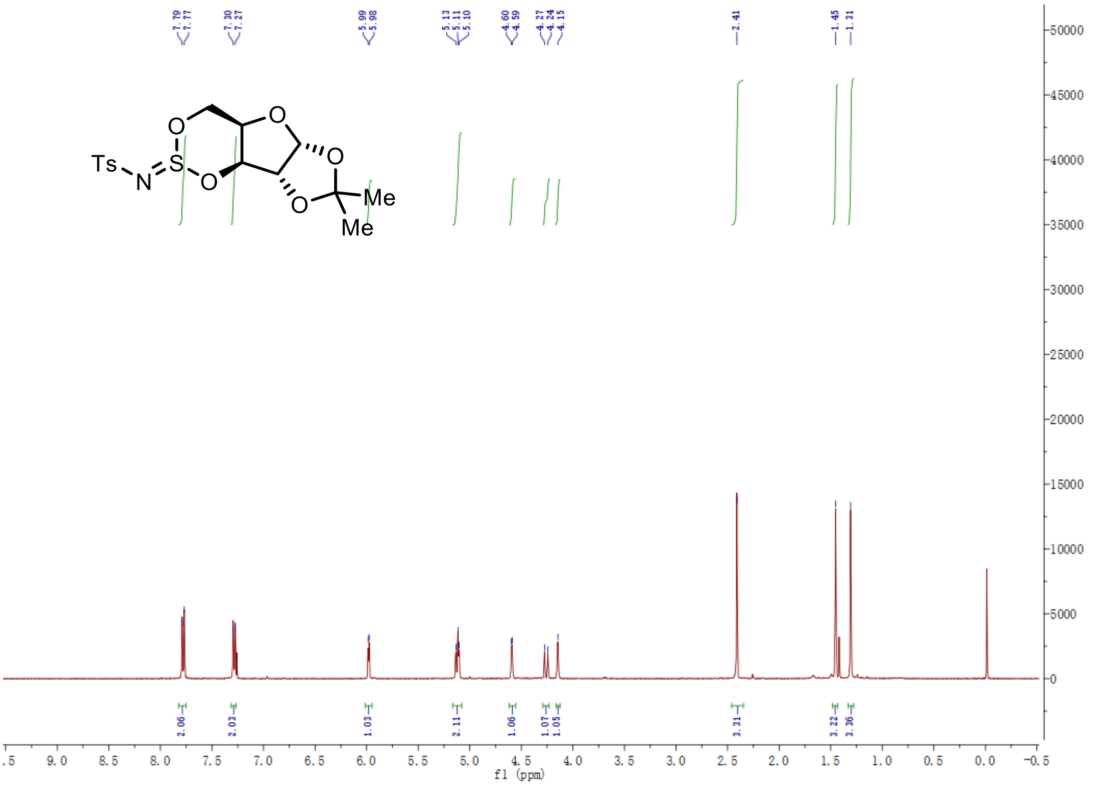

$^{13}C\{^{1}H\}$ NMR spectrum of compound **3x** (101 MHz, $CDCl_3$)

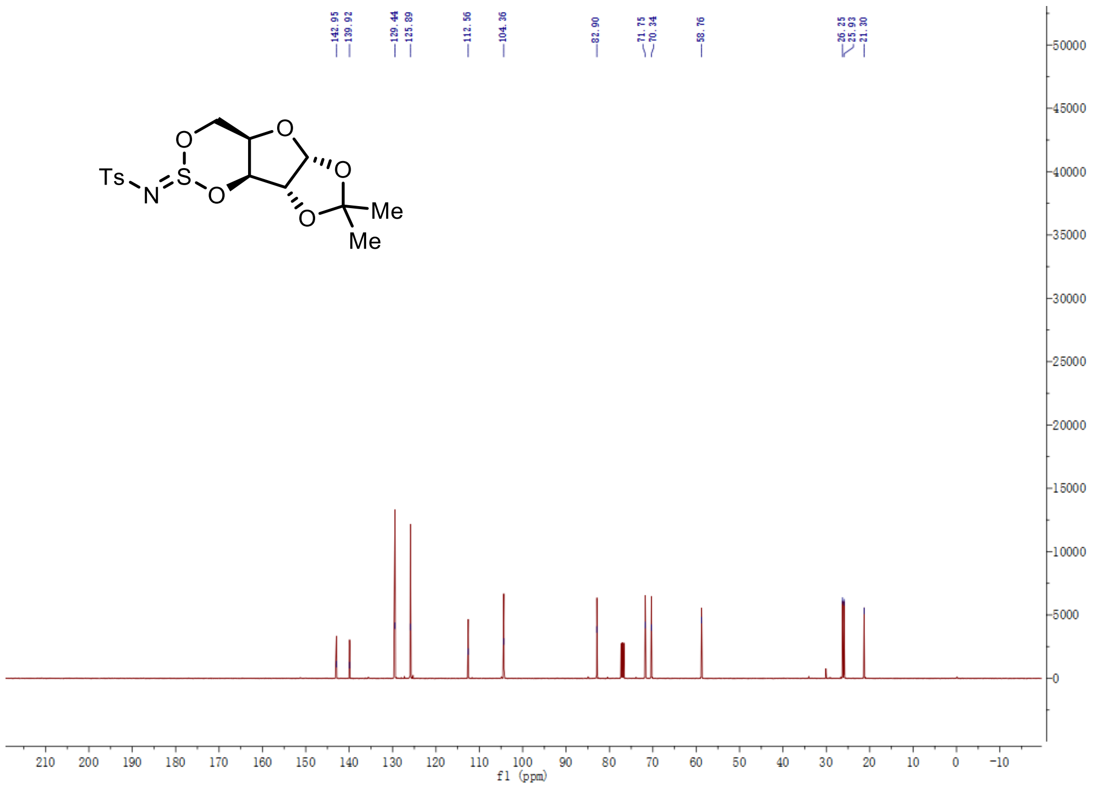

$^{1}H$ NMR spectrum of compound **3y** (400 MHz, $CDCl_3$)

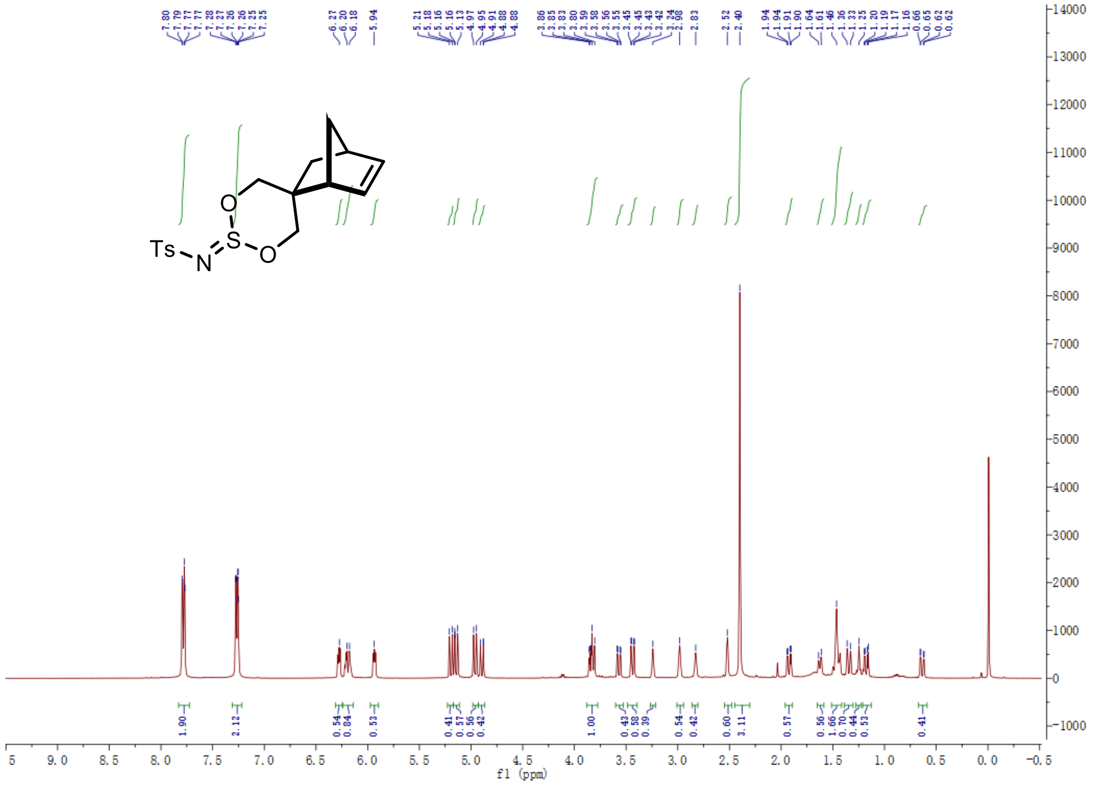

$^{13}C\{^{1}H\}$ NMR spectrum of compound **3y** (101 MHz, $CDCl_3$)

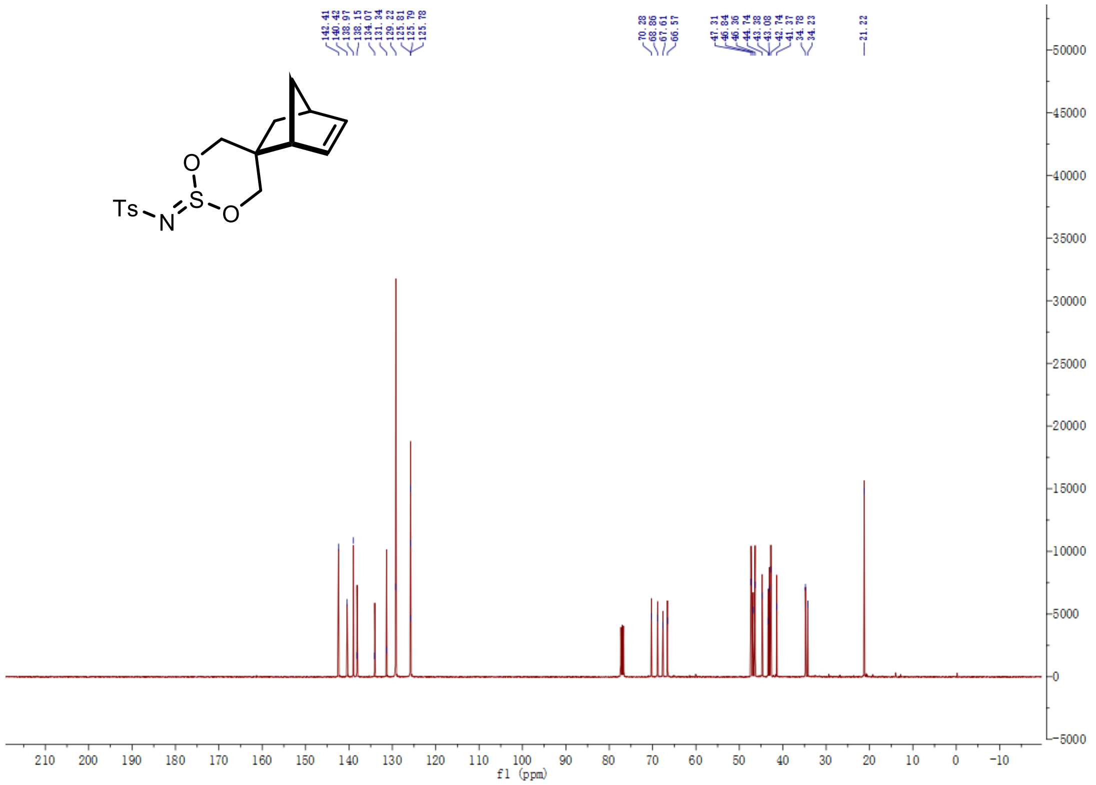

$^{1}H$ NMR spectrum of compound **3z** (400 MHz, $CDCl_3$)

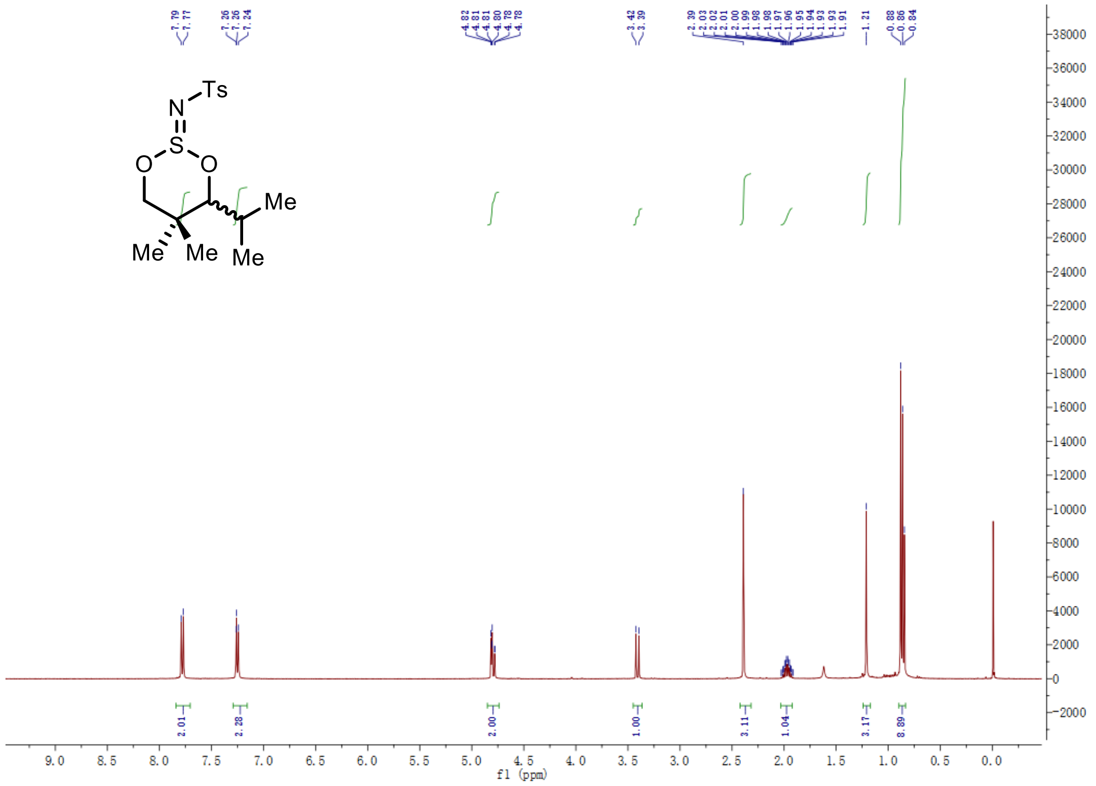

$^{13}C\{^1H\}$ NMR spectrum of compound **3z** (101 MHz, $CDCl_3$)

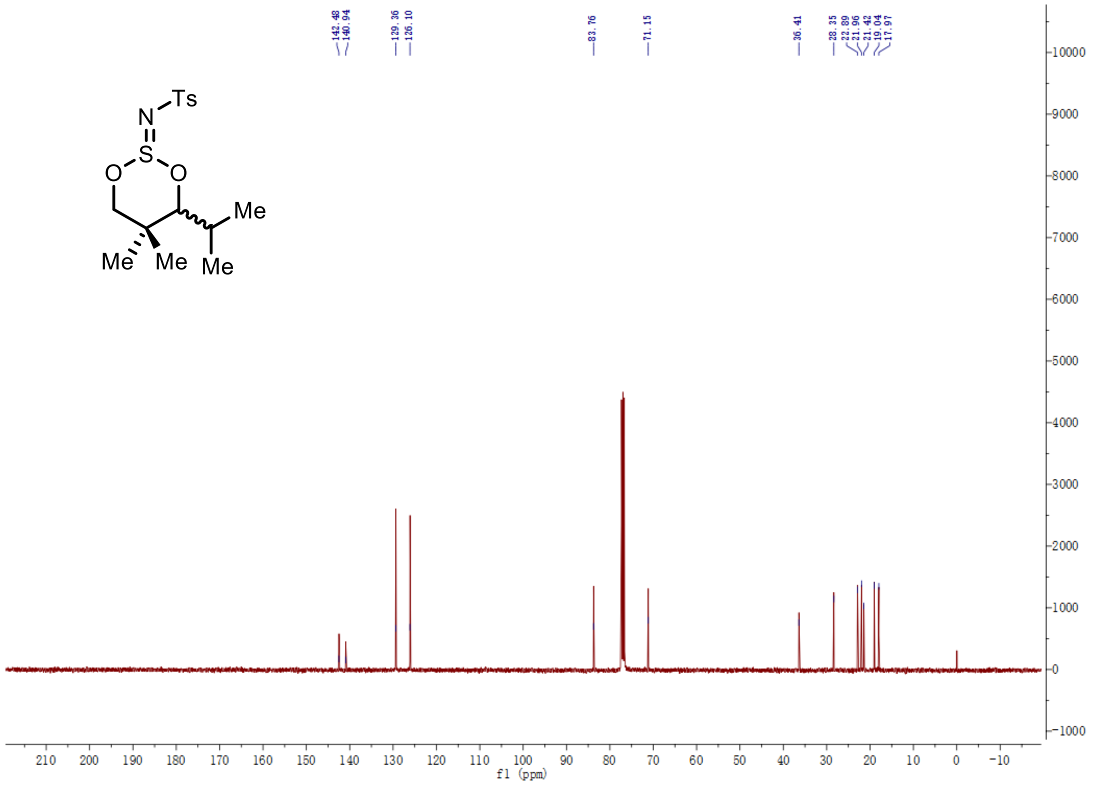

$^1H$ NMR spectrum of compound **4a** (600 MHz, $CDCl_3$)

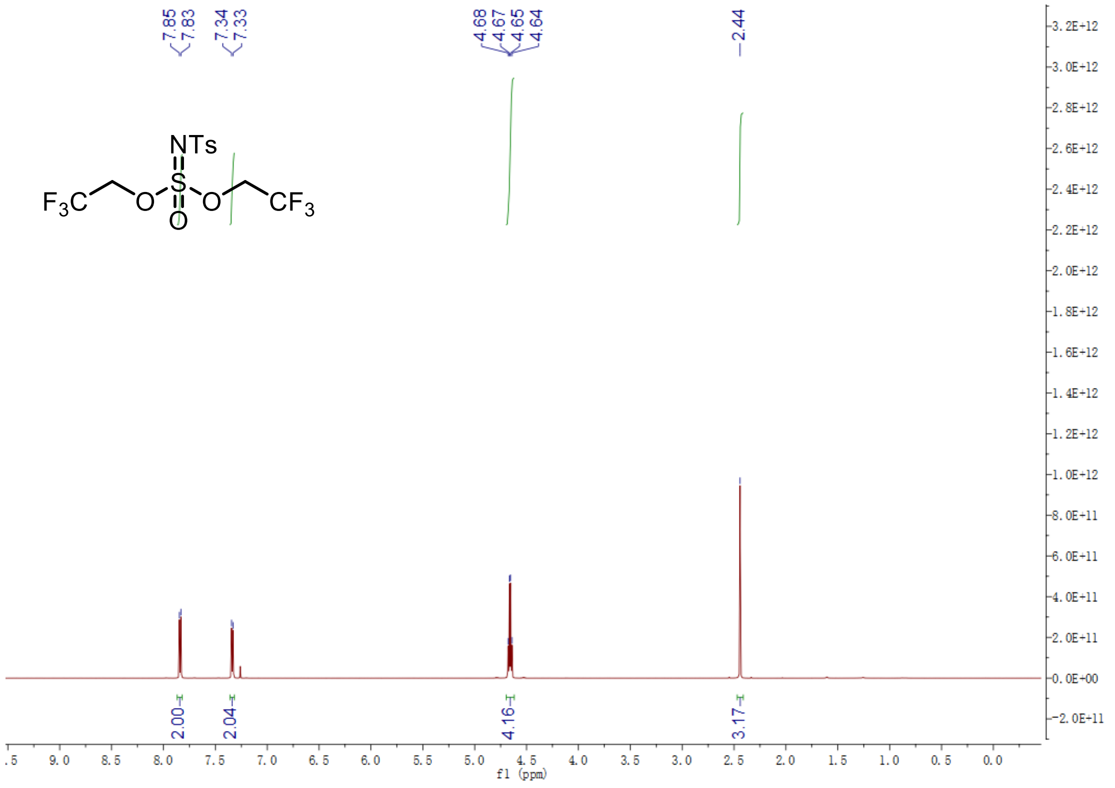

$^{13}C\{^{1}H\}$ NMR spectrum of compound **4a** (151 MHz, $CDCl_3$)

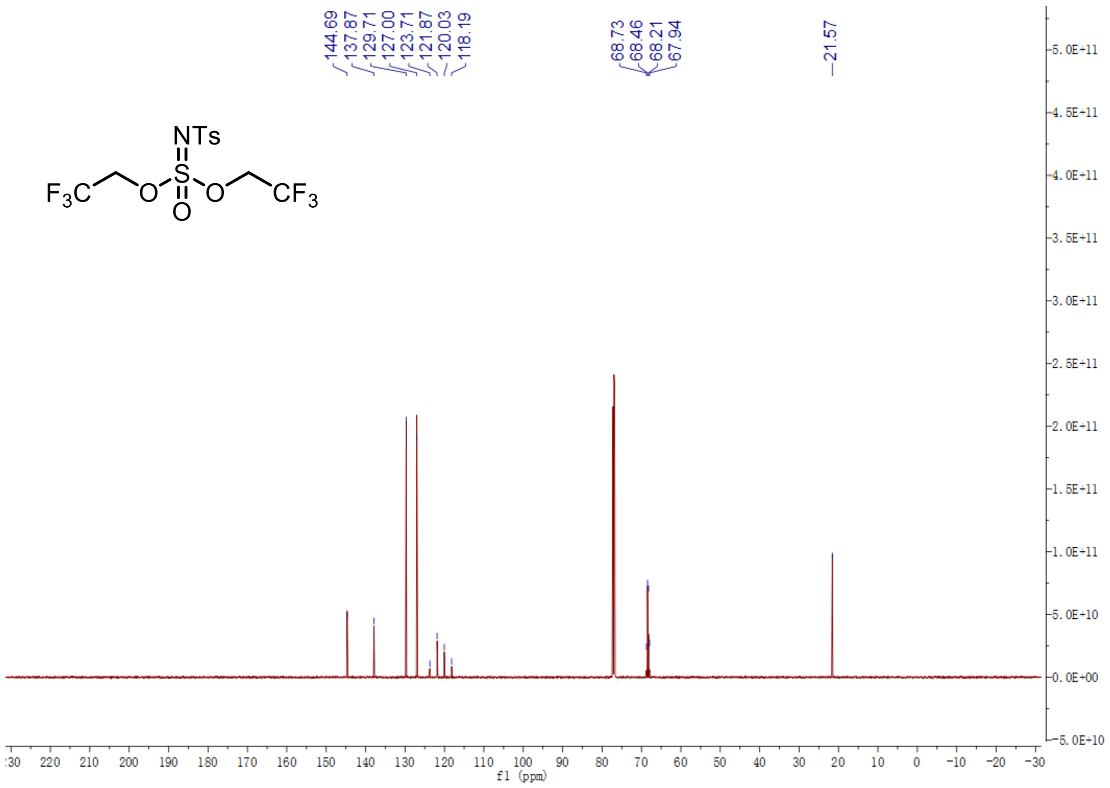


$^{19}F$ NMR spectrum of compound **4a** (564 MHz, $CDCl_3$)

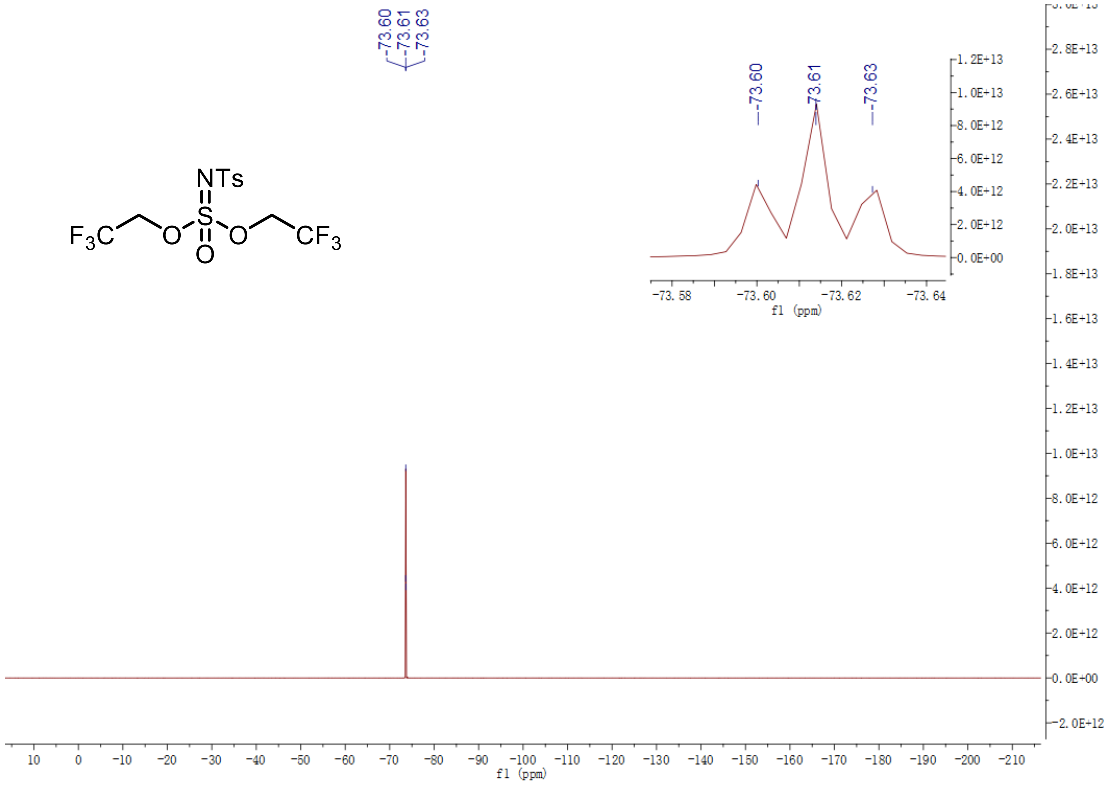

$^{1}$H NMR spectrum of compound **4b** (600 MHz, $CDCl_3$)

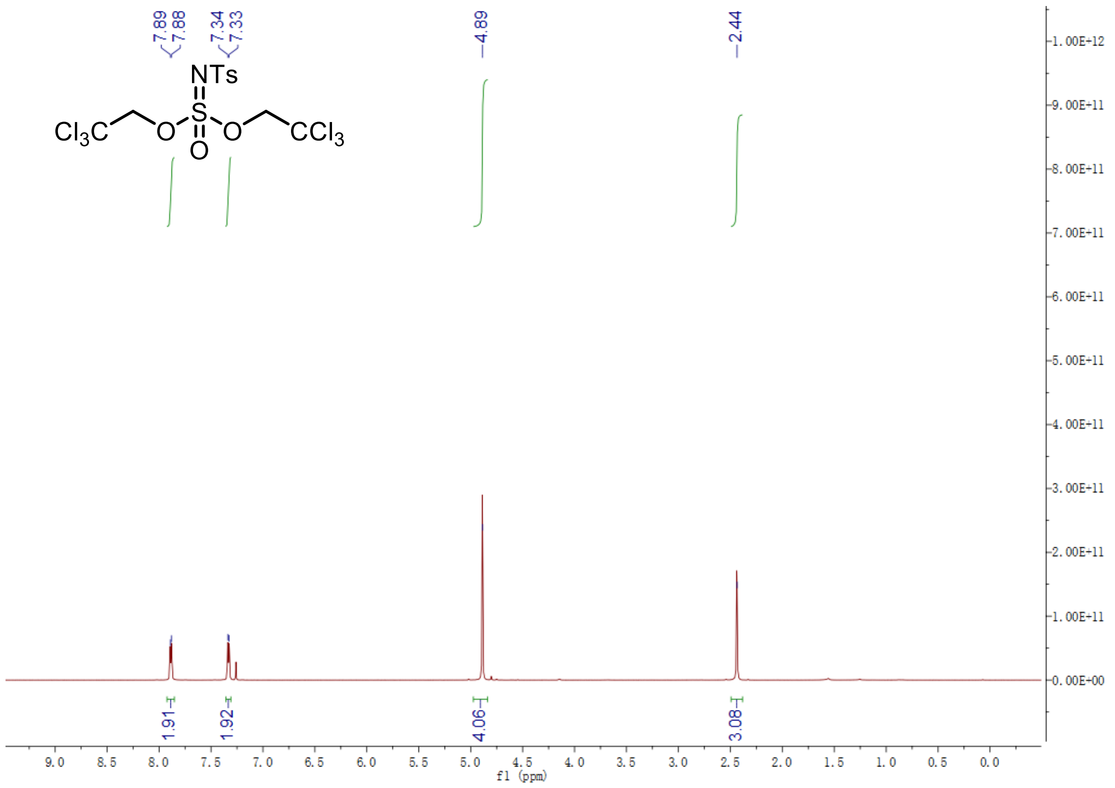


$^{13}$C{$^{1}$H} NMR spectrum of compound **4b** (151 MHz, $CDCl_3$)

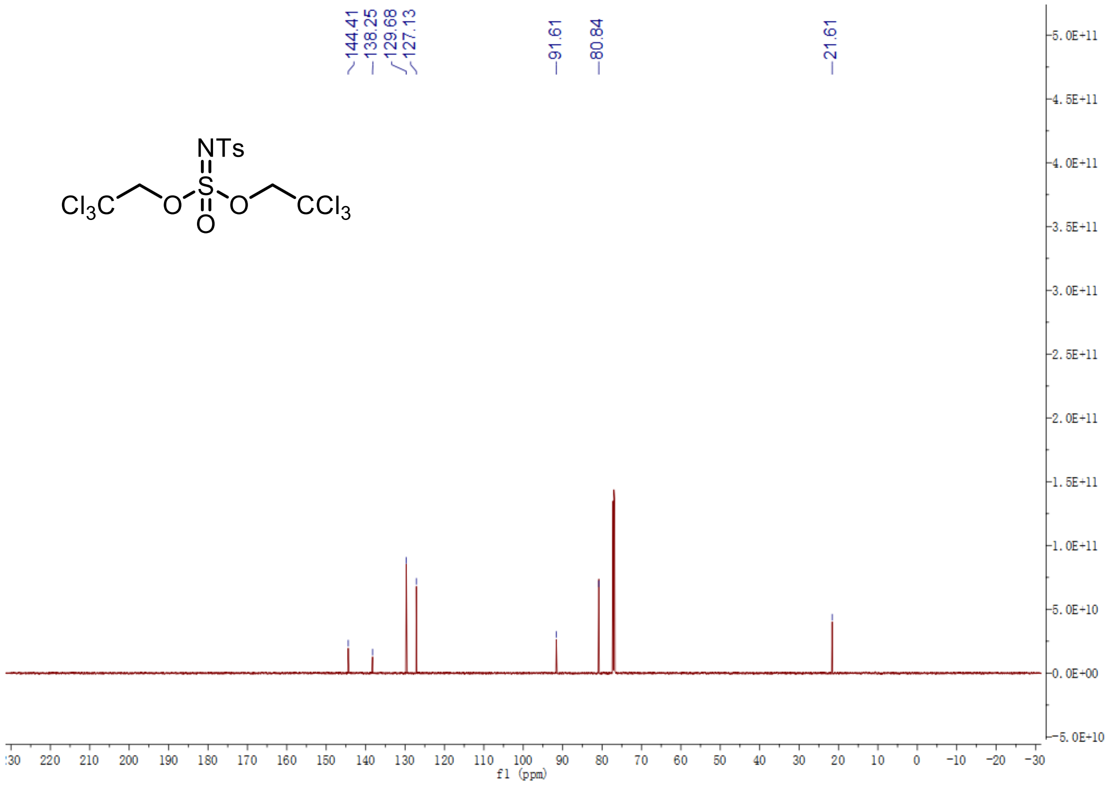

$^{1}$H NMR spectrum of compound **4c’** (600 MHz, $CDCl_3$)

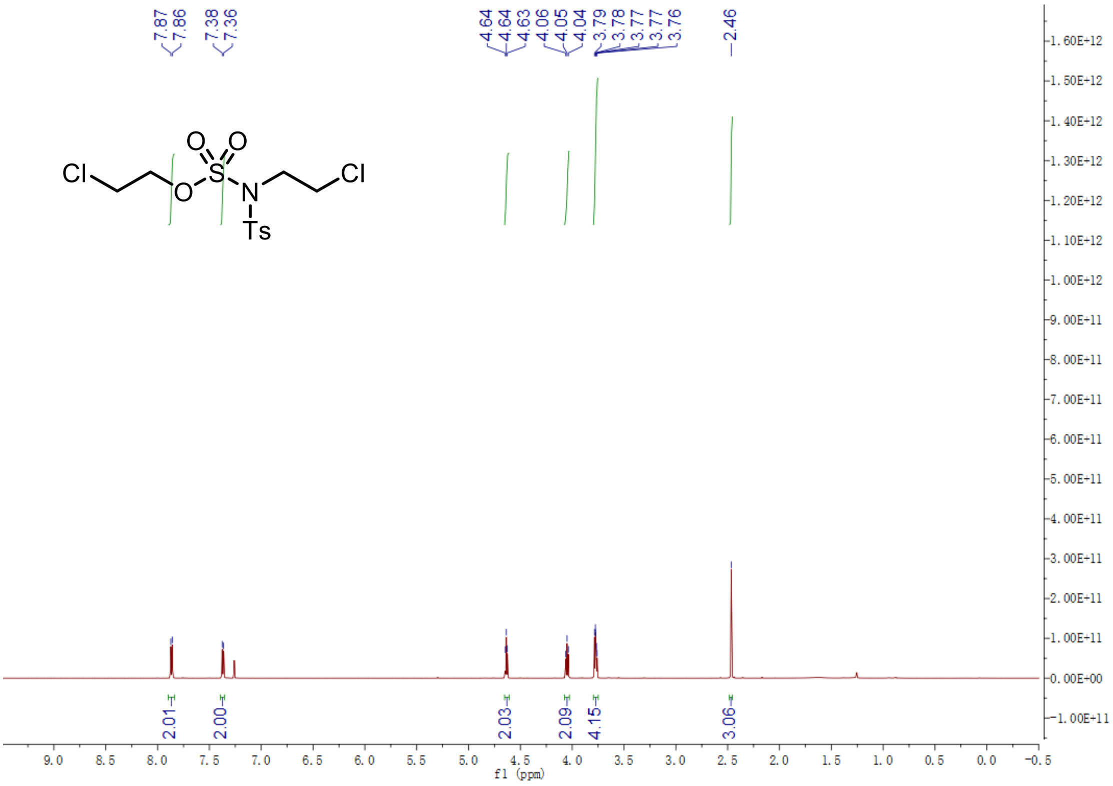


$^{13}$C{$^{1}$H} NMR spectrum of compound **4c’** (151 MHz, $CDCl_3$)

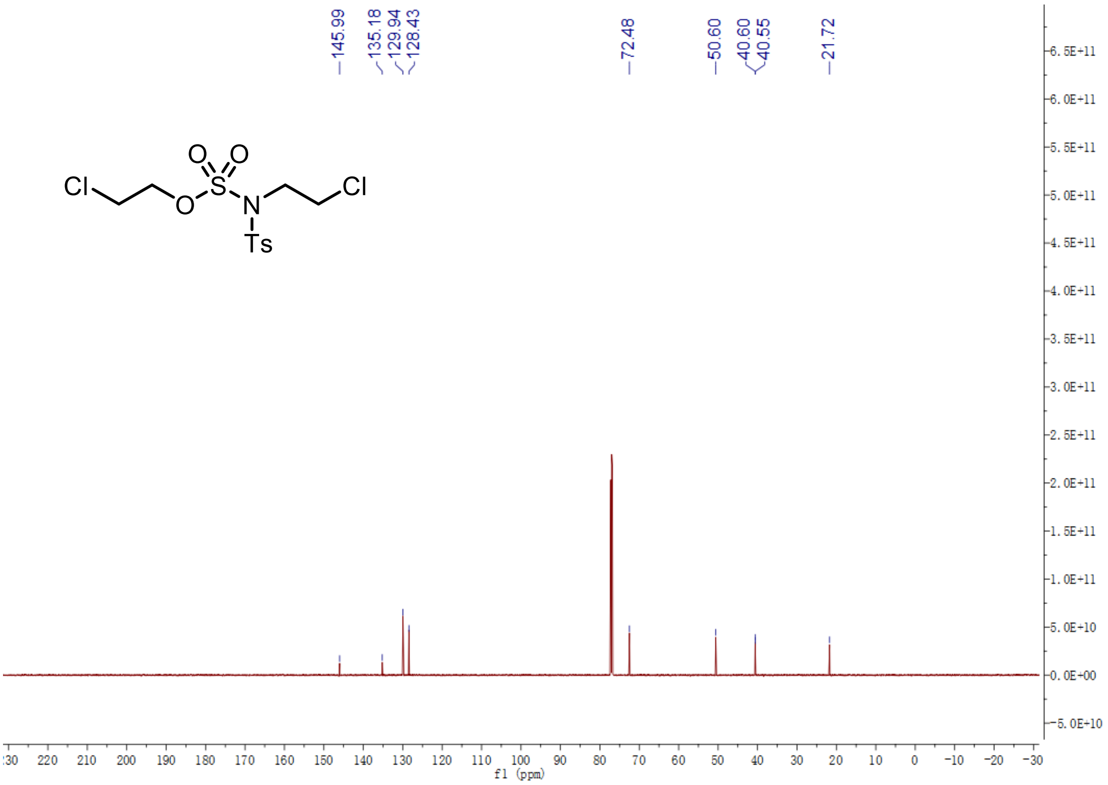

$^1$H NMR spectrum of compound **4d'** (600 MHz, $CDCl_3$)

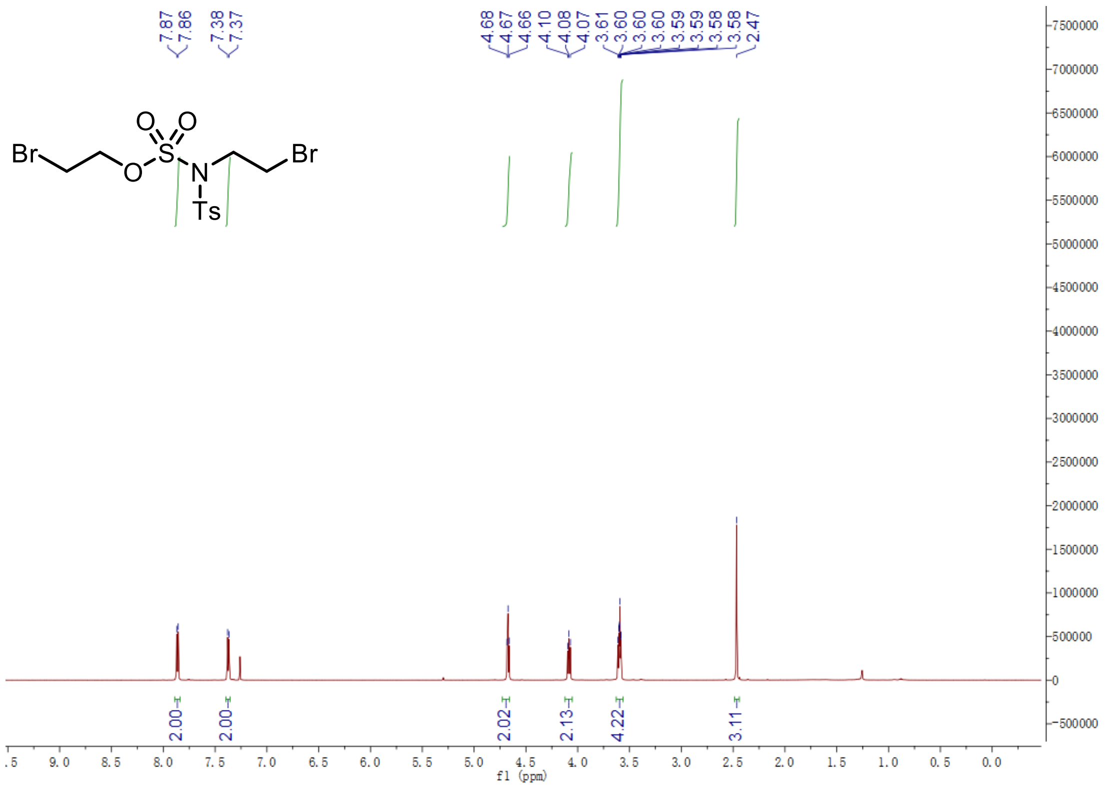

$^{13}$C{$^1$H} NMR spectrum of compound **4d'** (151 MHz, $CDCl_3$)

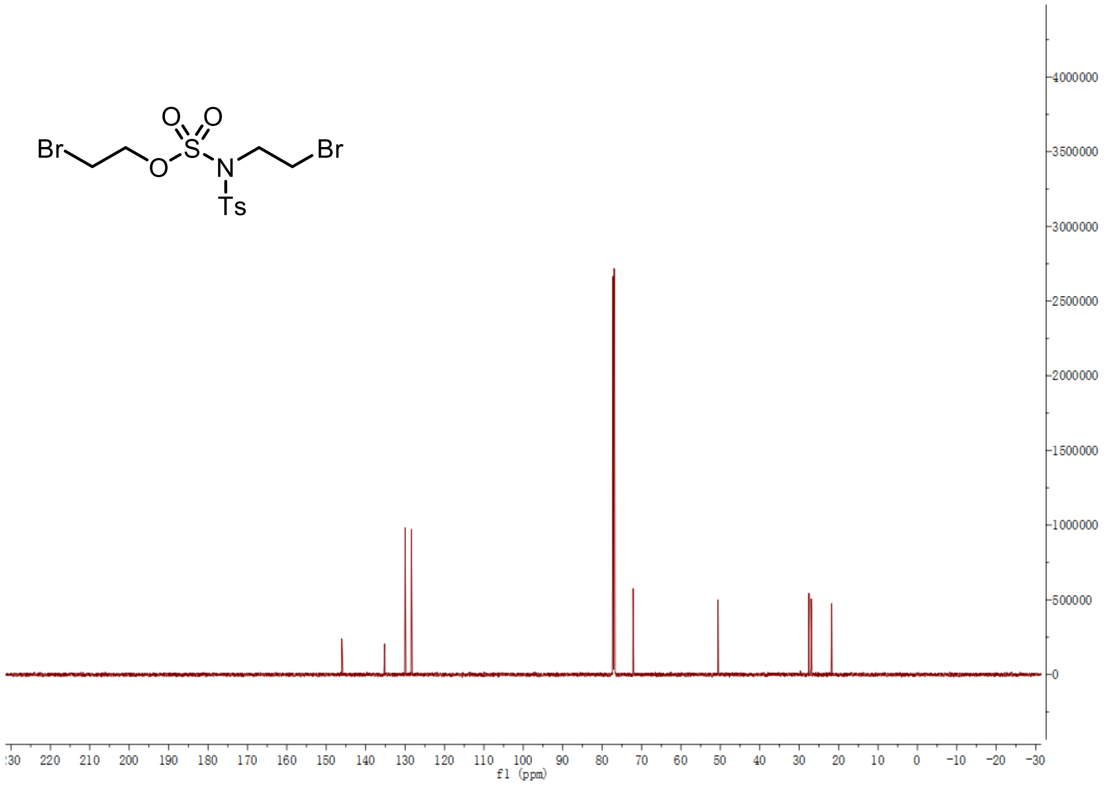

$^{1}H$ NMR spectrum of compound **4f** (600 MHz, $CDCl_3$)

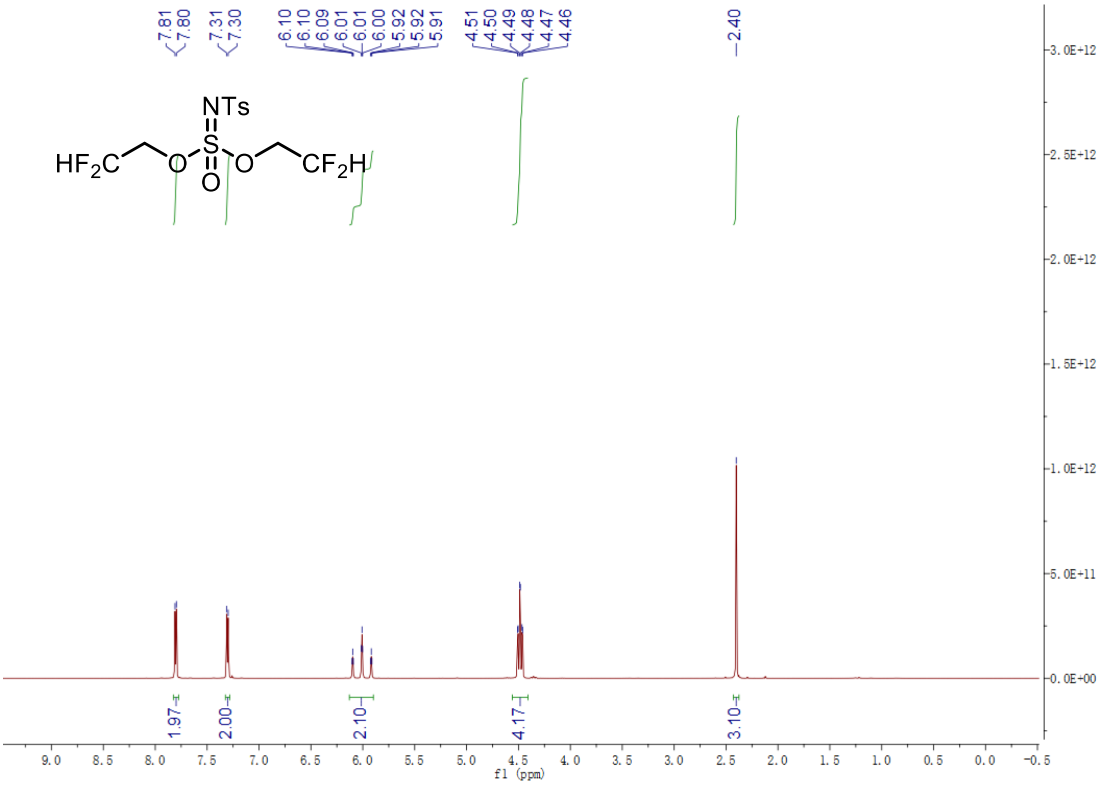


$^{13}C\{^{1}H\}$ NMR spectrum of compound **4f** (151 MHz, $CDCl_3$)

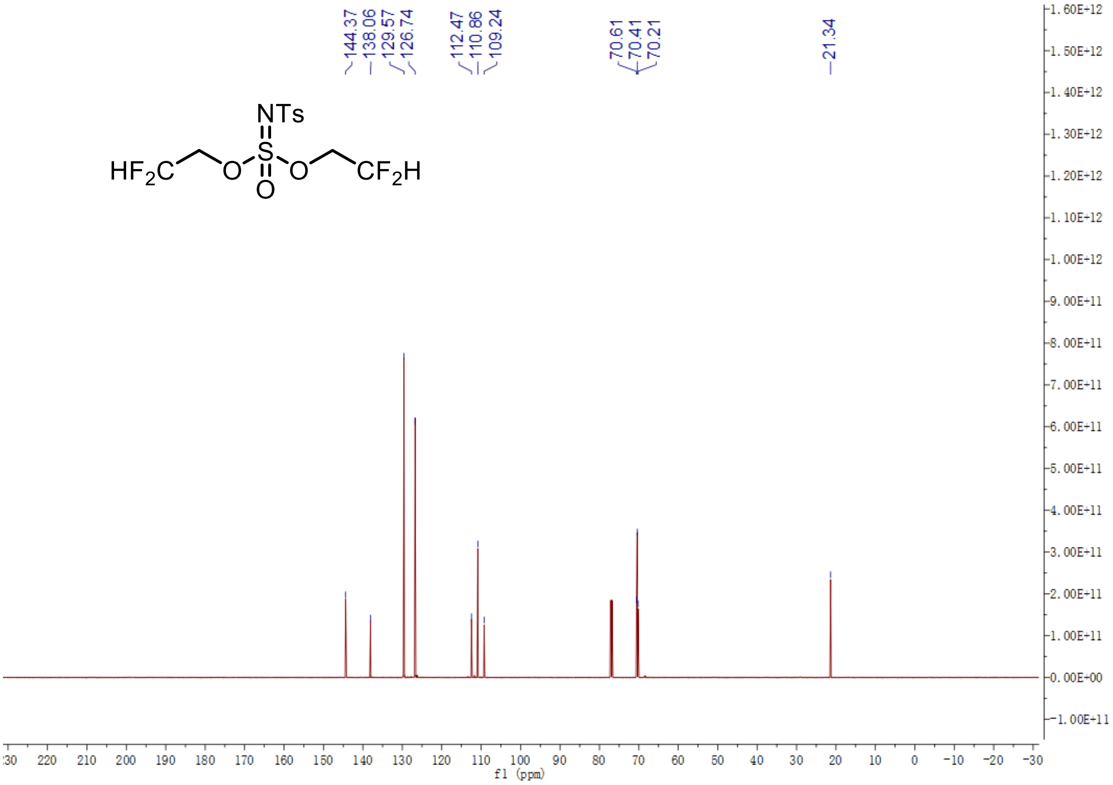

$^{19}$F NMR spectrum of compound **4f** (564 MHz, CDCl$_3$)

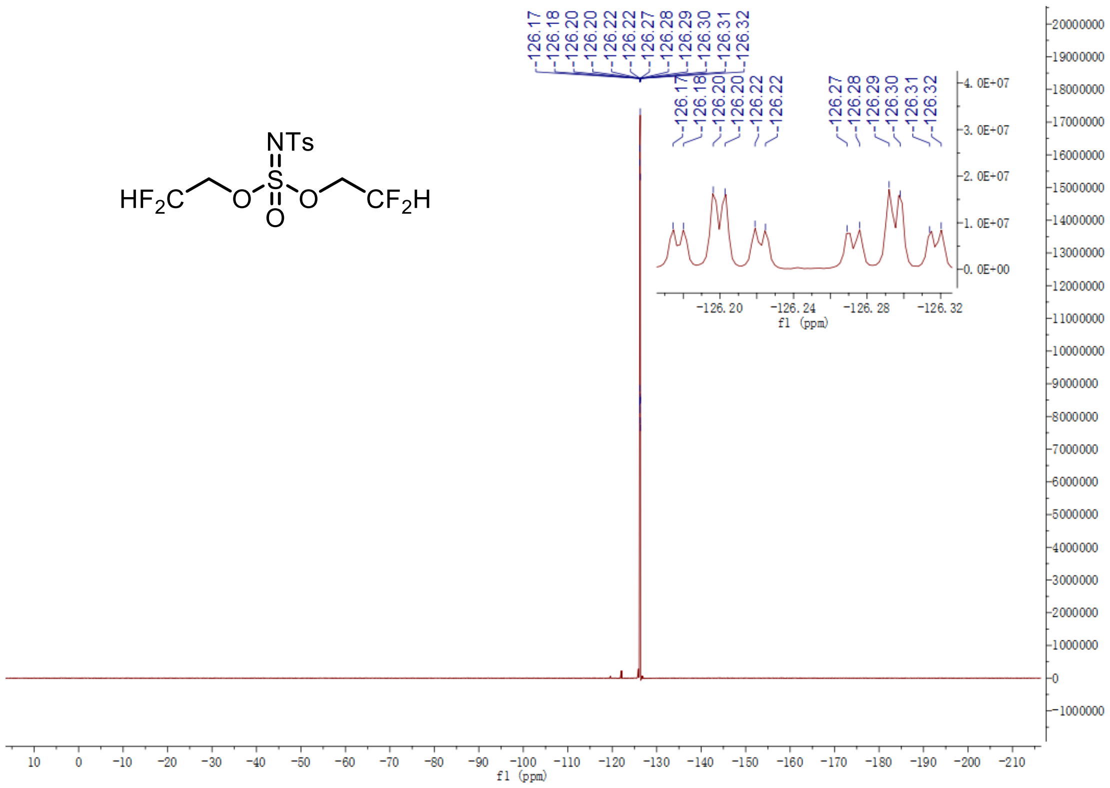


$^{1}$H NMR spectrum of compound **4g** (600 MHz, CDCl$_3$)

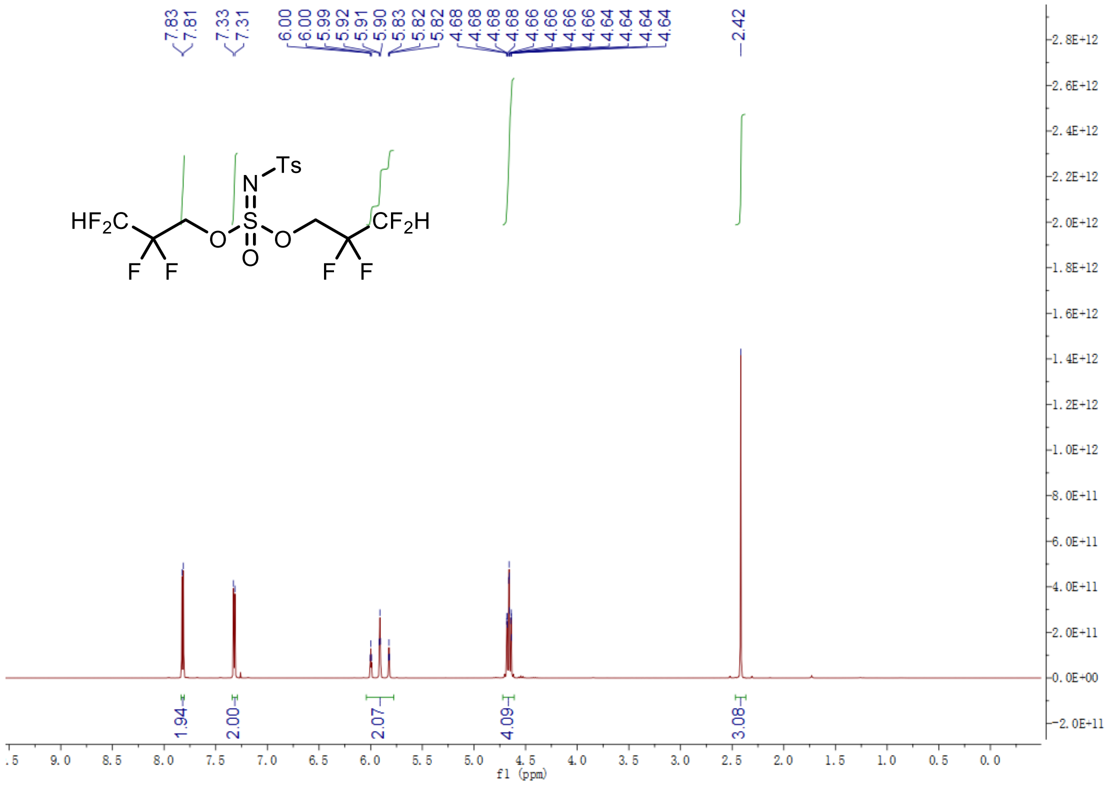

$^{13}C\{^{1}H\}$ NMR spectrum of compound **4g** (151 MHz, $CDCl_3$)

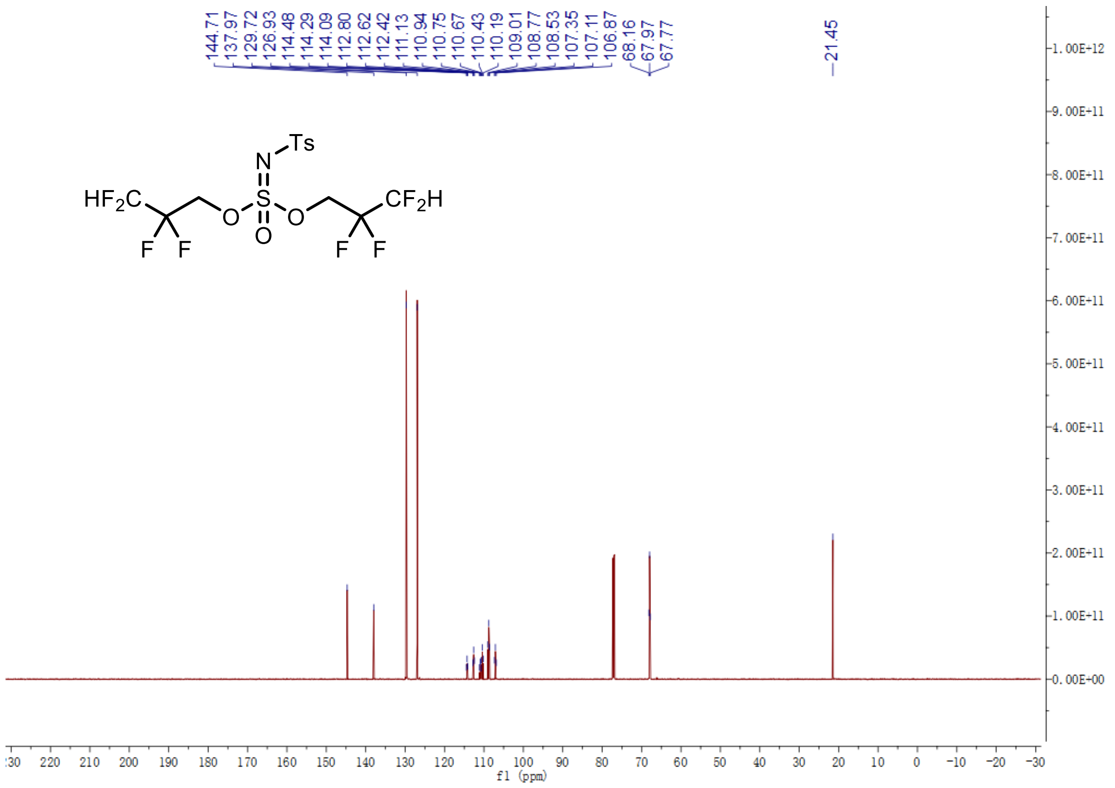

$^{19}F$ NMR spectrum of compound **4g** (564 MHz, $CDCl_3$)

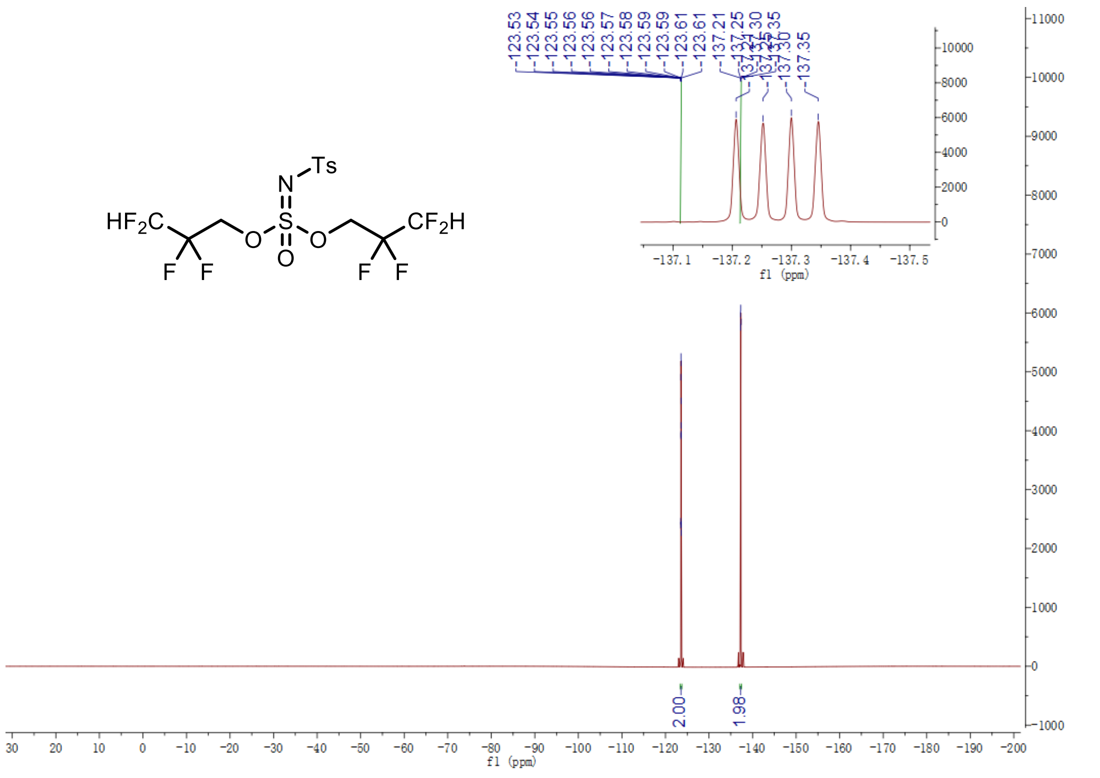

$^{1}$H NMR spectrum of compound **4h** (600 MHz, $CDCl_3$)

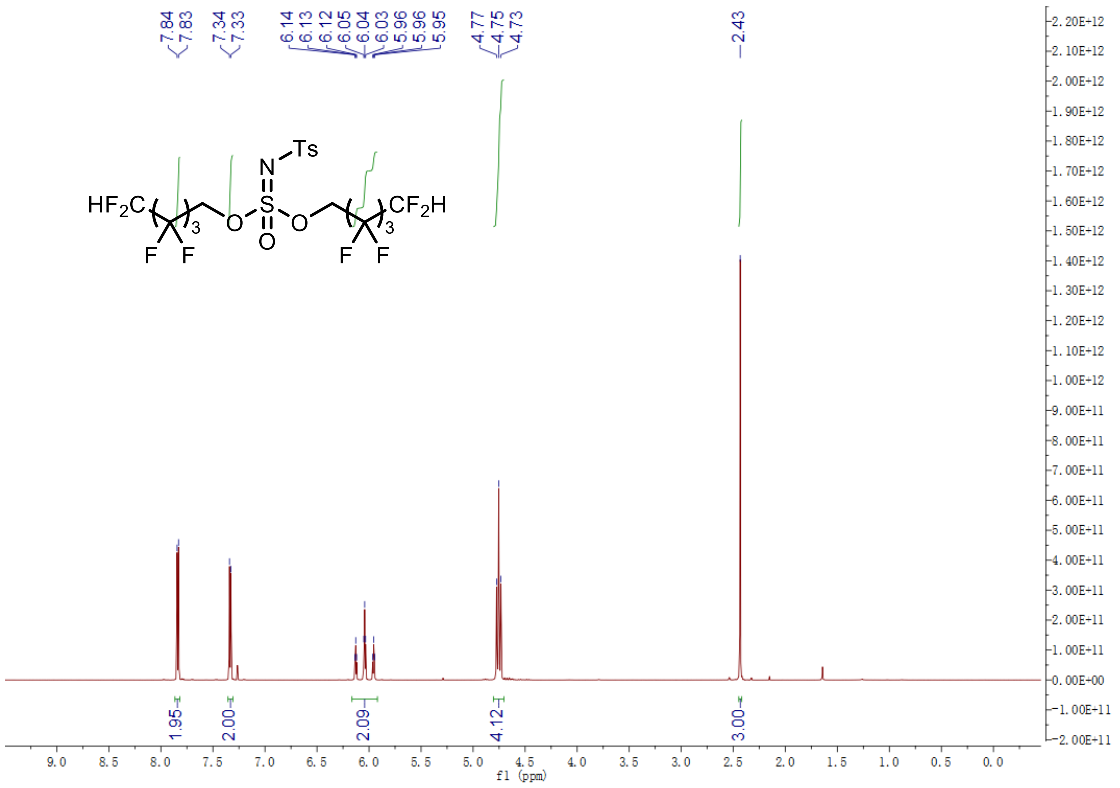

$^{13}$C{$^{1}$H} NMR spectrum of compound **4h** (151 MHz, $CDCl_3$)

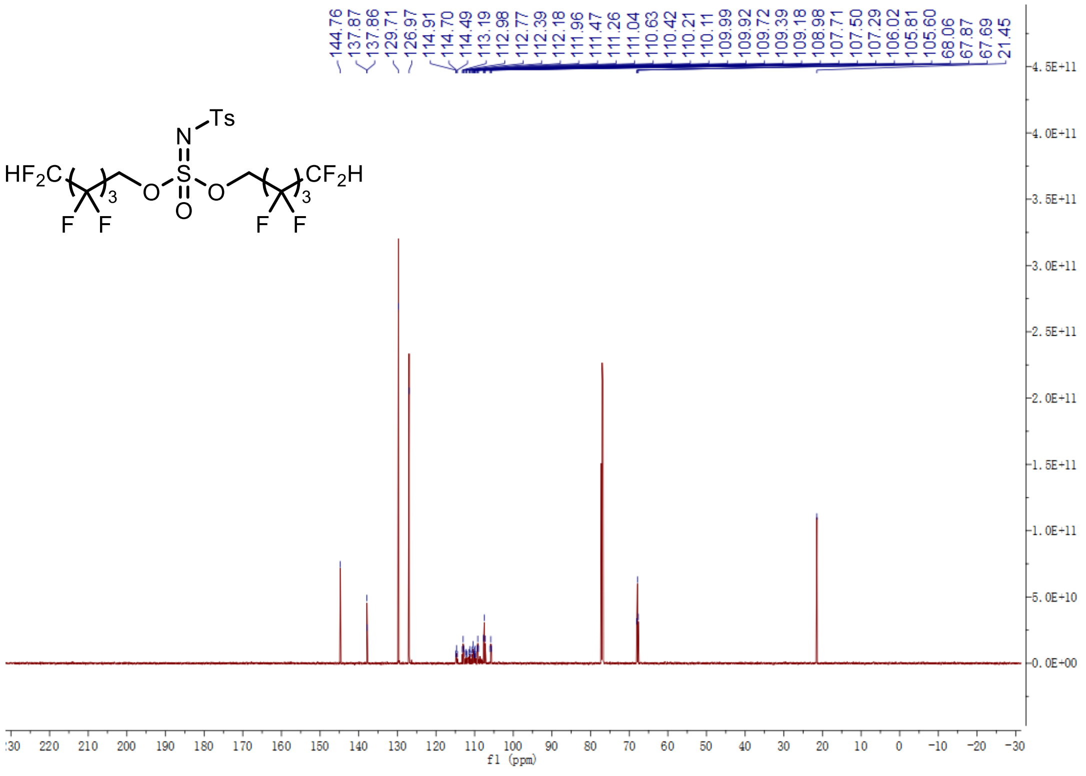

$^{19}$F NMR spectrum of compound **4h** (564 MHz, $CDCl_3$)

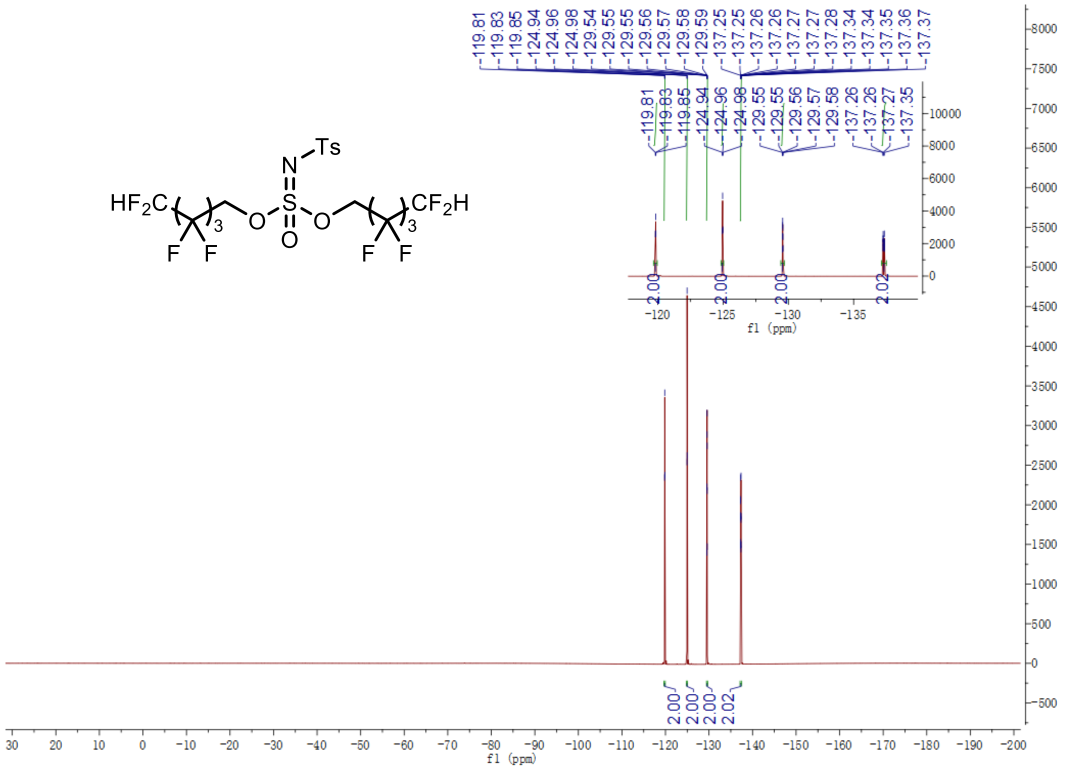

$^{1}$H NMR spectrum of compound **4i’** (600 MHz, $CDCl_3$)

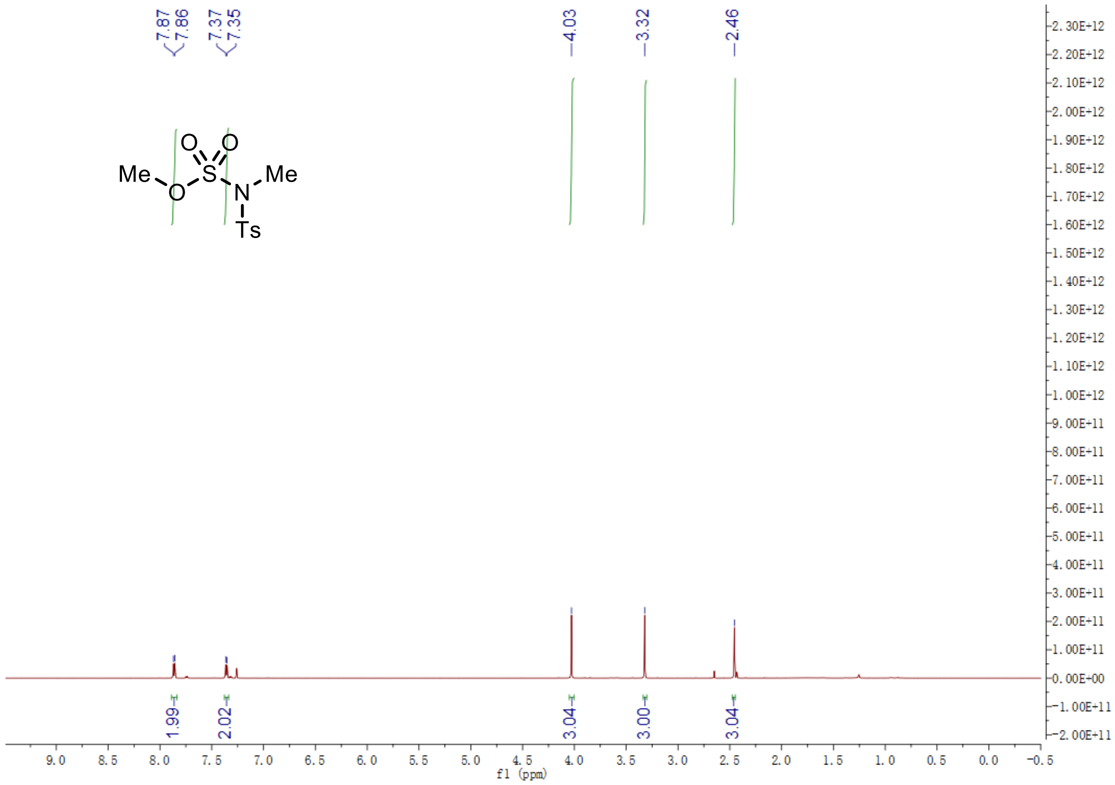

$^{13}$C{$^{1}$H} NMR spectrum of compound **4i’** (151 MHz, $CDCl_3$)

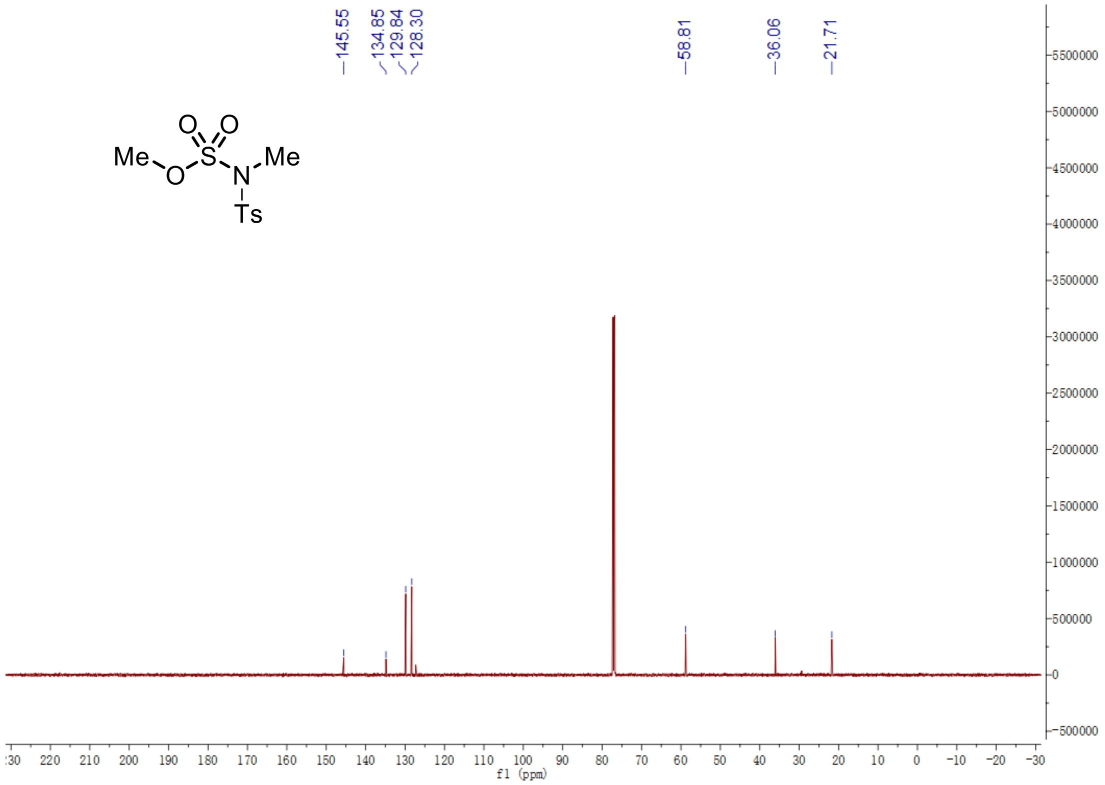


$^{1}$H NMR spectrum of compound **4j’** (600 MHz, $CDCl_3$)

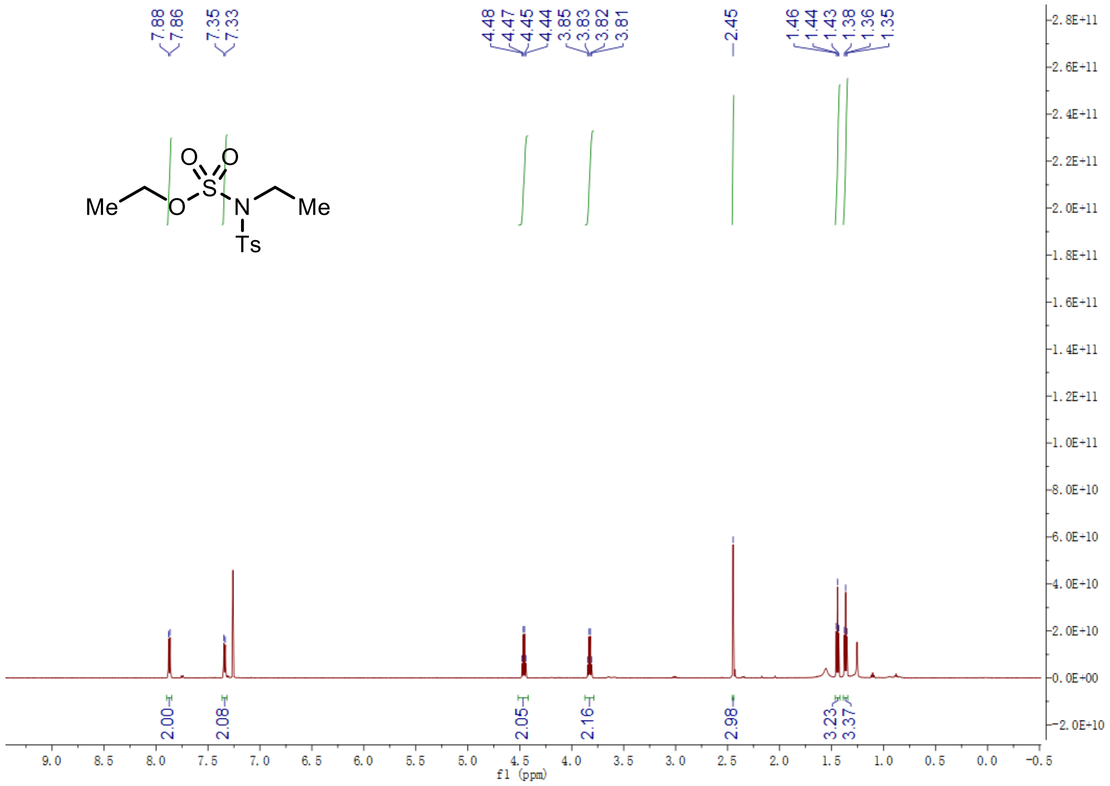

$^{13}C\{^{1}H\}$ NMR spectrum of compound **4j'** (151 MHz, $CDCl_3$)

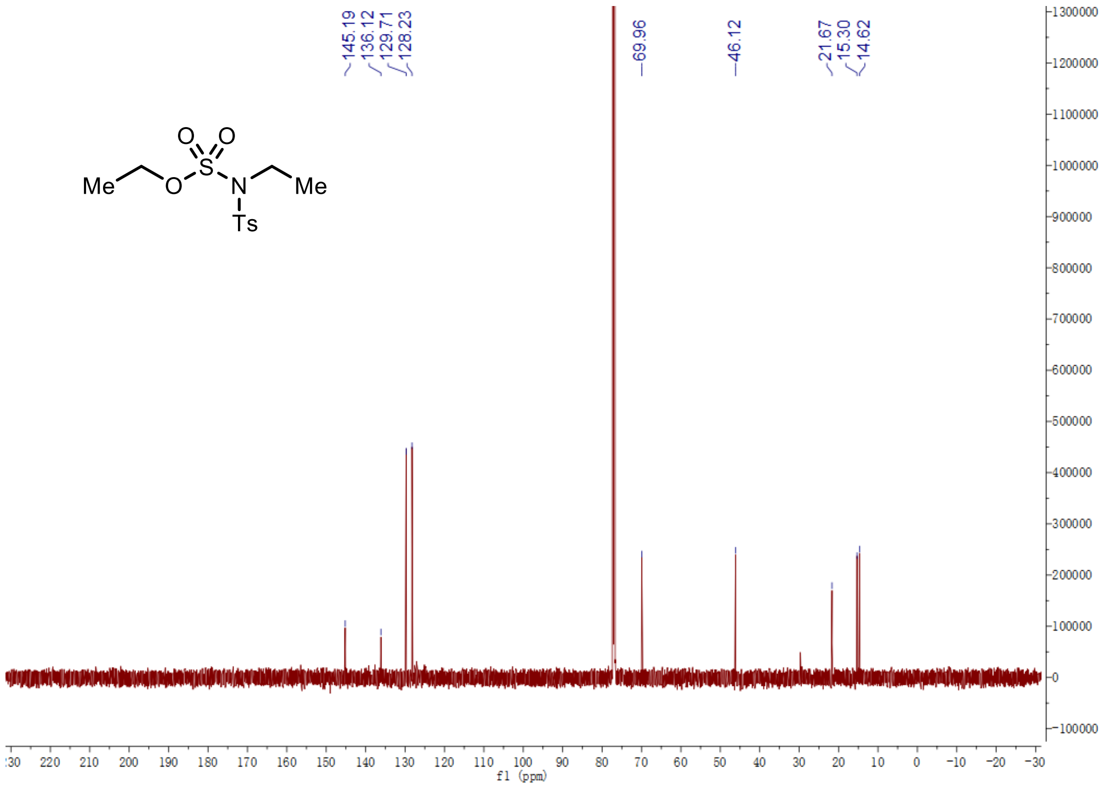


$^{1}H$ NMR spectrum of compound **4m** (600 MHz, $CDCl_3$)

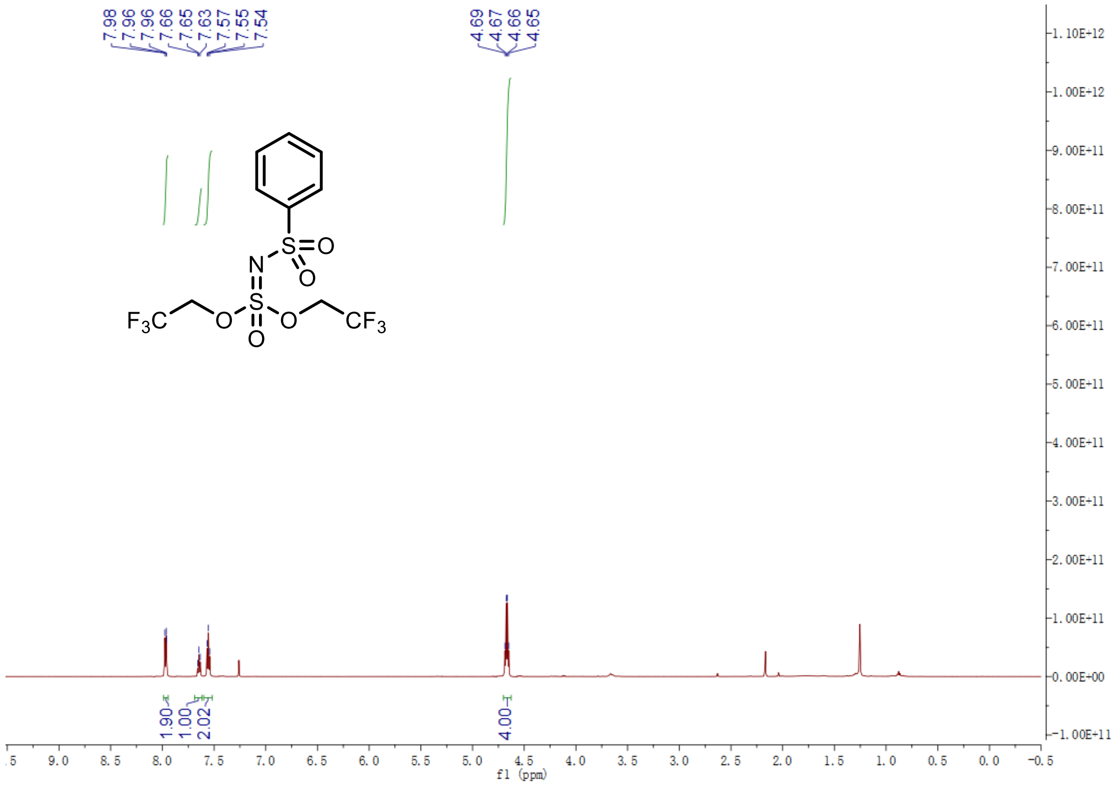

$^{13}C\{^1H\}$ NMR spectrum of compound **4m** (151 MHz, $CDCl_3$)

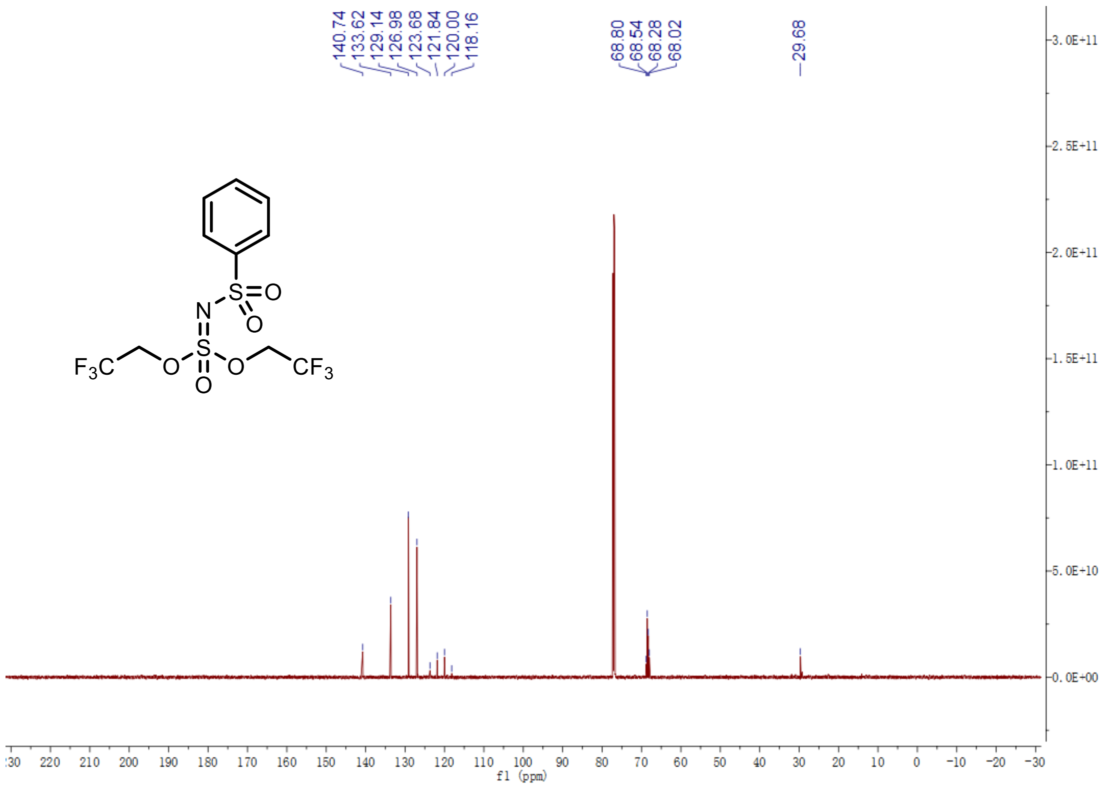


$^{19}F$ NMR spectrum of compound **4m** (564 MHz, $CDCl_3$)

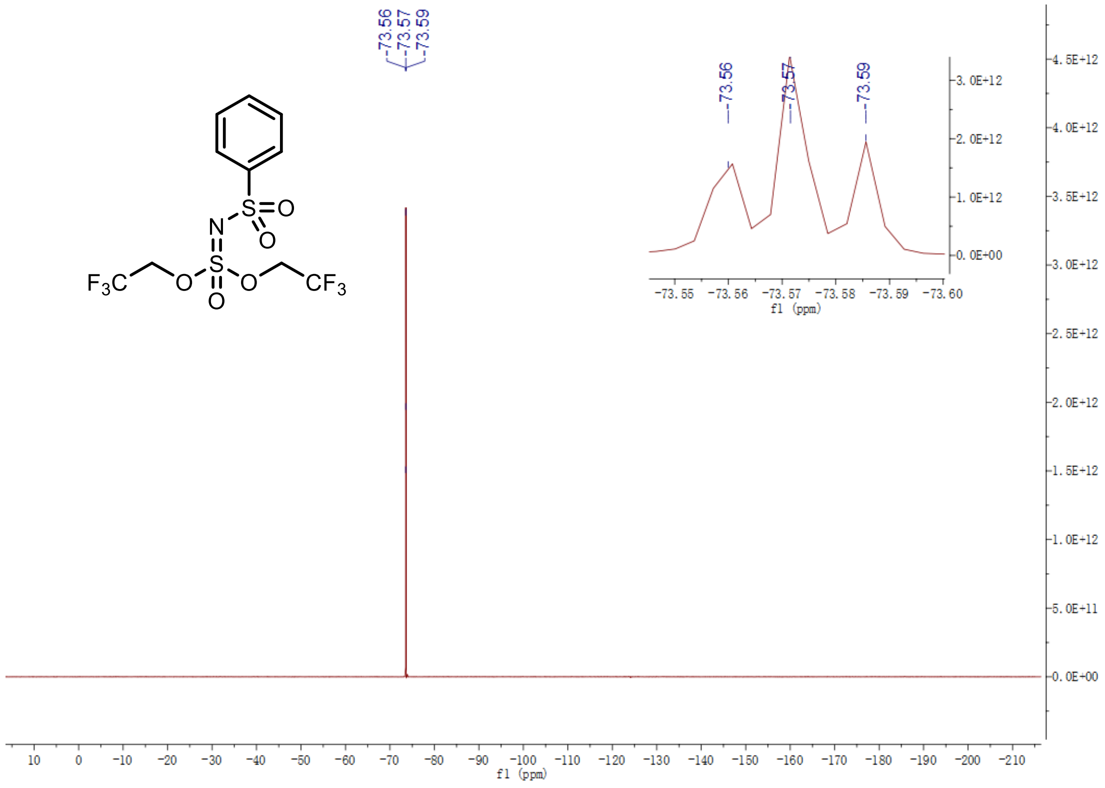

$^{1}$H NMR spectrum of compound **4n** (600 MHz, $CDCl_3$)

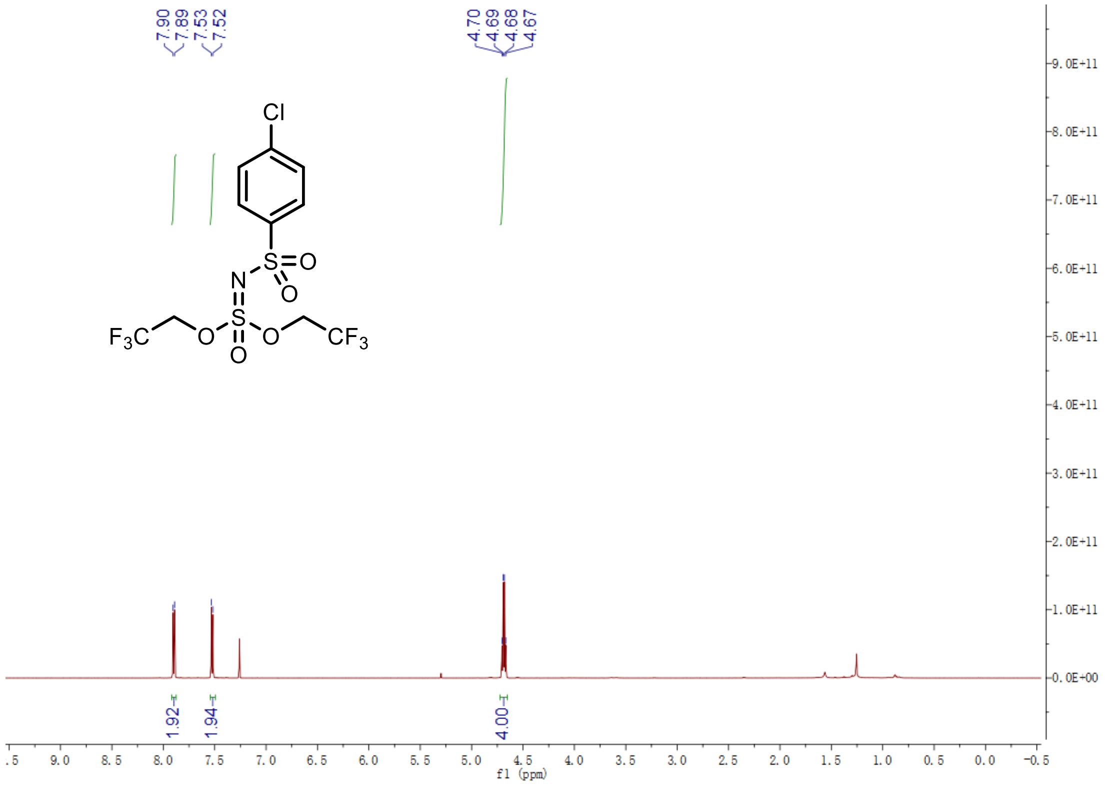


$^{13}$C{$^{1}$H} NMR spectrum of compound **4n** (151 MHz, $CDCl_3$)

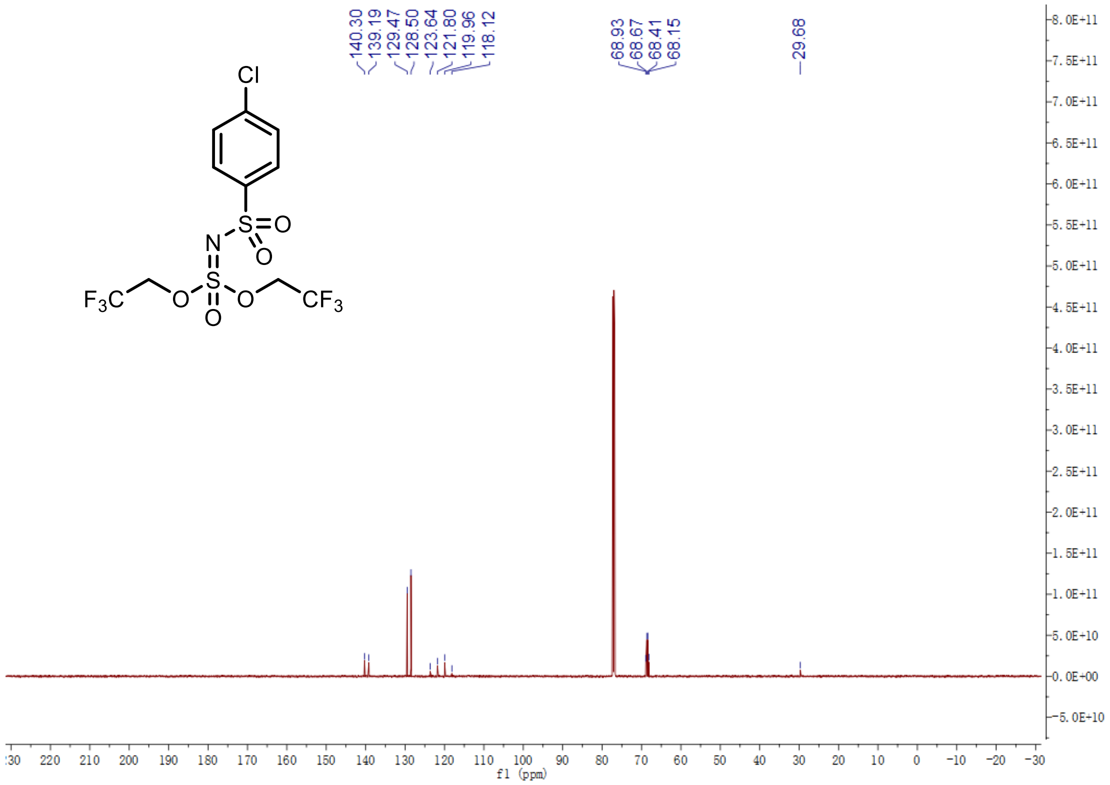

$^{19}$F NMR spectrum of compound **4n** (564 MHz, $CDCl_3$)

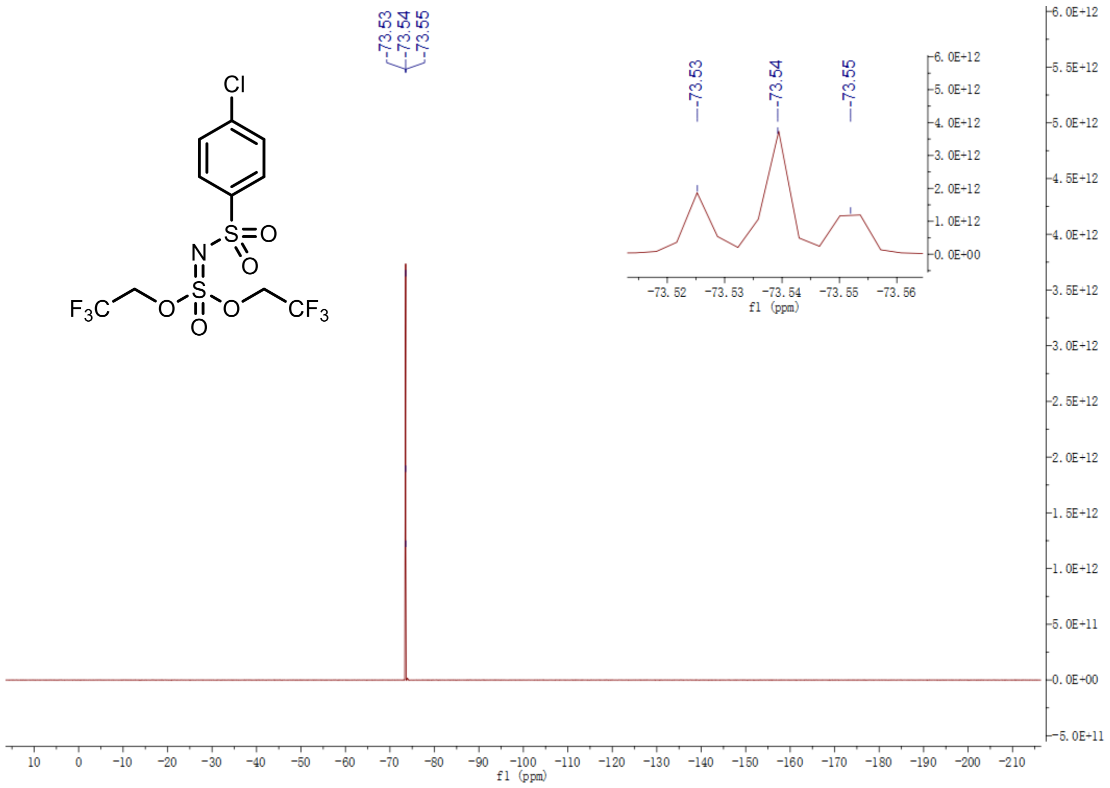


$^{1}$H NMR spectrum of compound **4o** (600 MHz, $CDCl_3$)

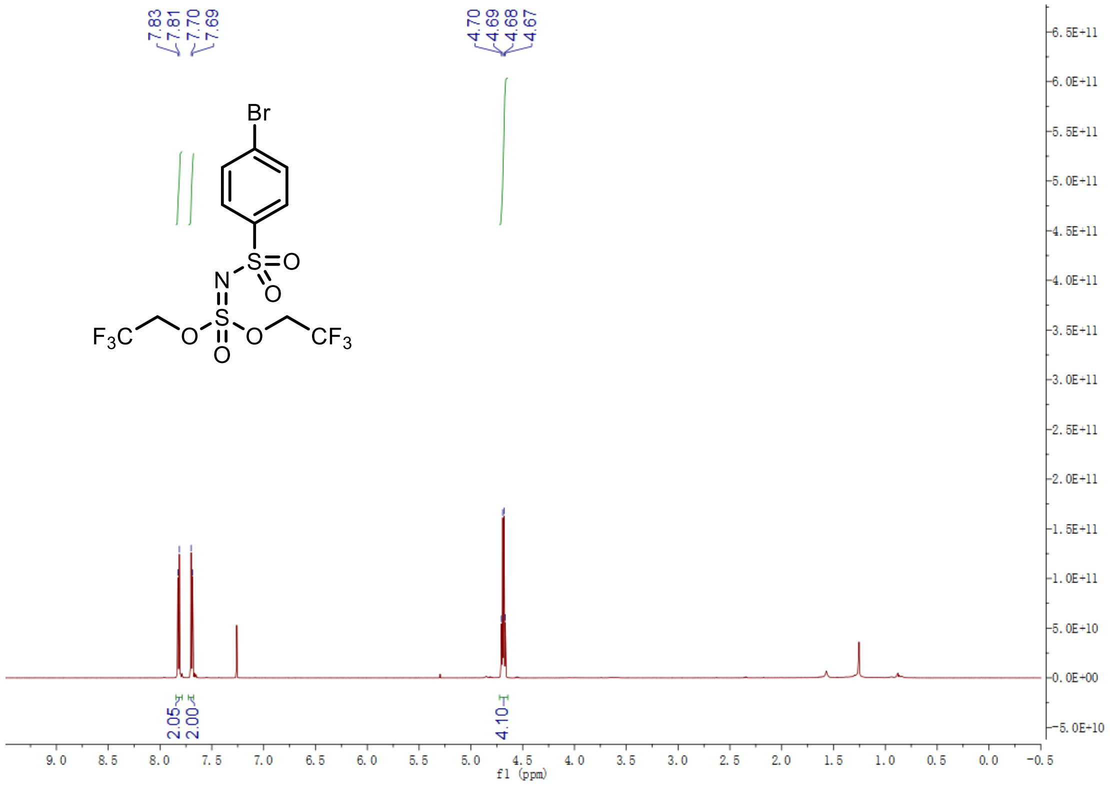

$^{13}C\{^1H\}$ NMR spectrum of compound **4o** (151 MHz, $CDCl_3$)

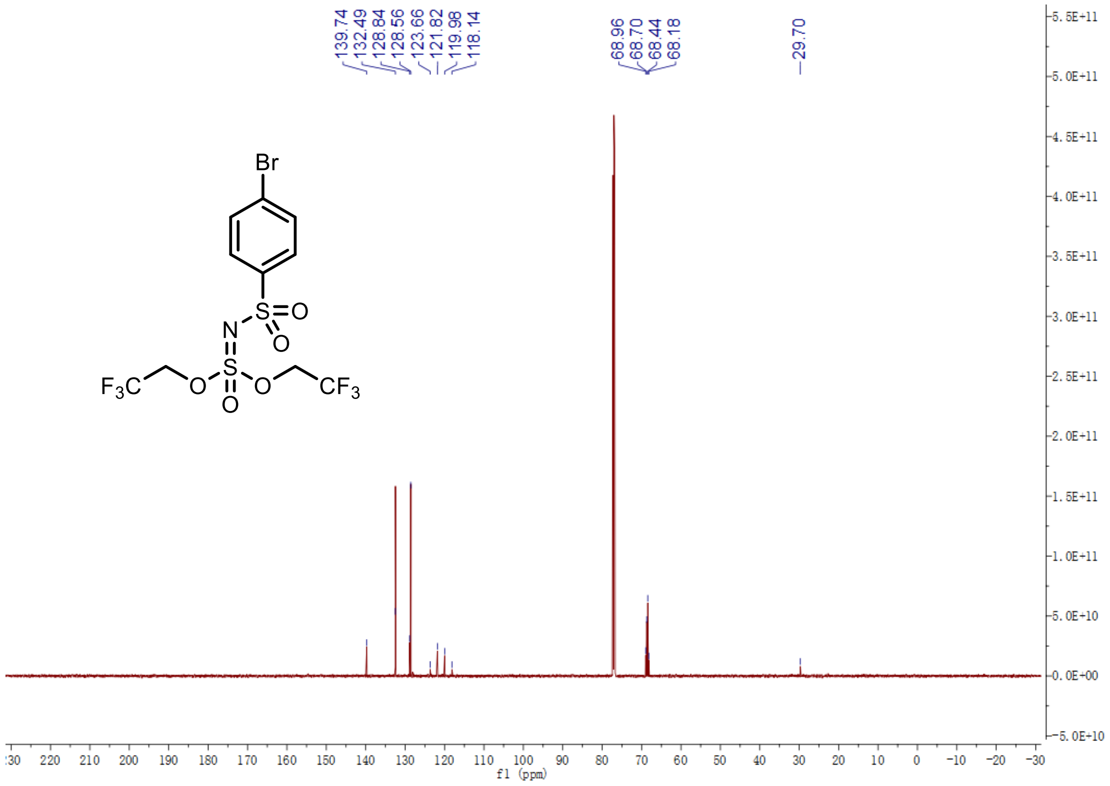


$^{19}F$ NMR spectrum of compound **4o** (564 MHz, $CDCl_3$)

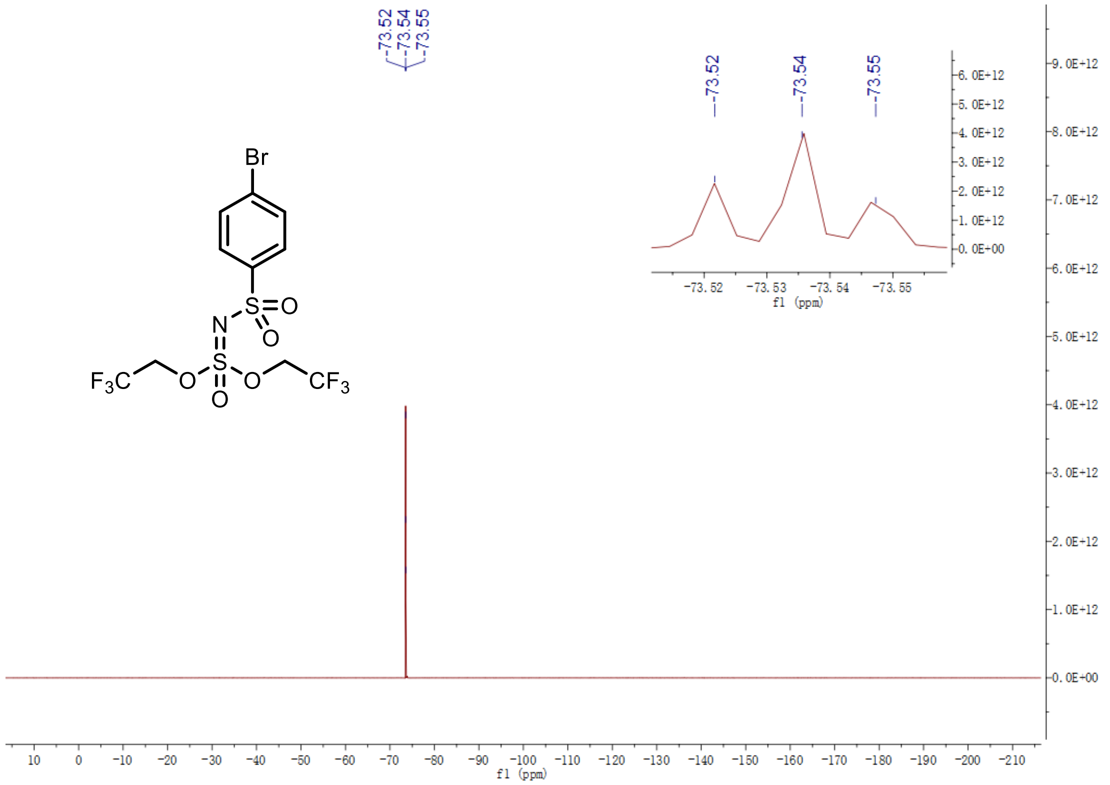

$^{1}$H NMR spectrum of compound **4p** (600 MHz, $CDCl_3$)

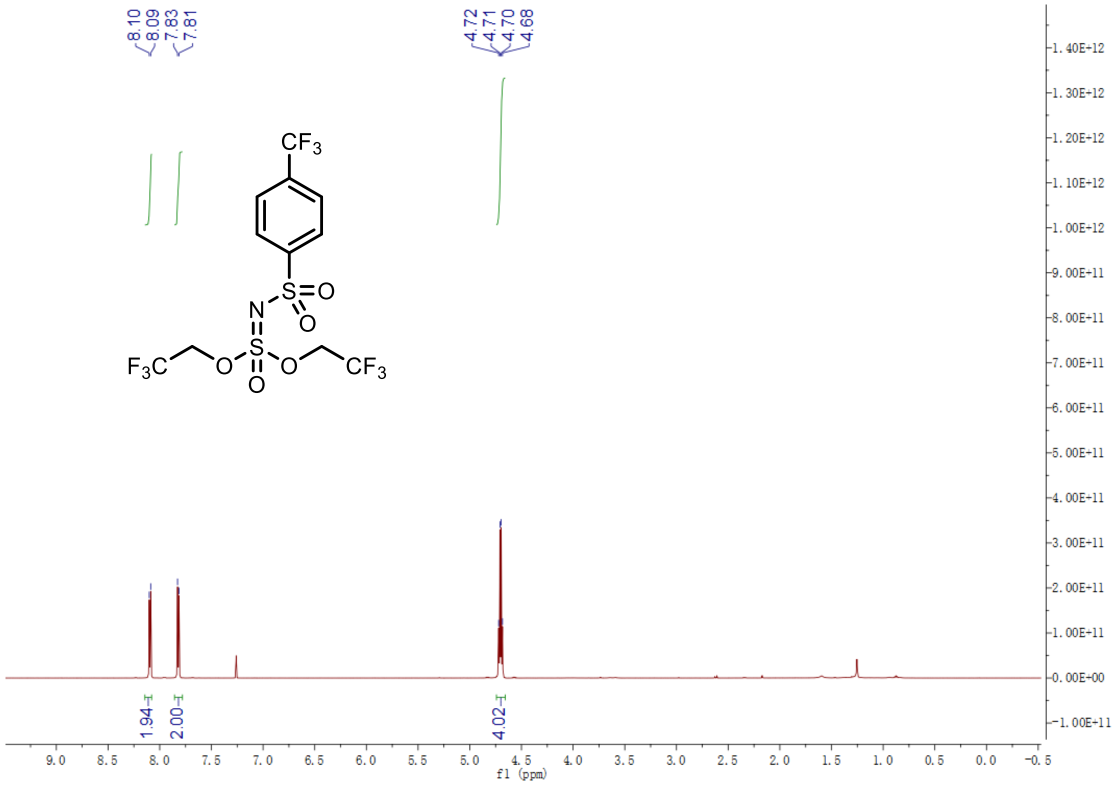

$^{13}$C{$^{1}$H} NMR spectrum of compound **4p** (151 MHz, $CDCl_3$)

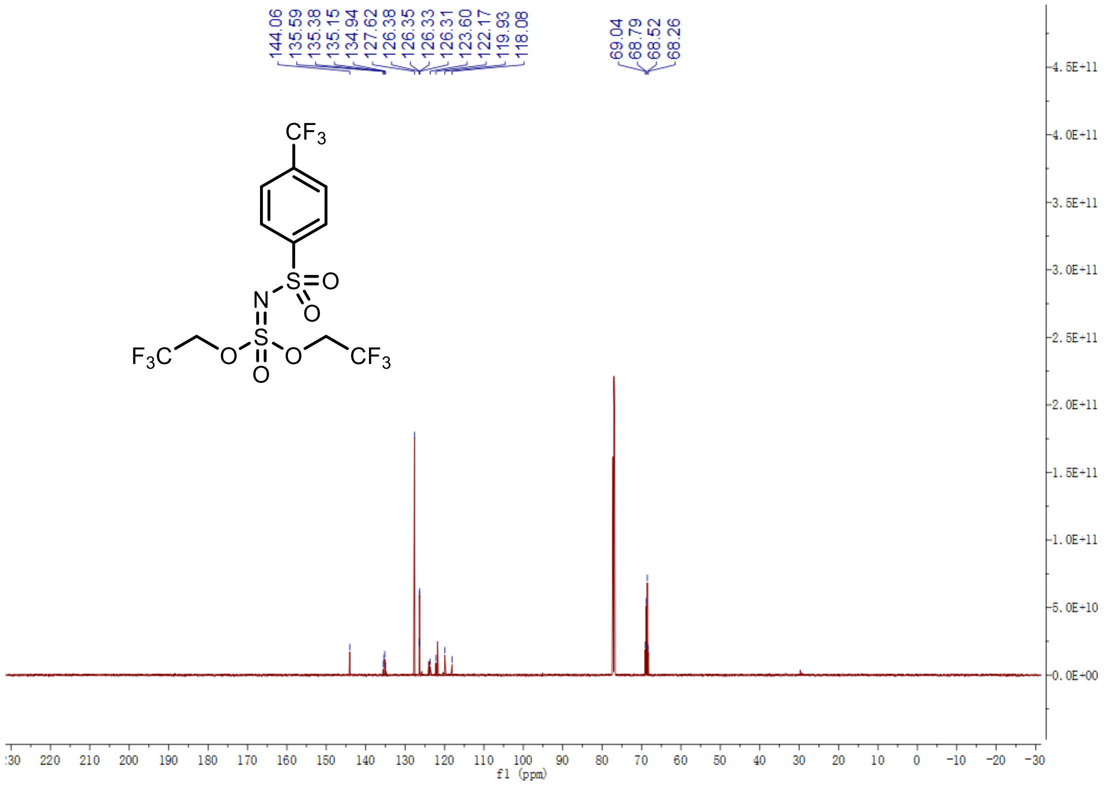

## $^{19}$F NMR spectrum of compound **4p** (564 MHz, $CDCl_3$)

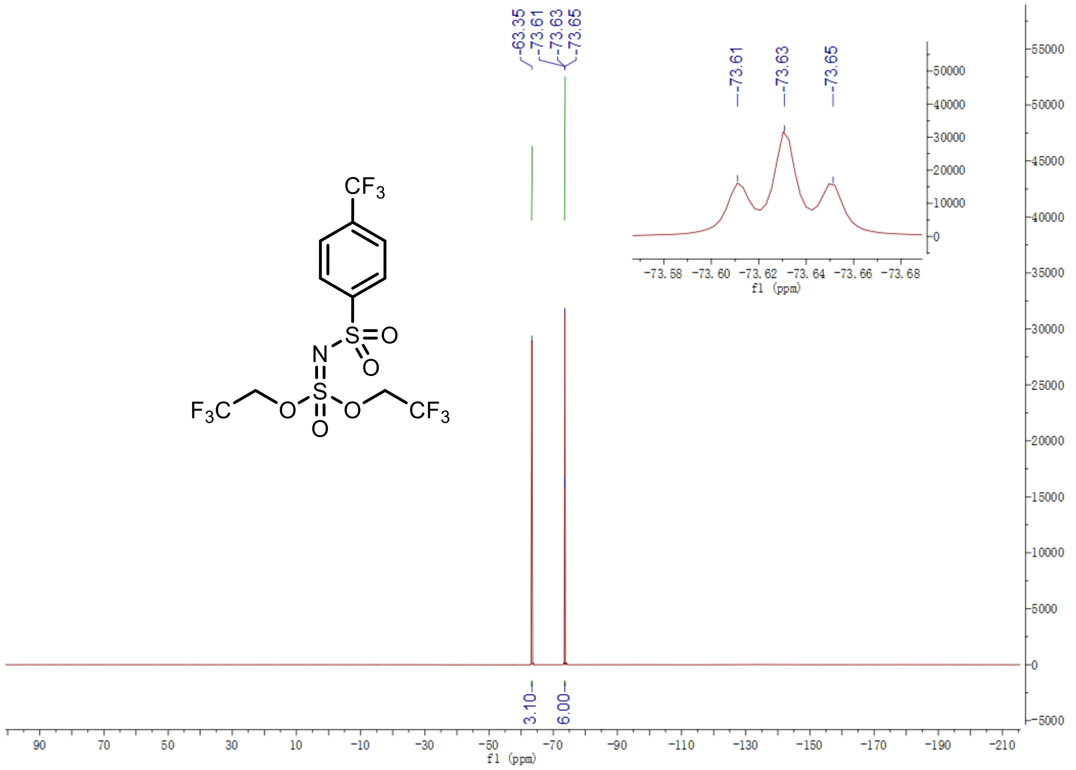


## $^{1}$H NMR spectrum of compound **4q** (600 MHz, $CDCl_3$)

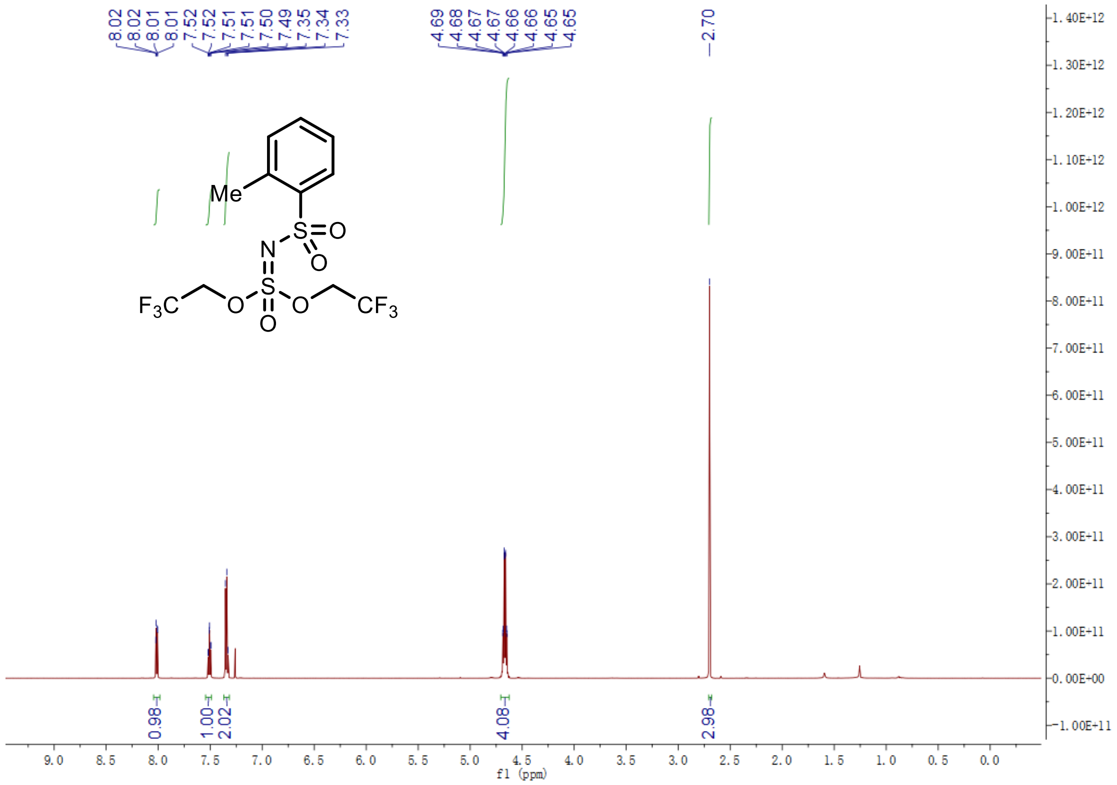

$^{13}C\{^{1}H\}$ NMR spectrum of compound **4q** (151 MHz, $CDCl_3$)

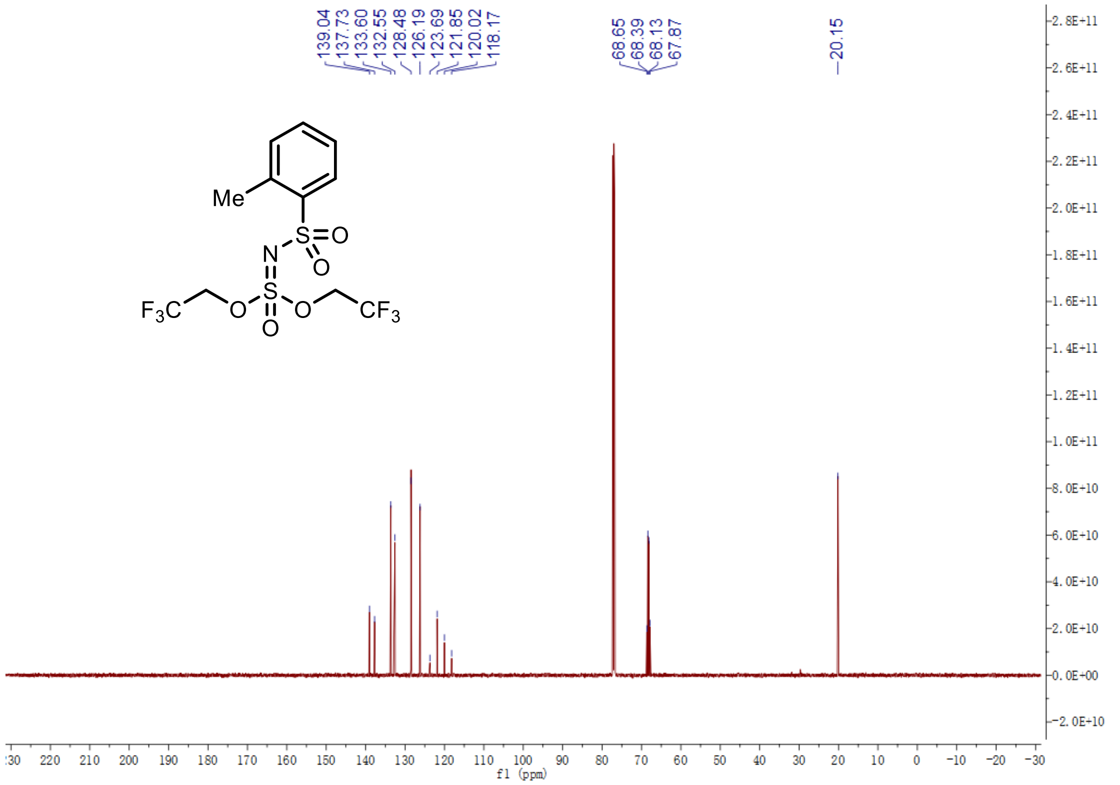


$^{19}F$ NMR spectrum of compound **4q** (564 MHz, $CDCl_3$)

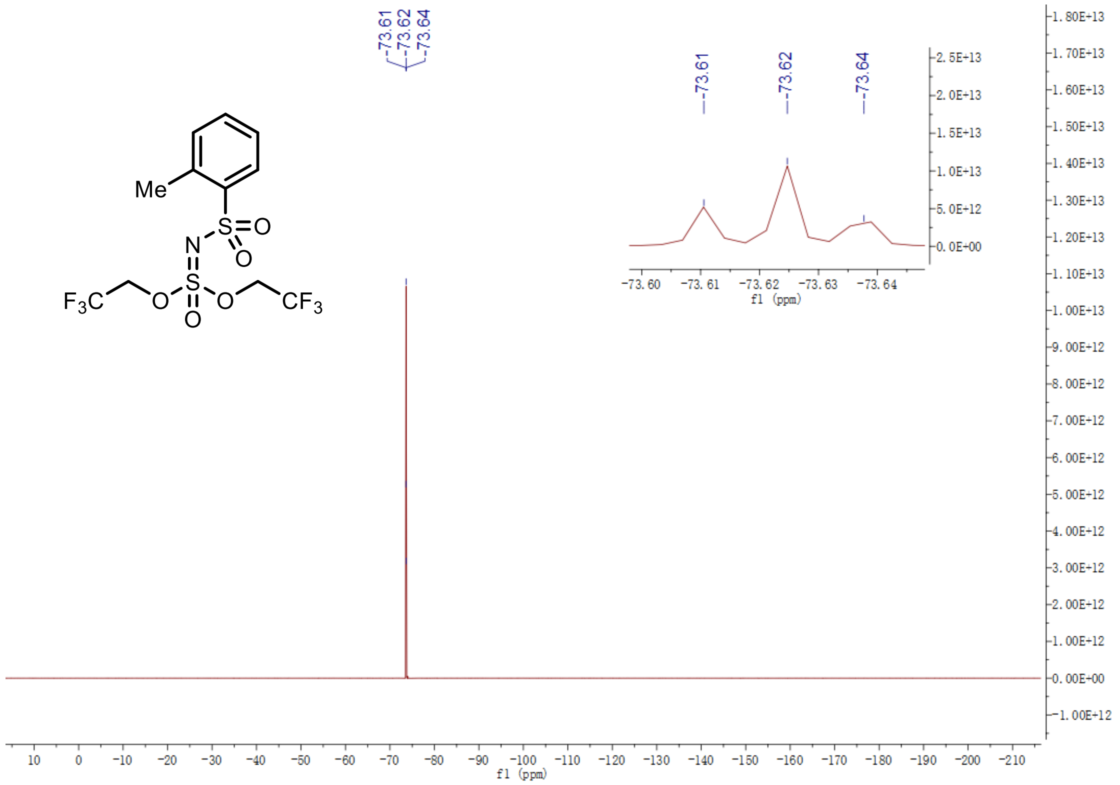

$^{1}$H NMR spectrum of compound **4r** (600 MHz, $CDCl_3$)

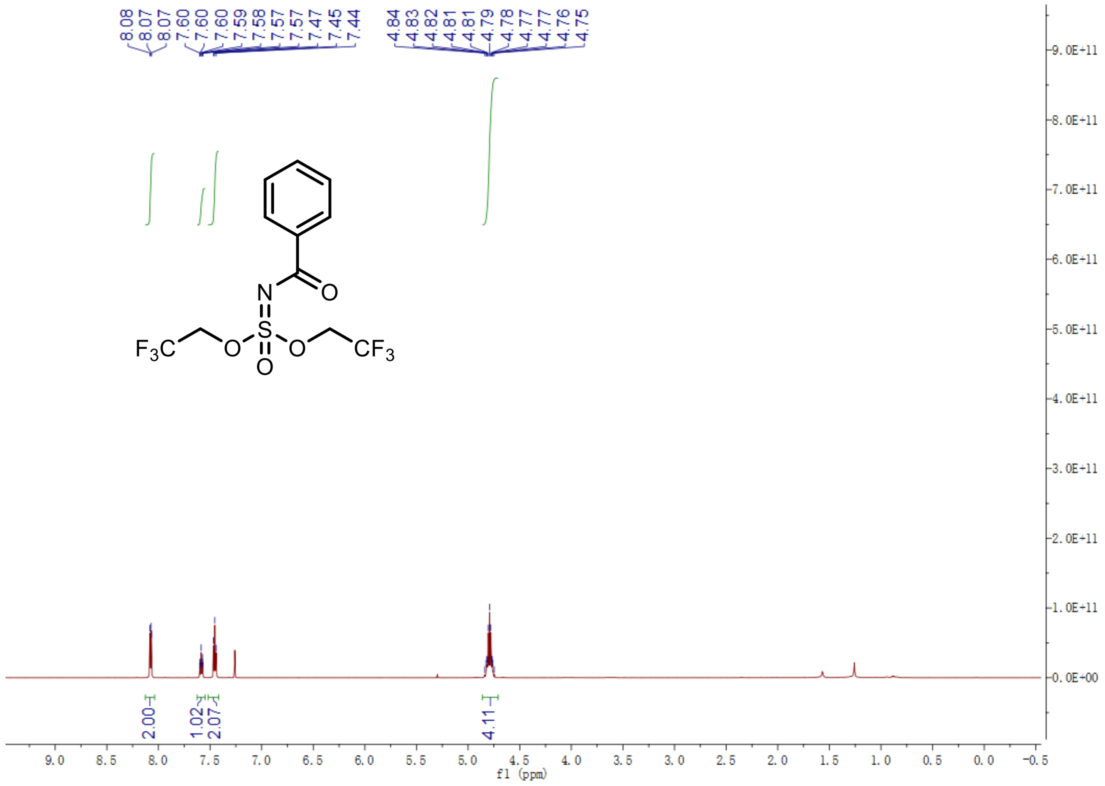

$^{13}$C{$^{1}$H} NMR spectrum of compound **4r** (151 MHz, $CDCl_3$)

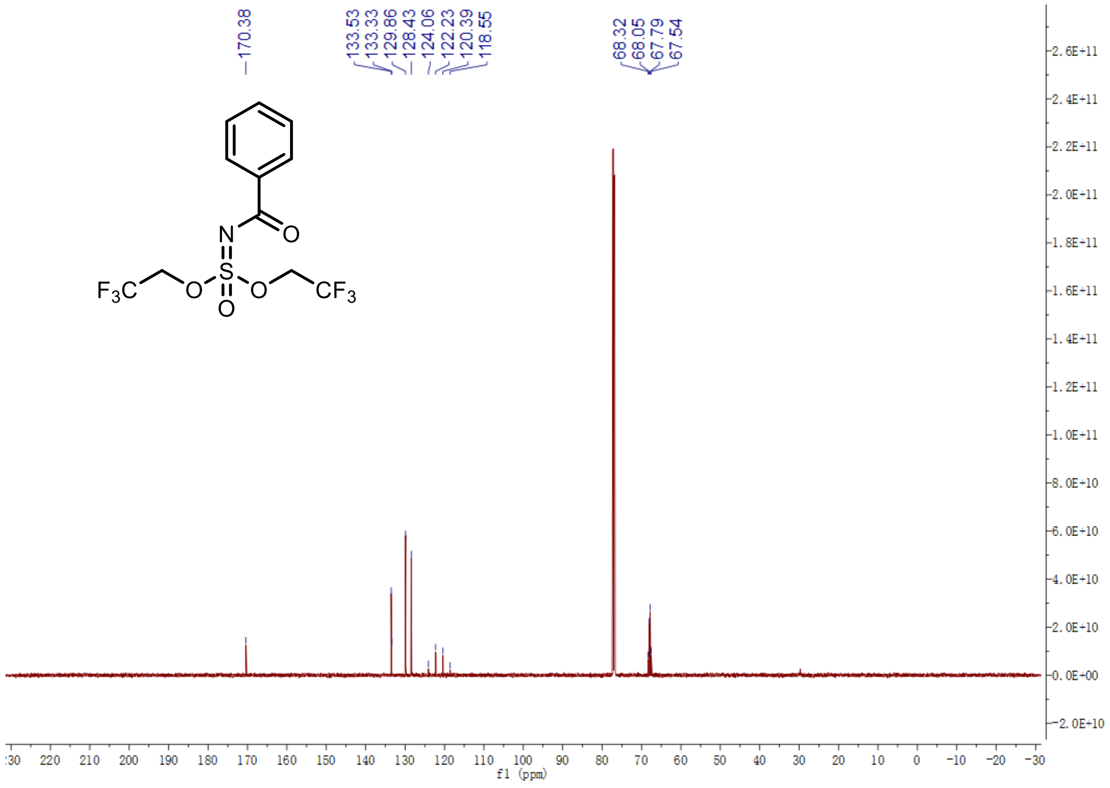

$^{19}$F NMR spectrum of compound **4r** (564 MHz, $CDCl_3$)

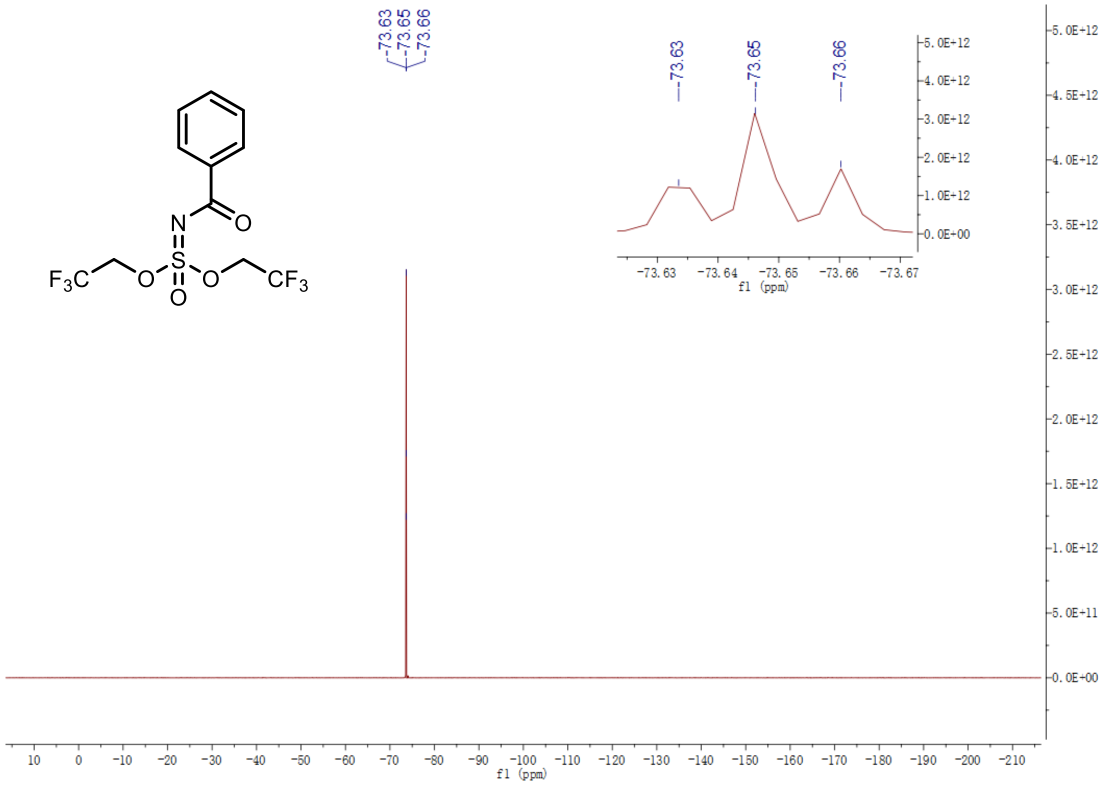


$^{1}$H NMR spectrum of compound **4s** (600 MHz, $CDCl_3$)

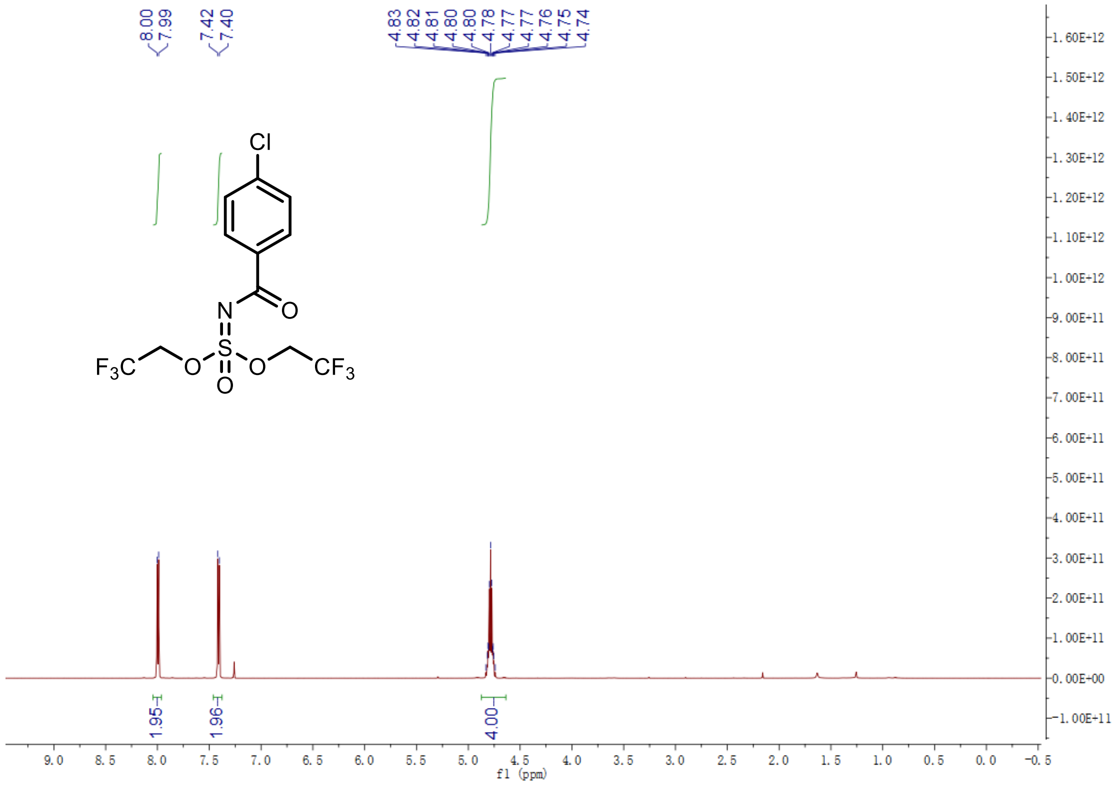

$^{13}C\{^{1}H\}$ NMR spectrum of compound **4s** (151 MHz, $CDCl_3$)

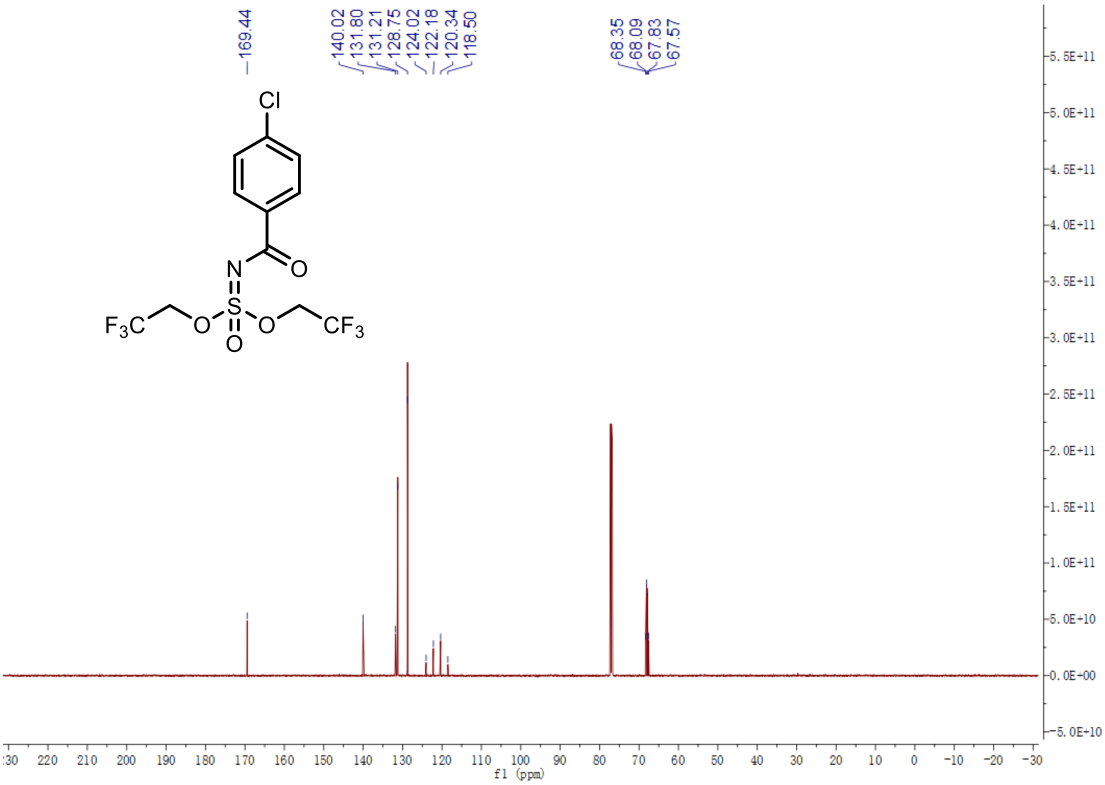


$^{19}F$ NMR spectrum of compound **4s** (564 MHz, $CDCl_3$)

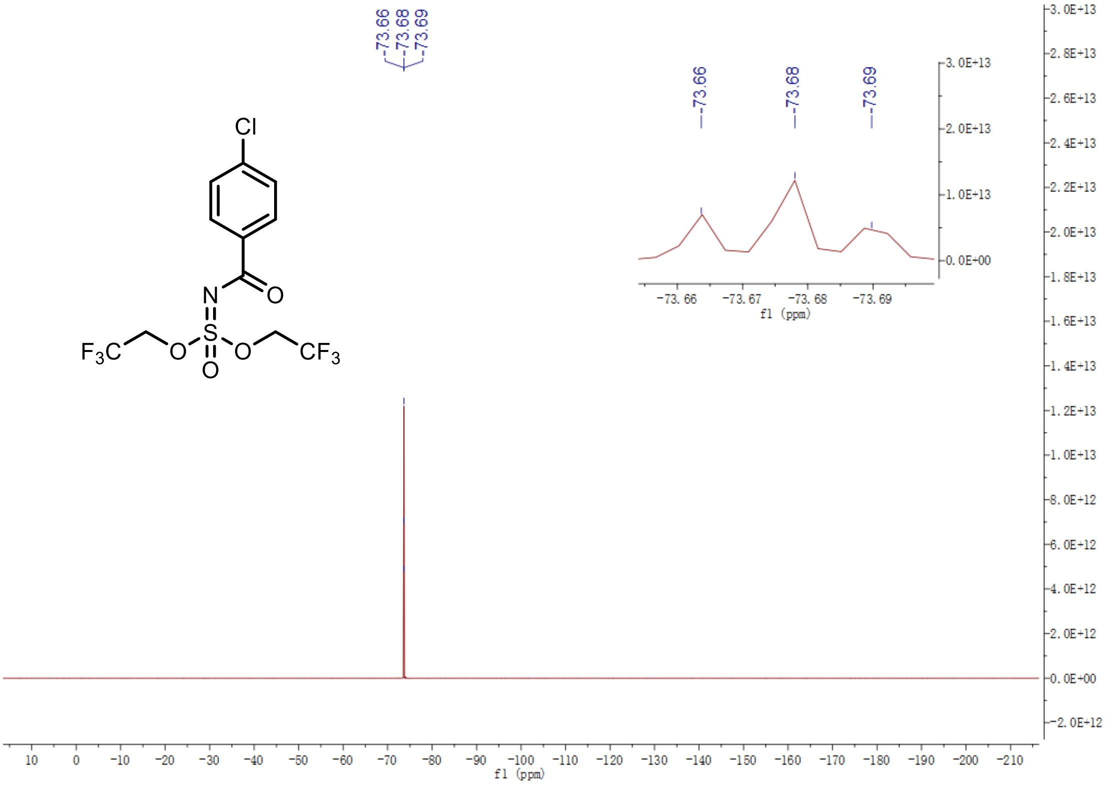

$^{1}H$ NMR spectrum of compound **4t** (600 MHz, $CDCl_3$)

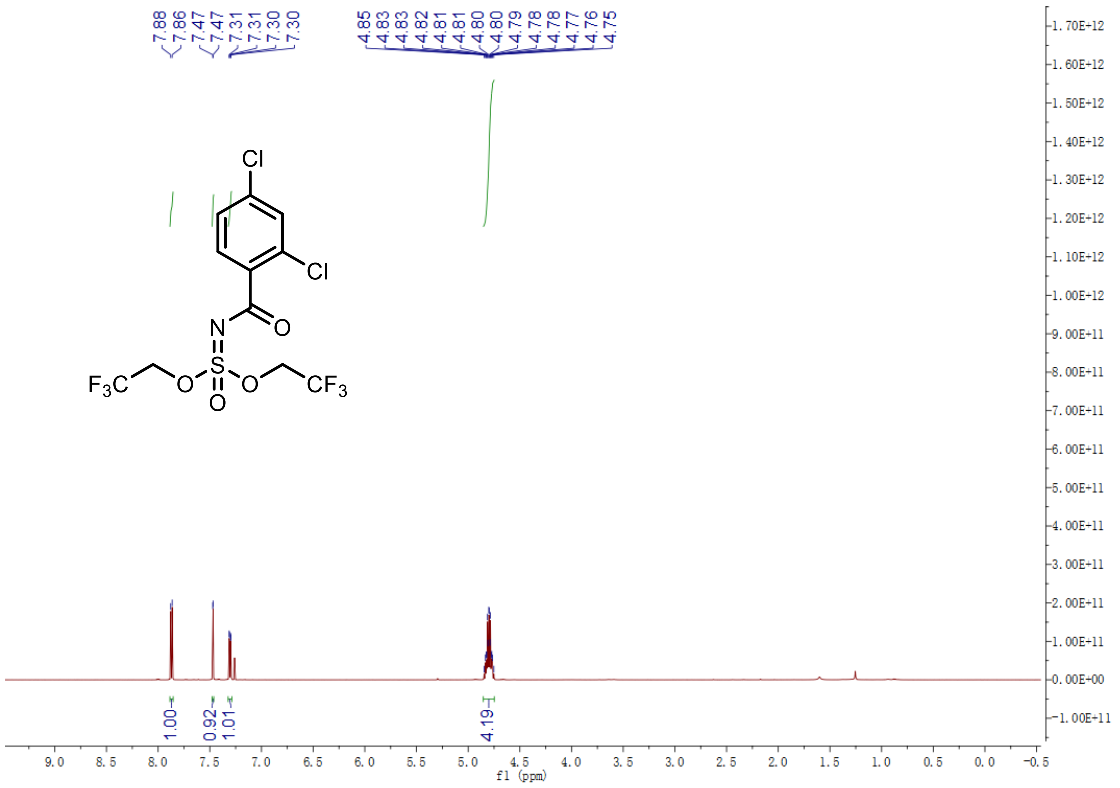

$^{13}C\{^{1}H\}$ NMR spectrum of compound **4t** (151 MHz, $CDCl_3$)

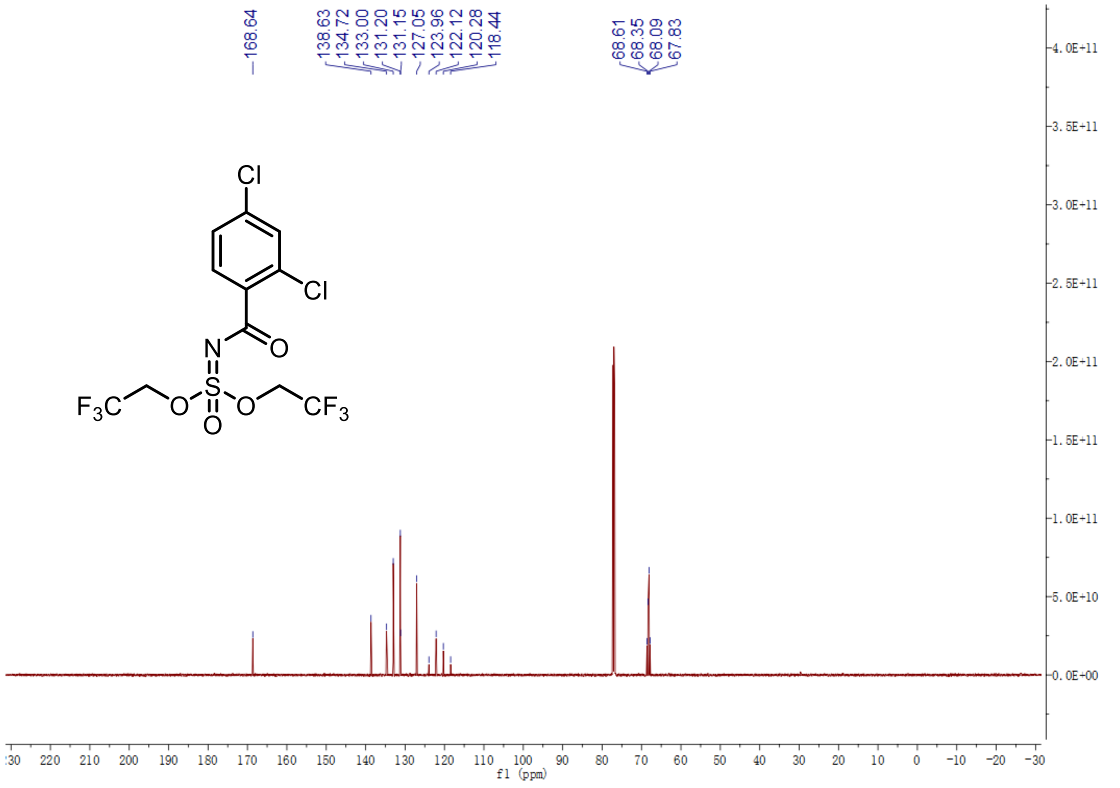

$^{19}$F NMR spectrum of compound **4t** (564 MHz, $CDCl_3$)

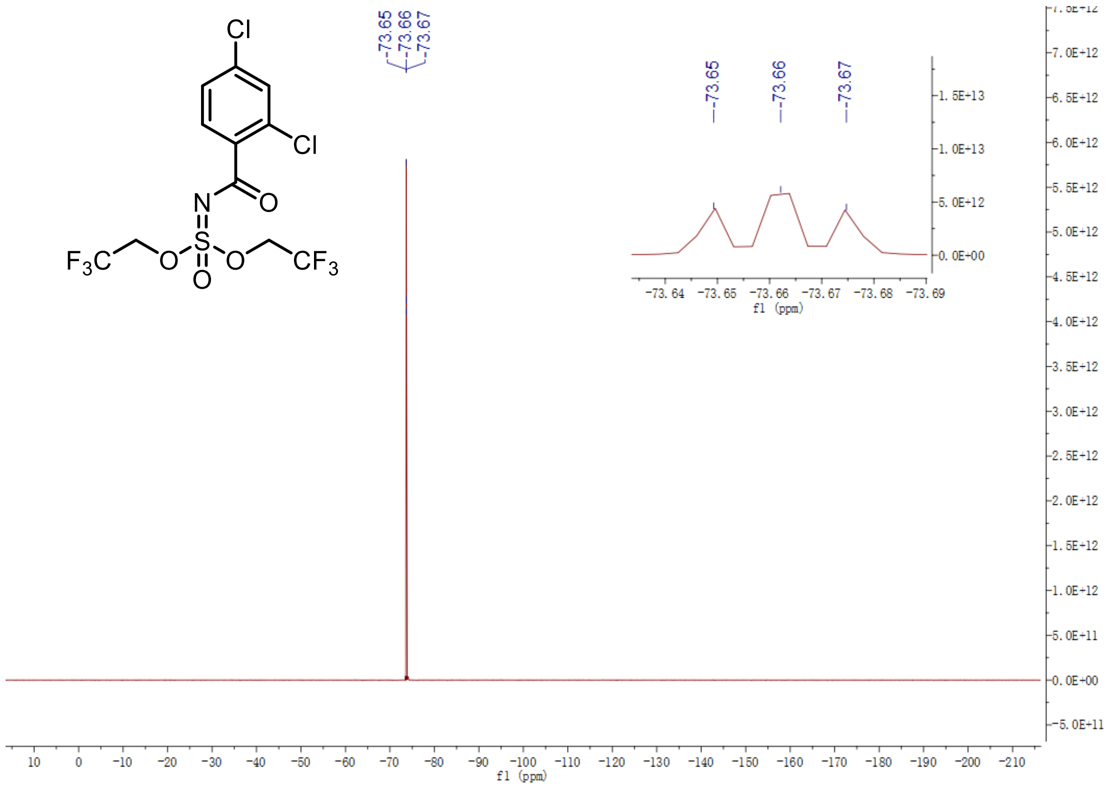

$^{1}$H NMR spectrum of compound **4u** (600 MHz, $CDCl_3$)

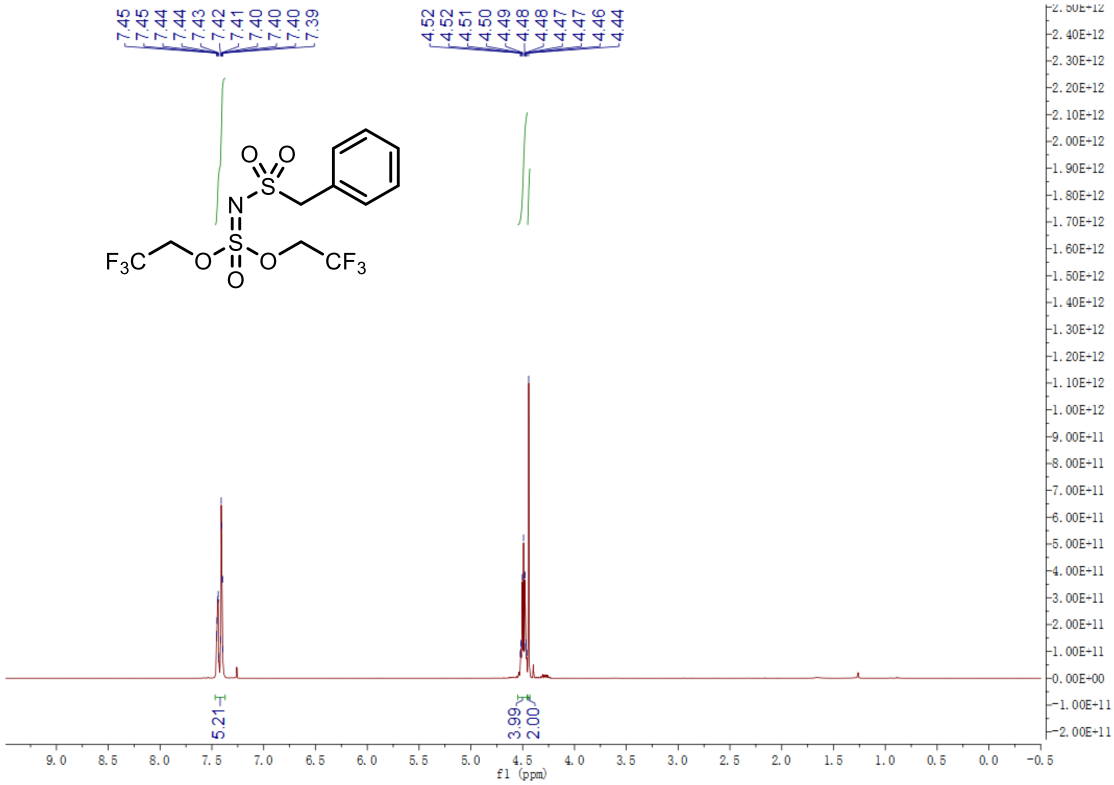

$^{13}C\{^{1}H\}$ NMR spectrum of compound **4u** (151 MHz, $CDCl_3$)

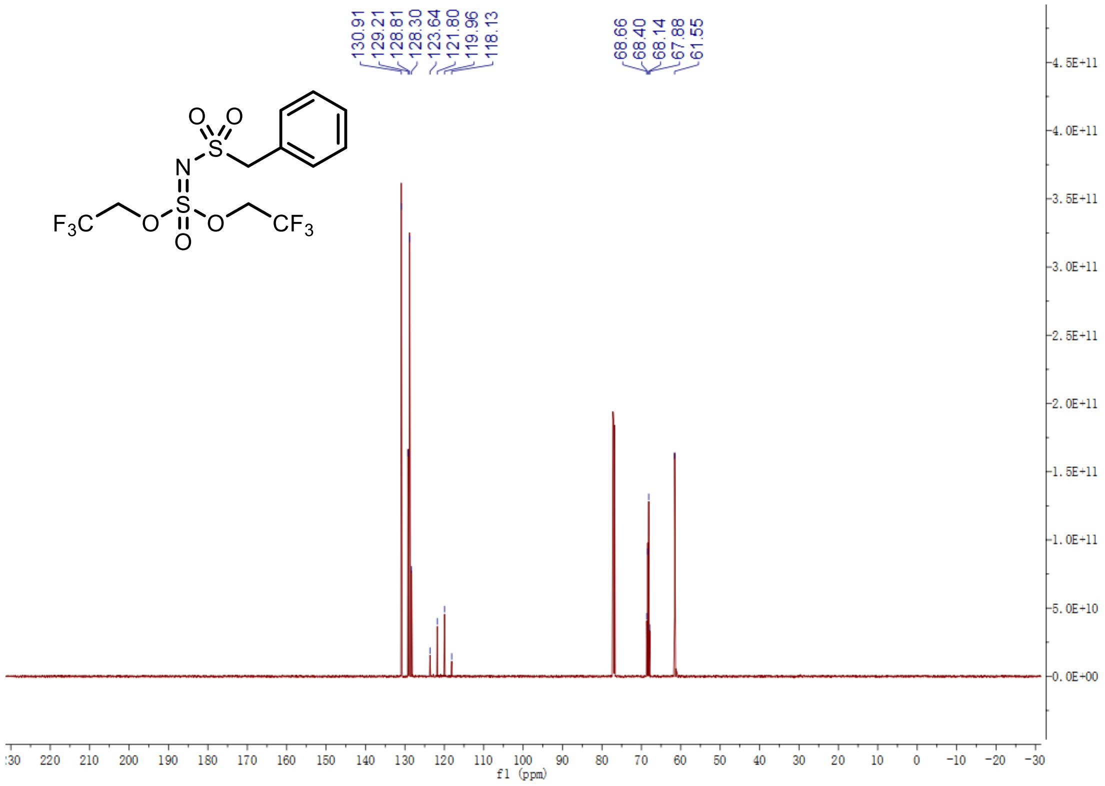


$^{19}F$ NMR spectrum of compound **4u** (564 MHz, $CDCl_3$)

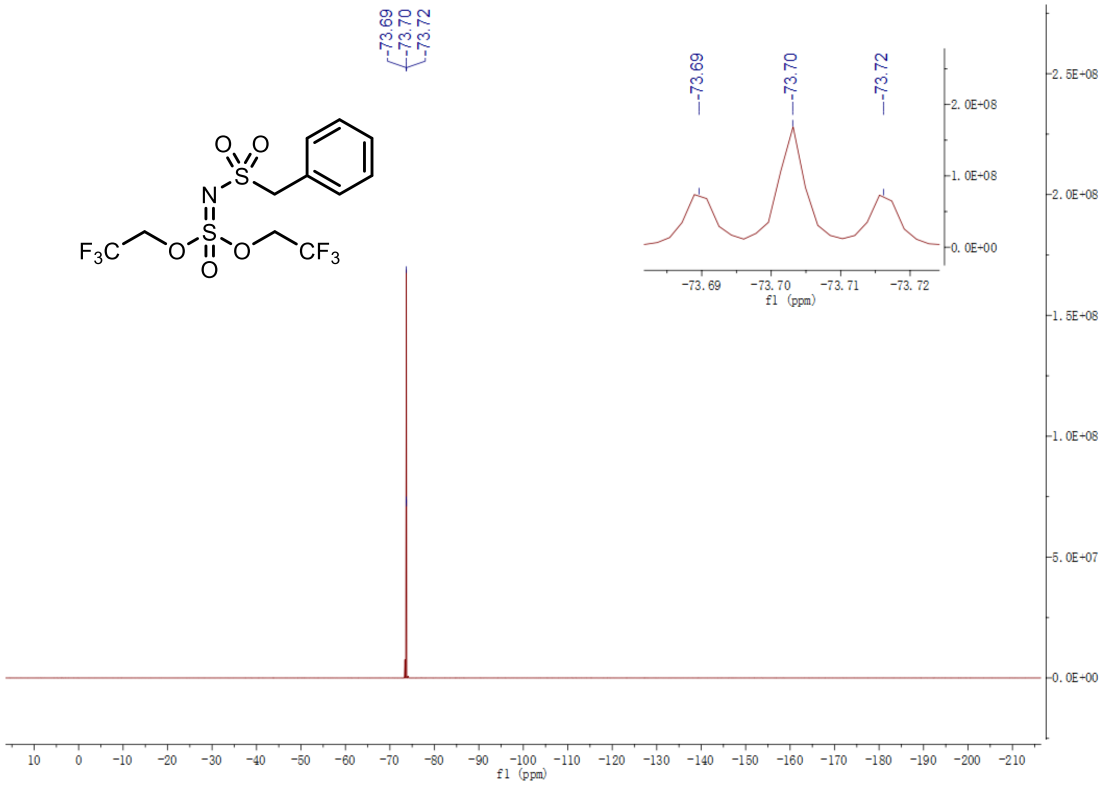

$^{1}$H NMR spectrum of compound **4v** (600 MHz, $CDCl_3$)

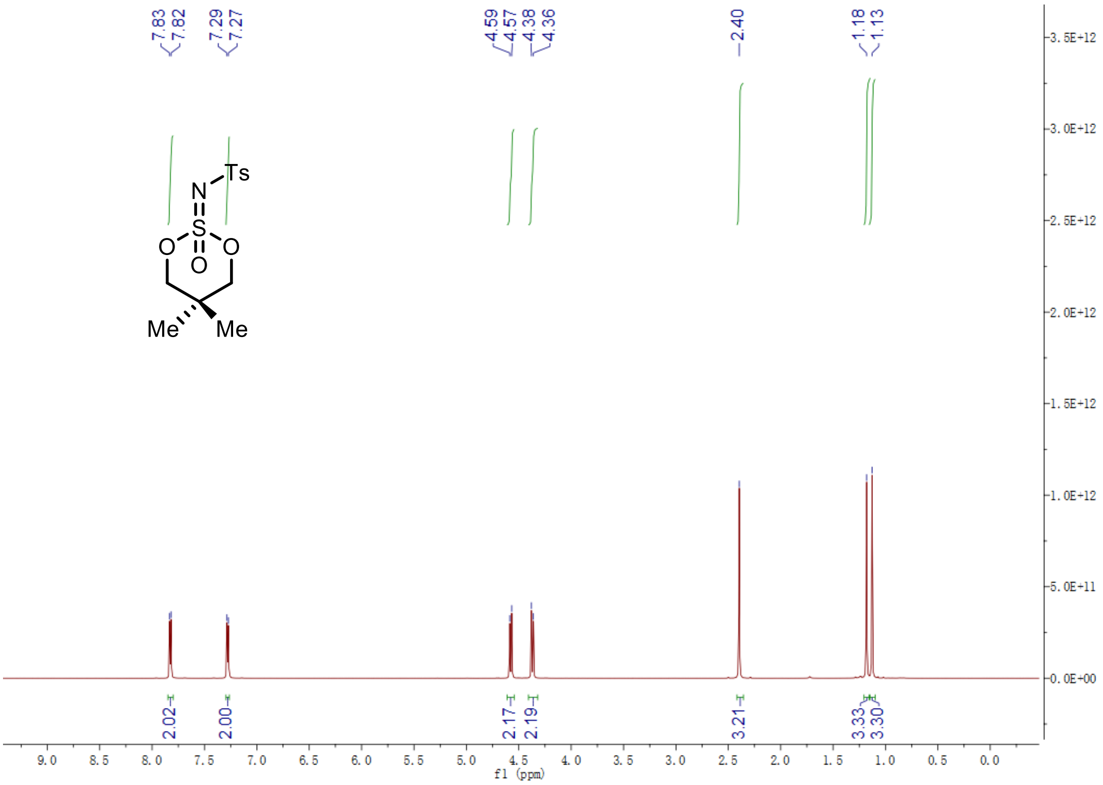

$^{13}$C{$^{1}$H} NMR spectrum of compound **4v** (151 MHz, $CDCl_3$)

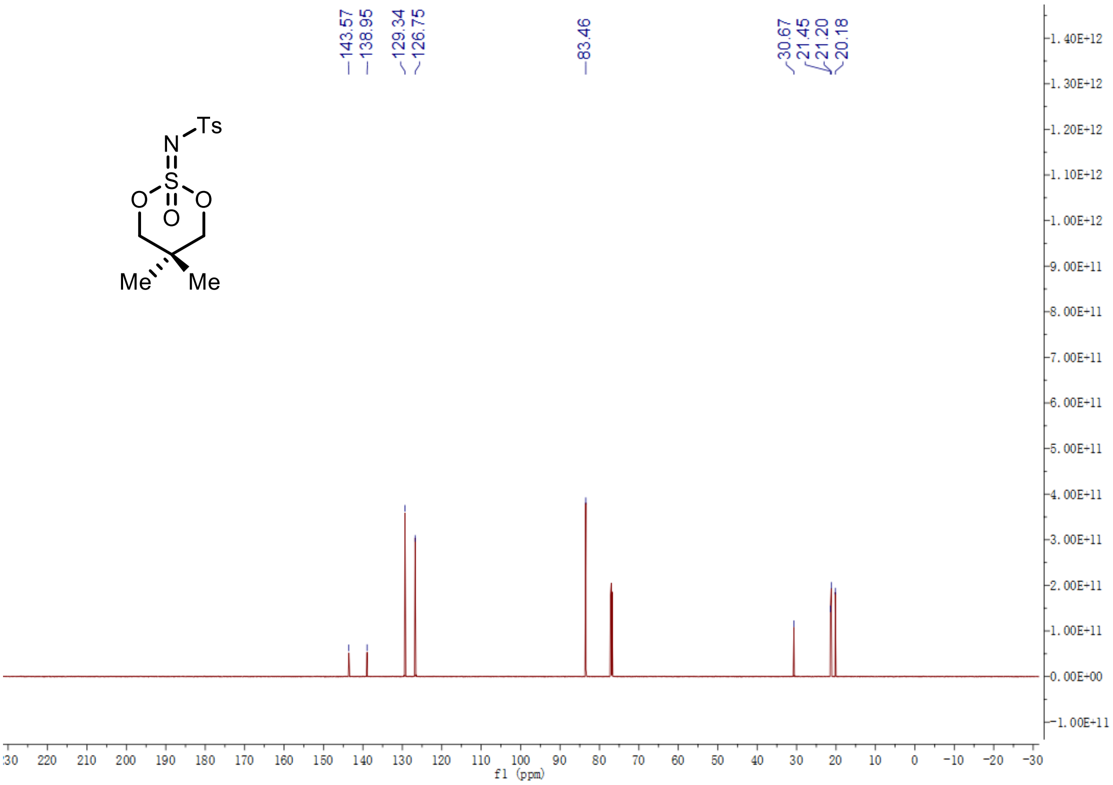

$^{1}$H NMR spectrum of compound **6a** (600 MHz, $CDCl_3$)

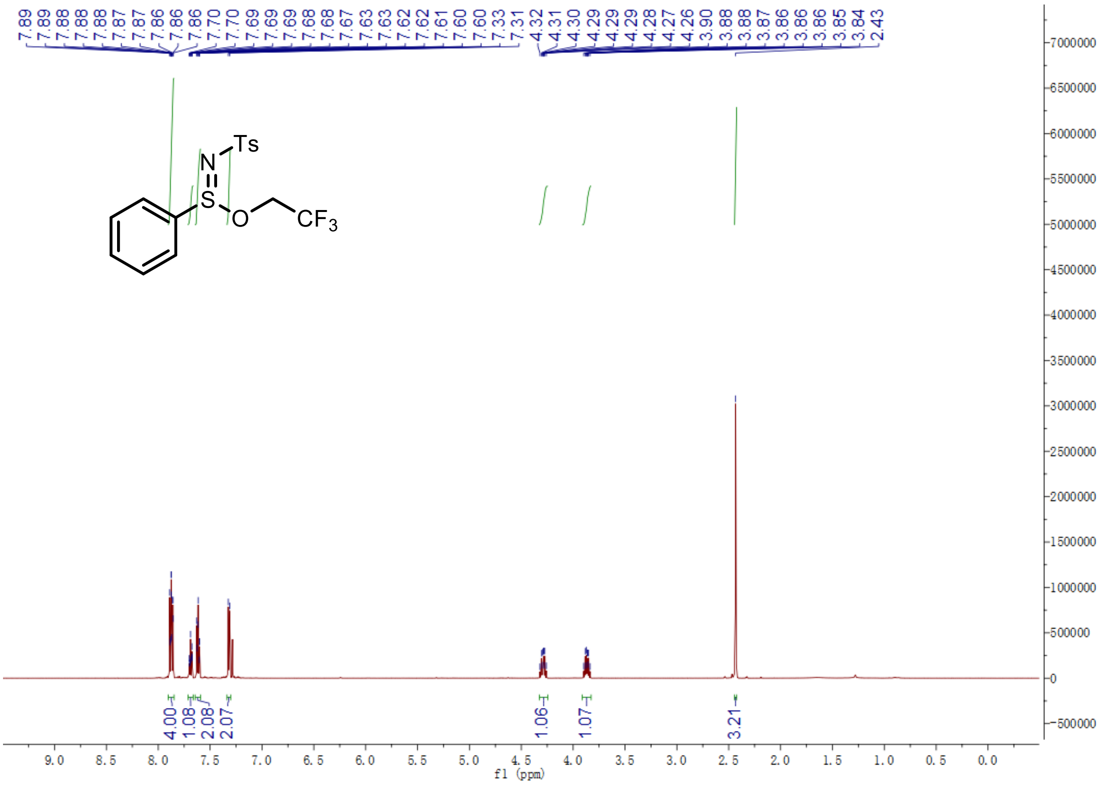

$^{13}$C{$^{1}$H} NMR spectrum of compound **6a** (151 MHz, $CDCl_3$)

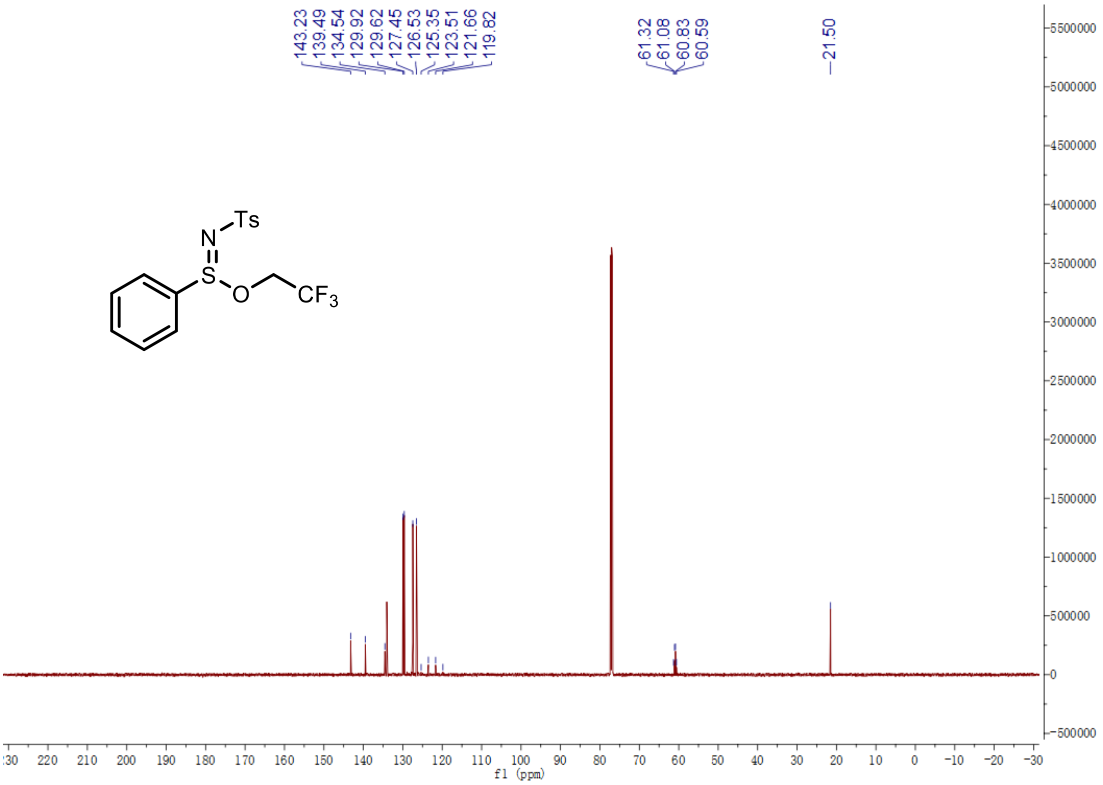

## $^{19}$F NMR spectrum of compound **6a** (564 MHz, $CDCl_3$)

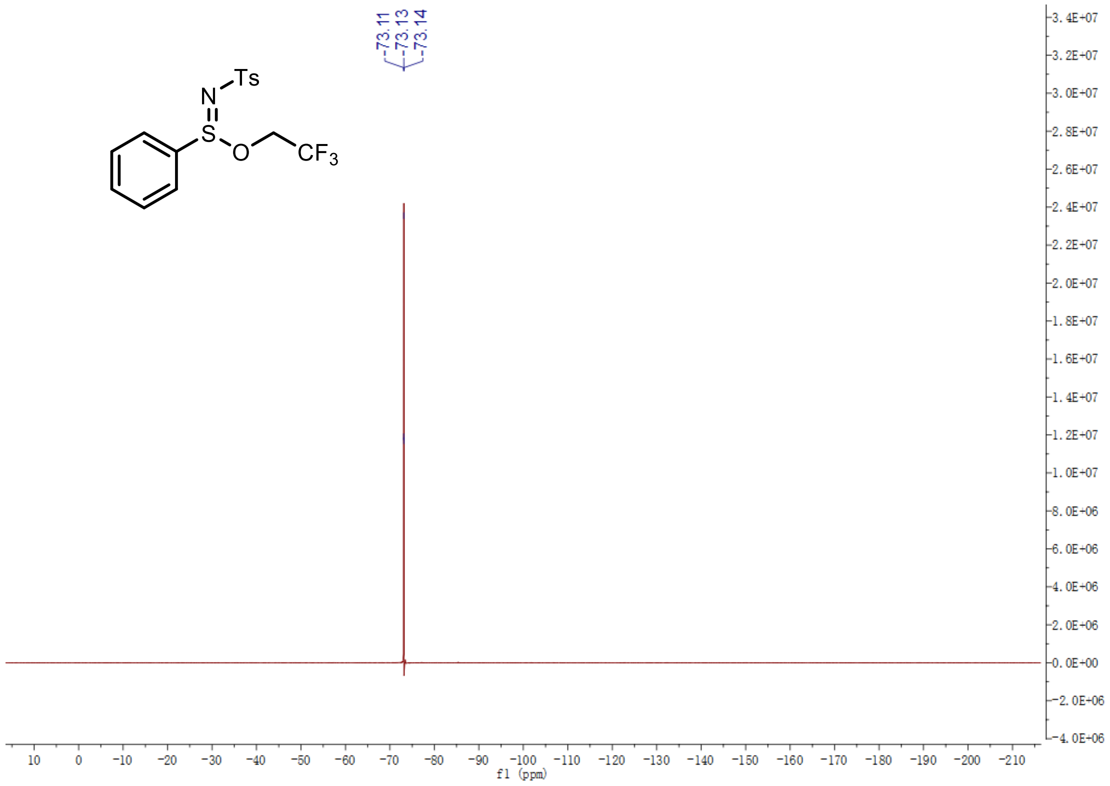

## $^{1}$H NMR spectrum of compound **6b** (600 MHz, $CDCl_3$)

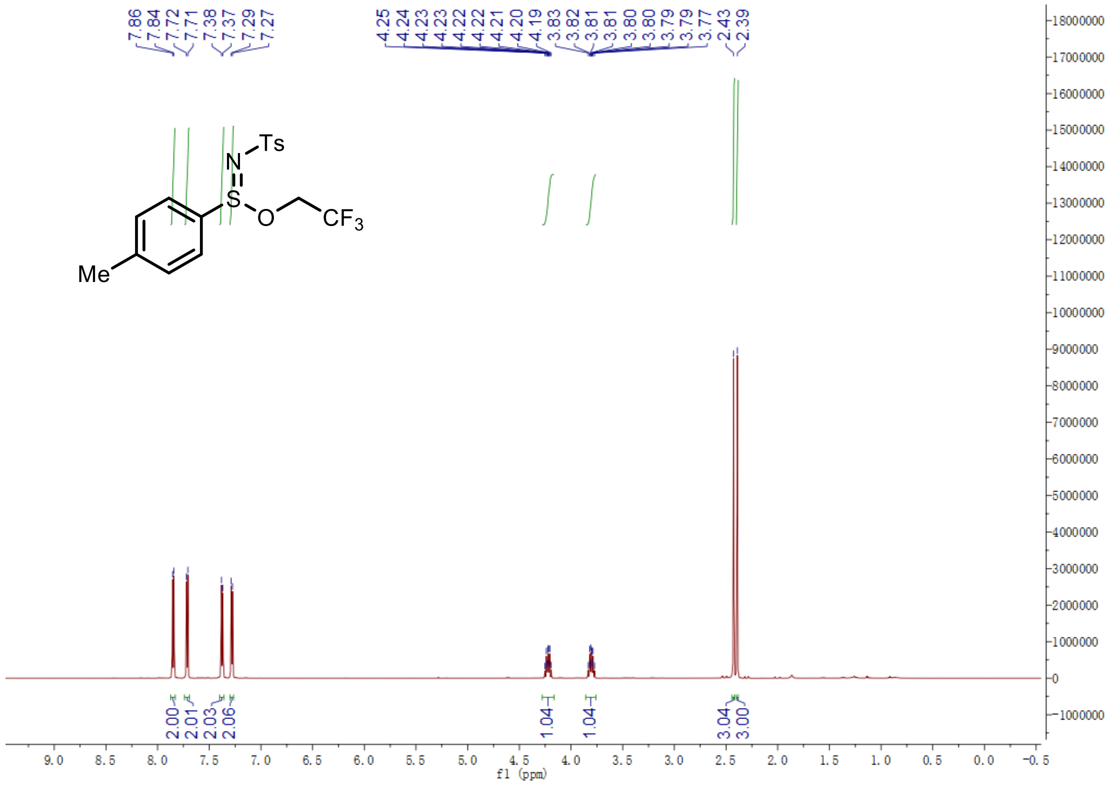

$^{13}C\{^{1}H\}$ NMR spectrum of compound **6b** (151 MHz, $CDCl_3$)

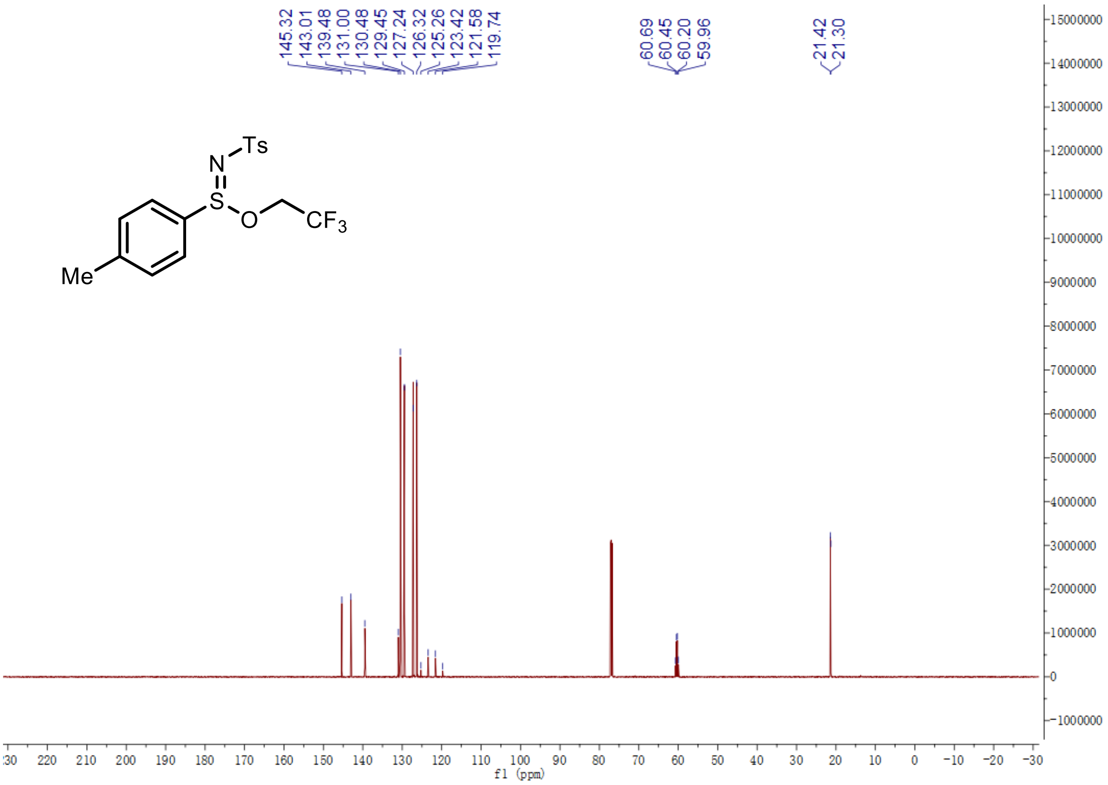


$^{19}F$ NMR spectrum of compound **6b** (564 MHz, $CDCl_3$)

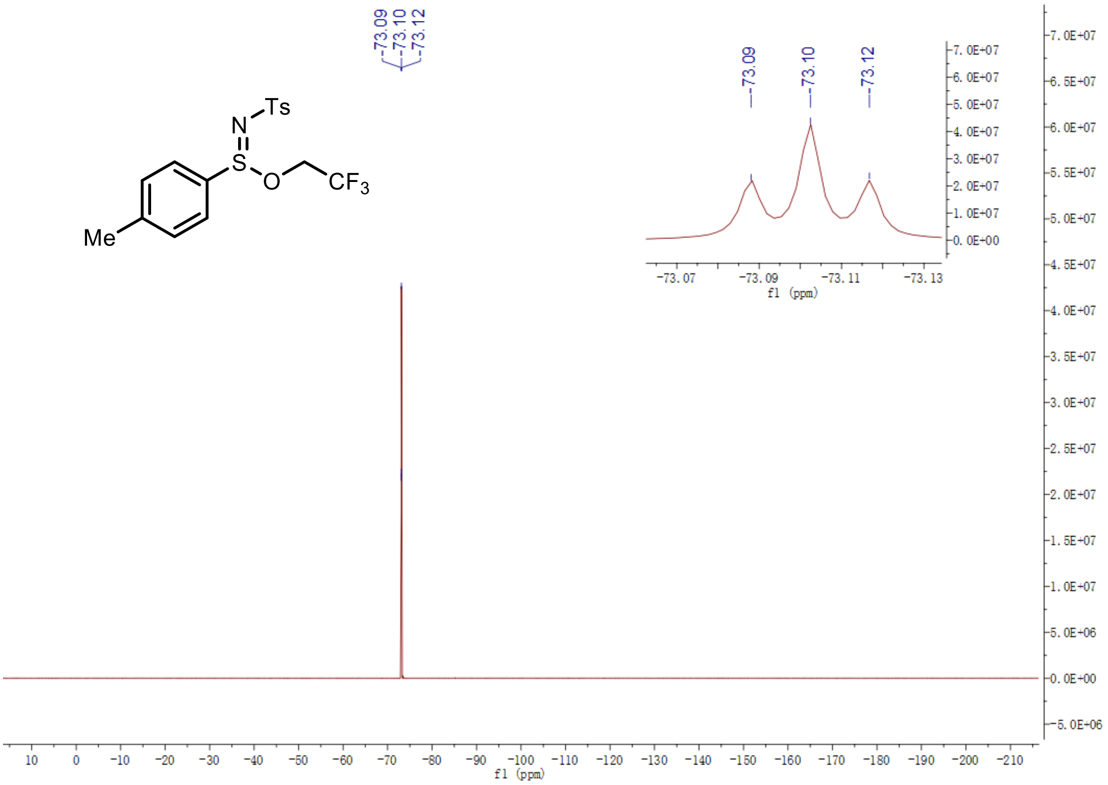

$^{1}H$ NMR spectrum of compound **6c** (600 MHz, $CDCl_3$)

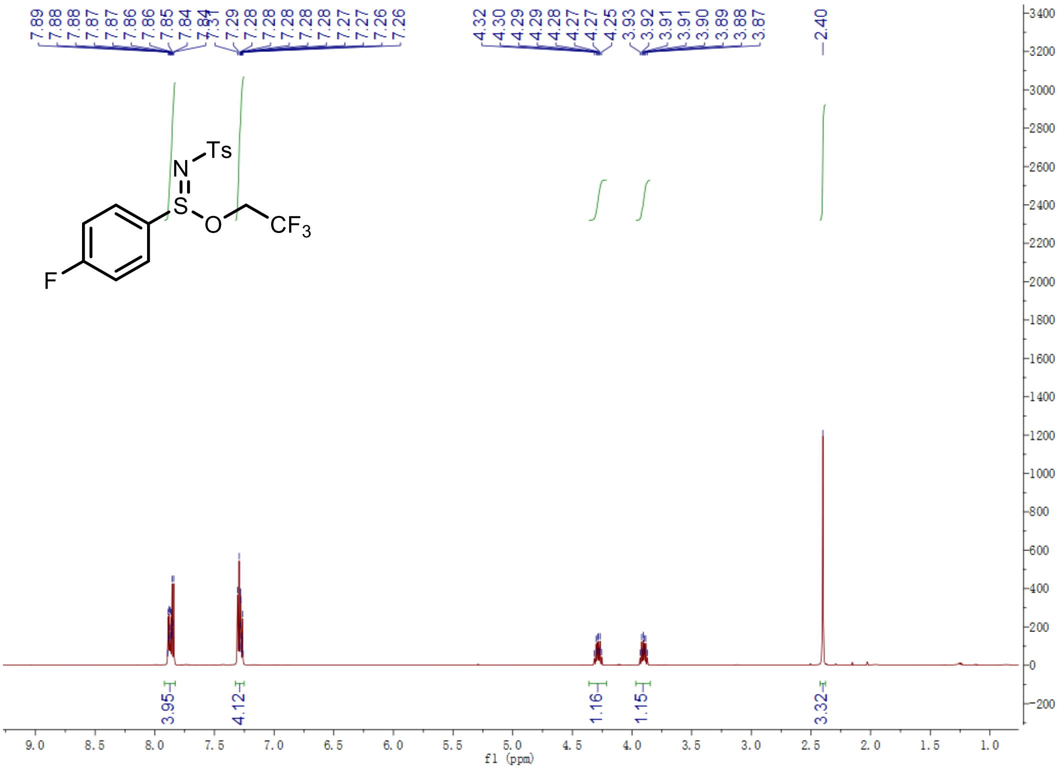

$^{13}C\{^{1}H\}$ NMR spectrum of compound **6c** (151 MHz, $CDCl_3$)

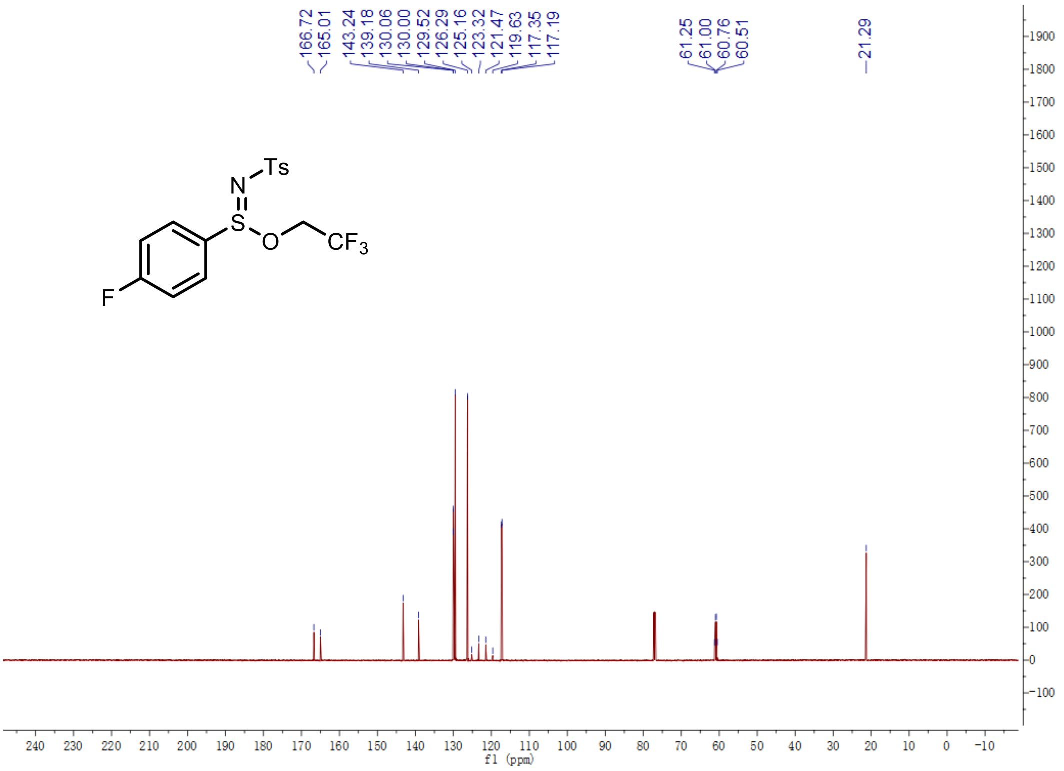

$^{19}$F NMR spectrum of compound **6c** (564 MHz, $CDCl_3$)

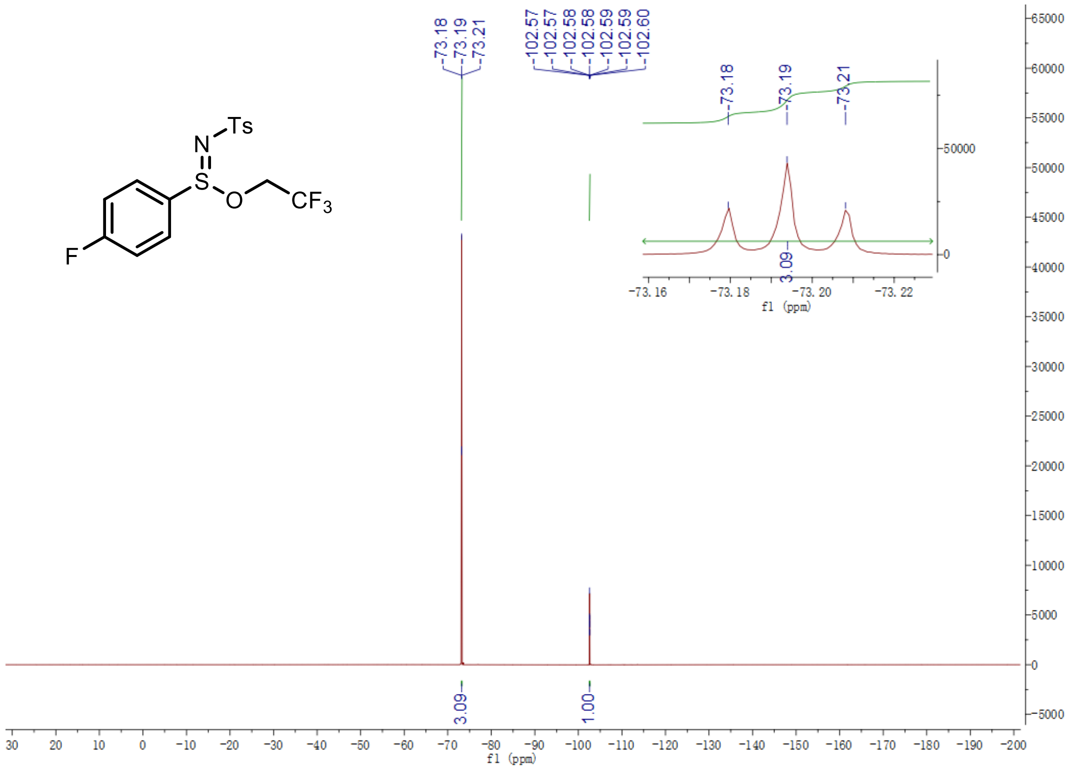


$^{1}$H NMR spectrum of compound **6d** (600 MHz, $CDCl_3$)

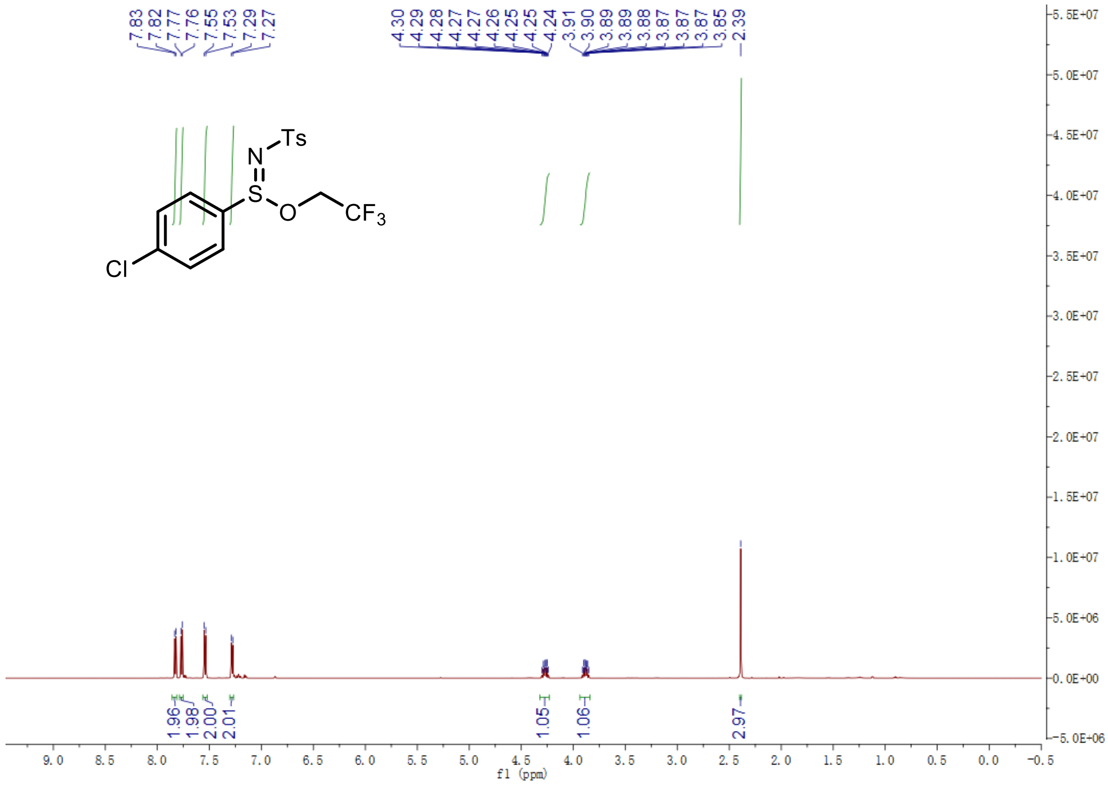

$^{13}C\{^{1}H\}$ NMR spectrum of compound **6d** (151 MHz, $CDCl_3$)

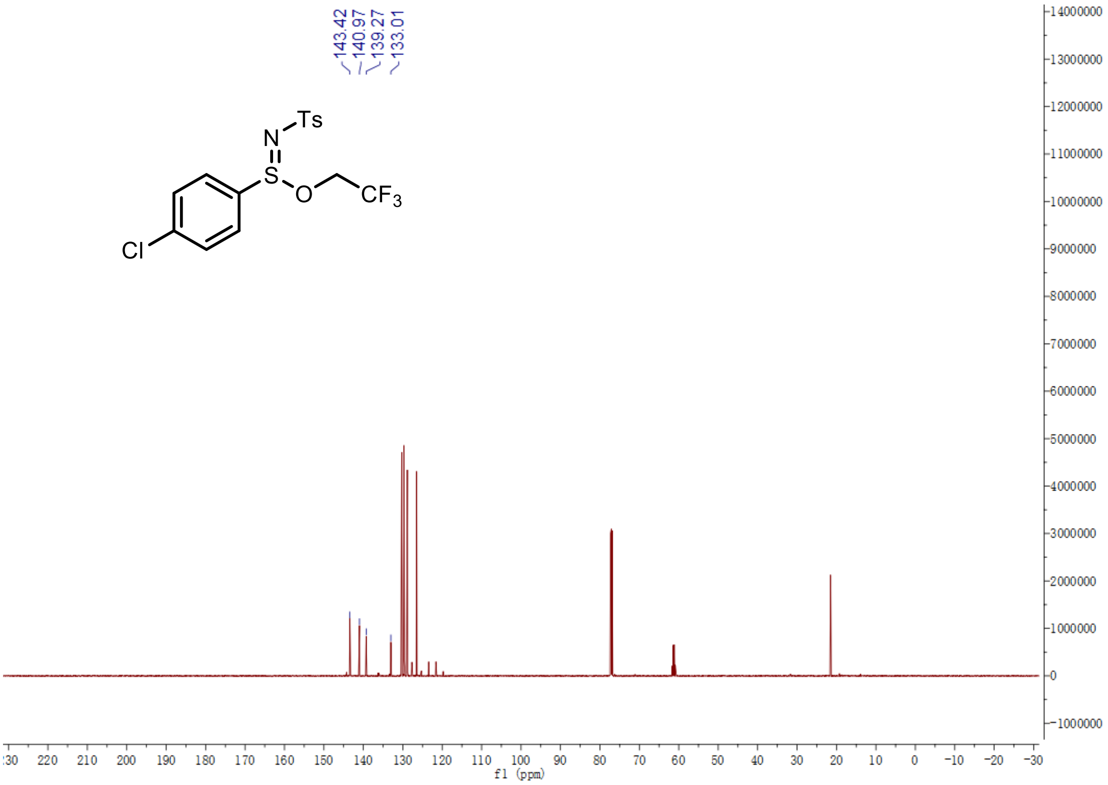


$^{19}F$ NMR spectrum of compound **6d** (564 MHz, $CDCl_3$)

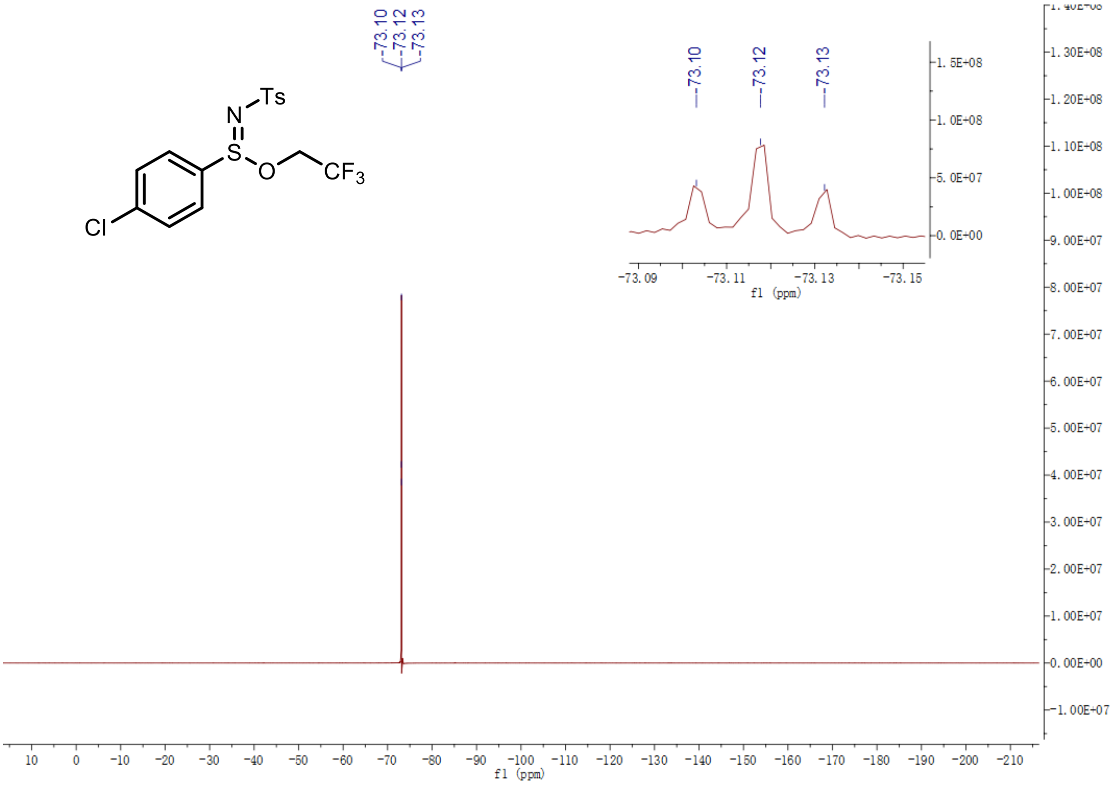

$^{1}H$ NMR spectrum of compound **6e** (600 MHz, $CDCl_3$)

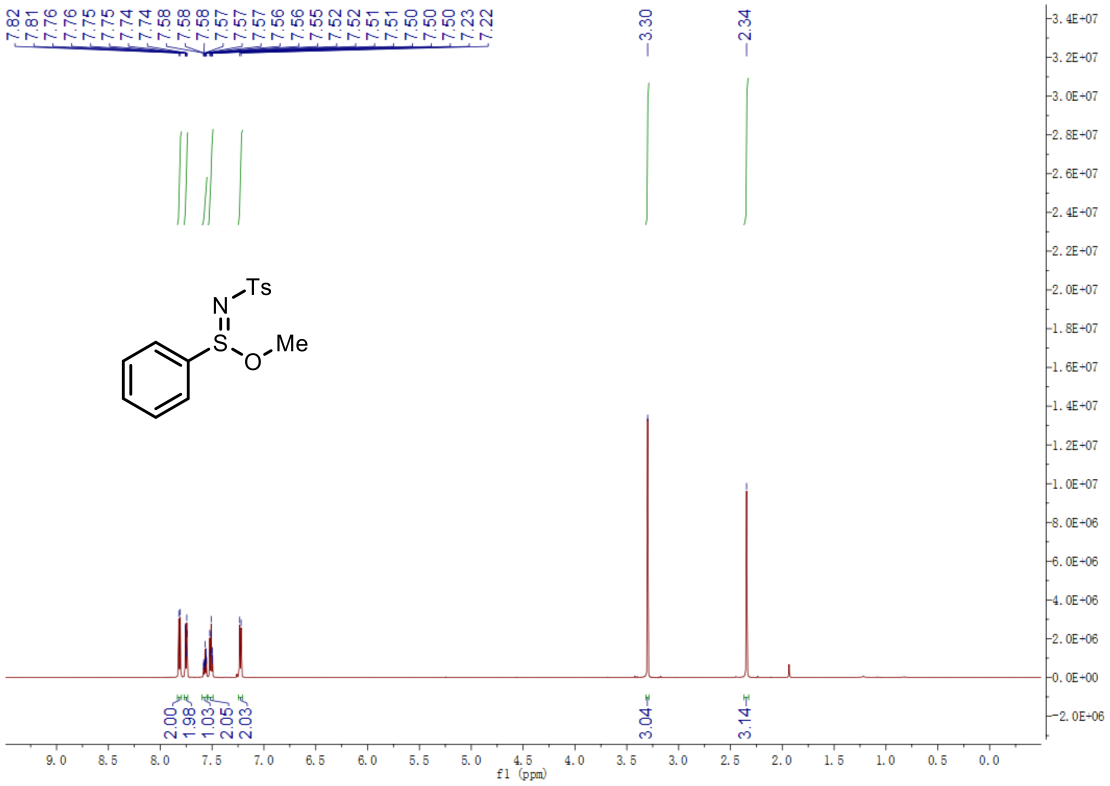


$^{13}C\{^{1}H\}$ NMR spectrum of compound **6e** (151 MHz, $CDCl_3$)

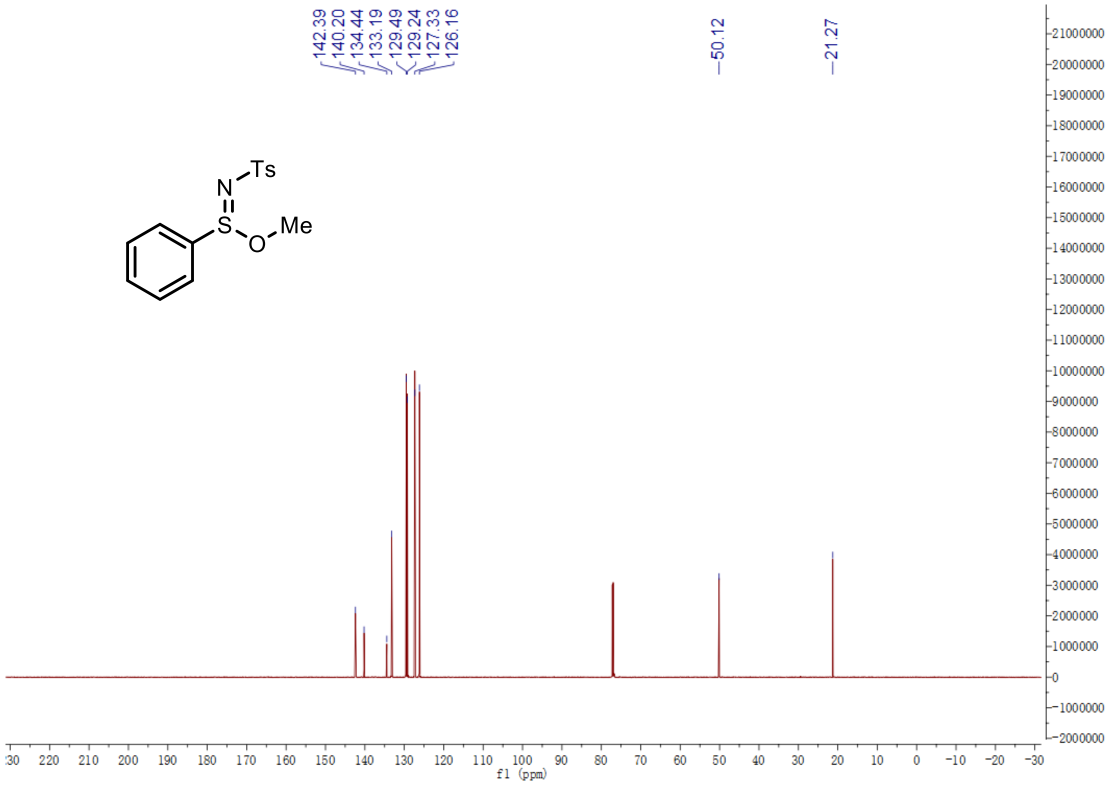

$^{1}$H NMR spectrum of compound **6f** (600 MHz, $CDCl_3$)

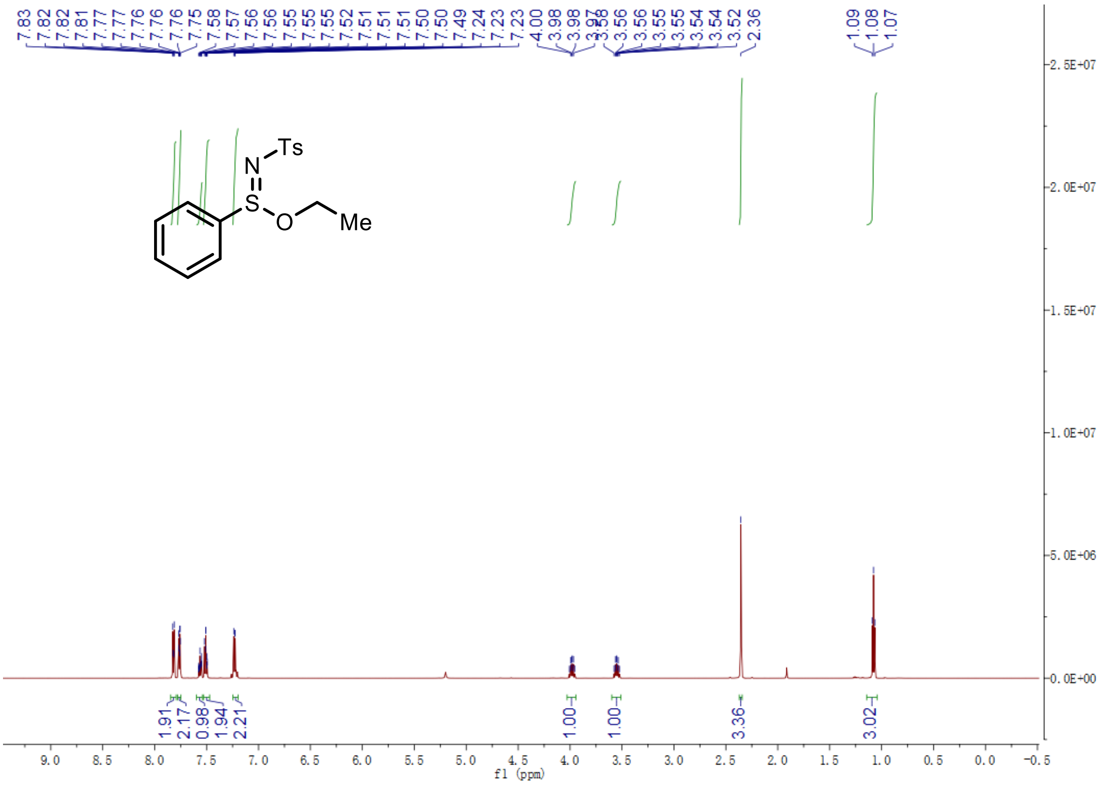


$^{13}$C{$^{1}$H} NMR spectrum of compound **6f** (151 MHz, $CDCl_3$)

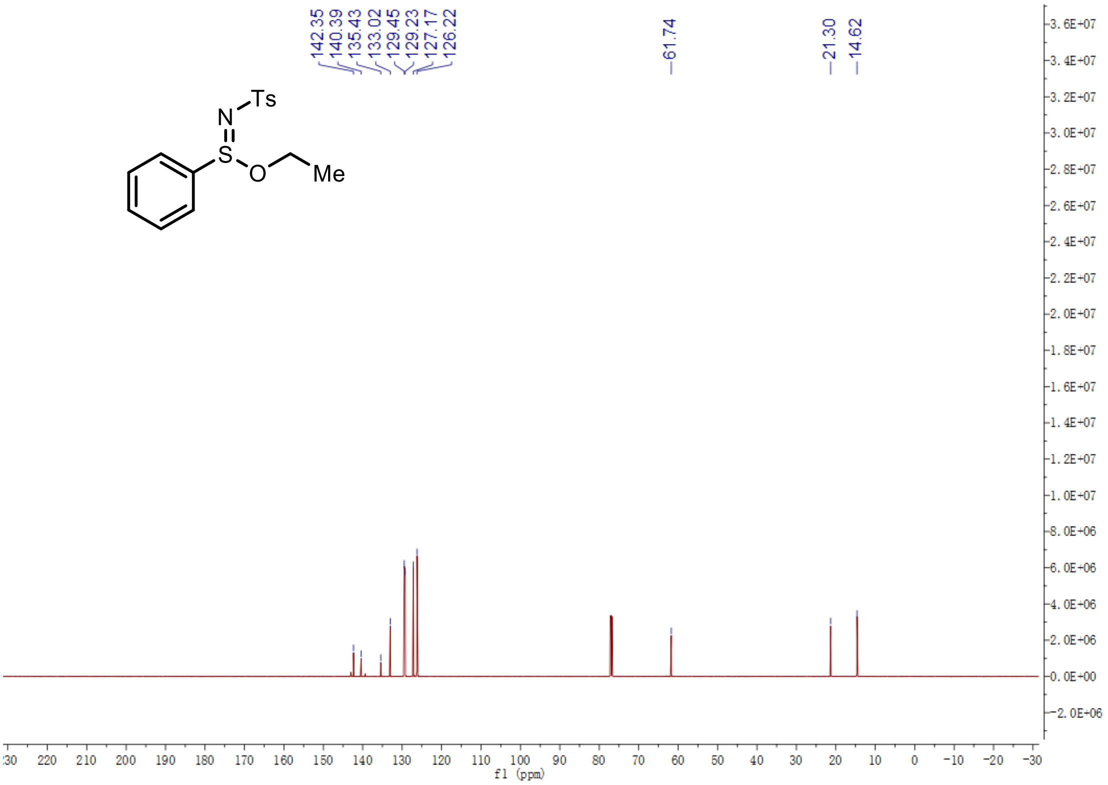

$^{1}$H NMR spectrum of compound **6g** (600 MHz, $CDCl_3$)

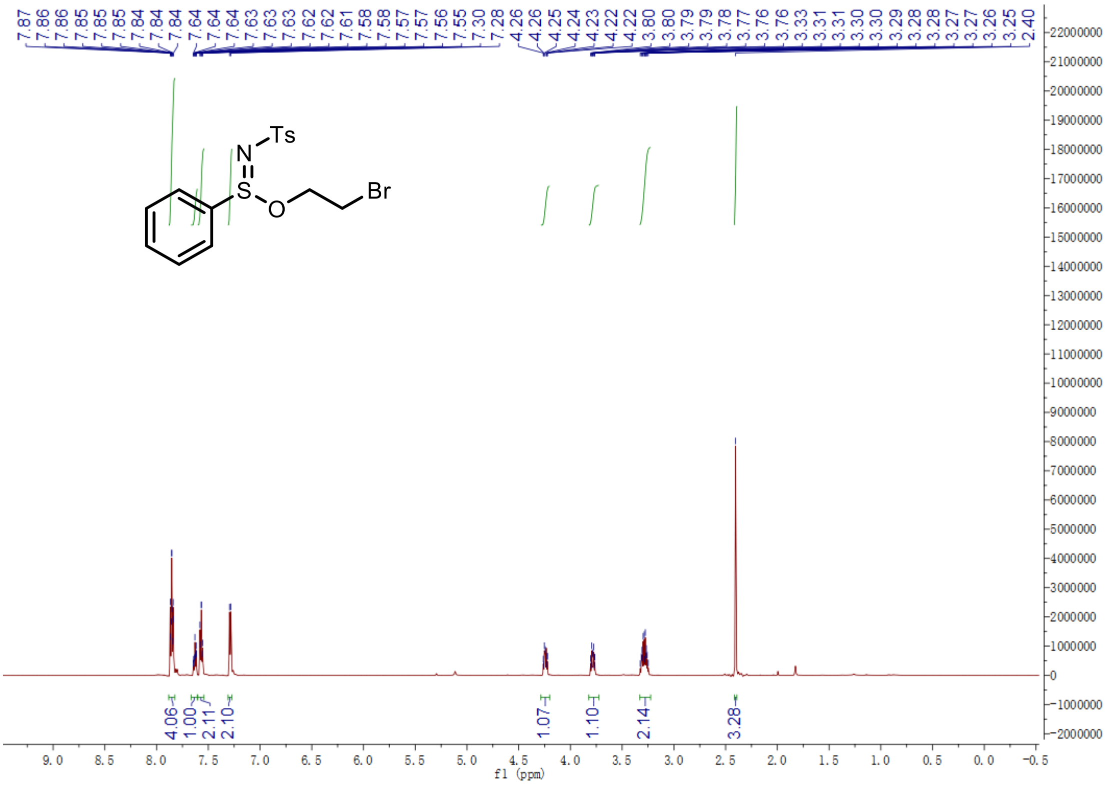


$^{13}$C{$^{1}$H} NMR spectrum of compound **6g** (151 MHz, $CDCl_3$)

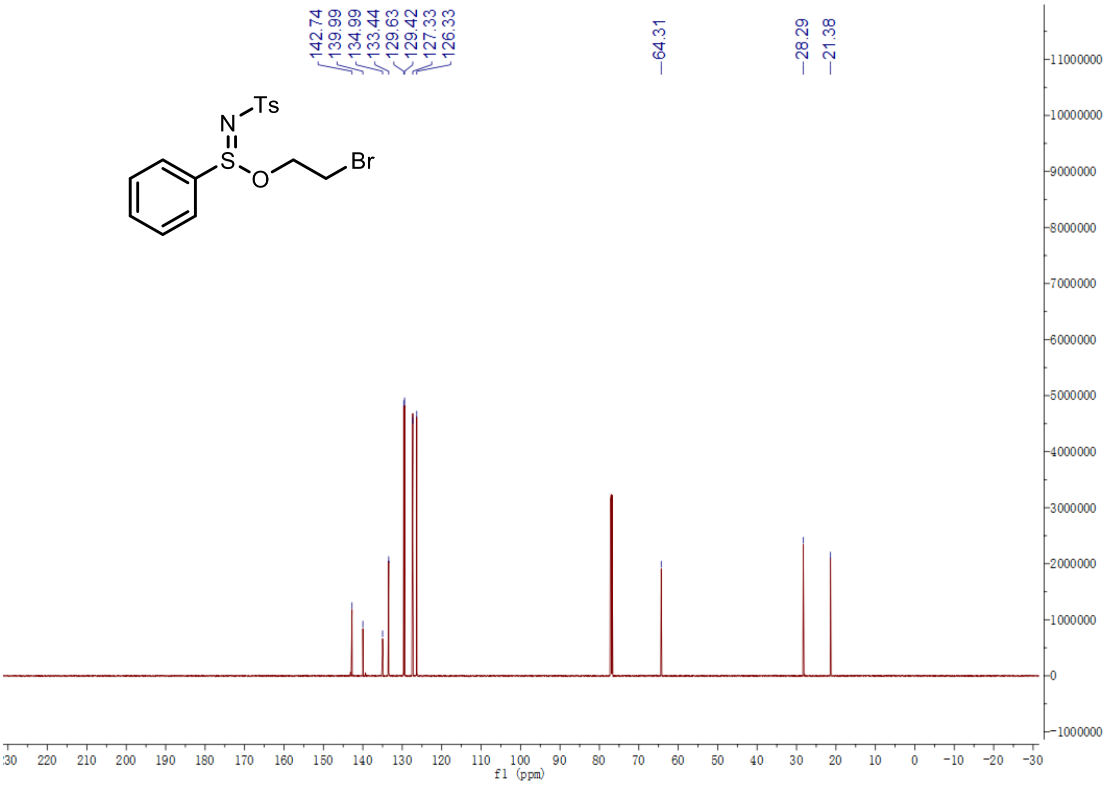

$^{1}H$ NMR spectrum of compound **6h** (400 MHz, $CDCl_3$)

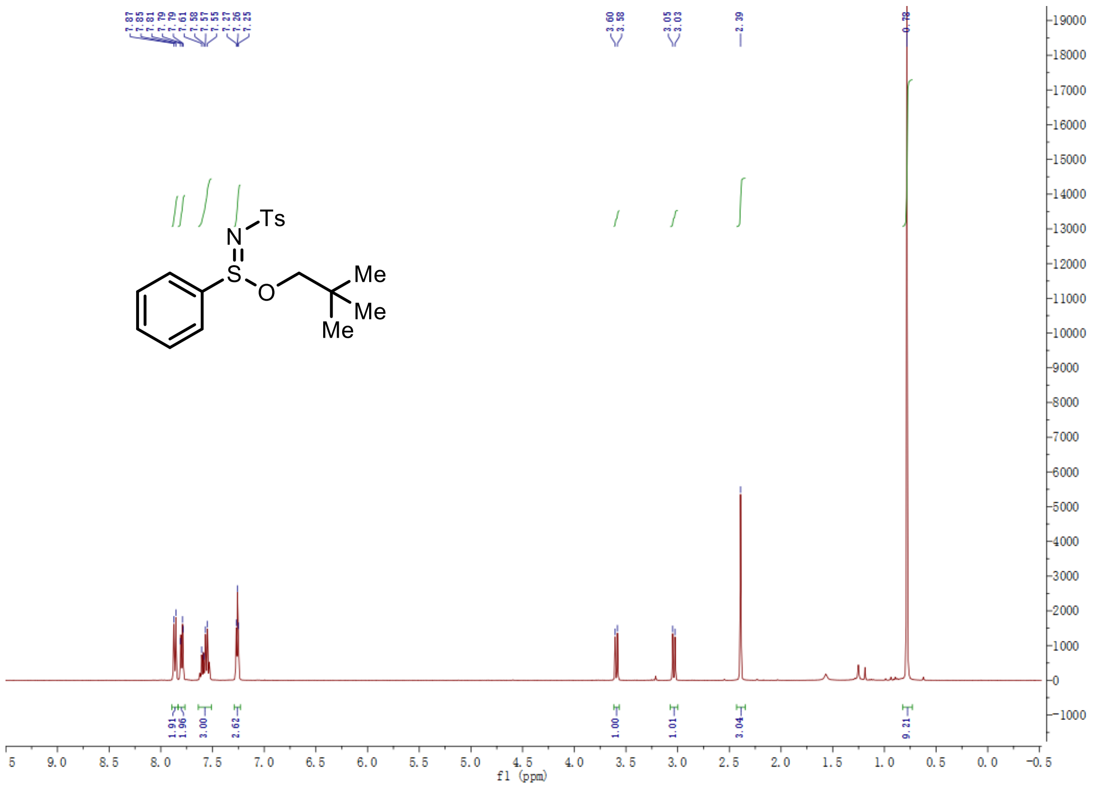


$^{13}C\{^{1}H\}$ NMR spectrum of compound **6h** (101 MHz, $CDCl_3$)

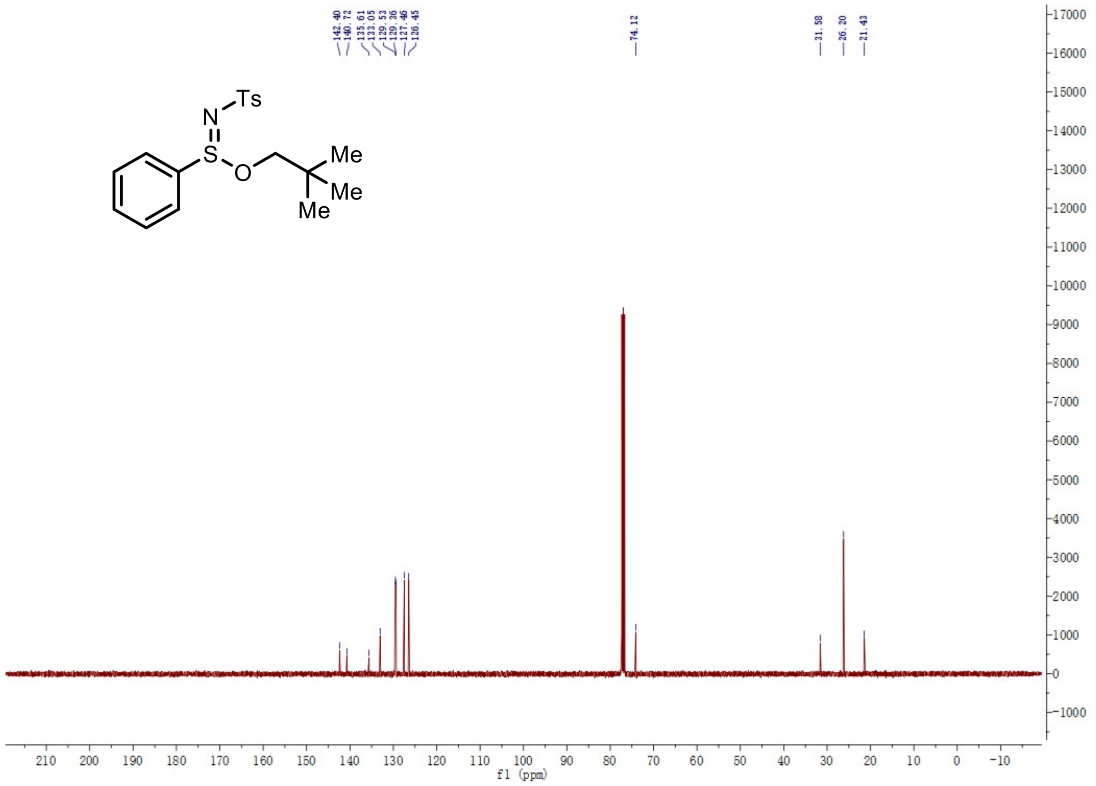

$^{1}H$ NMR spectrum of compound **6i** (400 MHz, $CDCl_3$)

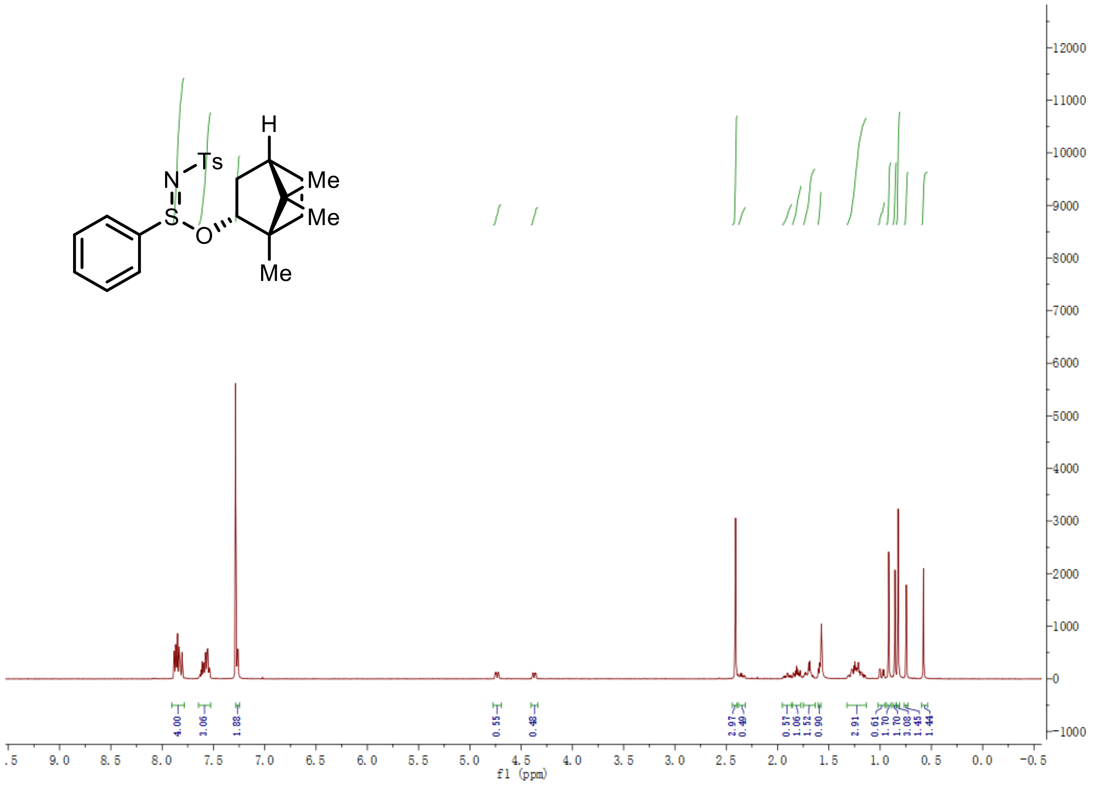


$^{13}C\{^{1}H\}$ NMR spectrum of compound **6i** (101 MHz, $CDCl_3$)

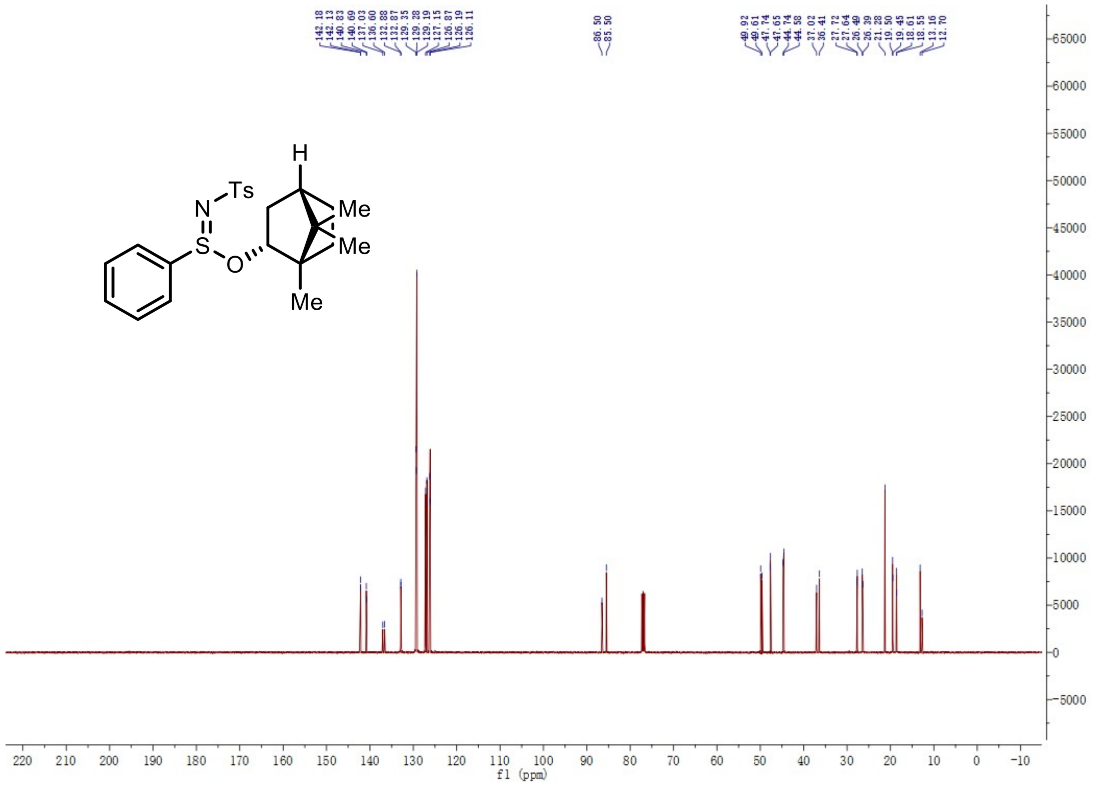

$^{1}$H NMR spectrum of compound **6j** (600 MHz, $CDCl_3$)

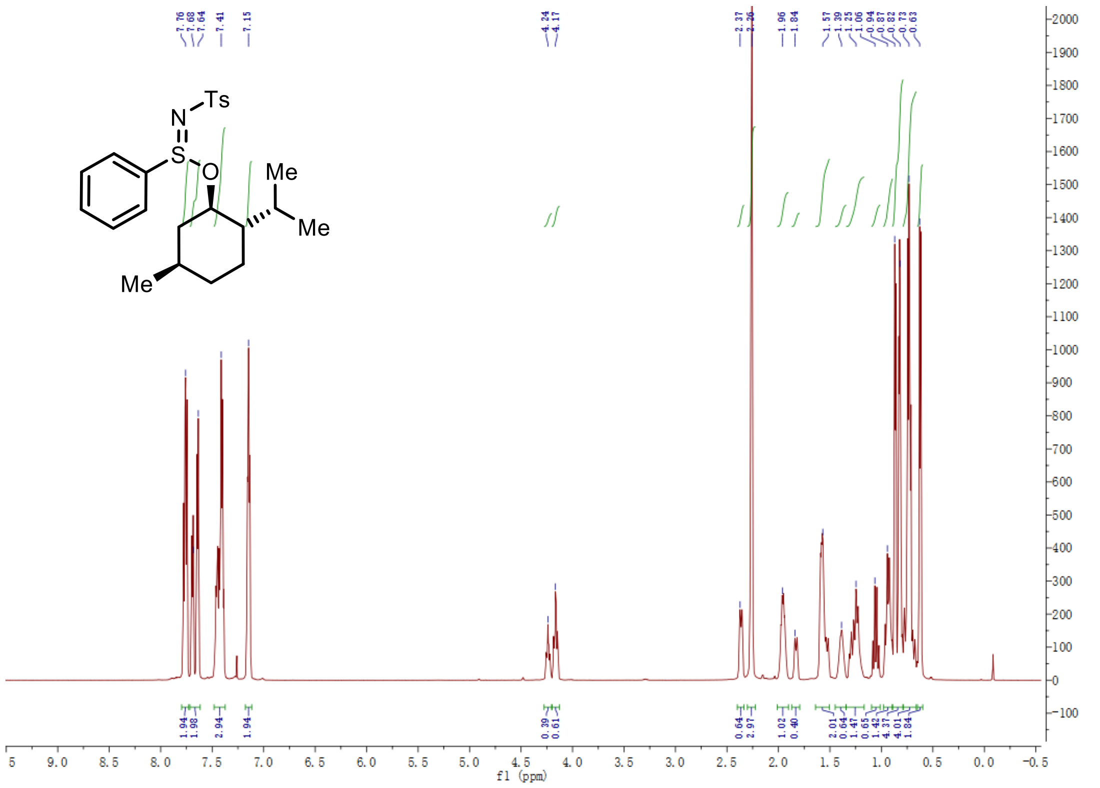

$^{13}$C{$^{1}$H} NMR spectrum of compound **6j** (151 MHz, $CDCl_3$)

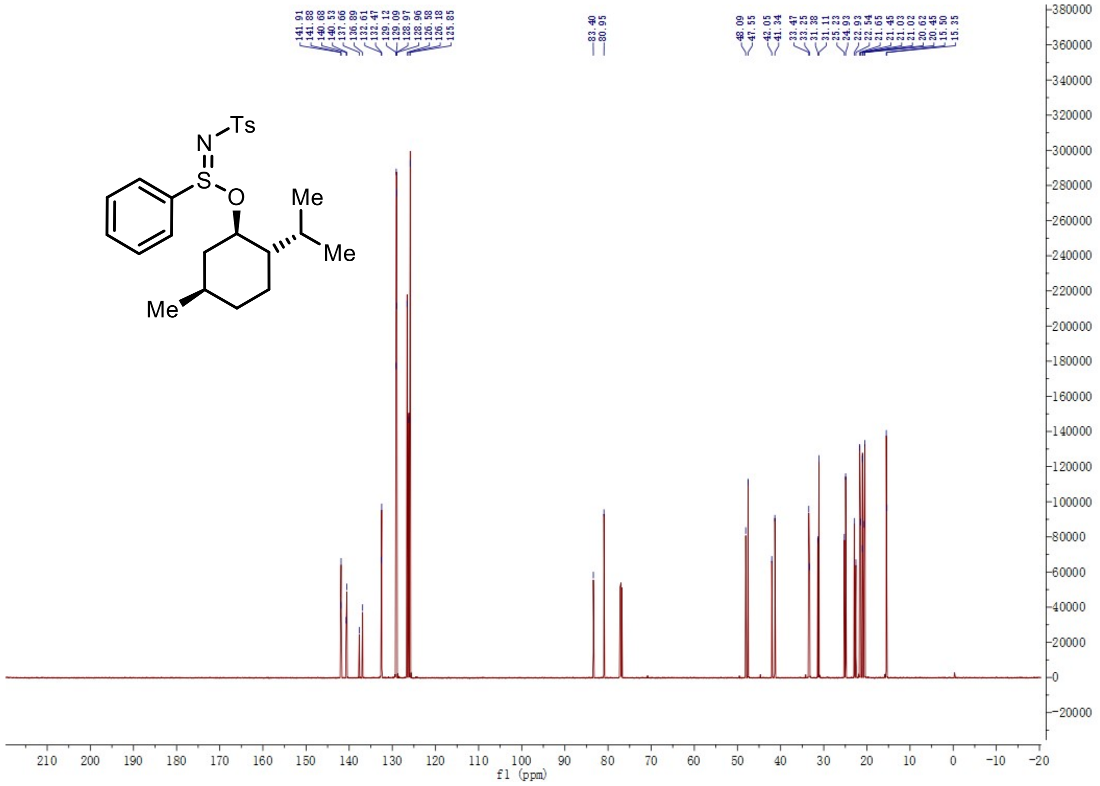

$^{1}$H NMR spectrum of compound **6k** (600 MHz, $CDCl_3$)

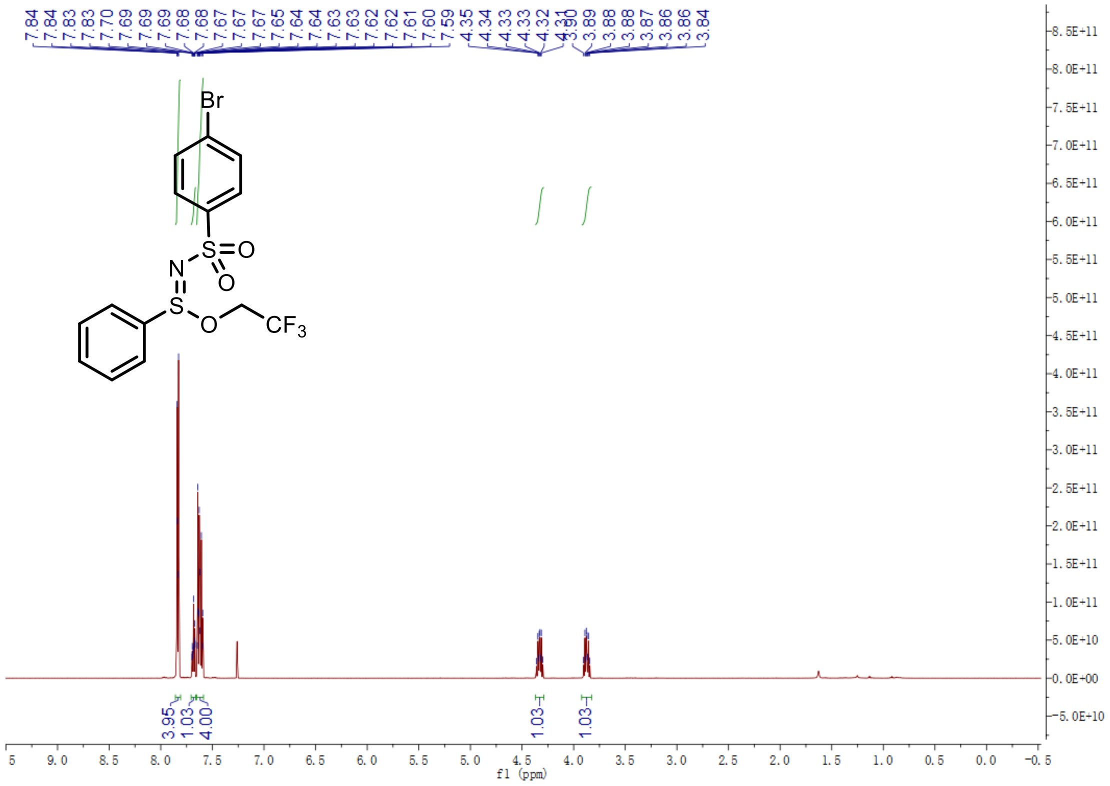

$^{13}C\{^1H\}$ NMR spectrum of compound **6k** (151 MHz, $CDCl_3$

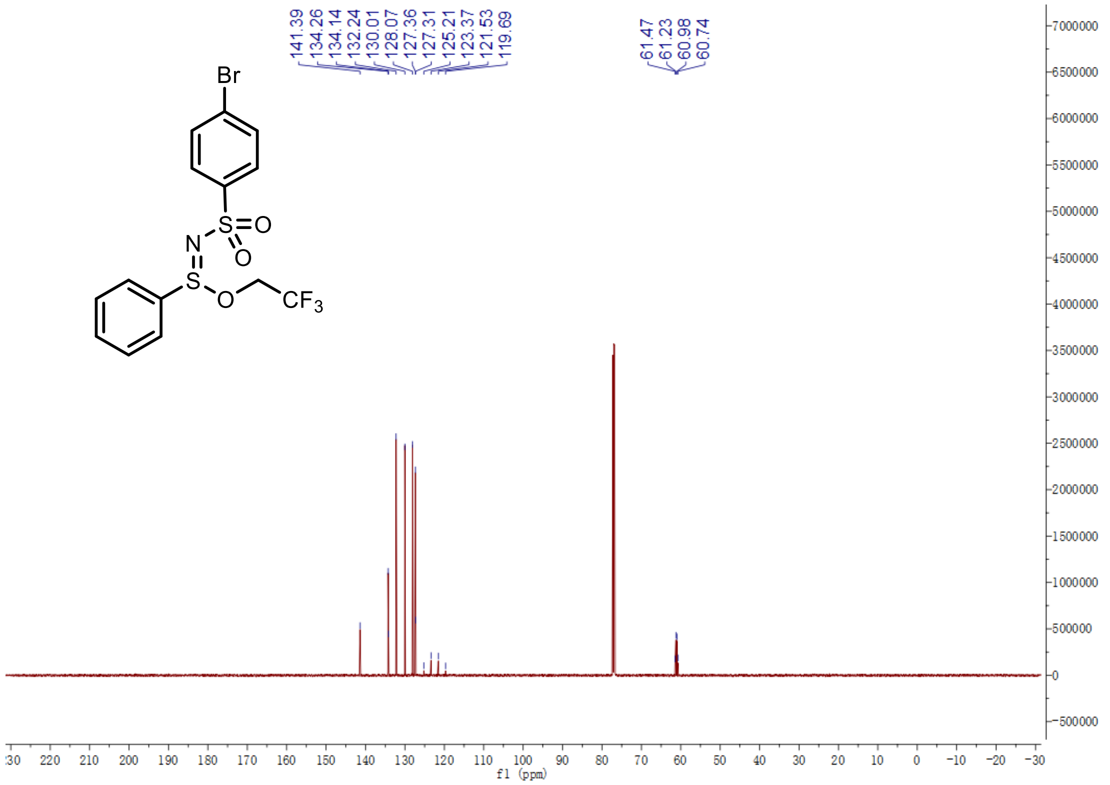

$^{19}$F NMR spectrum of compound **6k** (564 MHz, $CDCl_3$)

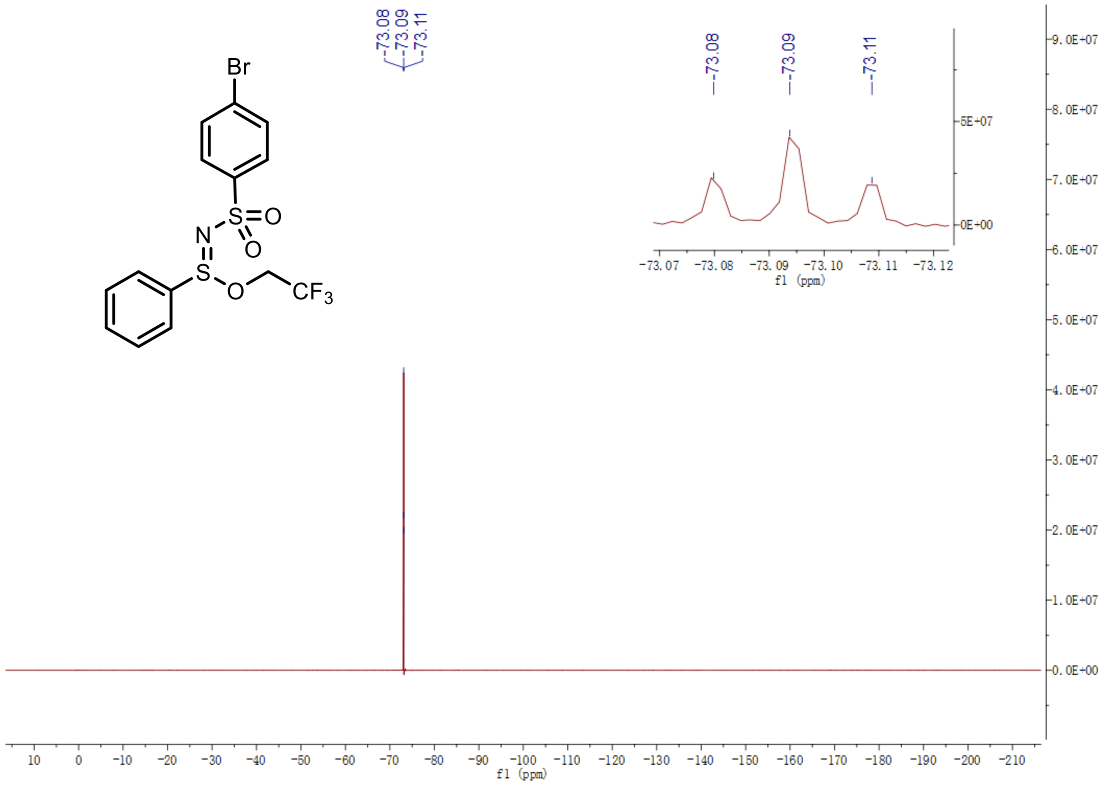

$^{1}$H NMR spectrum of compound **6l** (600 MHz, $CDCl_3$)

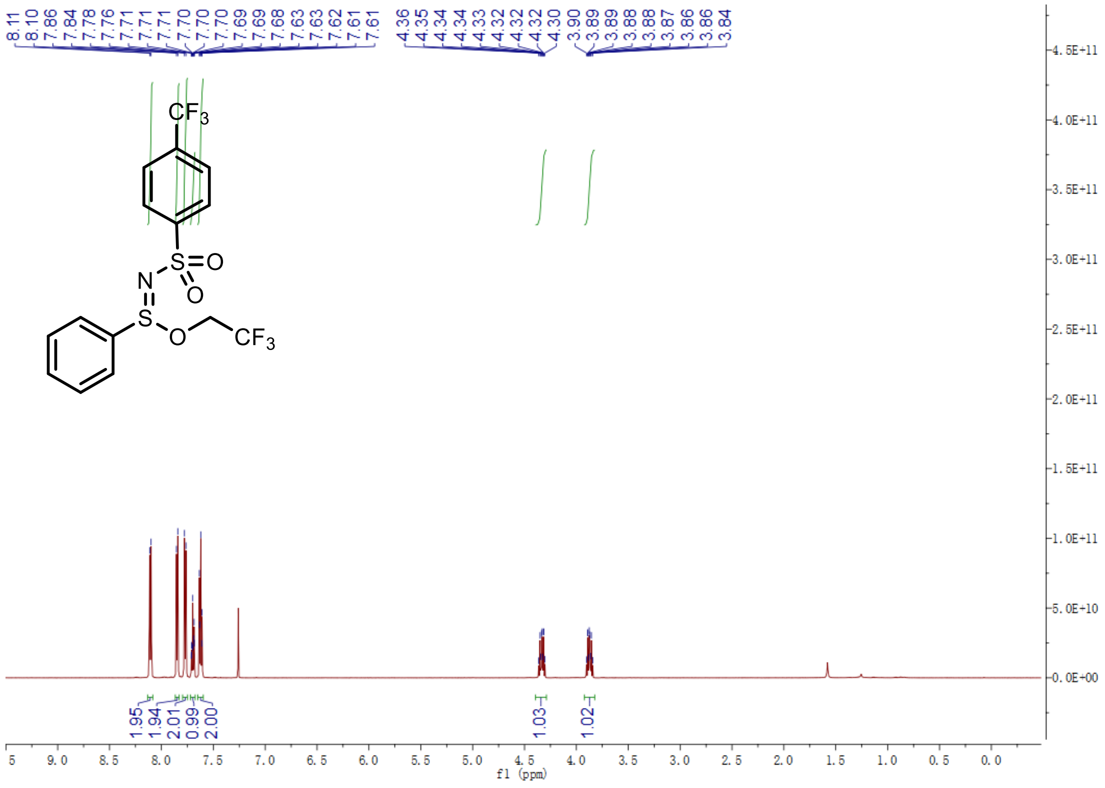

$^{13}C\{^{1}H\}$ NMR spectrum of compound **6l** (151 MHz, $CDCl_3$)

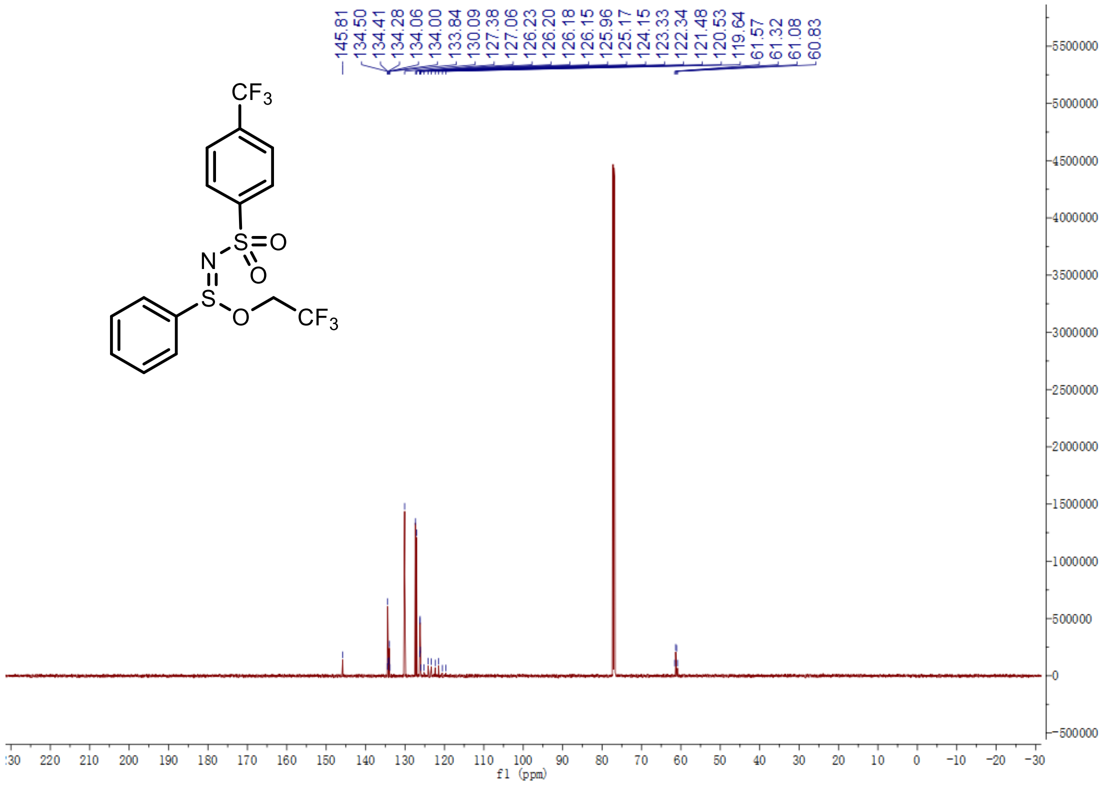


$^{19}F$ NMR spectrum of compound **6l** (564 MHz, $CDCl_3$)

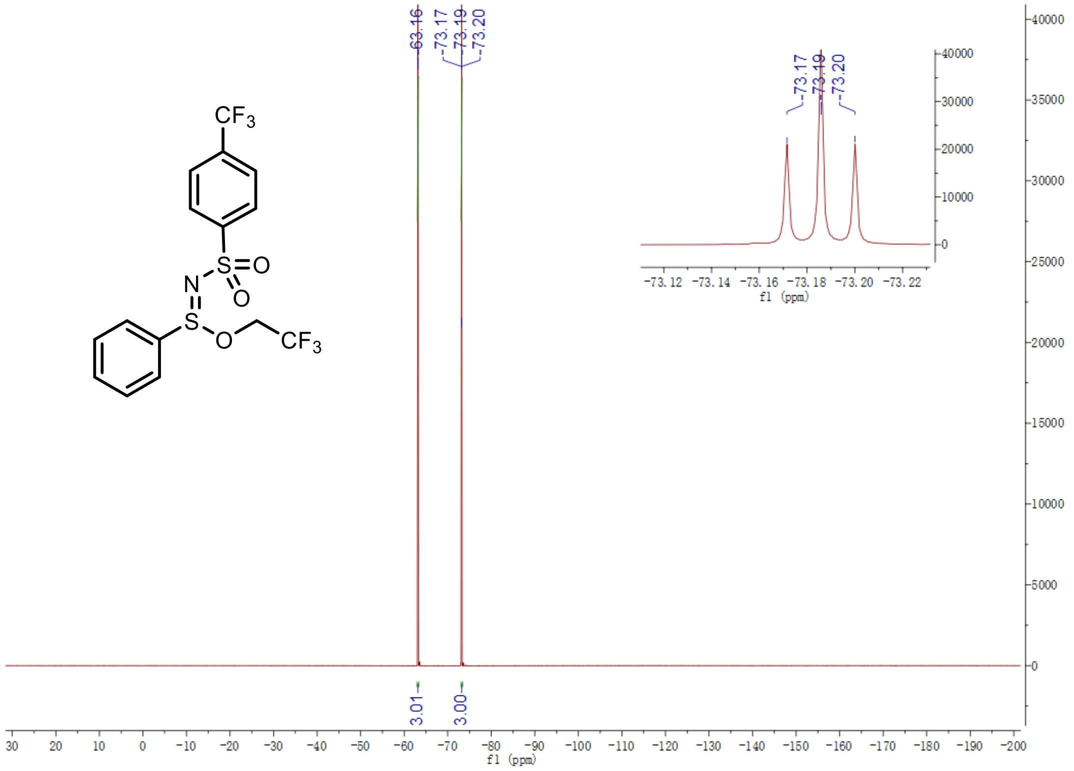

$^{1}H$ NMR spectrum of compound **6m** (600 MHz, $CDCl_3$)

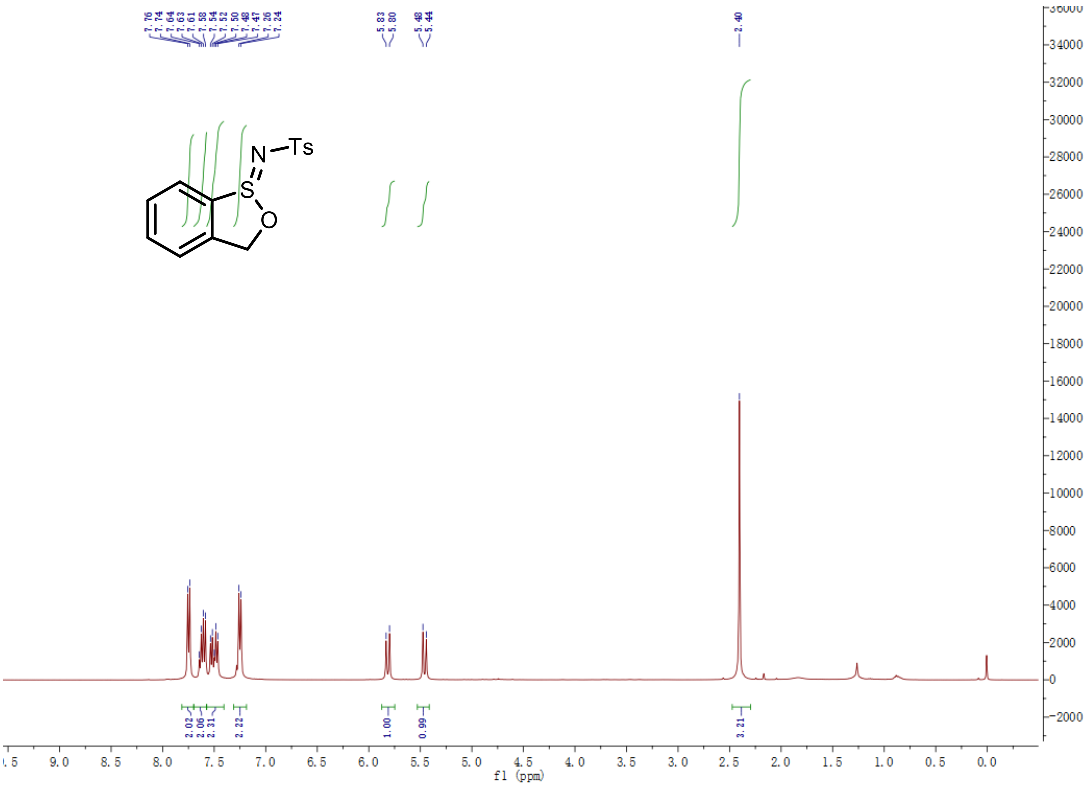

$^{13}C\{^{1}H\}$ NMR spectrum of compound **6m** (151 MHz, $CDCl_3$)

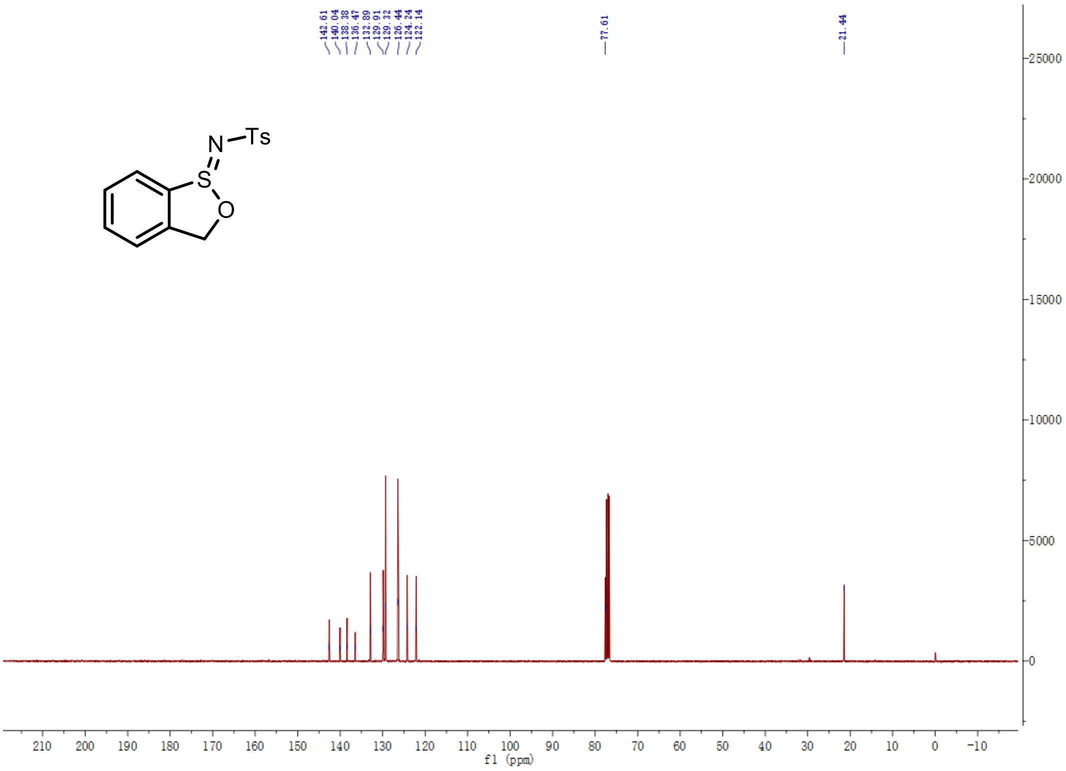

$^{1}$H NMR spectrum of compound **7a** (600 MHz, $CDCl_3$)

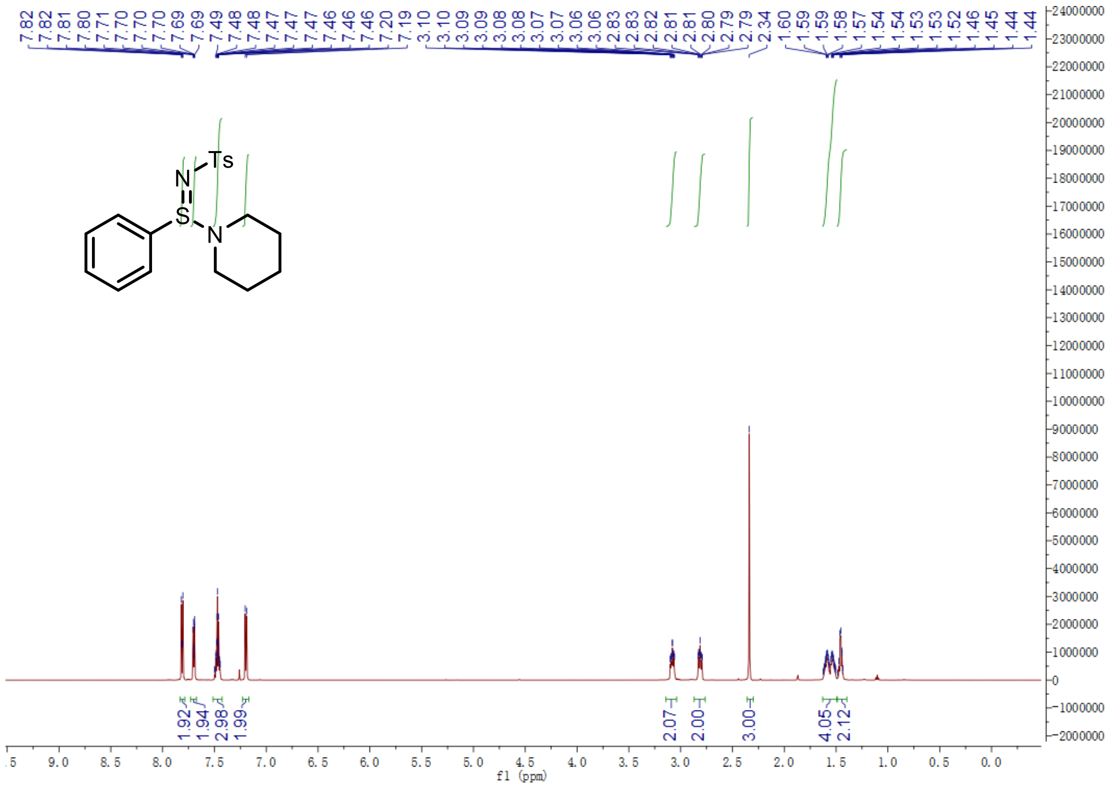

$^{13}$C{$^{1}$H} NMR spectrum of compound **7a** (151 MHz, $CDCl_3$)

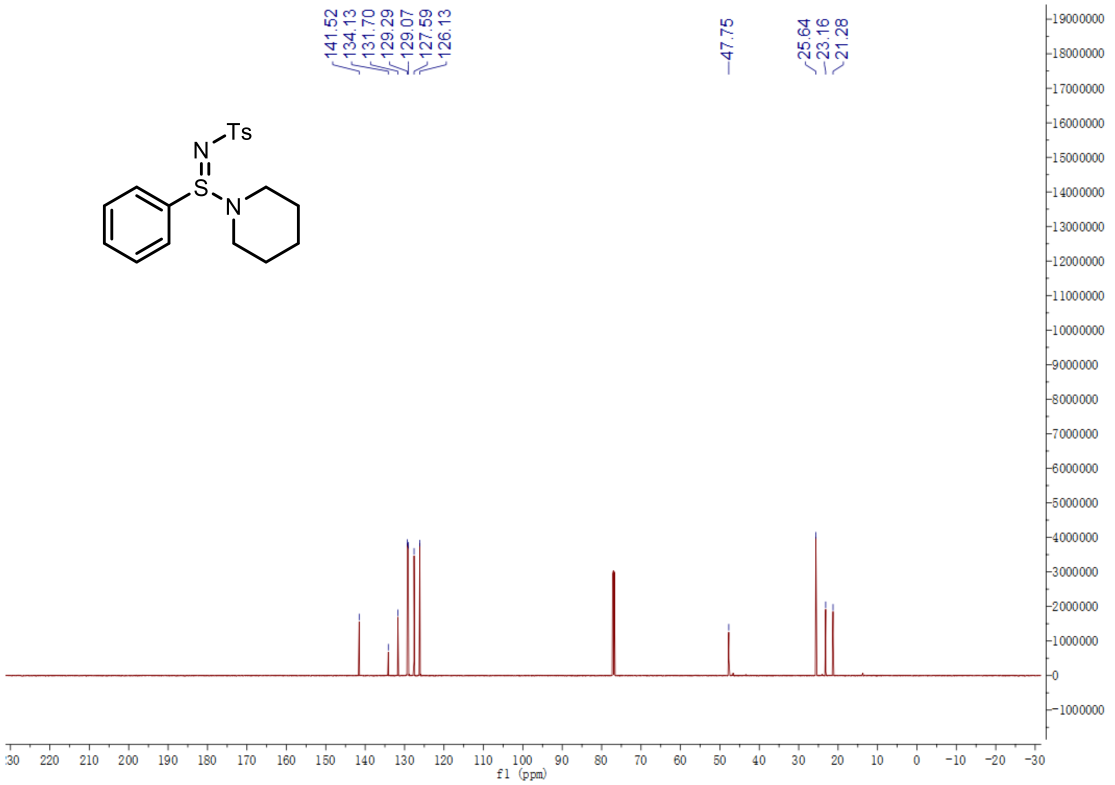

$^{1}$H NMR spectrum of compound **7b** (600 MHz, $CDCl_3$)

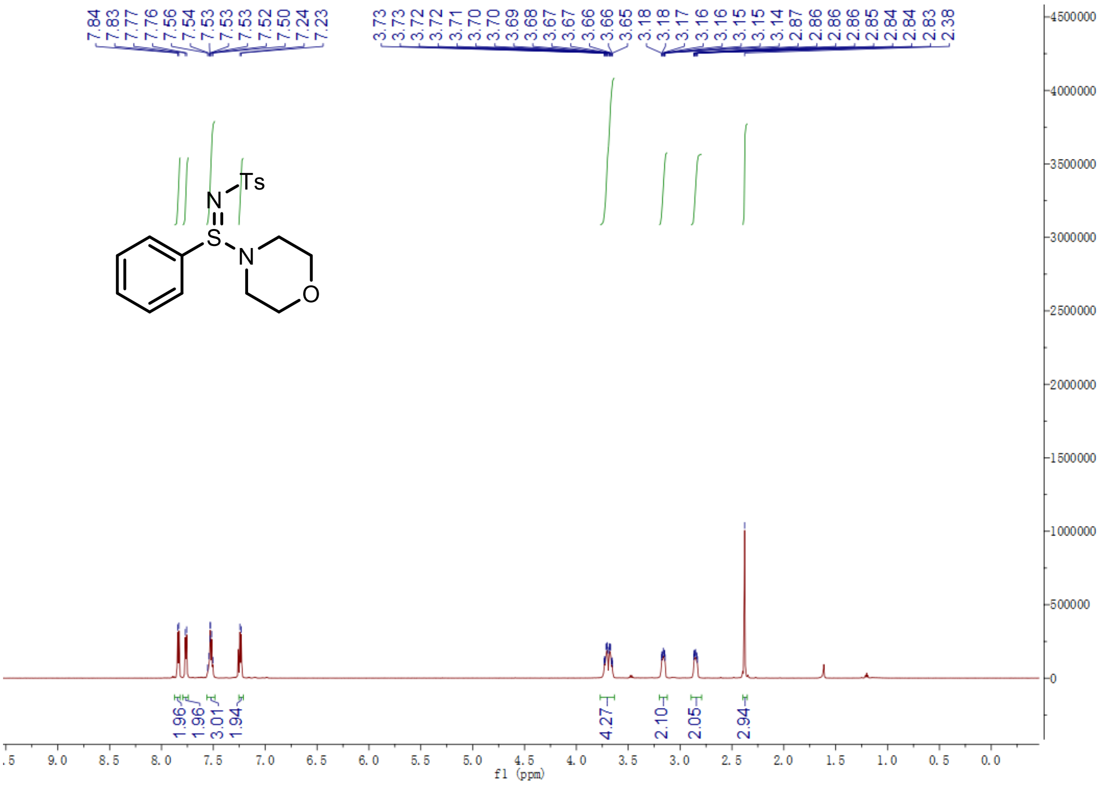


$^{13}$C{$^{1}$H} NMR spectrum of compound **7b** (151 MHz, $CDCl_3$)

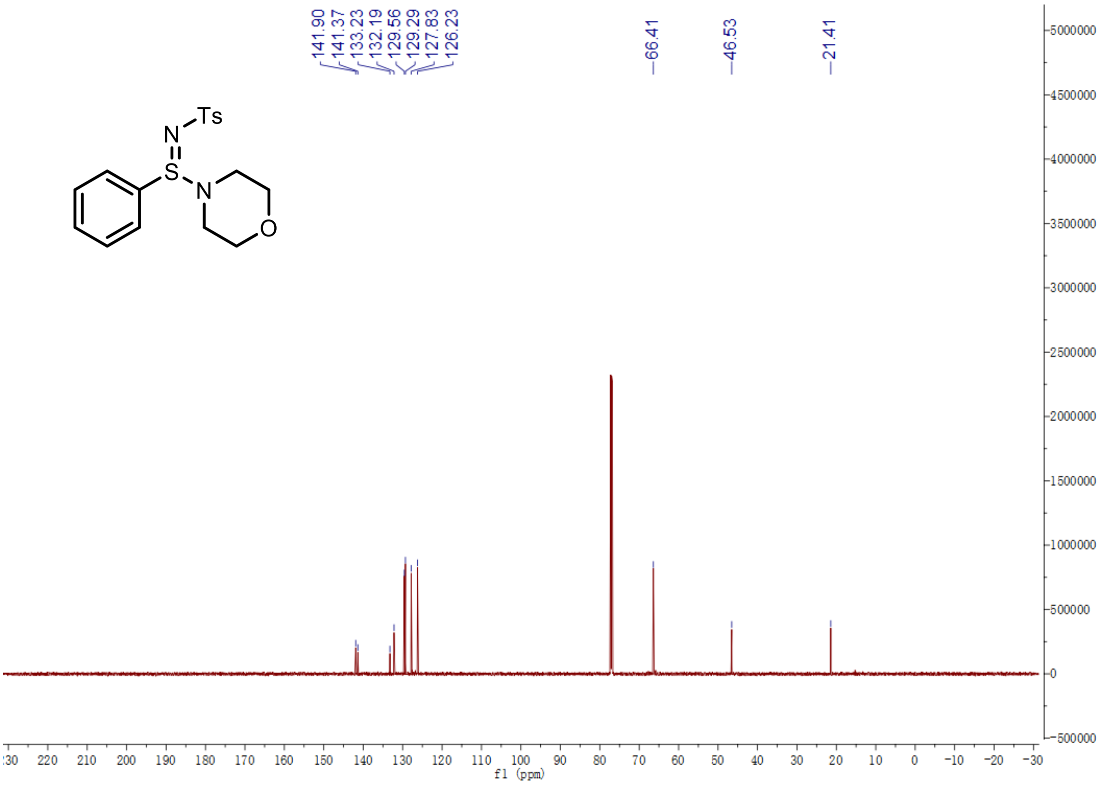

$^{1}H$ NMR spectrum of compound **7c** (600 MHz, $CDCl_3$)

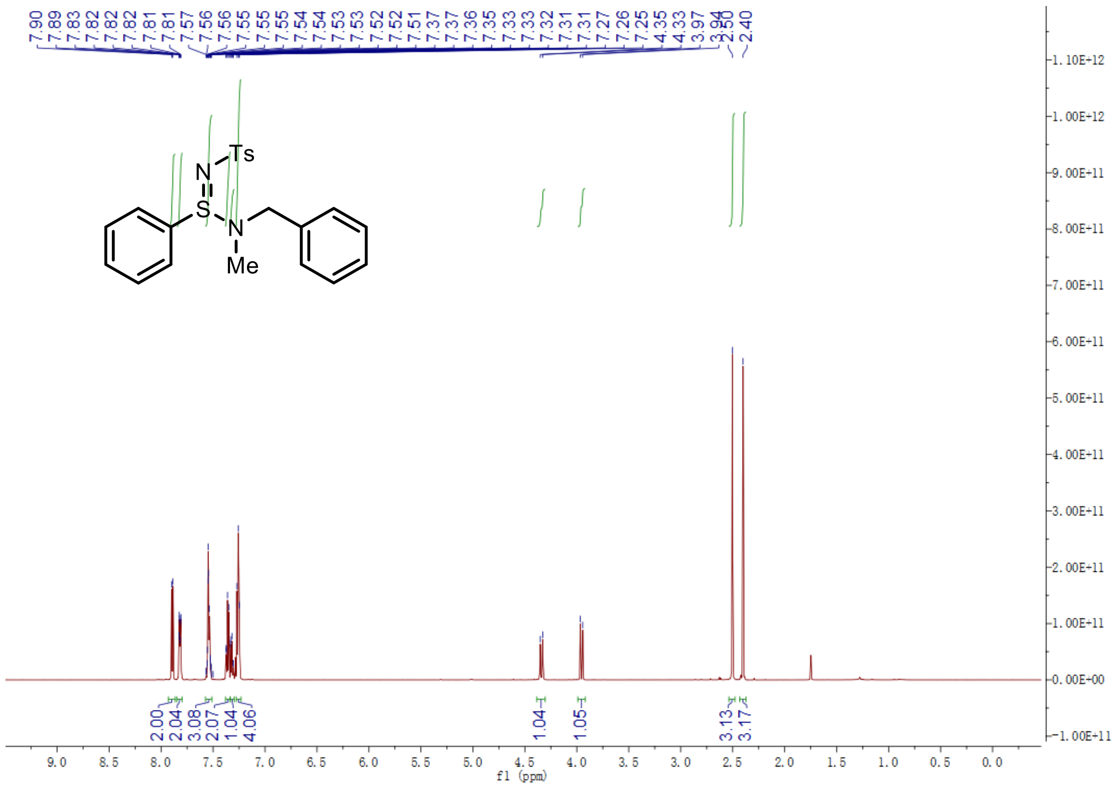

$^{13}C\{^{1}H\}$ NMR spectrum of compound **7c** (151 MHz, $CDCl_3$)

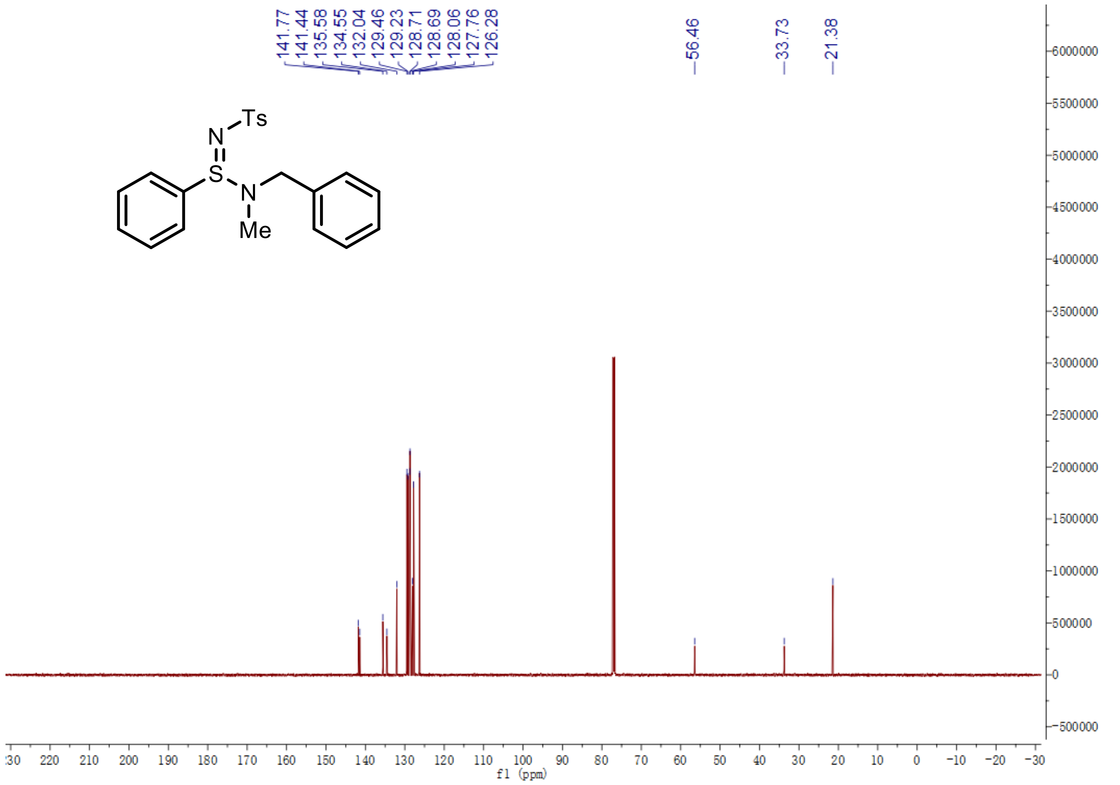

$^{1}$H NMR spectrum of compound **7d** (600 MHz, $CDCl_3$)

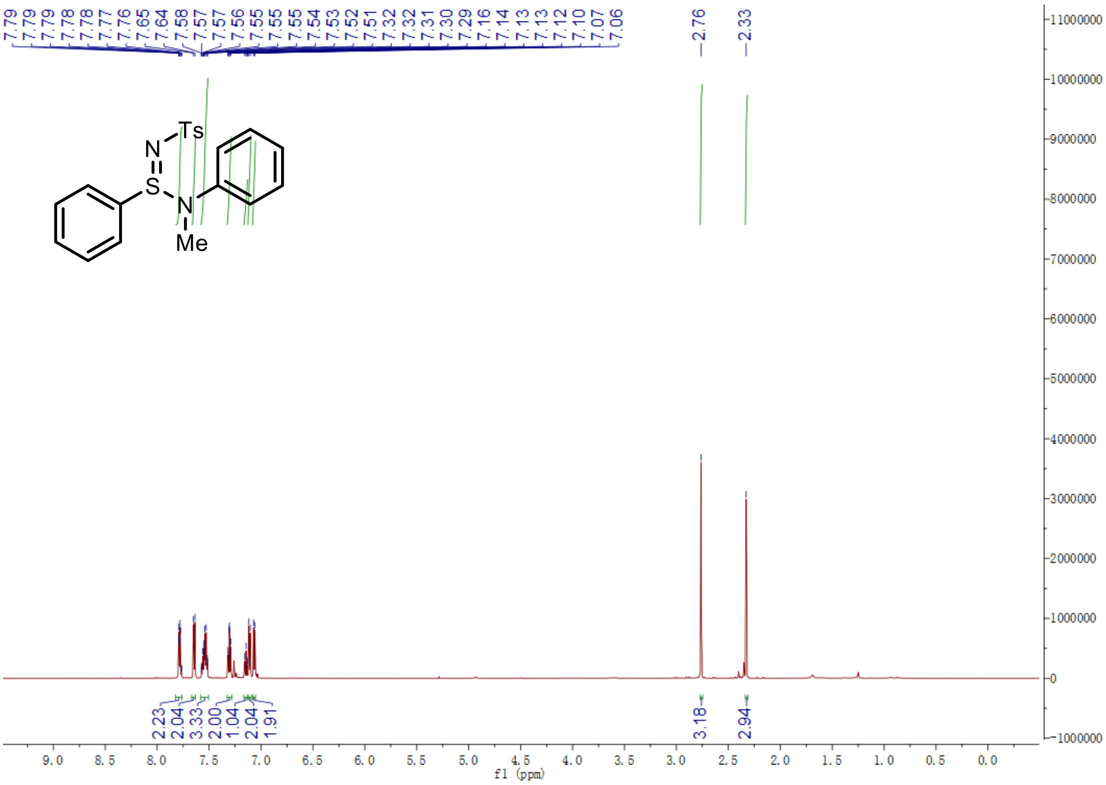

$^{13}$C{$^{1}$H} NMR spectrum of compound **7d** (151 MHz, $CDCl_3$)

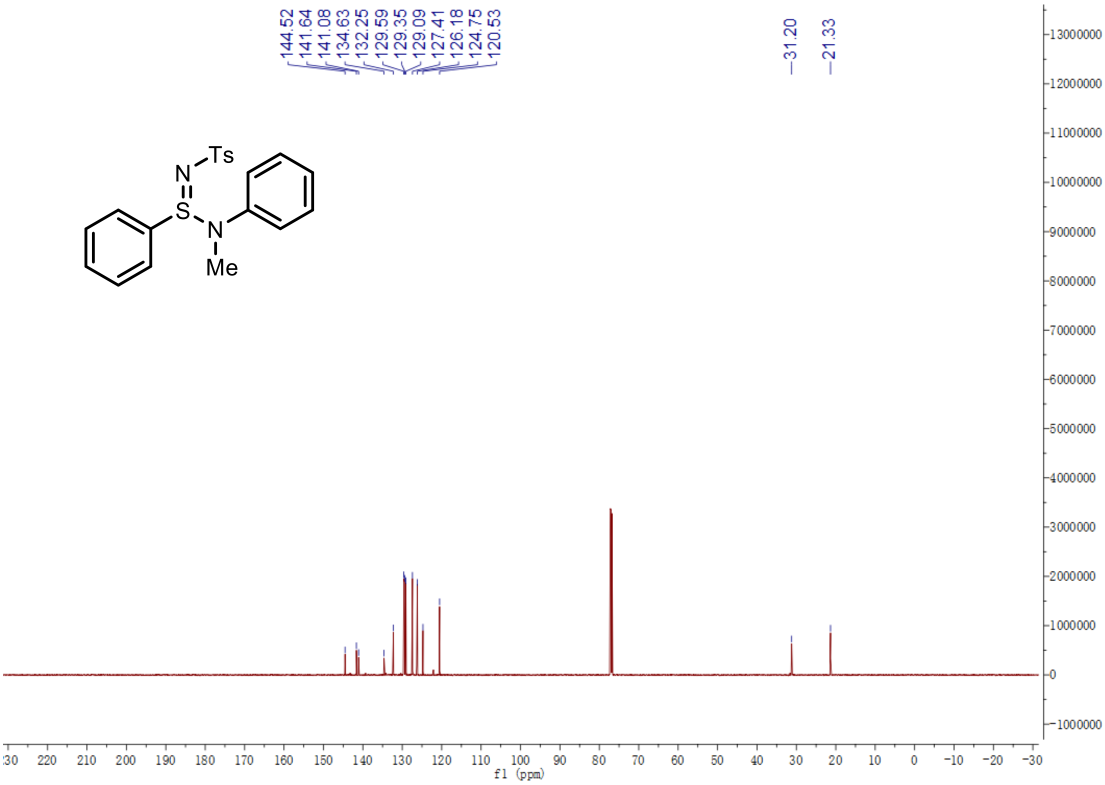

## $^{1}$H NMR spectrum of compound **7e** (600 MHz, $CDCl_3$)

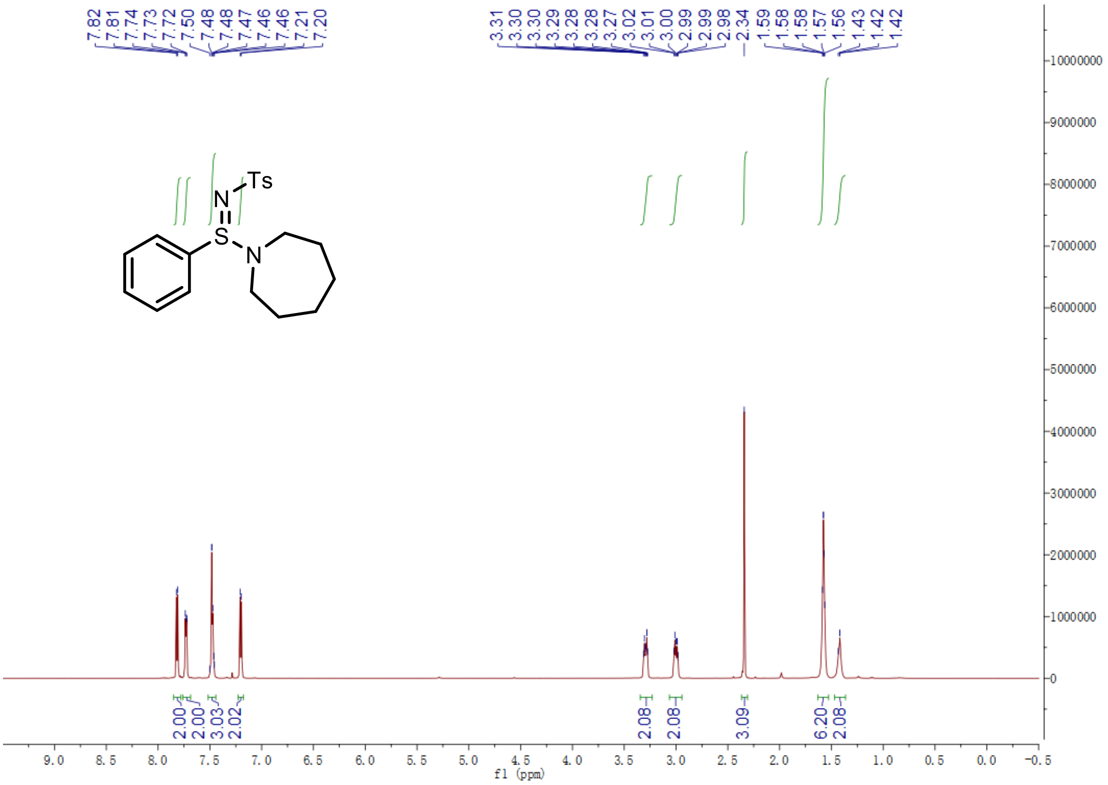


## $^{13}$C{$^{1}$H} NMR spectrum of compound **7e** (151 MHz, $CDCl_3$)

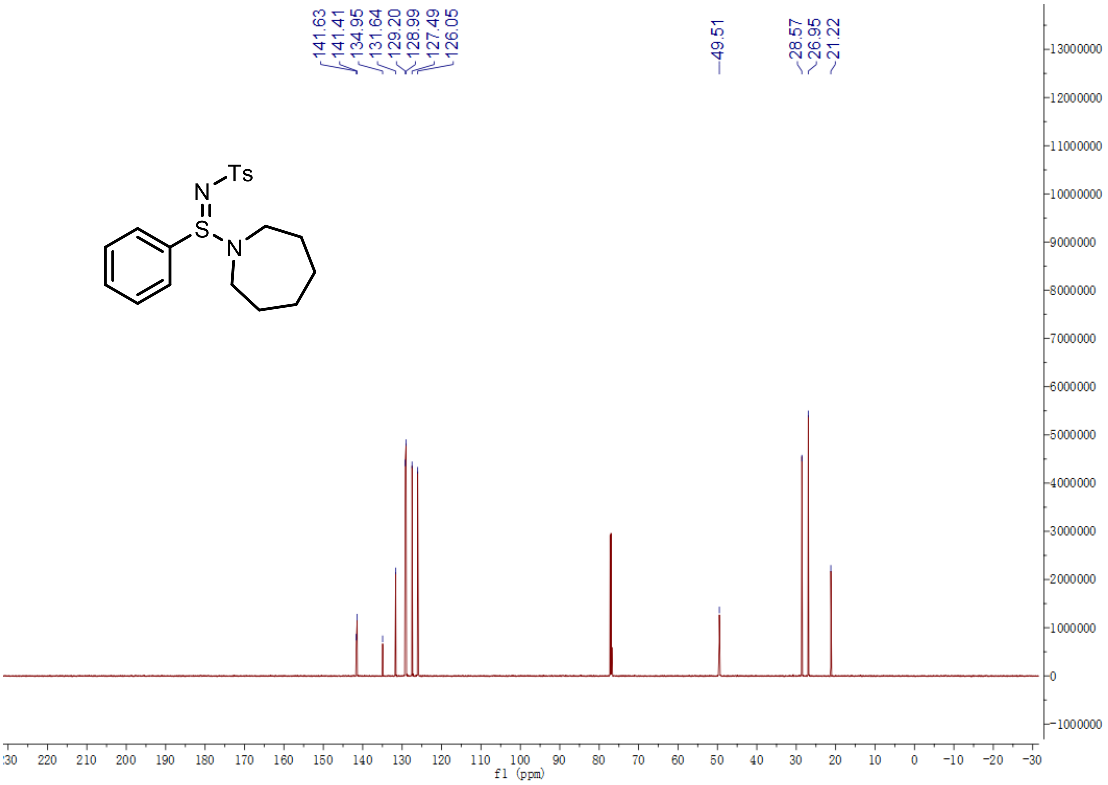

$^{1}$H NMR spectrum of compound **7f** (600 MHz, $CDCl_3$)

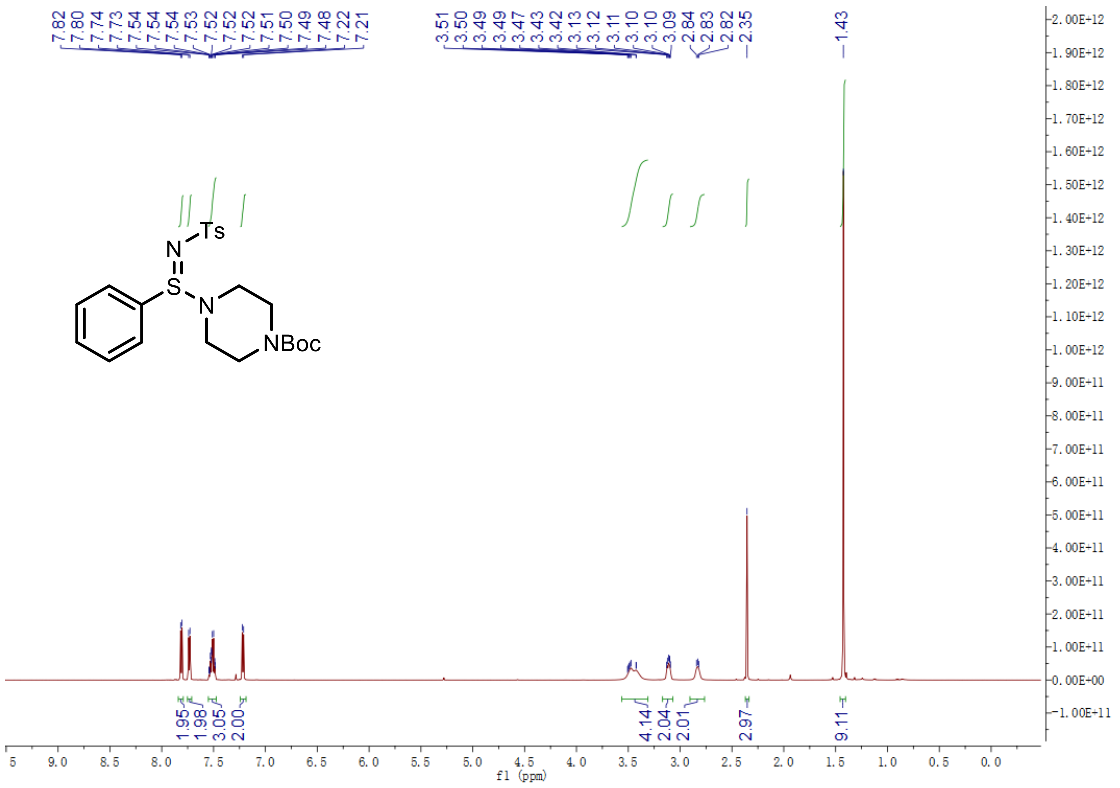

$^{13}$C{$^{1}$H} NMR spectrum of compound **7f** (151 MHz, $CDCl_3$)

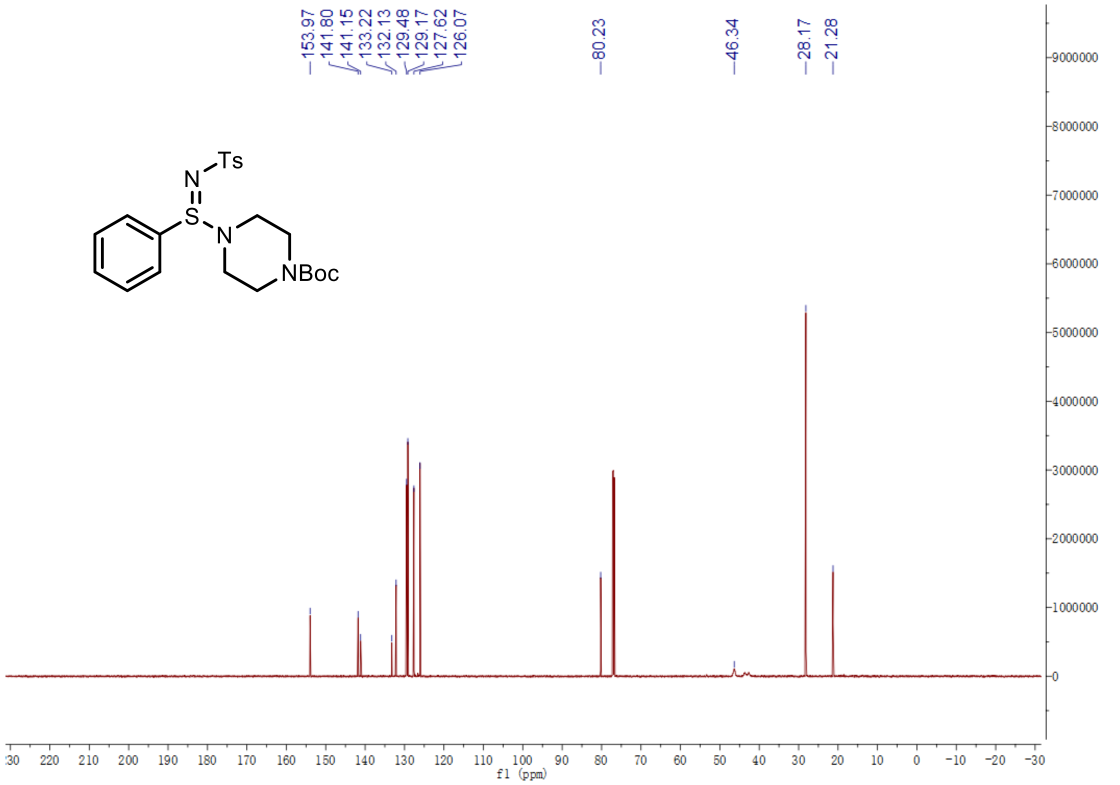

$^{1}H$ NMR spectrum of compound **7g** (600 MHz, $CDCl_3$)

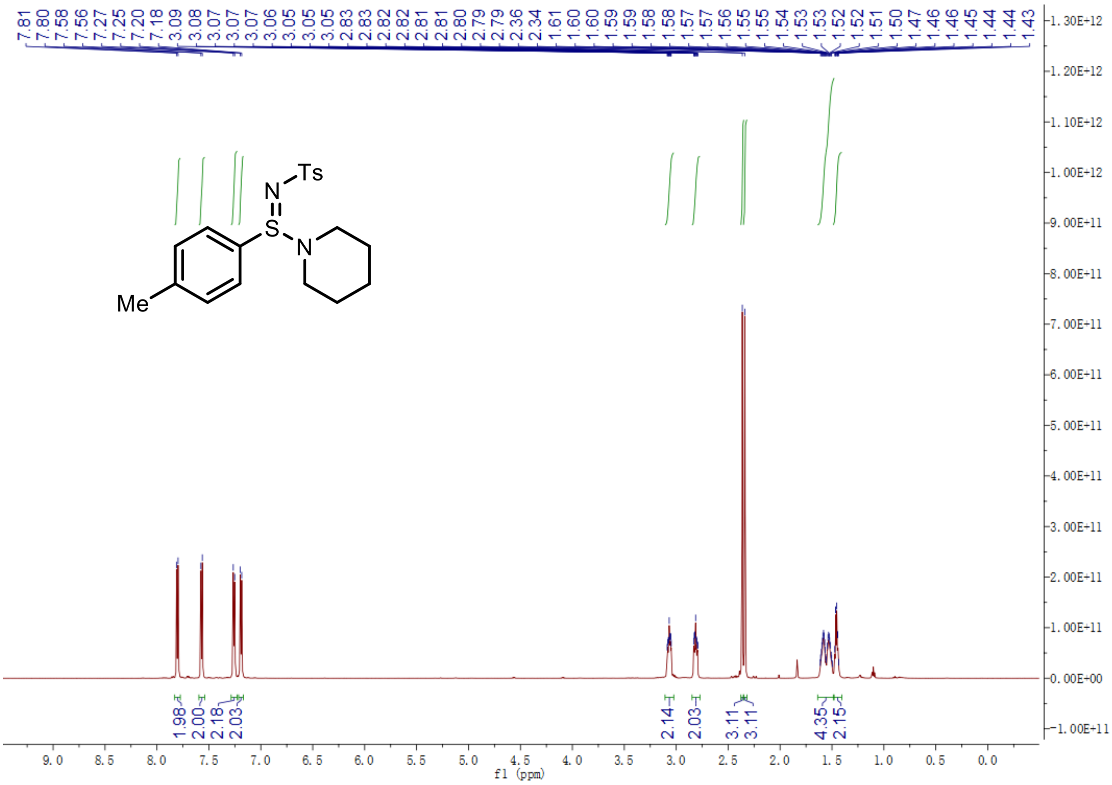


$^{13}C\{^{1}H\}$ NMR spectrum of compound **7g** (151 MHz, $CDCl_3$)

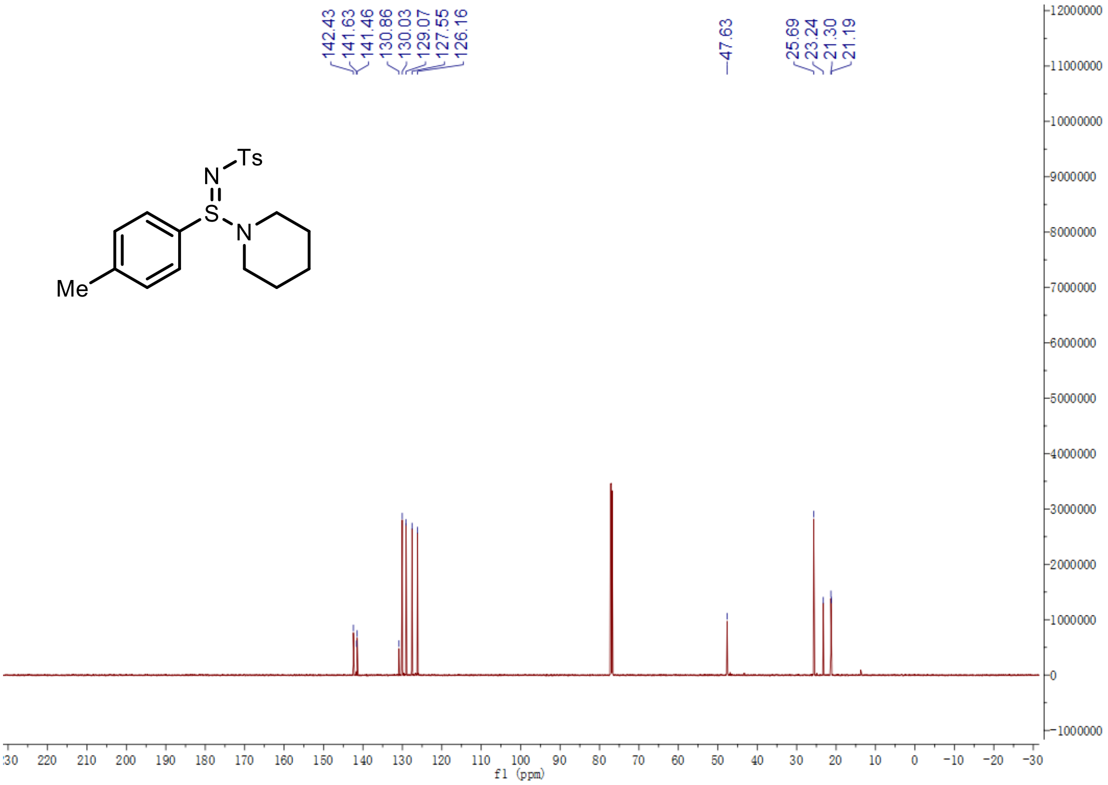

$^{1}$H NMR spectrum of compound **7h** (600 MHz, $CDCl_3$)

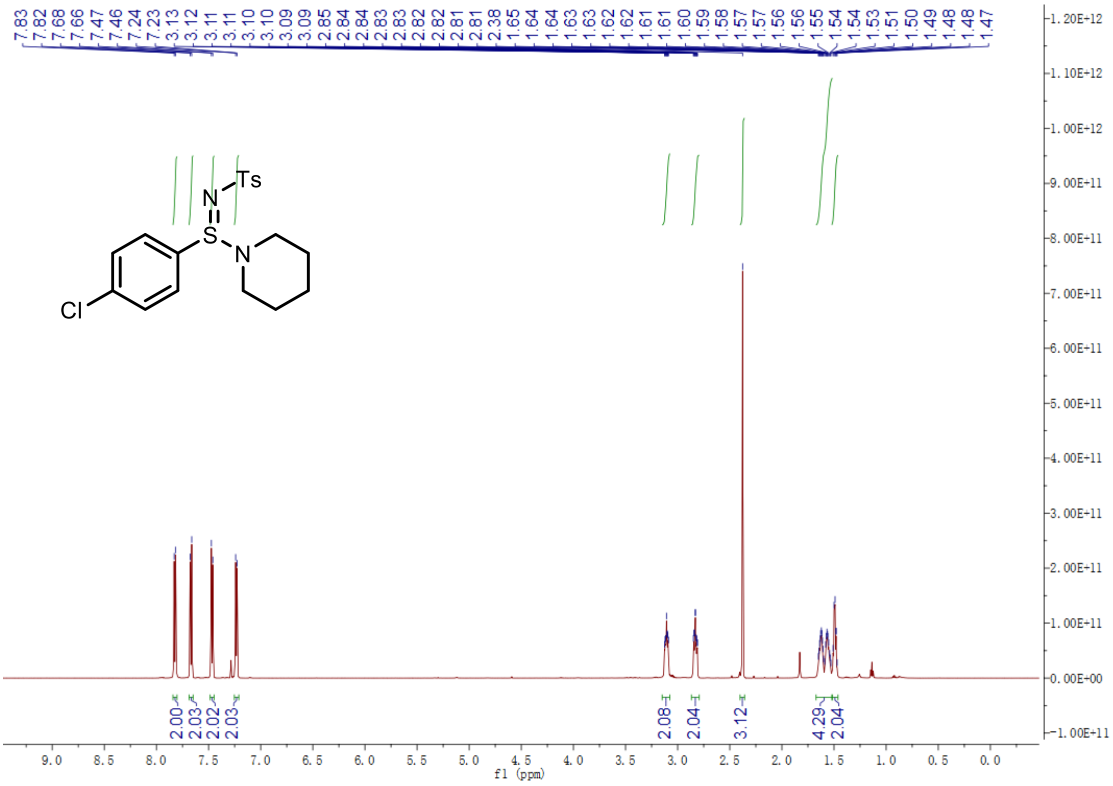


$^{13}$C{$^{1}$H} NMR spectrum of compound **7h** (151 MHz, $CDCl_3$)

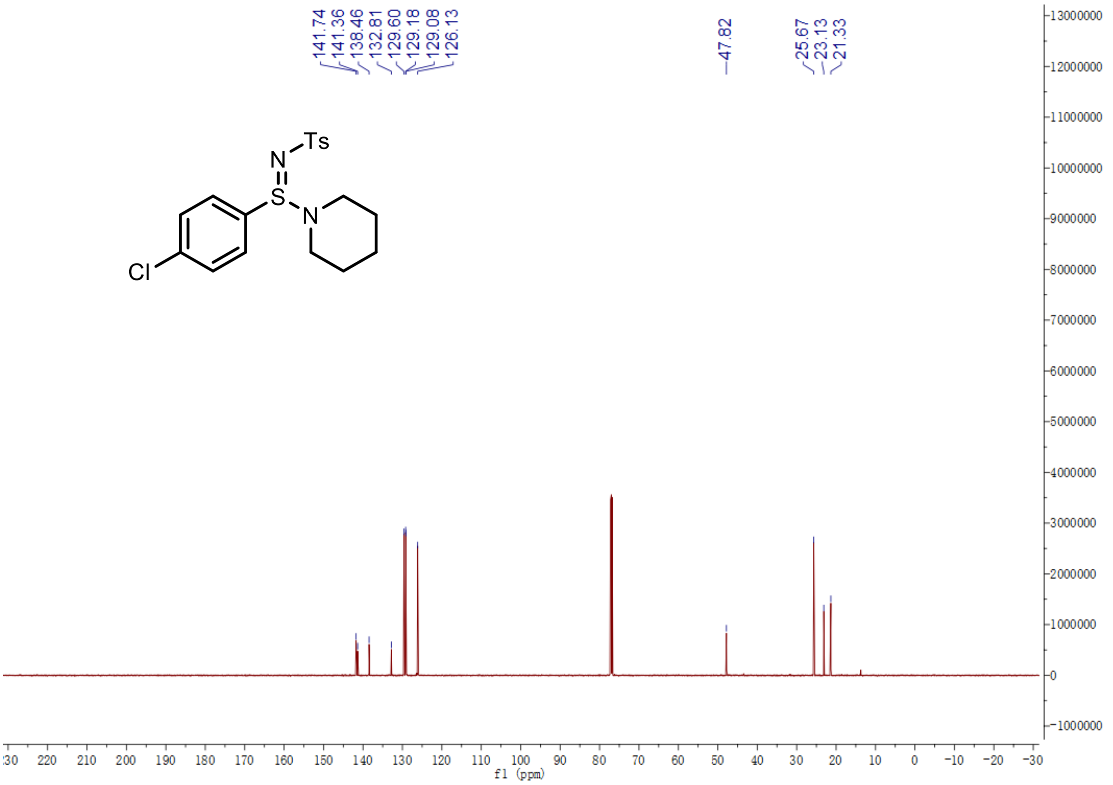

$^{1}$H NMR spectrum of compound **7i** (600 MHz, $CDCl_3$)

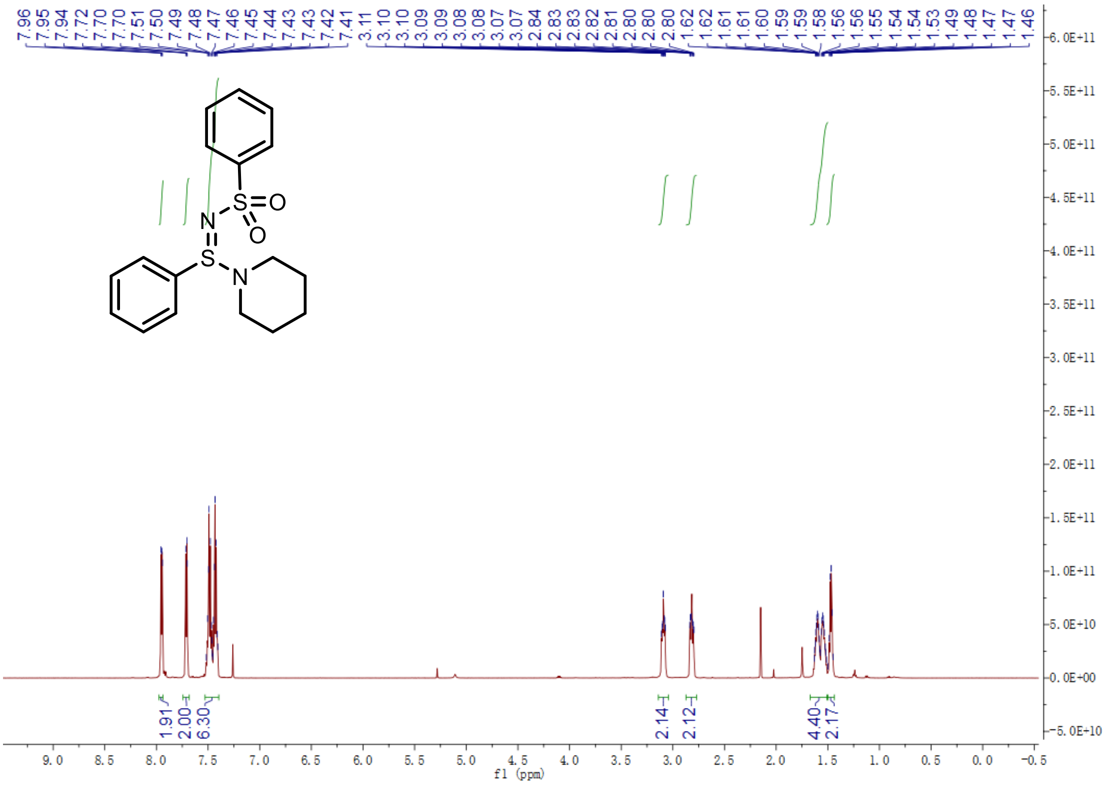

$^{13}$C{$^{1}$H} NMR spectrum of compound **7i** (151 MHz, $CDCl_3$)

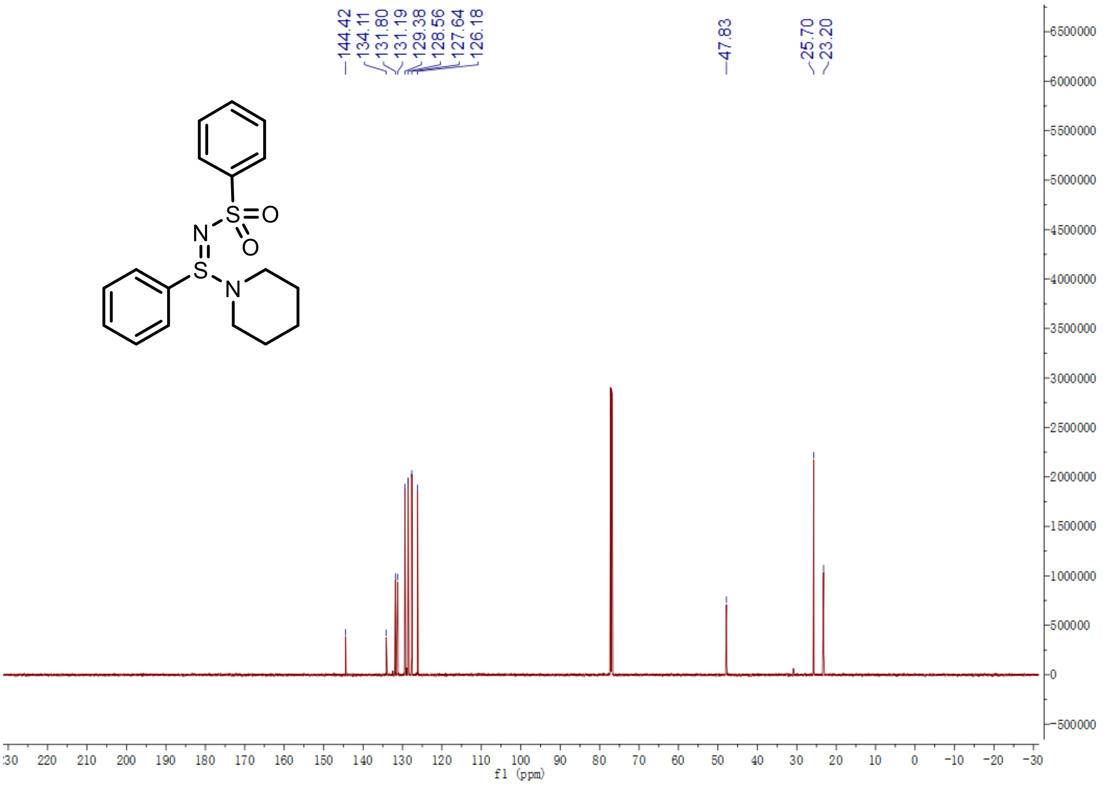

$^{1}H$ NMR spectrum of compound **7j** (600 MHz, $CDCl_3$)

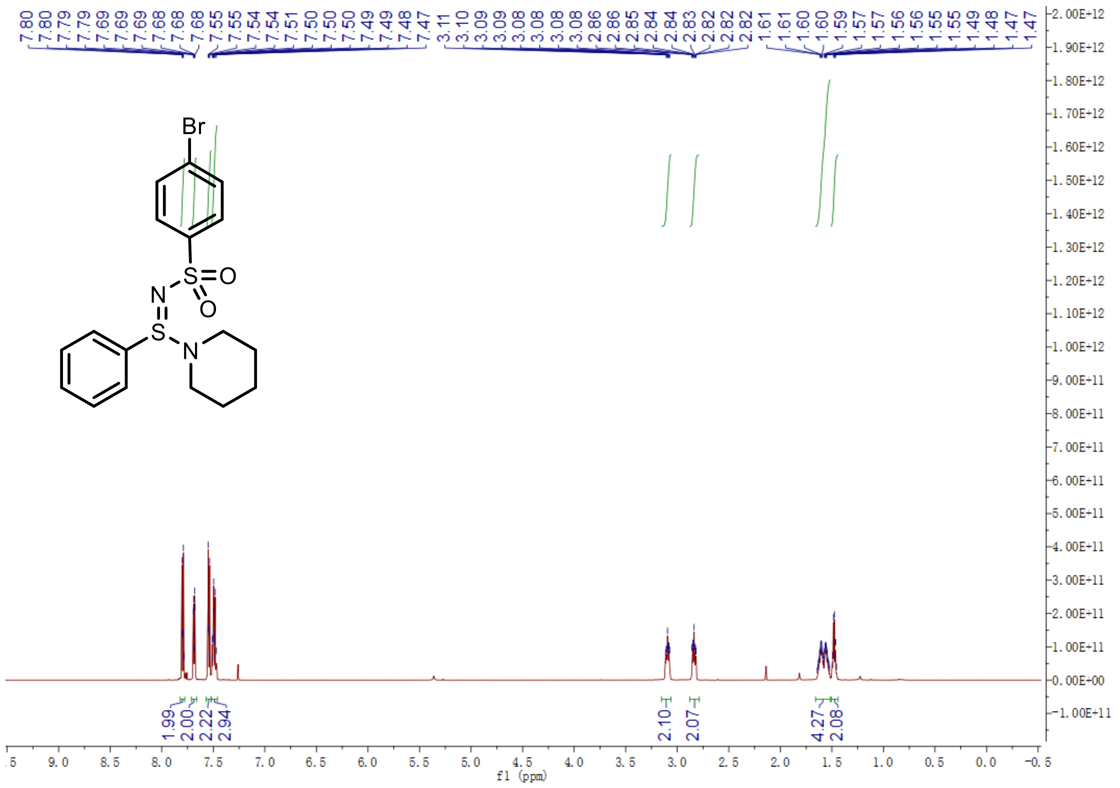


$^{13}C\{^{1}H\}$ NMR spectrum of compound **7j** (151 MHz, $CDCl_3$)

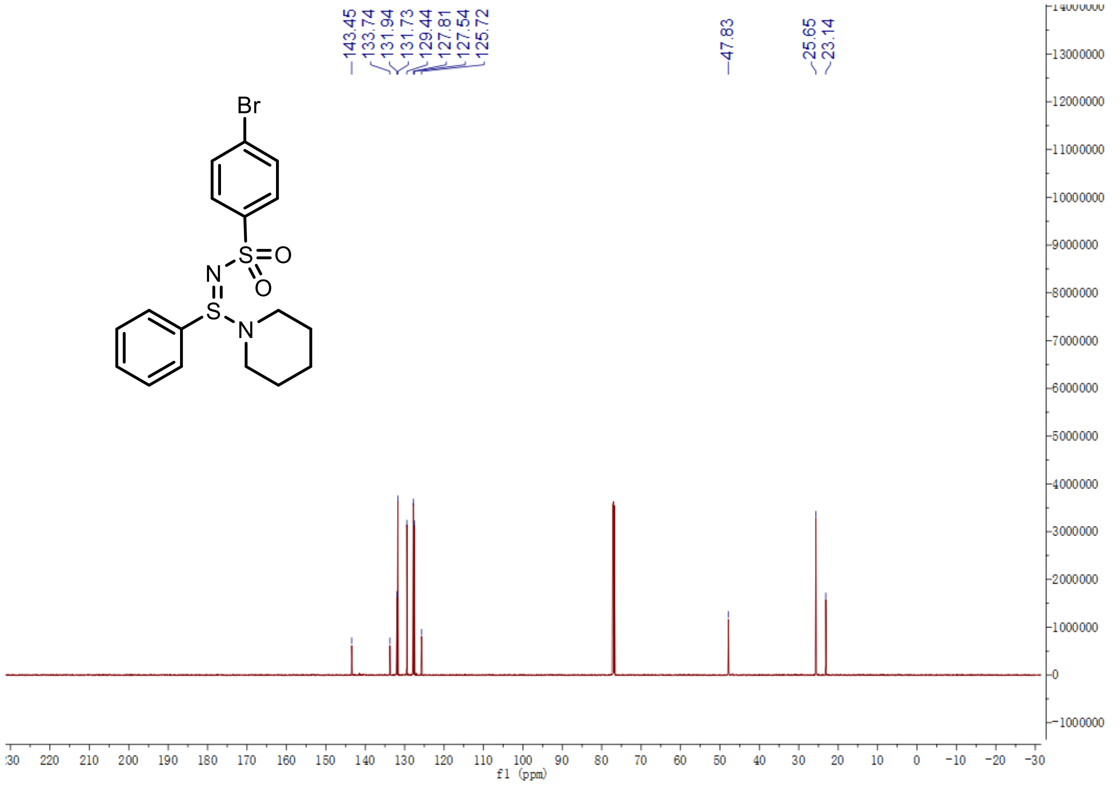

$^{1}$H NMR spectrum of compound **7k** (600 MHz, $CDCl_3$)

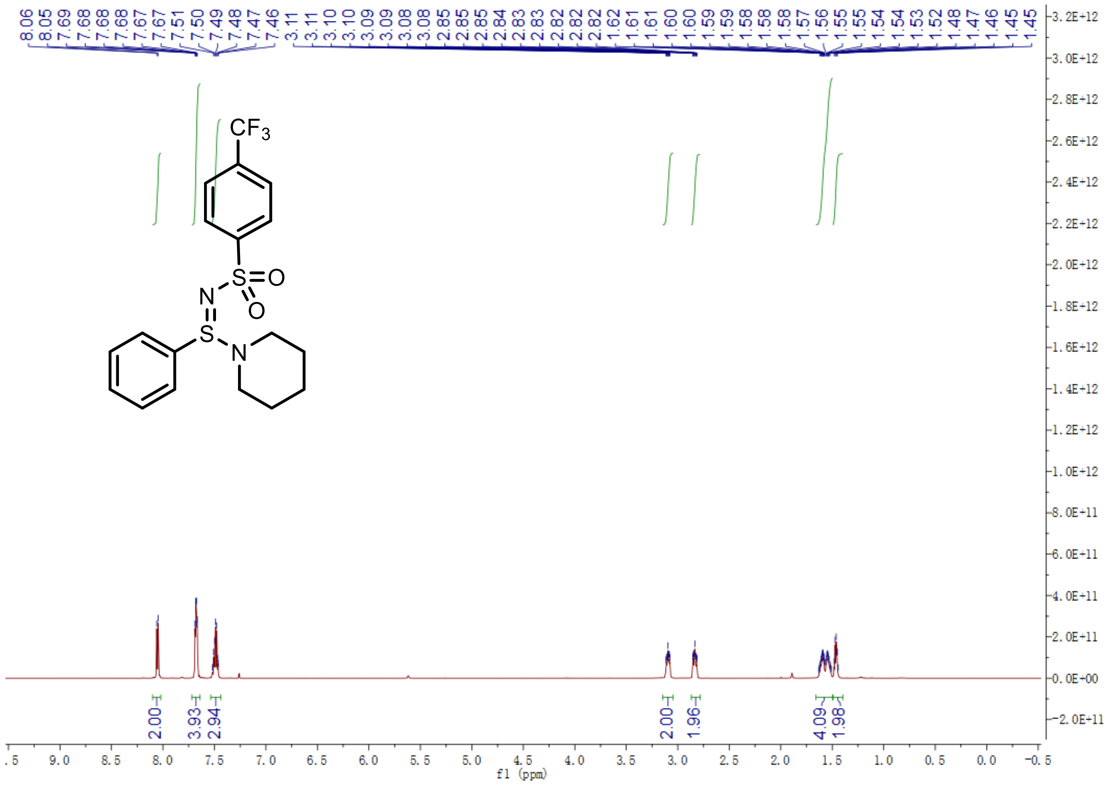

$^{13}$C{$^{1}$H} NMR spectrum of compound **7k** (151 MHz, $CDCl_3$)

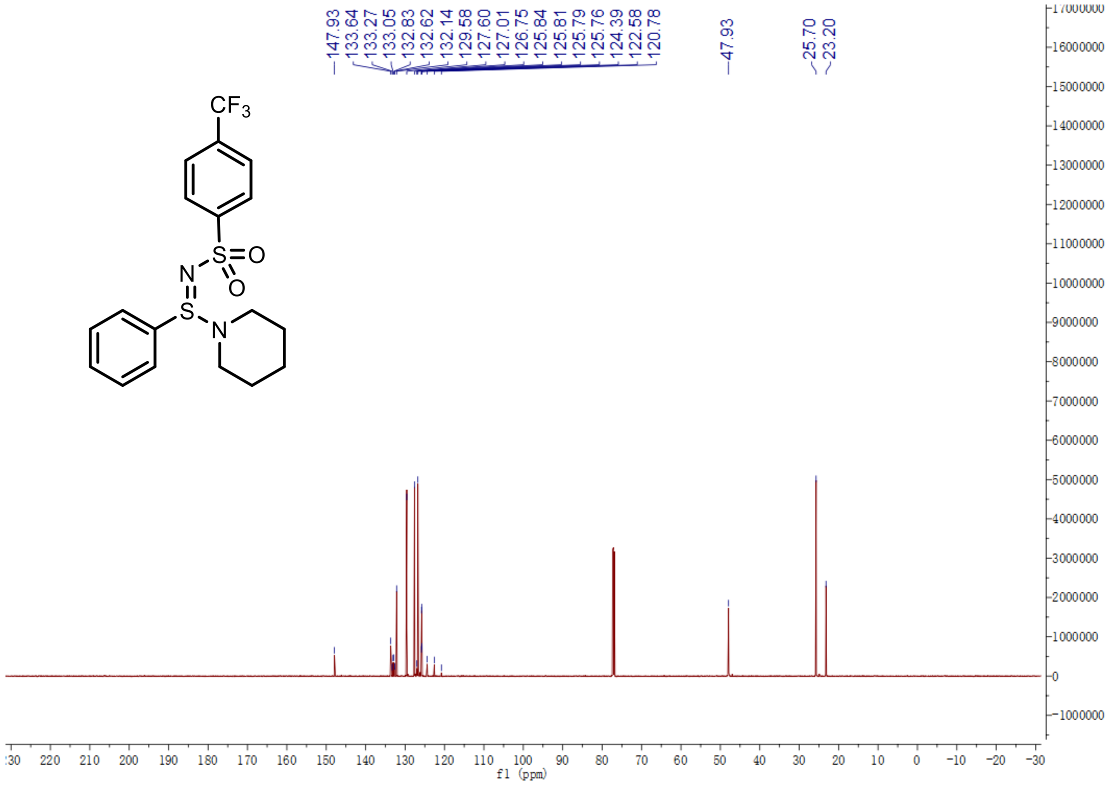

$^{19}F$ NMR spectrum of compound **7k** (564 MHz, $CDCl_3$)

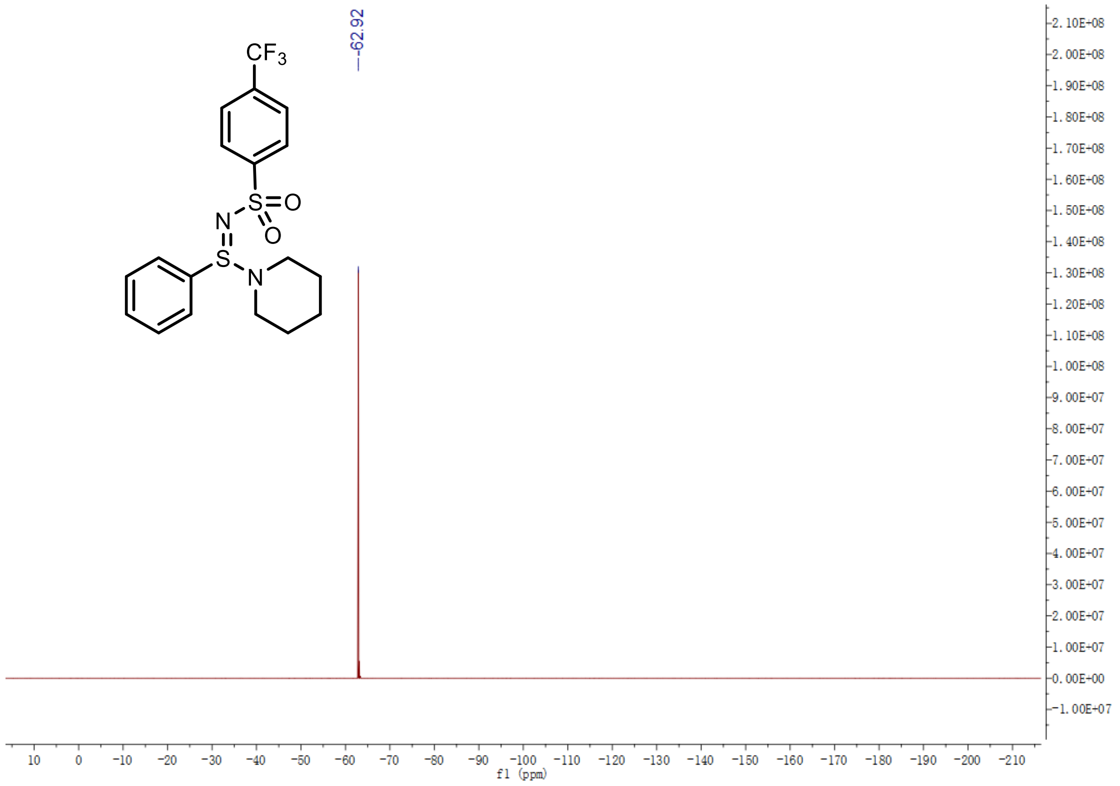


$^{1}H$ NMR spectrum of compound **7l** (600 MHz, $CDCl_3$)

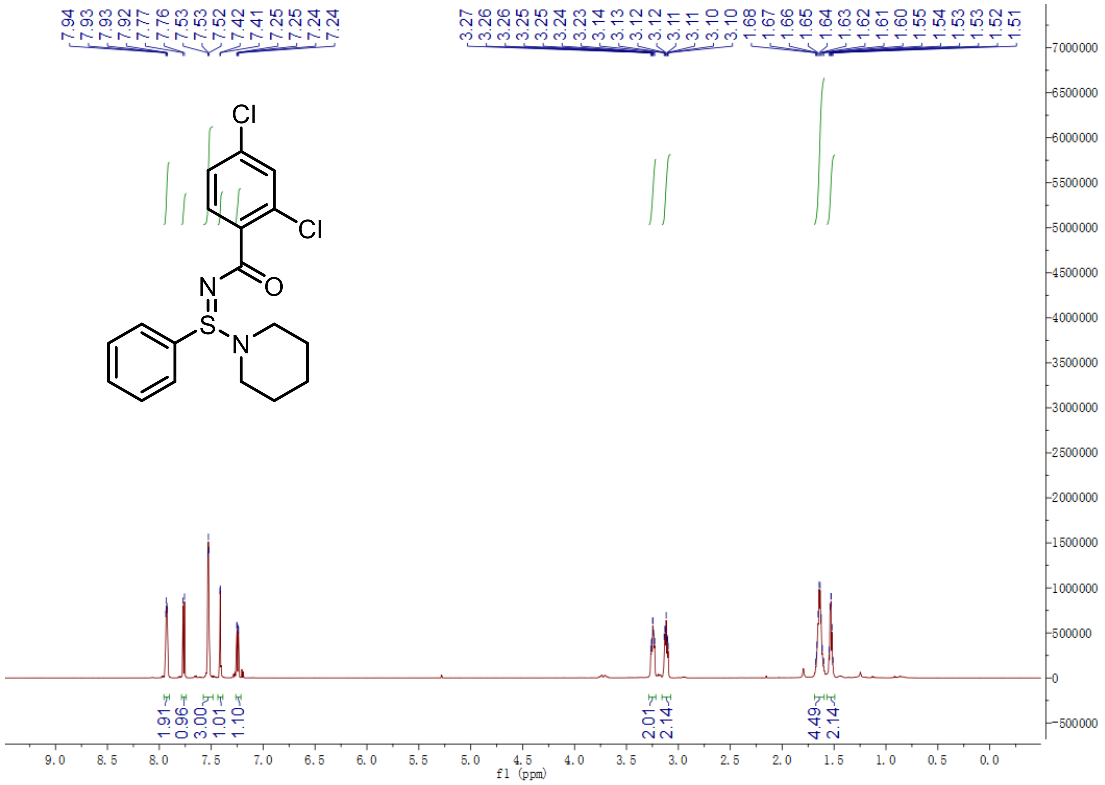

$^{13}C\{^1H\}$ NMR spectrum of compound **7l** (151 MHz, $CDCl_3$)

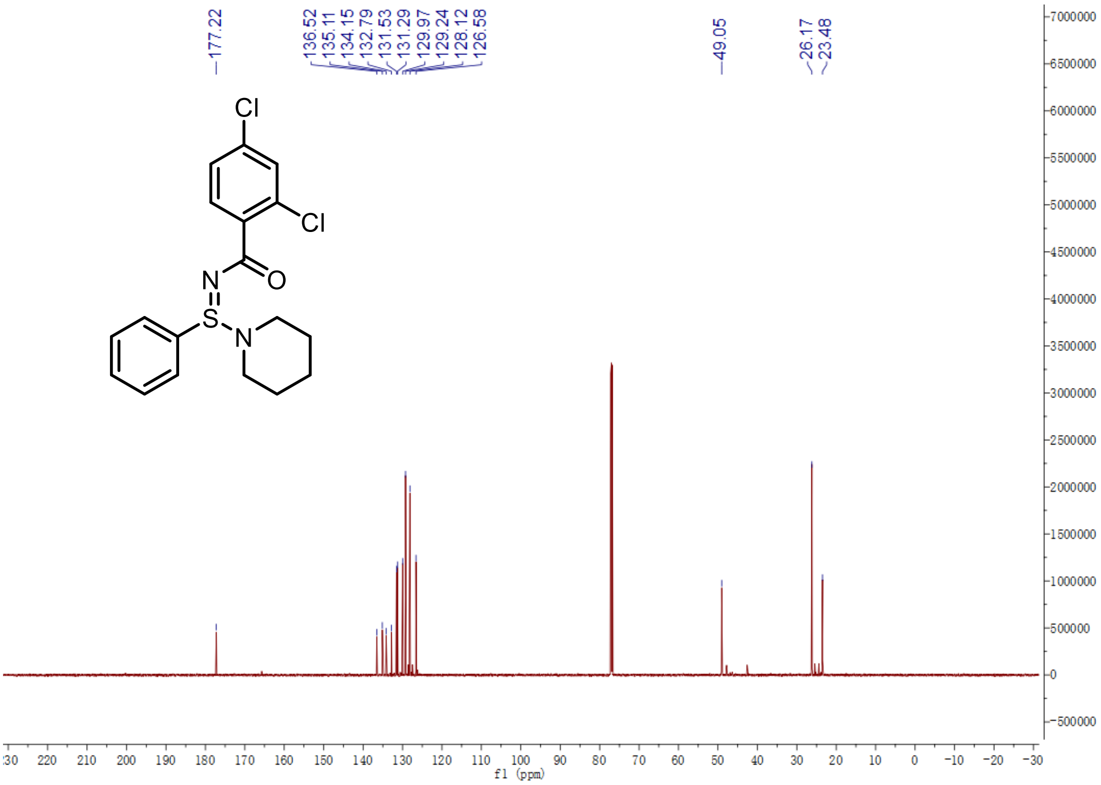


$^1H$ NMR spectrum of compound **8** (400 MHz, $CDCl_3$)

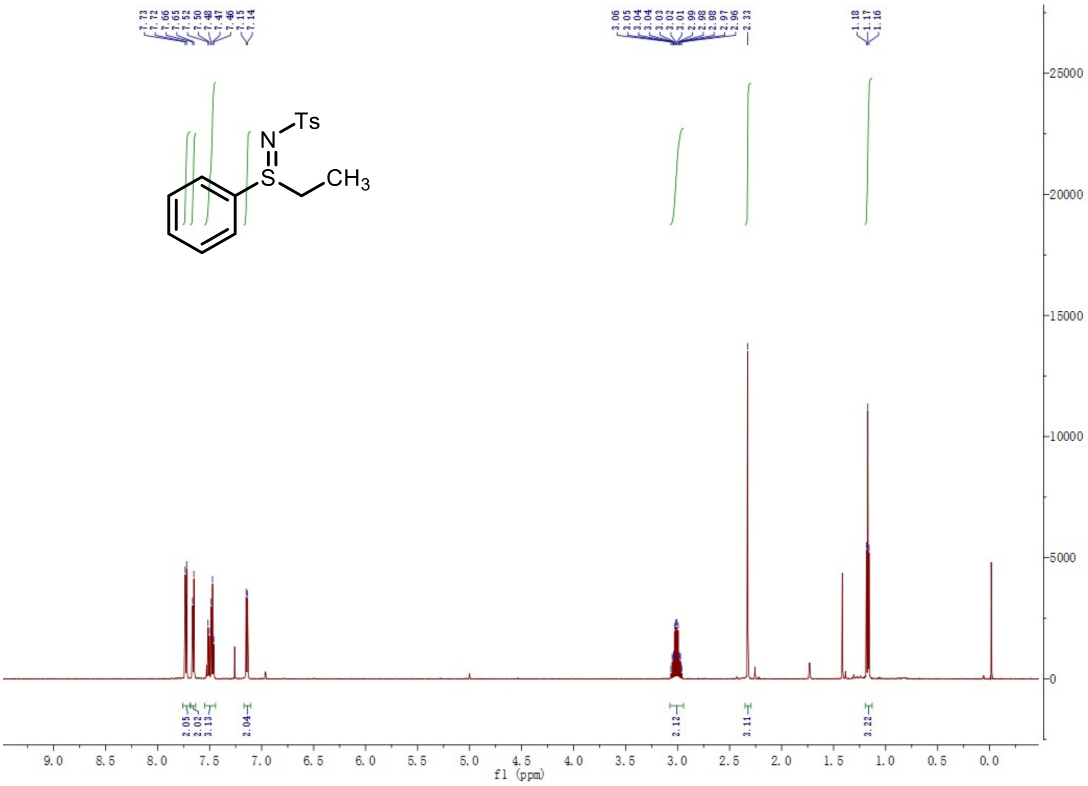

$^{13}C\{^1H\}$ NMR spectrum of compound **8** (101 MHz, $CDCl_3$)

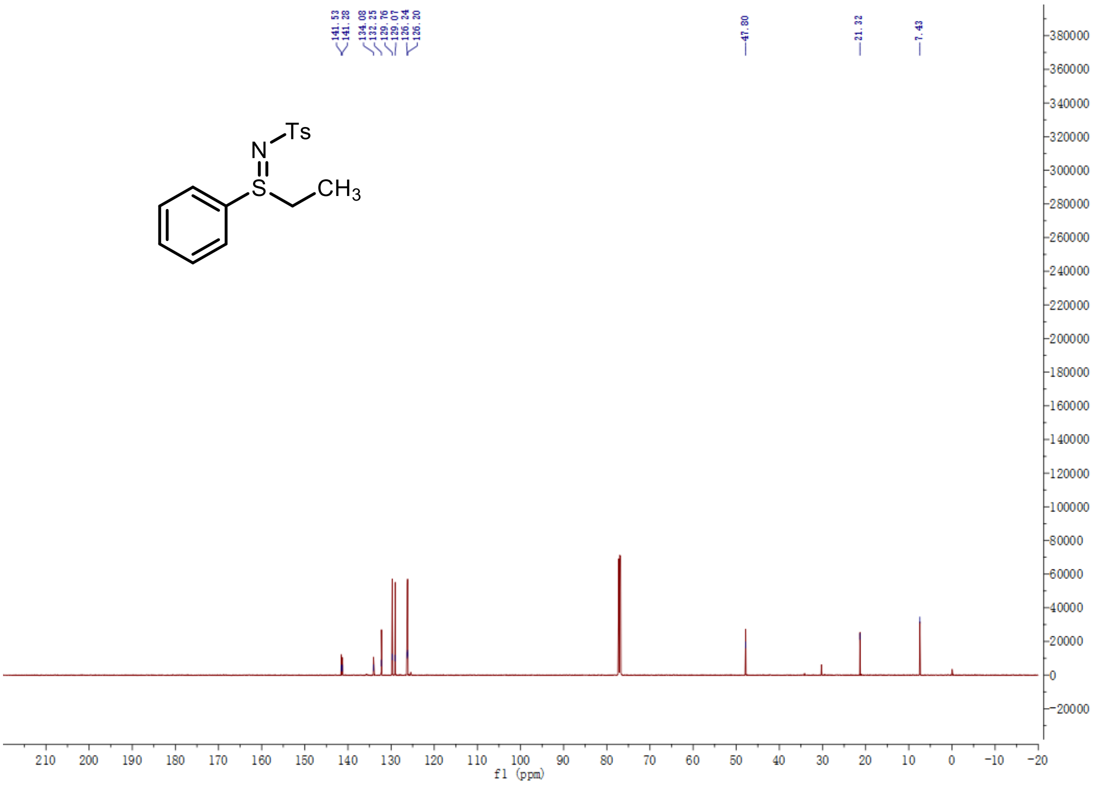

$^1H$ NMR spectrum of compound **9** (400 MHz, $CDCl_3$)

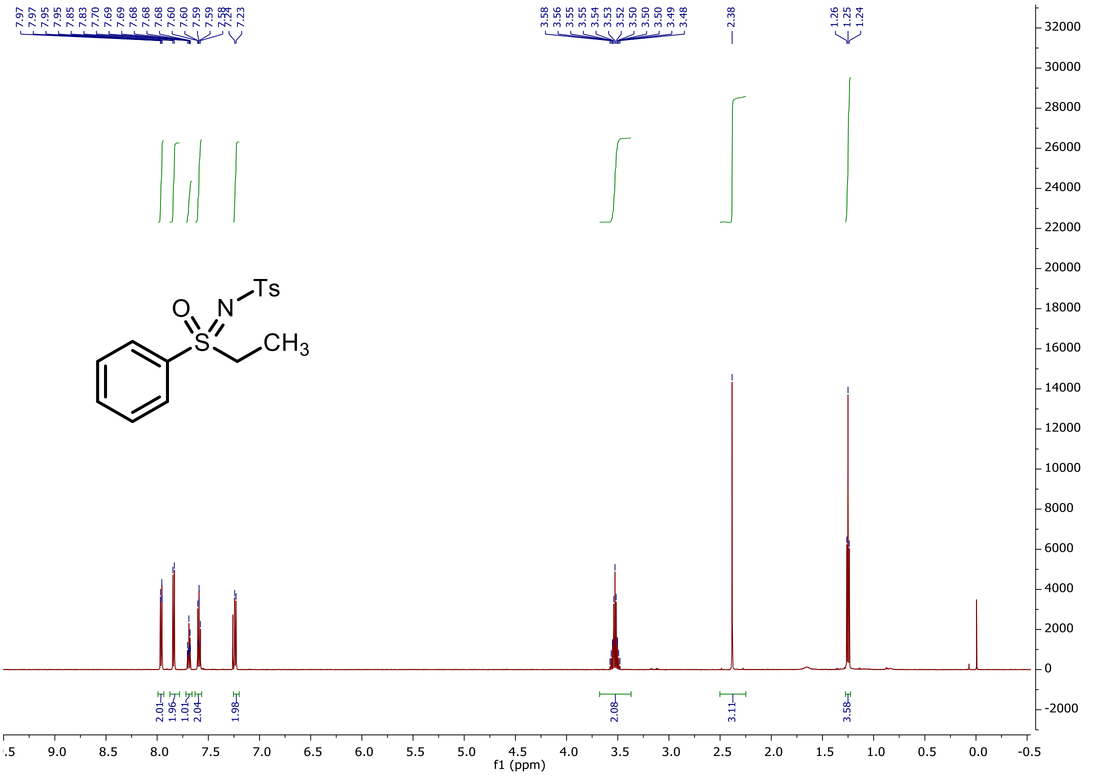

$^{13}C\{^{1}H\}$ NMR spectrum of compound **9** (101 MHz, $CDCl_3$)

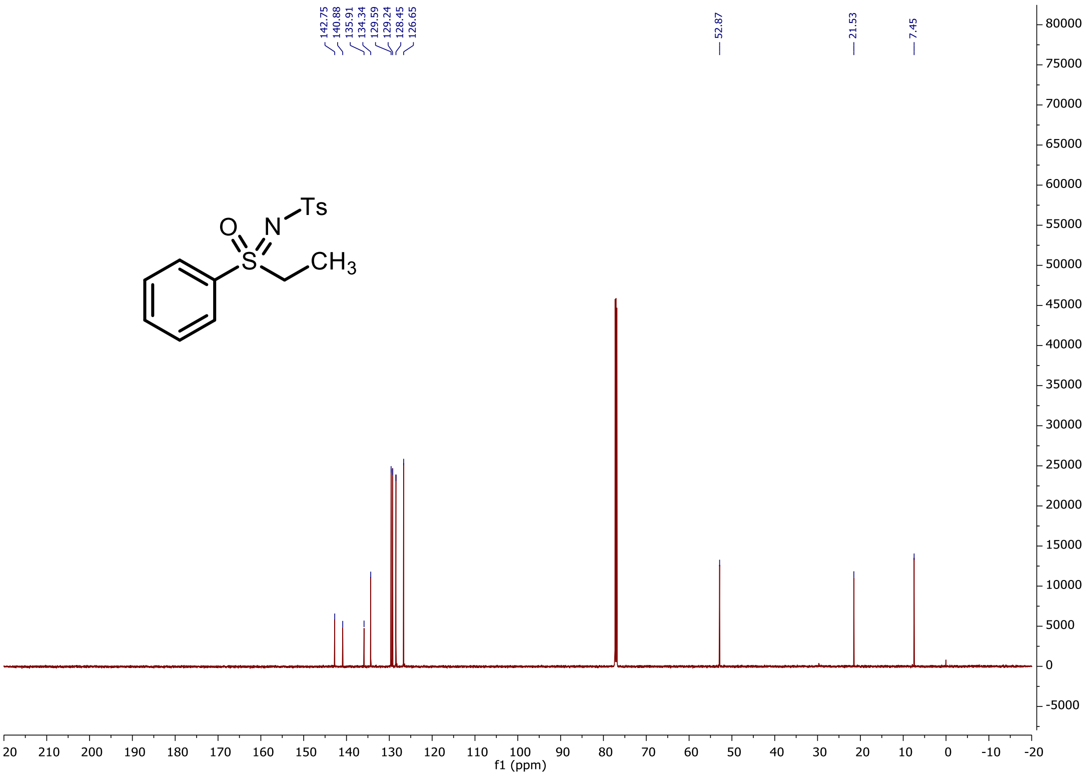


$^{1}H$ NMR spectrum of compound **10** (400 MHz, $CDCl_3$)

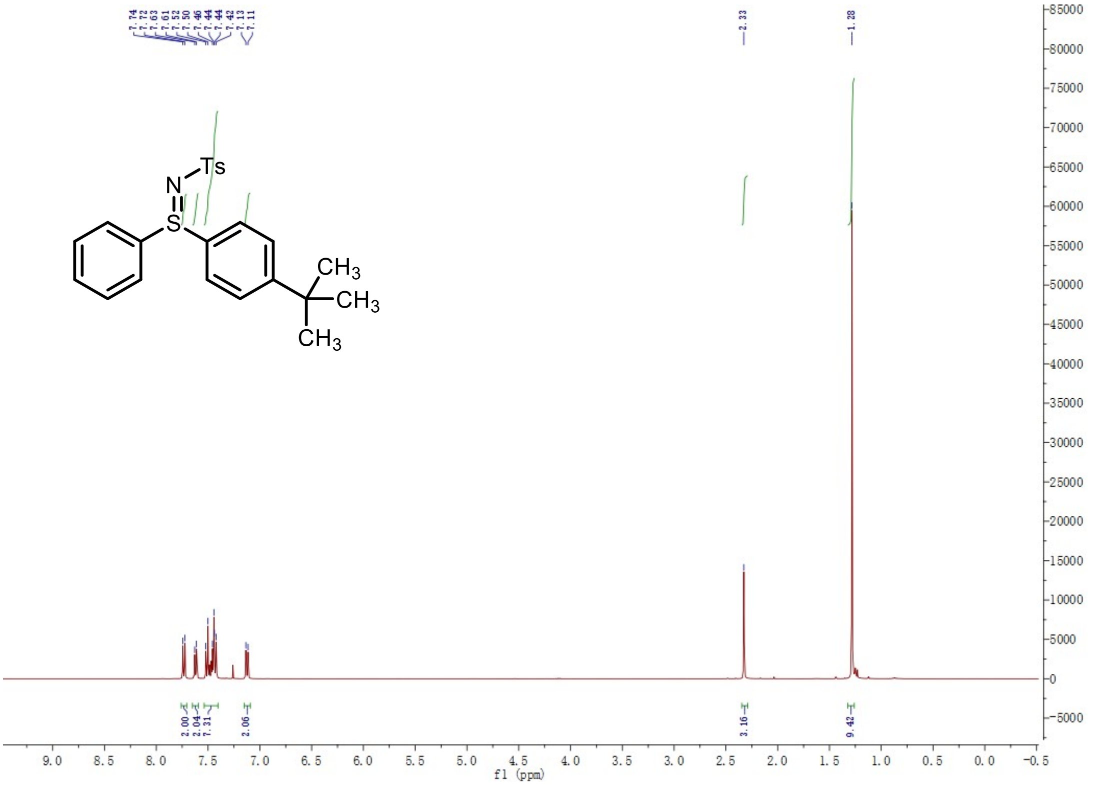

$^{13}C\{^{1}H\}$ NMR spectrum of compound **10** (101 MHz, $CDCl_3$)

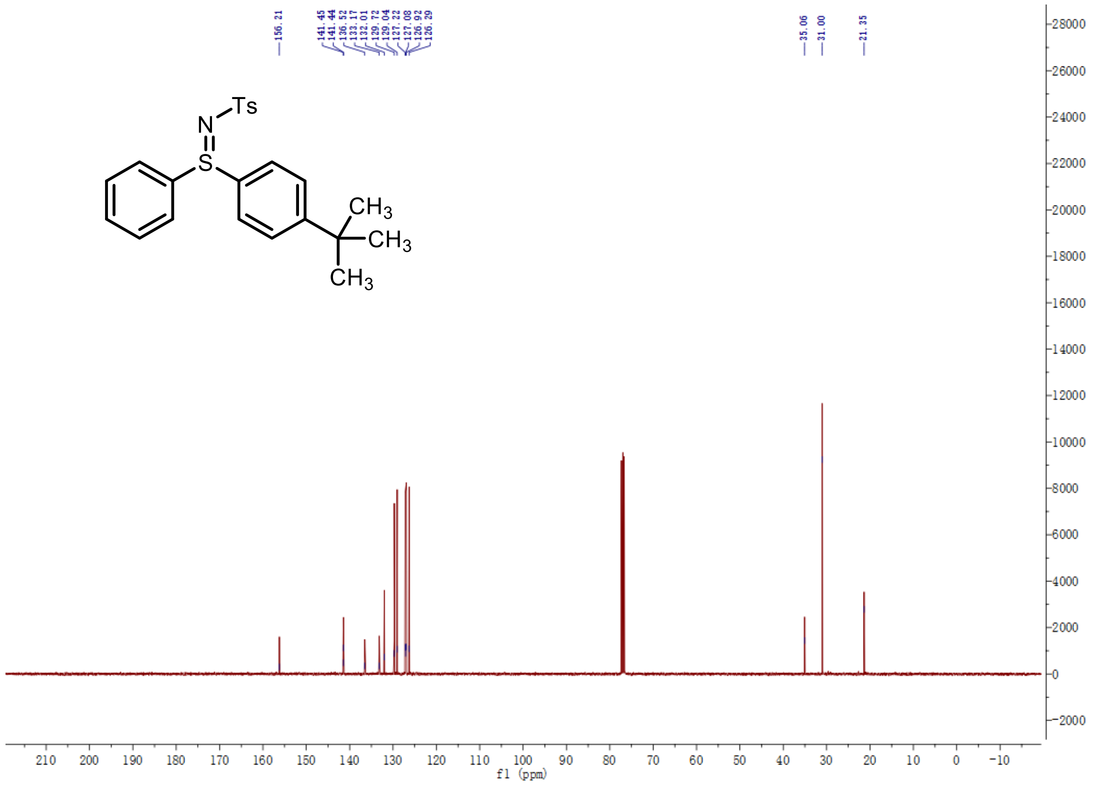

$^{1}H$ NMR spectrum of compound **11** (600 MHz, $CDCl_3$)

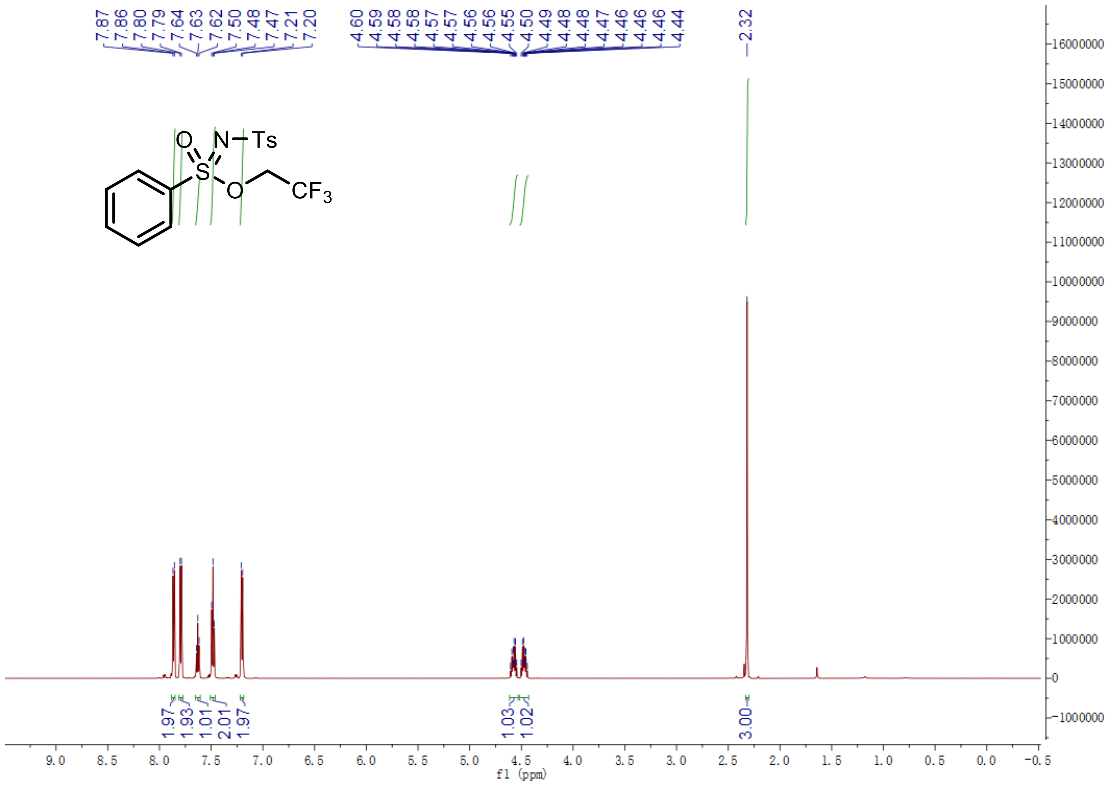

$^{13}C\{^{1}H\}$ NMR spectrum of compound **11** (151 MHz, $CDCl_3$)

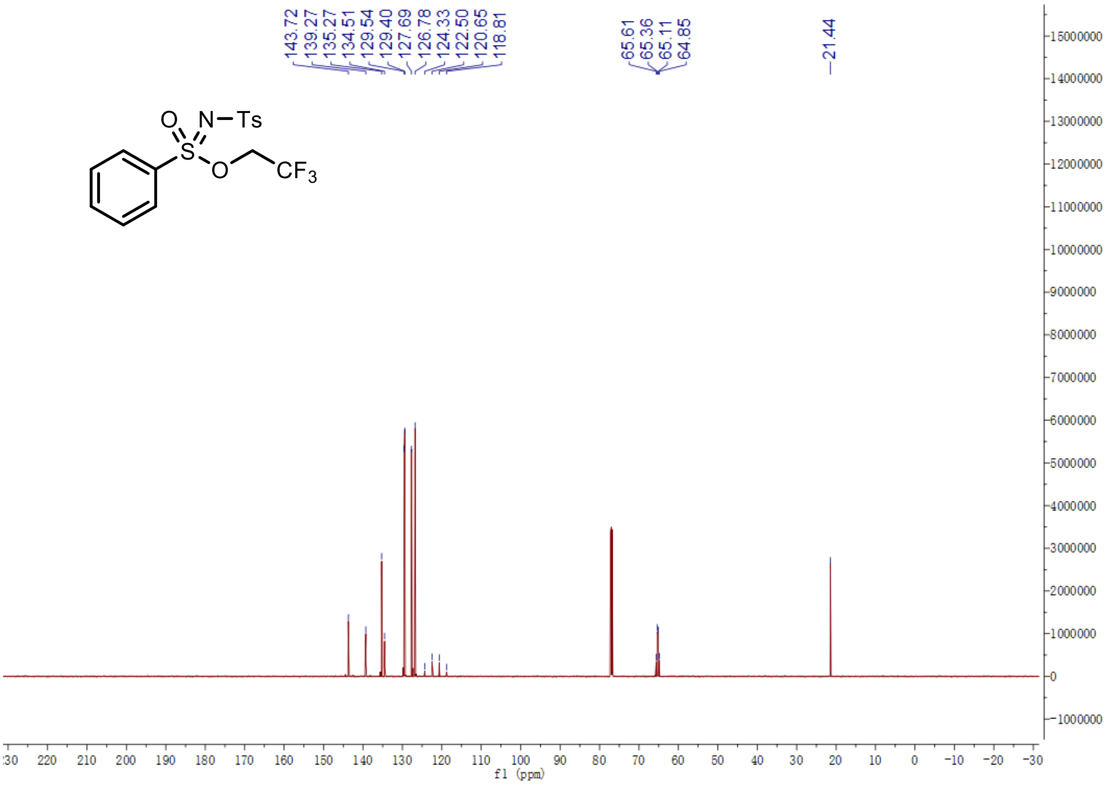


$^{19}F$ NMR spectrum of compound **11** (564 MHz, $CDCl_3$)

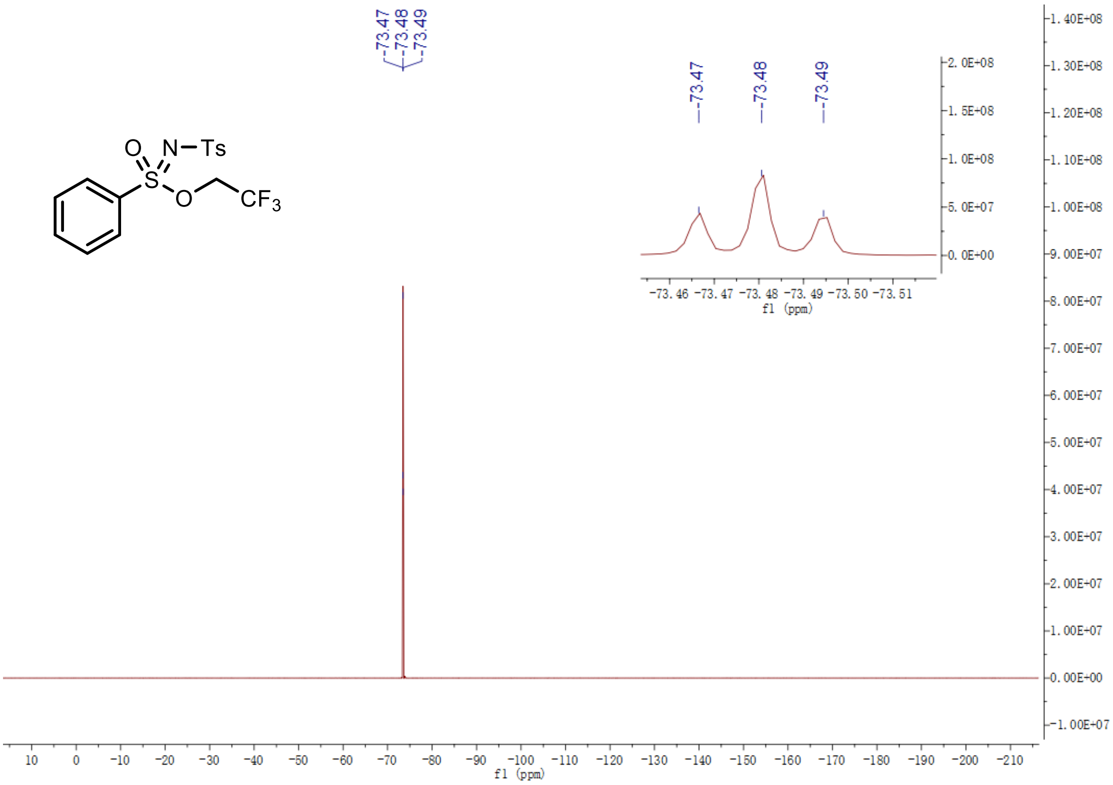

$^{1}$H NMR spectrum of compound **12** (600 MHz, $CDCl_3$)

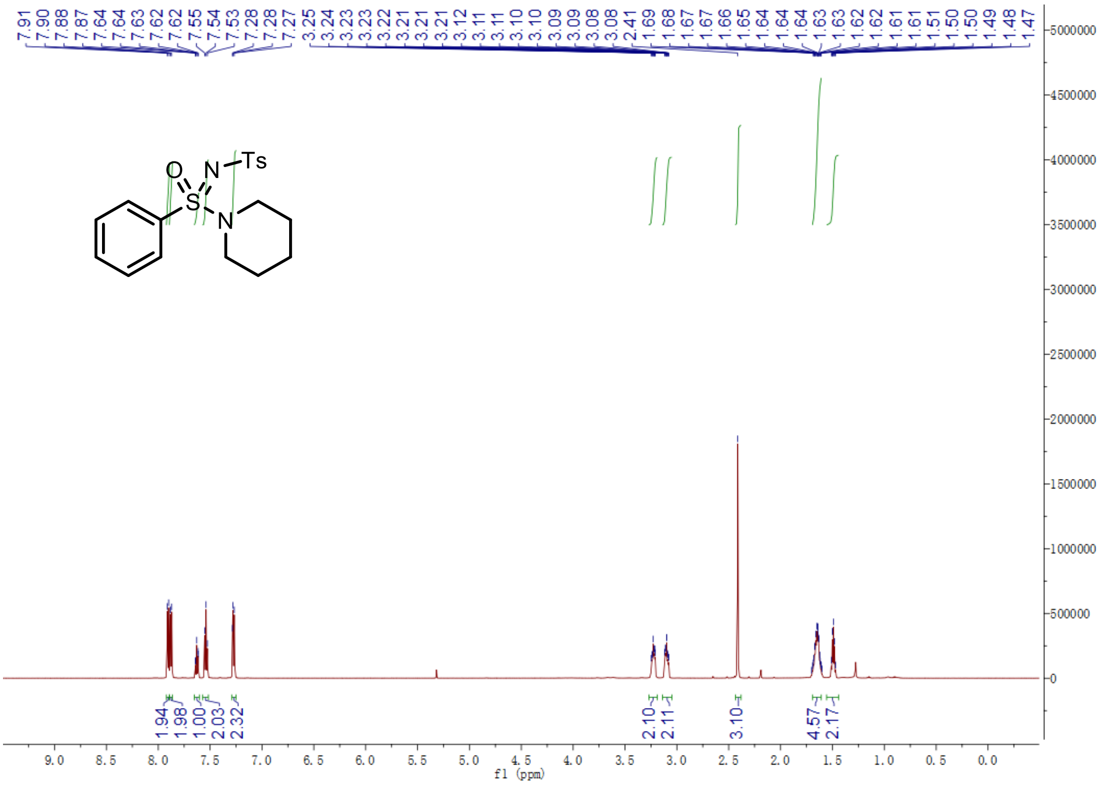


$^{13}$C{$^{1}$H} NMR spectrum of compound **12** (151 MHz, $CDCl_3$)

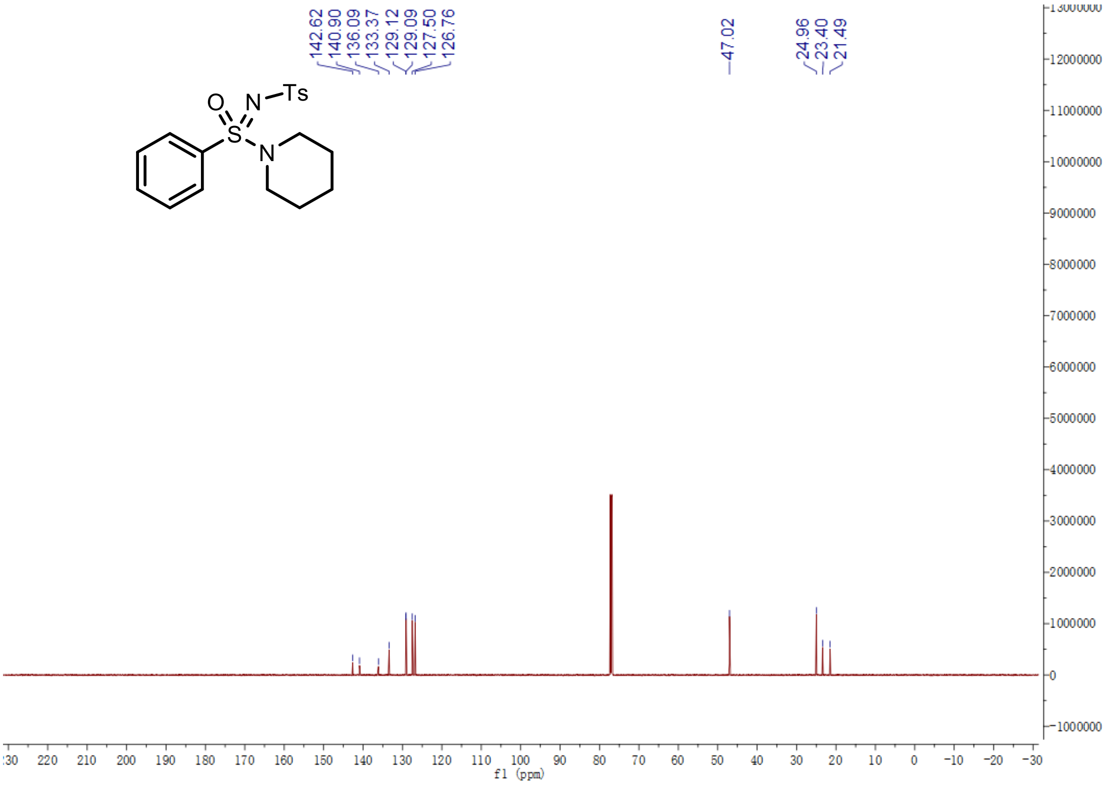

$^{1}$H NMR spectrum of compound **13** (400 MHz, DMSO-$d_6$)

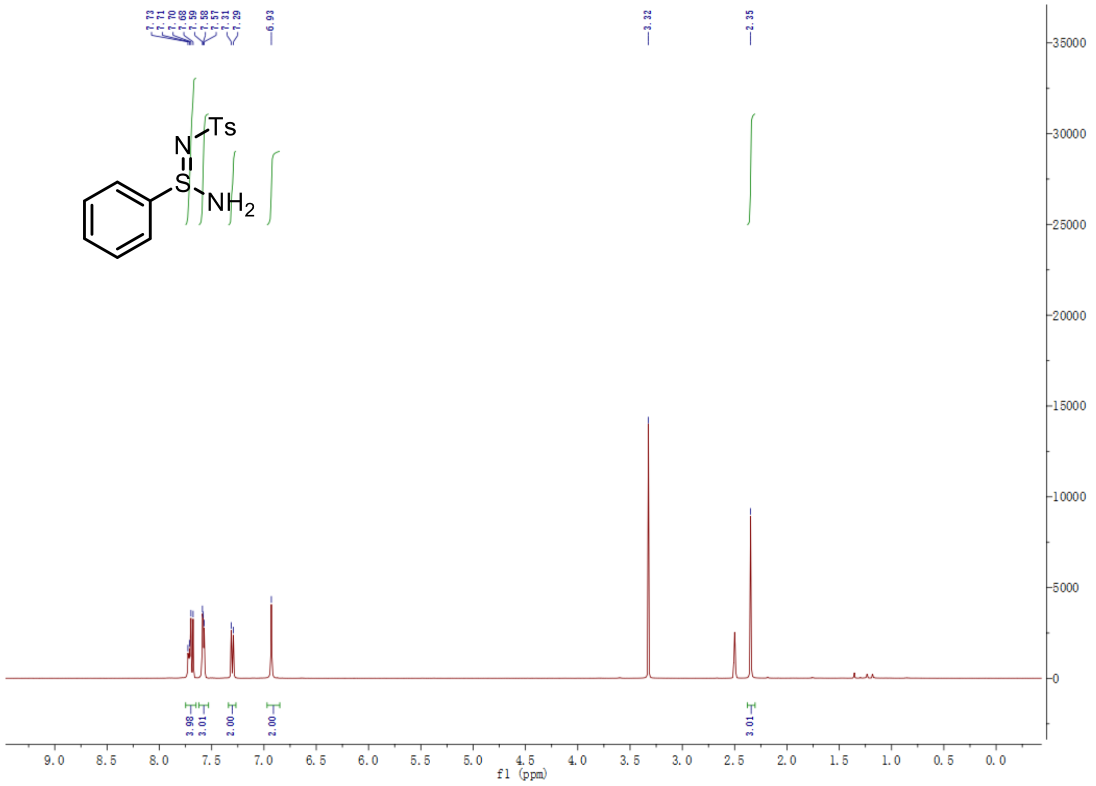

$^{13}$C{$^{1}$H} NMR spectrum of compound **13** (101 MHz, DMSO-$d_6$)

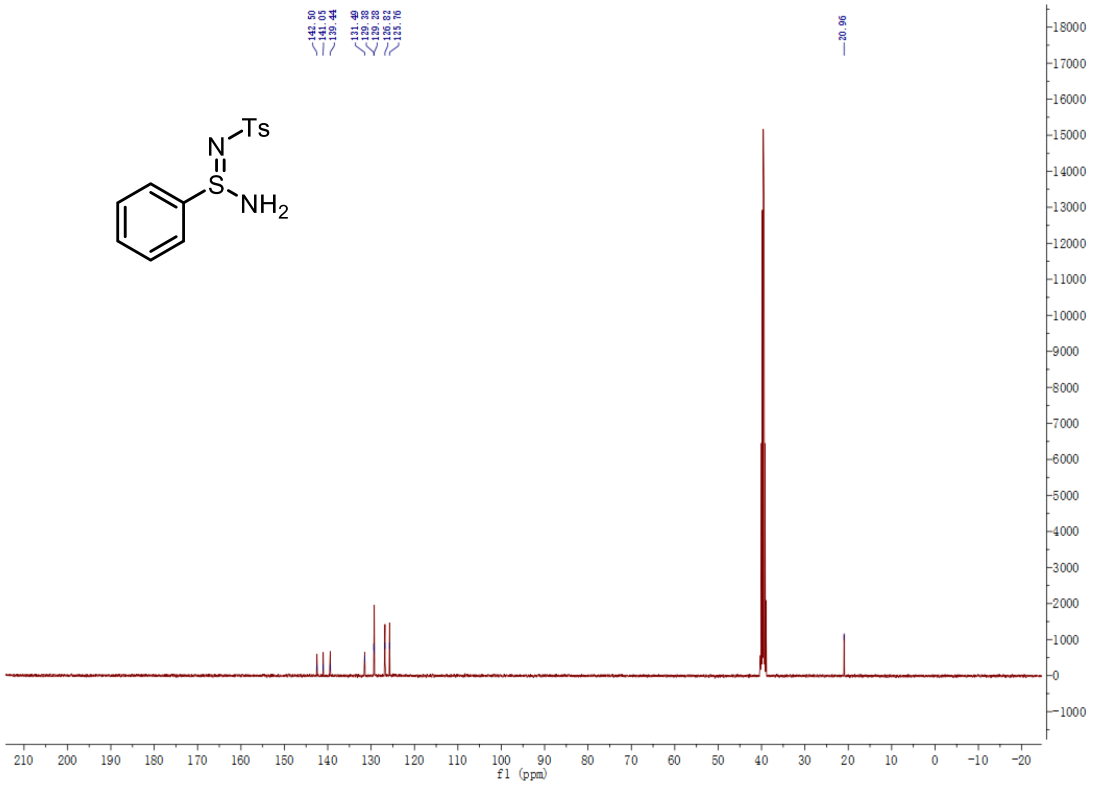

$^{1}$H NMR spectrum of compound **14** (400 MHz, $CDCl_3$)

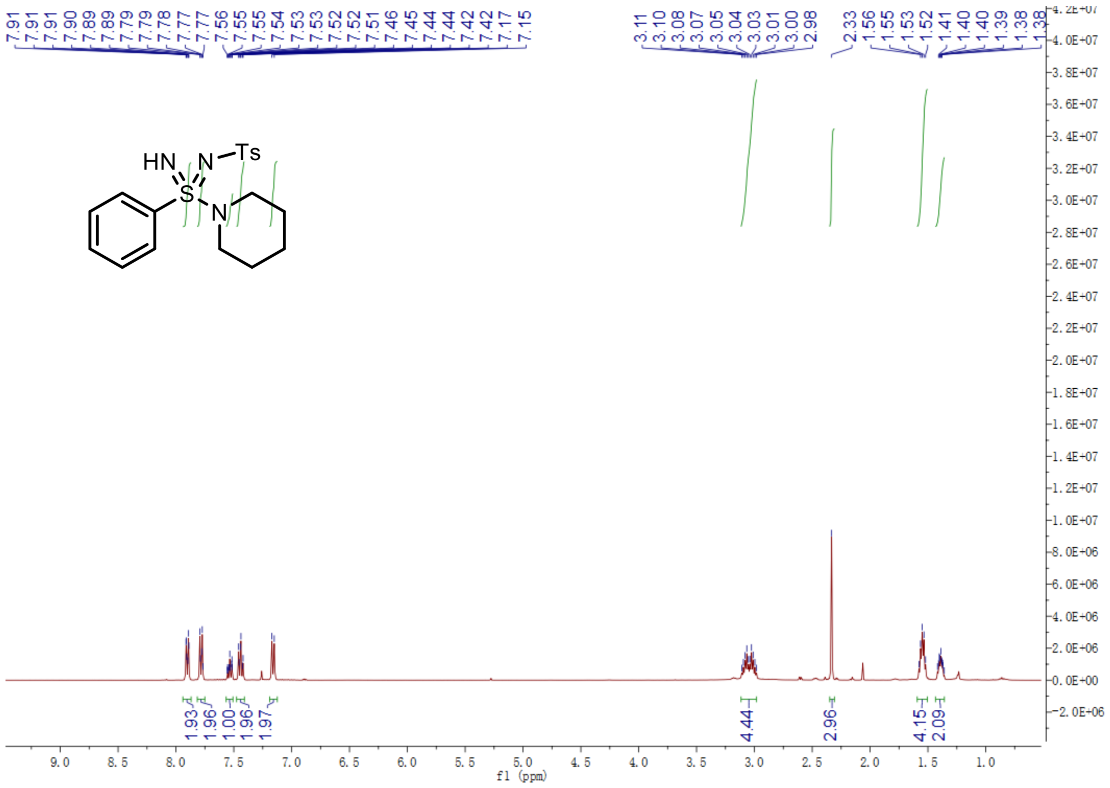

$^{13}C\{^{1}H\}$ NMR spectrum of compound **14** (101 MHz, $CDCl_3$)

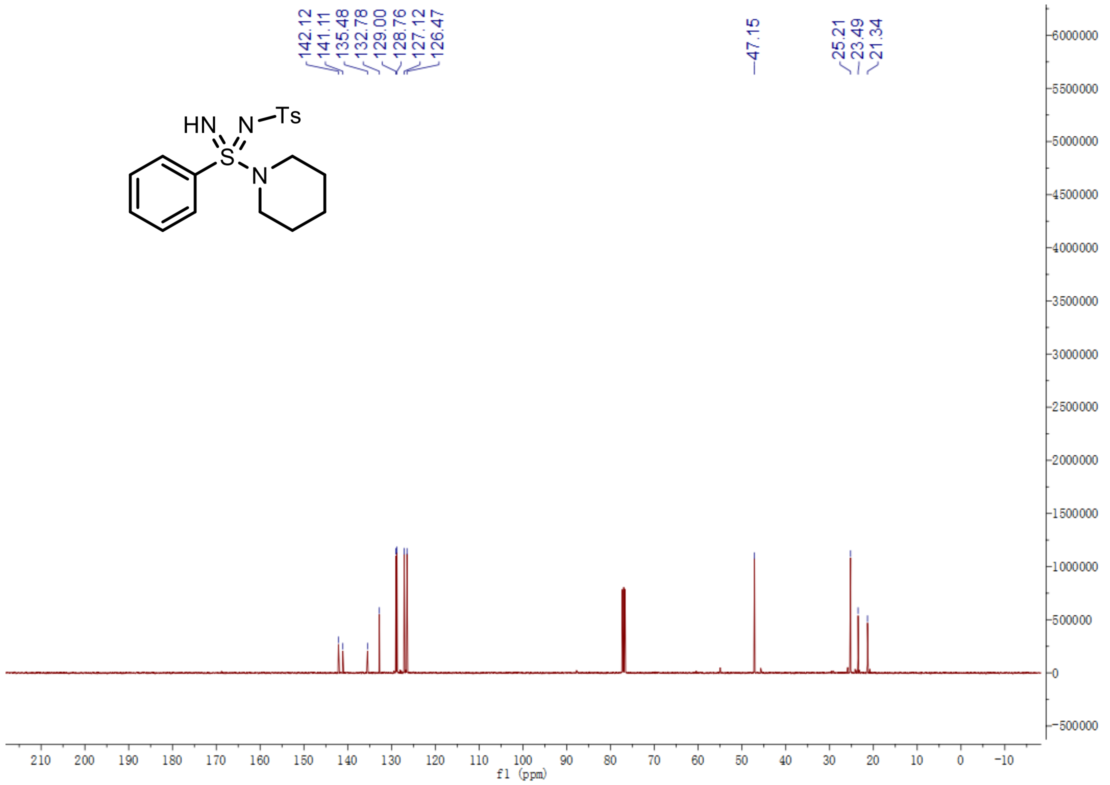

$^1$H NMR spectrum of compound **15** (600 MHz, $CDCl_3$)

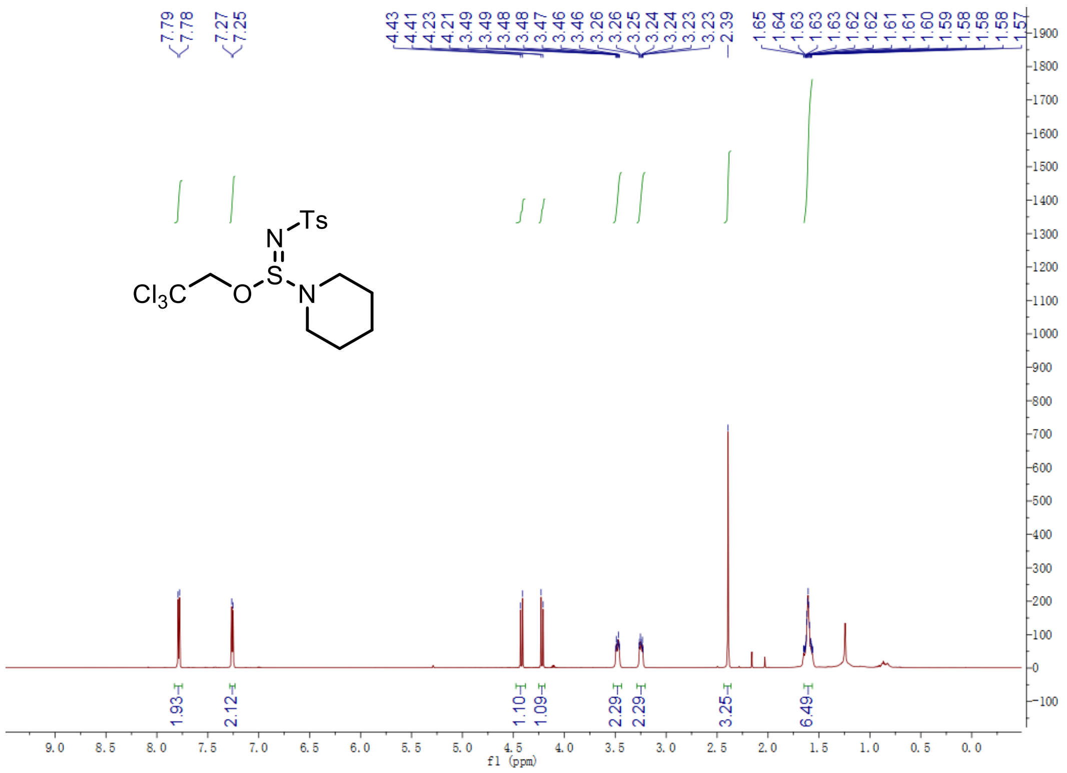

$^{13}C\{^1H\}$ NMR spectrum of compound **15** (151 MHz, $CDCl_3$)

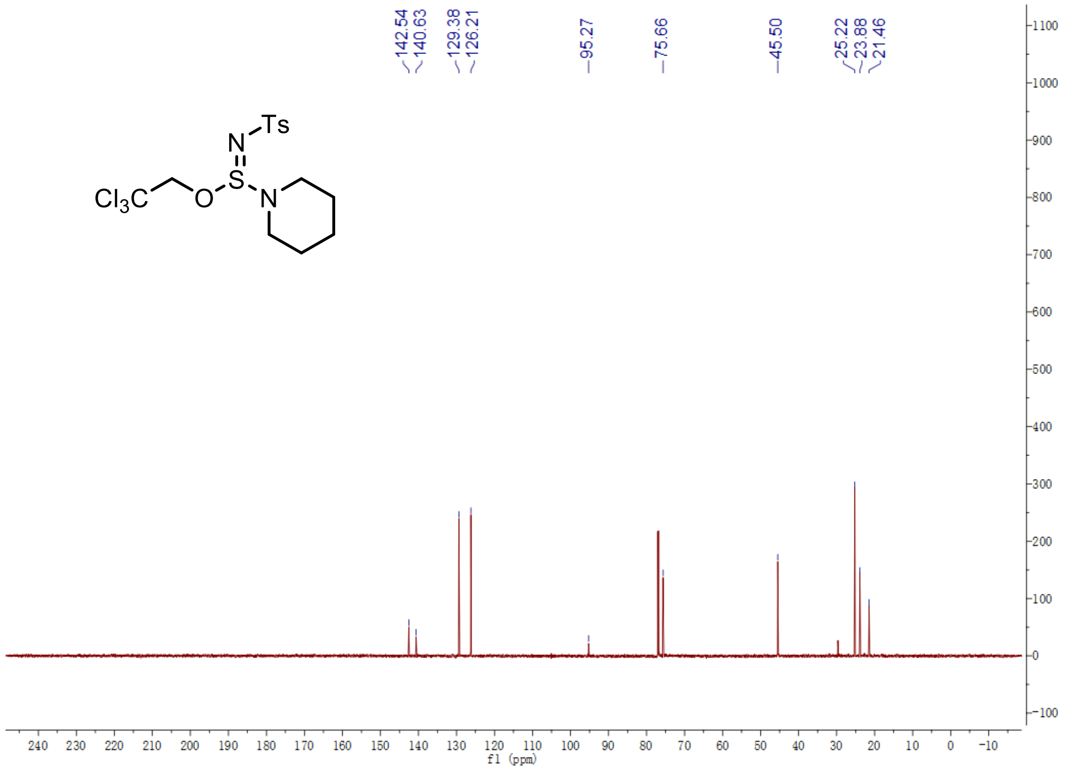

Supplement: SC-015-D4SC00500G-s001 [file SC-015-D4SC00500G-s001.pdf]
